# A General Strategy for the Synthesis of Jerangolids Enabled by π‐allyl Stille Coupling

**DOI:** 10.1002/chem.70928

**Published:** 2026-04-06

**Authors:** Janick Schug, Bernd Morgenstern, Johann Jauch

**Affiliations:** ^1^ Organic Chemistry II Saarland University Saarbrücken Germany; ^2^ B. Morgenstern, Service Center X‐Ray Diffraction Saarland University Saarbrücken Germany

**Keywords:** [1,4]‐anionotropic alkynyl shift, jerangolid, stannatrane, total synthesis, π‐allyl Stille coupling

## Abstract

Jerangolids are polyketide natural products with potent antifungal activity and low mammalian toxicity, originally isolated from *Sorangium cellulosum* So ce 307. Their structure, consisting of a δ‐lactone and a pyran unit connected by a chiral skipped 1,4‐diene, presents a significant synthetic challenge. Herein, we describe a general, modular strategy that enables the synthesis of all naturally occurring jerangolids and their stereoisomers, including the still elusive jerangolid H. The lactone fragment was built through a vinylogous Mukaiyama aldol reaction, with the hydroxymethyl group at C2 introduced in one step by Stille coupling with a novel *p*‐methoxybenzyloxymethyl (PMBM) stannatrane. The stereocenter at both C14 and C15 was generated via deoxygenation of a tertiary alcohol involving a [1,2]‐H‐shift. Using a highly modular approach recently developed by our group, the jerangolid framework was assembled in a single step through enantioselective π‐allyl Stille coupling of the two key fragments. Synthesis and comparison of both C14 epimers allowed us to unambiguously establish the total configuration of jerangolid H.

## Graphical abstract

A general strategy for the synthesis of jerangolids is established, featuring a late‐stage π‐allyl Stille coupling of two advanced, highly customizable building blocks to form the skipped diene core. The approach provides access to all naturally occurring jerangolids, including jerangolid H, whose configuration is confirmed by NMR analysis.

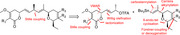

## 1 Introduction

**FIGURE 1:**
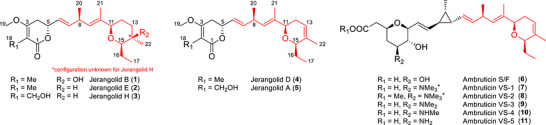
Structure of all naturally occurring jerangolids and ambruticins with the supposed pharmacophore marked in red.

Jerangolids are a class of polyketides first isolated in 1995 by Höfle et al. from the myxobacterium *Sorangium cellulosum* So ce 307 [1, 2]. Their structure features a chiral 1,4‐skipped diene connected to a δ‐lactone and a pyran substituent (Figure 1). They share a major part of their structural motif, which is assumed to be the pharmacophore responsible for biological activity, with the ambruticins, another class of polyketides first isolated from *Polyangium cellulosum* var. *fulvum* and *S. cellulosum* So ce 10 by Strandtmann et al. [3, 4, 5, 6, 7, 8, 9]. Similar to ambruticins, jerangolids display potent antifungal activity against a variety of pathogens, including *Mucor hiemalis*, *Trichosporon terrestre*, and *Trichoderma hamata* with minimal inhibitory concentrations (MIC) in the range of 0.07–7.0 mg mL^−1^ [2]. Both classes target the high osmolarity glycerol (HOG) signaling pathway in histidine kinase 1 (Hik1) expressing cells, leading to an intracellular accumulation of free fatty acids and glycerol, ultimately resulting in cell death [10, 11, 12, 13, 14]. Since this pathway is absent in mammals, jerangolids represent promising candidates for the development of novel antifungal drugs due to their remarkably low toxicity.

To date, total syntheses of all naturally occurring jerangolids, except jerangolid H (**3**), including some truncated analogues [15, 16, 17, 18], have been reported [19, 20, 21, 22]. Markó et al. achieved the first synthesis of jerangolid D (**4**) in 22 steps with a 6.1% yield in the longest linear sequence (LLS) [19]. Hanessian et al. subsequently synthesized jerangolid A (**5**, 23 steps, 1.9% LLS) [20], followed by Hahn et al. with jerangolid E (**2**, 23 steps, 4.0% LLS) [21], and most recently, our group completed the synthesis of jerangolid B (**1**, 20 steps, 5.7% LLS) [22].

**SCHEME 1:**
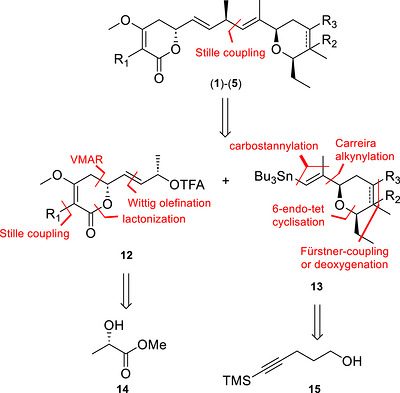
Retrosynthetic analysis of the jerangolids.

While previous strategies relied on olefination reactions to construct the skipped diene core from three building blocks, we have recently shown that this motif can be accessed in a single step via enantioselective sp^3^–sp^2^ Stille coupling (Scheme 1) [22]. We envisioned that our strategy could also be applicable to the synthesis of all jerangolids, including the still elusive jerangolid H (**3**).Retrosynthetically, the jerangolids can be disconnected at C8‐C9 to a trifluoroacetate **12** and a vinylstannane **13**. For the synthesis of Jerangolid A (**5**) and H (**3**), we planned to install the hydroxymethyl group at C2 in **12** in a single step as a PMB‐protected ether via homologation of an α‐iodolactone through Stille coupling. The stereocenter at C14 required for jerangolid E (**2**) and H (**3**) can be generated by deoxygenation of the corresponding tertiary alcohol previously employed in our synthesis of jerangolid B (**1**). The olefin at C13–C14 required for Jerangolid A (**5**) and D (**4**) can be accessed through a Kochi‐Fürstner coupling of the corresponding vinyl triflate, obtained from an earlier intermediate within the same strategy. This ultimately leads to the same starting materials L‐(−)‐lactate (**14**) and 5‐trimethylsilyl‐pent‐4‐yn‐1‐ol (**15**) for all the here‐mentioned jerangolids.

## 2 Results and Discussion

**SCHEME 2:**
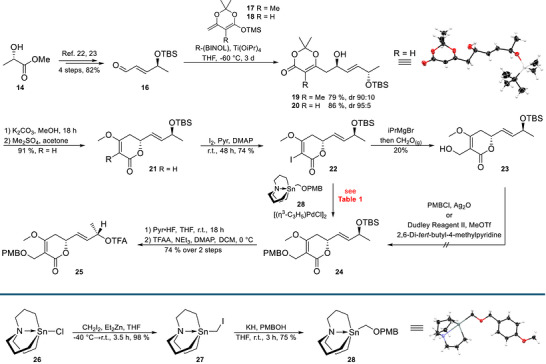
Synthesis of lactone **25** and *p*‐methoxybenzyloxy methyl stannatrane **28** (for details see Supporting Information).

Construction of the lactone building block started from the aldehyde **16**, which was synthesized in four steps starting from L‐(−)‐lactate (**14**) in 82% yield as previously described (Scheme 2) [22, 23]. Next, the aldehyde **16** was subjected to vinylogous Mukaiyama aldol reaction (VMAR) [24]. We found that silylketene acetal **18**, lacking the methyl substituent at C2, delivered the desired allylic alcohol **20** in 86% yield with a highly improved diastereoselectivity of 95:5 compared to **17**, delivering **19** in only 90:10 *dr* [22]. As previously discussed [22], the aldehyde forms a mismatched pair with R‐(BINOL), which reduces overall stereoselectivity. The additional decrease observed for the C2‐substituted silylketene acetal likely stems from steric interactions between its methyl group and the chiral ligand. These results indicate that C2‐substituted silylketene acetals are generally challenging substrates for this reaction class.

Subsequent one‐pot sequence of cyclisation with K_2_CO_3_ in MeOH and methylation with Me_2_SO_4_ in acetone delivered the desired lactone **21** in 91% yield [25]. For the synthesis of Jerangolid A (**5**) and H (**3**), we initially planned to introduce the hydroxymethyl group at C2 via halogen‐metal exchange and reaction with formaldehyde, first employed by Hanessian et al. [20] α‐Iodination afforded the vinyl iodide **22** in 74% yield. Unfortunately, even after extensive experimentation, subsequent halogen‐metal exchange and reaction with freshly cracked formaldehyde delivered the desired α‐hydroxymethyl lactone **23** in only 20% yield, with the main product being the dehalogenated lactone **21** [26]. Further attempts to introduce the PMB group using PMBCl/Ag_2_O at 50°C in DMF or Dudley reagent II resulted in complete decomposition of the starting material [27, 28], likely due to the inherent instability of the lactone fragment under either basic or Lewis acidic conditions [29, 30, 31, 32] Because of this instability, protecting the hydroxymethyl group as a PMB ether was crucial, as this is one of the few groups that can be cleaved orthogonally under neutral conditions using DDQ. We therefore had to devise a strategy that allows us to introduce a *p*‐methoxybenzyloxy methyl (PMBM) group in a single step.

Our initial attempt to introduce the PMBM group via Stille coupling of **22** using tributyl[(4‐methoxyphenyl)methyl] stannane resulted either in no conversion or complete decomposition (Table 1, entries 1 and 2) [33, 34, 35, 36]. As α‐alkoxy stannanes are known to transmetallate only sluggishly [37], we reasoned that this step in the catalytic cycle was inhibited. In such cases, the transmetallation rate can often be enhanced by the addition of fluoride salts [38, 39], which generate a pentacoordinated tin species, thereby weakening the Sn─C bond [40, 41]. However, the addition of 2.0 equiv. of KF resulted in complete decomposition of the starting material, most likely due to side reactions involving the TBS ether (Table 1, entry 2).

**TABLE 1:** Optimization for the synthesis of lactone **24** via Stille coupling (see Table 1)^a^ (for details see Supporting Information).

	Conditions^a^	Yield (%) 24 (21)^b^
1^c^	5 mol% Pd(PPh_3_)_4_, 10 mol% CuI, THF, 80°C, 45 min	‐^d^
2^c^	5 mol% Pd(PPh_3_)_2_Cl_2_, 2.0 equiv. KF, THF, 74°C, 30 min	‐^e^
3	5 mol% Pd(PPh_3_)_4_, THF, 66°C, 30 min	36 (18)
4	5 mol% Pd(dppf)Cl_2_, THF, 66°C, 30 min	34 (13)
5	5 mol% [(η^3^‐C_3_H_5_)PdCl]_2_, 40 mol% PCy_3_, toluene, 60°C, 10 min	50 (18)
6	5 mol% [(η^3^‐C_3_H_5_)PdCl]_2_, 20 mol% *t*Bu_2_MePHBF_4_, toluene, 58°C, 10 min	‐^e^
7	5 mol% [(η^3^‐C_3_H_5_)PdCl]_2_, 20 mol% Ad_3_P, toluene, 60°C, 10 min	39 (22)
8	5 mol% [(η^3^‐C_3_H_5_)PdCl]_2_, 20 mol% Ad_2_ *n*BuP, toluene, 58°C, 10 min	82 (6)

^a^ Reactions were performed in a microwave at a 0.25–1.00 mmol scale using 1.1 equiv. of p‐methoxybenzyl methyl stannatrane (**28**).

^b^ Yield of **21** was determined via 1H‐NMR.

^c^ Tributyl[(4‐methoxyphenyl)methyl] stannane was used instead [33, 34, 35, 36].

^d^ No reaction occurred.

^e^ Complete decomposition of lactone **22**.

An alternative strategy to selectively weaken the Sn─C bond and therefore improve the transmetallation rate is achieved by internal coordination using stannatranes (Scheme 2) [42, 43, 44, 45, 46, 47, 48]. In their seminal work, Vedejs et al. demonstrated that even alkyl groups, which are typically the most challenging substrates, can be selectively transmetallated under mild conditions [44, 49]. The required stannatrane **28** was generated in two steps starting from commercial 5‐chloro‐1‐aza‐5‐stannabicyclo[3.3.3.]undecane (**26**). Iodomethylation with CH_2_I_2_ and Et_2_Zn furnished **27** in 98% yield, and subsequent reaction with PMBOH using KH delivered stannatrane **28** in 75% yield as a crystalline solid [50, 51].

Stille coupling of vinyl iodide **22** using stannatrane **28** and 5 mol% Pd(PPh_3_)_4_ under microwave irradiation afforded the desired lactone, albeit in only 36% yield, accompanied by substantial amounts of **21** as a side product (Table 1, entry 3). Changing to Pd(dppf)Cl_2_ led to similar results (Table 1, entry 4). As the vinyl iodide **22** itself is a difficult substrate owing to the high electron density at C2, we concluded that under these conditions, the rate‐limiting step is no longer the transmetallation but the oxidative addition. Consequently, strongly donating ligands such as trialkylphosphines are expected to accelerate the oxidative addition step [52], thereby increasing yields. Sterically demanding phosphine ligands are also able to lower the energy barrier of the reductive elimination by promoting the formation of a three‐coordinate Pd species (53, 54, 55).

As anticipated, switching to 5 mol% [(η^3^‐C_3_H_5_)PdCl]_2_ together with 40 mol% PCy_3_ as a sterically demanding, electron‐rich ligand in toluene increased yields to an acceptable 50% (Table 1, entry 5). Using toluene as a solvent provided the additional advantage that the formed stannatrane iodide side product crystallized out of solution at room temperature and could be recovered in near‐quantitative yield via simple filtration, while also serving as a convenient indicator of reaction progress. By further increasing sterical demand, we were able to identify Ad_2_ *n*BuP as the optimal ligand affording the desired lactone **24** in an exceptional 82% yield (Table 1, entry 8). Any further increase in sterical demand, such as in the use of Ad_3_P or *t*Bu_2_MePHBF_4_, proved to be detrimental, leading to either decreased yield or complete decomposition (Table 1, entries 6 and 7) [56]

Deprotection of compound **24** had to be done using Pyr•HF, as even slightly Lewis acidic conditions, such as 2 mol% of Bi(OTf)_3_ in water/MeCN, additionally led to the formation of PMB cleaved product [57]. Final trifluoroacylation delivered the desired lactone **25** in 74% yield over two steps (Scheme 2).

**SCHEME 3:**
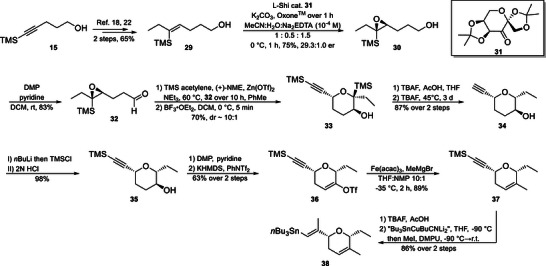
Synthesis of pyrane **38** (for details see Supporting Information).

Next, we turned our focus to the synthesis of the pyrane building block for Jerangolid A (**2**) and D (**3**) (Scheme 3). The synthesis started with the preparation of alkene **29** in two steps from commercially available 5‐trimethylsilyl‐pent‐4‐yn‐1‐ol (**15**), as previously described by our group [18, 22]. Enantioselective epoxidation with the L‐fructose‐derived L‐Shi catalyst **31** afforded epoxide **30** in 75% yield (er = 96.5:3.5) [58, 59]. Subsequent Dess‐Martin oxidation and Carreira alkynylation with TMS acetylene and (+)‐NME [60, 61], with the aldehyde being added slowly over 10 h, provided the corresponding propargylic alcohol in a 10:1 dr. Intramolecular BF_3_•OEt_2_ catalyzed 6‐*endo*‐tet epoxide opening delivered pyrane **33** as a single diastereomer in 64% yield after chromatographic purification. For further discussion of this epoxide opening reaction, see our previous paper [22].

To install the C14 methyl‐substituted alkene, both TMS groups had to be cleaved first while the C14 hydroxy group was still present, as deprotection at C15 does not proceed without the adjacent OH group [62]. Therefore, stepwise deprotection delivered the secondary alcohol **34** in 87% yield. Subsequent double TMS protection and selective alcohol deprotection sequence then provided the TMS‐protected alkyne **35** in near‐quantitative yield. A two‐step sequence comprising oxidation to ketone and selective deprotonation at C13 with KHMDS, followed by reaction with PhNTf_2_, delivered vinyltriflate **36** in 68% yield.

Attempts to couple vinyl triflate **37** with Me_2_CuLi provided high conversion under various conditions, but were hardly reproducible, often furnishing chromatographically inseparable mixtures of **37** with an unsubstituted alkene side product. Best yield was achieved via Kochi–Fürstner coupling using MeMgBr and Fe(acac)_3_ in a mixture of THF:NMP 10:1, providing alkene **37** in 89% yield [63, 64]. Subsequent deprotection and carbostannylation gave vinyl‐stannane **38** in 86% yield over two steps [65]

reproducible, often furnishing chromatographically inseparable mixtures of **37** with an unsubstituted alkene side product. Best yield was achieved via Kochi–Fürstner coupling using MeMgBr and Fe(acac)_3_ in a mixture of THF:NMP 10:1, providing alkene **37** in 89% yield [63, 64]. Subsequent deprotection and carbostannylation gave vinyl‐stannane **38** in 86% yield over two steps [65].

**SCHEME 4:**
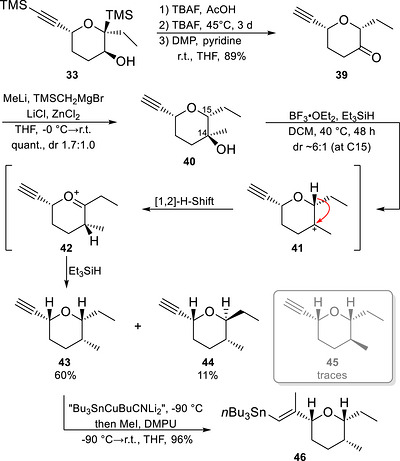
Synthesis of 14‐*epi* pyrane 46 (for details see Supporting Information).

The only building block left to be synthesized was the vinylstannane needed for the synthesis of Jerangolid E (**2**) and H (**3**) (Scheme 4). Synthesis began again with pyrane **33**, in which the C14 methyl substituent was installed via a three‐step sequence comprising double TMS deprotection, oxidation to the ketone **39**, and methyl addition following the procedure of Hatano et al. [66, 67, 68, 69], furnishing the tertiary alcohol **40** in quantitative yield as a separable 1.7:1 diastereomeric mixture.

To obtain the correct configuration at C14 in Jerangolid E (**2**) and H (**3**), the tertiary alcohol **40** had to be stereoselectively deoxygenated. As a tertiary carbenium is formed as an intermediate, the initial configuration at C14 was inconsequential in this step. Therefore, the epimeric mixture of **40** was reacted with BF_3_•OEt_2_ and Et_3_SiH for 48 h at 40°C [70], providing a separable 6:1 mixture of C15 epimers **43** and **44** in 71% overall yield, with the desired product **45** only detected in trace amounts. The absolute configuration of both products was unambiguously confirmed by NOESY experiments. The formation of the C15 diastereomer **45** can be rationalized by the generation of the tertiary carbocation **41**, which undergoes a [1,2]‐hydride shift to give the resonance‐stabilized oxacarbenium ion **42**, which is then reduced by Et_3_SiH from either face at C15, ultimately affording both epimers. This outcome turned out to be somewhat serendipitous, as it enabled the selective preparation of 14‐*epi* stannane **46** via subsequent carbostannylation in 96% yield.

**SCHEME 5:**
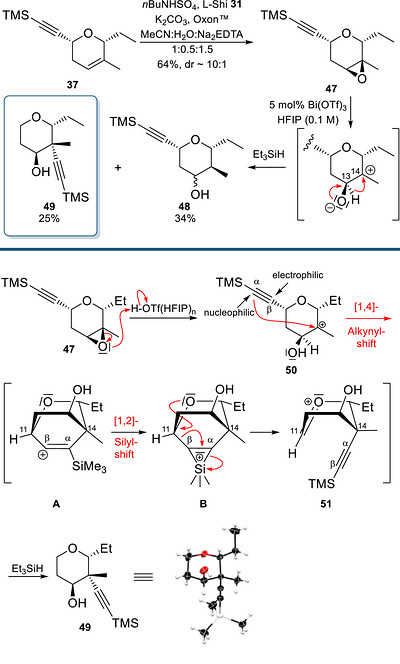
Attempted synthesis of pyrane 43 resulting in the [1,4]‐ alkynyl shift product 49 (for details see Supporting Information).

In an effort to exploit the [1,2]‐hydride shift, we stereoselectively converted alkene **37** into the corresponding epoxide using L‐Shi catalyst **31**, affording **47** in 64% yield and 10:1 dr (Scheme 5). In the presence of a Lewis acid, the epoxide **47** was expected to undergo Meinwald‐rearrangement, yielding a secondary alcohol upon reduction of the intermediate ketone. Subsequent deoxygenation of alcohol **48** would then furnish the desired compound.

Accordingly, epoxide **47** was treated following the procedure described by Lebœuf et al. [71], with two equivalents of Et_3_SiH and 5 mol % Bi(OTf)_3_ in HFIP at 0°C for 20 min, affording 34% of the target compound **48** as a 2.4:1.0 mixture of its two C13 epimers after chromatography. The low selectivity with regard to the secondary alcohol **48** can be explained by stereospecific epoxide opening by Bi(OTf)_3_, stereospecific 1,2‐Hydride shift from C13 to C14, and reduction of the resulting carbonyl group with low stereoselectivity to the secondary alcohol (Scheme 5).

In addition, 25% of an unknown crystalline compound was isolated and identified by x‐ray crystallography as an [1,4]‐alkynyl shift product **49**, in which the alkynyl substituent originally attached to C11 had migrated to C14. The formation of product **49** can be rationalized mechanistically through protonation of epoxide **47** by the in situ‐generated trifluoromethanesulfonic acid, leading to the tertiary carbocation **50** (Scheme 5). The pyrane can then adopt a boat‐like conformation, allowing the nucleophilic α‐C in the TMS‐ethynyl group (α‐silyl effect) to attack the tertiary carbocation to form the TMS‐vinyl cation **A**, stabilized by the β‐silyl effect. **A** undergoes a 1,2‐silyl shift via intermediate **B** to yield the oxacarbocation **51**. The latter is finally reduced by Et_3_SiH to pyrane **49**. Although, to the best of our knowledge, such a 1,4‐TMS ethinyl shift has not yet been described in the literature, the proposed mechanism is fully consistent with the known alpha and beta silyl effects and analogous reactions of vinylsilanes [72, 73, 74, 75, 76]. Due to their low tendency to migrate, such alkynyl shifts require either harsh conditions or prior alkyne activation, for example, by complexation with Au(III) or Co_2_(CO)_8_. [77, 78]. Notably, we observed this rearrangement exclusively in HFIP using Et_3_SiH, with no reaction occurring even after 2 d when NaCNBH_3_ or alternative solvents were used.

Attempts to perform the reaction of **47** to **48** with BF_3_•OEt_2_ and Et_3_SiH in DCM at −78°C led to the formation of a dimeric side product. As no efficient means to prepare **48** could be found, this strategy was ultimately abandoned.

**SCHEME 6:**
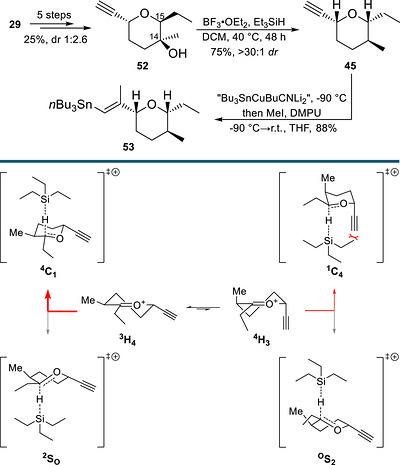
Synthesis of pyrane **45** and explanation for the encountered stereoselectivity (for details see Supporting Information).

Despite this setback, we remained committed to the idea of exploiting the [1,2]‐hydride shift. The rapid hydride migration, likely responsible for the negligible formation of the desired product **45**, suggested that deoxygenation of the *S*‐configured C15 epimer of **40** would allow us to obtain the correct configuration at C14. Reduction of the resulting oxacarbenium ion at C15 would then be expected to provide a separable mixture of both epimers. Accordingly, the required pyrane **52** was prepared in five steps starting from alkene **29** in 25% overall yield as a 1:2.6 diastereomeric mixture in an analogous way to **40** (Scheme 6). Subsequent deoxygenation provided the desired pyrane **45** almost exclusively, with an unexpectedly high diastereoselectivity of  > 30:1 at C15. Final carbostannylation led to the stannane **53** in 88% yield.

This surprising and somewhat counterintuitive selectivity in the reduction of the oxacarbenium ion at C15 can be easily rationalized by the energy differences of the conformers formed in the transition state [79, 80]. After the [1,2]‐hydride shift, the oxacarbenium ion exists in a dynamic equilibrium between the two half‐chair conformations **^3^H_4_** and **^4^H_3_** [81]. According to the Curtin–Hammett principle, the position of this equilibrium is not expected to influence the diastereoselectivity, as the barrier for interconversion between conformers is low, allowing rapid equilibration. Hydride transfer from triethylsilane to the **^3^H_4_** conformer from the Si face leads to an energetically favorable **^4^C_1_** conformer in the transition state, in which the substituents at C14 and C11 occupy equatorial positions and steric interactions with the ethyl groups of the silane are minimized. In contrast, hydride transfer to the **^4^H_3_** conformer from the Re face leads to an energetically less favorable **^1^C_4_** conformer in the transition state, as now both substituents are axial and additional steric repulsion arises between the alkynyl substituent at C11 and triethylsilane. The alternative twist‐boat transition states **^2^S_O_** and **°S_2_**, formed through Re attack on **^3^H_4_** or Si attack on **^4^H_3_**, respectively, are typically substantially higher in energy than the chair‐like transition states and are therefore not expected to contribute [80].

This would also explain the less pronounced diastereoselectivity observed for epimer **40**. In this case, the methyl substituent would be axial at C14 in the **^4^C_1_** conformer and equatorial in the **^1^C_4_** conformer. Although the **^4^C_1_** conformer remains preferred due to the dominant steric repulsion of the alkynyl substituent at C11 with triethylsilane in the **^1^C_4_** conformer, the energy difference between these two transition states is expected to be significantly smaller, thus resulting in low stereoselectivity.

**SCHEME 7:**
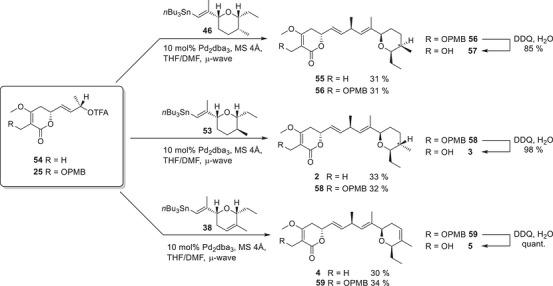
Synthesis of jerangolid A (**5**), D (**4**), E (**2**), H (**3**), as well as 14‐*epi* jerangolid E (**55**) and 14‐*epi* jerangolid H (**57**) via π‐allyl Stille coupling. Reactions were conducted using 10 mol% Pd_2_dba_3_, MS 4 Å in THF:DMF 2:1 under pulsed microwave irradiation (40 W, 1 min of irradiation followed by 1 min cooldown, repeated for up to 10 cycles) (for details see Supporting Information).

With all building blocks successfully synthesized, the stage was set for the π‐allyl Stille coupling (Scheme 7) [22, 82, 83, 84]. As discussed in our previous publication, the configuration at C8 is exclusively determined by the starting material, since epimerization of unsymmetrical 1,3‐disubstituted allylic substrates such as **25** via an η^3^–η^1^–η^3^ pathway is not possible. In our case, inversion of configuration at C8 is expected, as transmetallation of the vinylstannane to the Pd(II) center occurs prior to C–C bond formation.

Both lactone **25** or **54** were coupled with either vinylstannane 46 or 53 using catalytic amounts of Pd_2_dba_3_ under pulsed microwave irradiation in a 2:1 mixture of THF and DMF (Scheme 7). This provided Jerangolid E (2) and 14‐*epi* Jerangolid E (**55**), as well as the OPMB‐protected jerangolid H **58** and 14‐*epi* Jerangolid H **56**, in reproducible yields of 31%–33% after chromatographic purification. Cleavage of the PMB protecting group using DDQ in a 20:1 mixture of DCM and H_2_O finally furnished jerangolid H (**3**) in 85% as well as 14‐*epi* jerangolid H (**57**) in near quantitative yield. By comparison of the NMR data of our synthetic 14‐*epi*‐jerangolid H (**57**) and jerangolid H (**3**) with those of natural jerangolid H (**3**), we could unambiguously prove that C14 is also *S* configured, as is the case for jerangolid E (**2**).

In the same manner, π‐allyl Stille coupling of either lactone **25** or **54** with vinylstannane 38 afforded jerangolid D (**4**) in 30% yield as well as jerangolid A (**5**) in 32% yield after deprotection using DDQ (Scheme 7).

## 3 Conclusion

In summary, we have demonstrated that the modular synthetic strategy previously developed for the synthesis of jerangolid B (1) is general and can be employed for the synthesis of all other naturally occurring jerangolids and stereoisomers thereof. Using this approach, we have successfully synthesized jerangolid A (**5**, 27 steps, 4.5% yield, 16 steps LLS), jerangolid D (**4**, 23 steps, 4.0% yield, 15 steps LLS), jerangolid E (**2**, 21 steps, 5.1% yield, 13 steps LLS), and jerangolid H (**3**, 24 steps, 4.8% yield, 14 steps LLS) as well as 14‐*epi* Jerangolid E (**55**) and 14‐*epi* Jerangolid H (**57**).

Key steps included the installation of the PMB protected hydroxymethyl group at C2 in a single step by Stille coupling using a novel *p*‐methoxybenzyloxymethyl (PMBM) stannatrane, formation of pyran **33** fragment through Carreira alkynylation of aldehyde 32 with subsequent cyclization, a stereoselective [1,2]‐H‐Shift generating both configurations at C14 and C15, the Kochi‐Fürstner coupling used for the synthesis of pyrane **37**, and finally stereoselective π‐allyl Stille coupling of the respective building blocks to stereoselectively generate the chiral skipped diene core motif in a single step.

With this highly flexible strategy established, further work will focus on systematic derivatization of the jerangolid framework, particularly at C2, to enable comprehensive structure–activity relationship (SAR) studies and the development of a diverse jerangolid analog library.

## Conflicts of Interest

The authors declare no conflicts of interest.

## Supporting information




**Supporting Information**: The authors have cited additional references within the Supporting Information [85, 86, 87, 88].

## Data Availability

The data that support the findings of this study are available free of charge at “https://chemistry-europe.onlinelibrary.wiley.com/doi/10.1002/chem.70928”, including experimental details, characterization data for all new compounds, ^1^H‐, ^13^C‐, and 2D‐NMR spectra, and crystallographic data. Deposition numbers 2520695, 2520696, 2520699 contain the supplementary crystallographic data for this paper. This data can be obtained free of charge via the joint Cambridge Crystallographic Data Centre (CCDC) and Fachinformationszentrum Karlsruhe. The raw NMR data are available upon request from the corresponding author or on nmrXiv at https://doi.org/10.57992/NMRXIV.P164.
