# Supplementary material for: A General Strategy for the Synthesis of Jerangolids Enabled by π‐allyl Stille Coupling
Source: Chemistry. 2026 Apr 6;32(23):e70928. doi: 10.1002/chem.70928 (PMC13282915; doi:10.1002/chem.70928)
Supplement: Supplementary file 1 — Supporting Information: The authors have cited additional references within the Supporting Information [85, 86, 87, 88]. [file CHEM-32-e70928-s001.pdf]

Supporting Information
©Wiley-VCH 2025
69451 Weinheim, Germany

A General Strategy for the Synthesis of Jerangolids Enabled by π-allyl Stille Coupling

Janick Schug,[a] Bernd Morgenstern,[b] Johann Jauch*[a]

[a] J. Schug, Prof. Dr. J. Jauch
Organic Chemistry II, Saarland University
66123 Saarbrücken, Germany
E-mail: j.jauch@mx.uni-saarland.de

[b] B. Morgenstern
Service Center X-ray Diffraction, Saarland University
66123 Saarbrücken, Germany

Table of Contents

Abbreviation list 3
Synthetic procedures 6
NMR spectra 56
X-Ray crystallographic data 122
Literature 156

Abbreviation list

[α]	optical rotation
Ac	acyl
Ar	aryl
BINOL	1,1’-Bi-2-naphthol
b.p.	boiling point
brsm	based on recovered starting material
dba	Dibenzylideneacetone
DCC	N,N’-Dicyclohexylcarbodimiide
DCM	Dichloromethane
DIBALH	Diisobutylaluminiumhydride
DIPA	Diisopropylamine
DMAP	4-Dimethylaminopyridine
DMF	N,N-Dimethylformamide
DMP	Dess-Martin periodinane
DMPU	N,N’-Dimethylpropylenurea
EDTA	Ethylenediaminetetraacetic acid
eq.	equivalents
Et	ethyl
HPLC	high performance liquid chromatography
HRMS	high resolution mass spectrometry
ImH	Imidazole
*i*Pr	isopropyl
LDA	Lithiumdiisopropylamide
Me	methyl
MS	molecular sieves
MTPA	α-Methoxy-α-trifluoromethylphenylacetic acid
MW	microwave
*n*Bu	*n*-butyl
*n*Hex	*n*Hexyl
NME	N-Methylephedrine
NMR	nuclear magnetic resonance
Ph	phenyl
PNB	paranitrobenzoyl
ppm	part per million
R_f	retention factor
r.t.	room temperature
sat.	saturated
TBAF	tetrabutylammoniumfluoride
TBS	*tert*-butyldimethylsilyl

TES	triethylsilyl
Tf	triflyl
TFA	trifluoroacyl
TFAA	trifluoroacetic anhydride
THF	Tetrahydrofurane
TMS	trimethylsilyl
TLC	thin layer chromatography

General Information

All reactions were run under an inert atmosphere in dried (heat gun) glassware unless stated otherwise. Anhydrous solvents used in reactions were purchased in HPLC grade quality and were additionally freshly distilled under N_2 atmosphere. **THF**, **Et_2O** and **toluene** were distilled from sodium/benzophenone while **DCM** and **DIPA** were distilled from CaH_2. Solvents used for flash chromatography and for the extraction of aqueous phases were distilled prior to their use. All other chemicals were used as purchased in the highest available purity without further purification. All reactions were heated using an oil bath unless stated otherwise.

Silica gel for **flash chromatography** was purchased from Merck, Darmstadt, Germany (Silica 60, particle size 40–63 µm). **TLC** plates for reaction monitoring were purchased from Merck, Darmstadt, Germany ($Si60_{254}$ glass plates 50 × 100 mm). Visualization was achieved with a combination of UV detection (254 nm), iodine vapor and an anisaldehyde solution (85 mL MeOH, 10 mL HOAc, 5 mL concentrated H_2SO_4, 0.5 mL p-anisaldehyde) or $KMNO_4$ solution (12.5 g $KMNO_4$, 62.5 g, Na_2CO_3, 1.25 L H_2O)

Reversed-phase flash chromatography was performed using a Büchi Reveleris® Prep Chromatography System with Büchi FlashPure Select C18 30 µm spherical cartridges.

NMR spectra were recorded with a BRUKER AV II 400 NMR spectrometer (^1H = 400 MHz, ^{13}C = 100 MHz) or an AV 500 (^1H = 500 MHz, ^{13}C = 125 MHz). Chemical shifts (d) are given in ppm. Deuterated chloroform $CDCl_3$ was used as the solvent with its chemical shifts at $\delta(^1H)$ = 7.26 ppm and $\delta(^{13}C)$ = 77.0 ppm being used as the internal standard. Multiplicities of the splitting patterns were abbreviated as follows: s (singlet), d (doublet), tr (triplet), q (quartet), quint (quintet), sext (sextet), sept (septet), m (multiplet), b (broad). All coupling constants are given in the unit Hertz (Hz). Structural assignments were made with additional information from gCOSY, gHSQC-DEPT, and gHMBC experiments. NMR spetra were analyzed using MestReNova Version 14.2.0-26256 from Mestrelab Research S.L.

Melting points were measured with a BÜCHI150 melting point device and are uncorrected.

Chiral **high-performance liquid chromatography (HPLC)** analysis was performed on a Merck Hitachi system consisting of a Merck Hitachi pump L-7100, Merck Hitachi autosampler L-7250, Merck Hitachi column oven L-7300, Merck Hitachi UV detector L-7455 and a Merck Hitachi experimental part 50 interface D-7000. The chiral chromatography columns used for normal phase chromatography were CHIRALCEL OD-H (250 x 4.6 mm) and CHIRALCEL OB-H (250 x 4.6 mm) from DAICEL CHEMI-CAL INDUSTRIES.

Specific optical rotations for enantiomerically pure or enriched substances were measured on a 241 MC polarimeter from PERKIN-ELMER with a sodium vapor lamp (λ = 589.3 nm) and are given in 10^{-1} deg•cm^2•g^{-1}. The cuvettes used have a length of 1.0 dm. The concentrations c of the samples are given in g/100 mL. $CHCl_3$ used as solvent refers to $CHCl_3$ with amylene as stabilizer.

High-resolution mass spectra (HRMS) were measured at the Service Center for Mass Spectroscopy at Saarland University by Dr. Klaus Hollemeyer on a SolariX 7 Tesla MALDI/ESI/APPI/FTICR Imaging MS from Bruker Daltonics GmbH using a quadrupole mass analyzer.

Single crystal X-ray diffraction analysis was carried out by the service center for X-ray diffraction of Saarland University on a Bruker D8 Advance diffractometer.

Synthetic procedures

Methyl (S)-2-((*tert*-butyldimethylsilyl)oxy)propanoate S1

In a 250 mL round bottom flask under N_2 methyl (S)-2-hydroxypropanoate **9** (5.21 g, 50 mmol, 1.0 eq.) is dissolved in 50 mL anhydrous DCM and cooled to 0 °C. Imidazole (5.11 g, 75 mmol, 1.5 eq.) and DMAP (611 mg, 5.0 mmol, 0.1 eq.) are successively added to this solution followed by TBSCl (8.29 g, 55 mmol, 1.1 eq.). The cooling bath is removed and the resulting colorless suspension is stirred for 5 h. The suspension is quenched by adding sat. NH_4Cl solution. The phases are separated and the aqueous phase is extracted three times with 50 mL DCM. The combined organic phases are dried with $MgSO_4$. Filtration over a 2-3 cm pad of silica gel 60 and concentration in vacuo affords the product **S1** as a colorless oil (10.47 g, 48 mmol, 96%) which used without further need for purification.

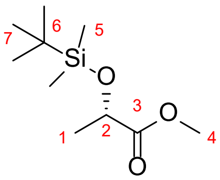

$C_{10}H_{22}O_3Si$
M = 218.37 g•mol^{-1}

TLC: R_f (pentane/Et_2O 1/1) = 0.82

^{1}H-NMR (400 MHz, $CDCl_3$, δ in ppm): 4.33 (q, J = 6.7 Hz, 1H, H2), 3.72 (s, 3H, H4), 1.40 (d, J = 6.7 Hz, 3H, H1), 0.90 (s, 9H, H7), 0.10 (s, 3H, H5), 0.07 (s, 3H, H5‘).

^{13}C-NMR (100 MHz, $CDCl_3$, δ in ppm): 174.7 (C3), 68.6 (C2), 52.0 (C4), 25.9 (C7), 21.5 (C1), 18.5 (C6), -4.8 (C5), -5.1 (C5‘).

The spectral data are in agreement with literature reports.[1]

Methyl (S)-4-((*tert*-butyldimethylsilyl)oxy)pent-2-enoate S2

An oven dried 2L three necked round bottom flask with an addition funnel and a N_2 inlet is charged with the TBS protected (S)-2-hydroxypropanoate **S1** (44.6 g, 200 mmol, 1.0 eq.). To this 400 mL of dry DCM are added and the solution is cooled down to below -70 °C (internal temperature) with a dry ice bath. Then DIBALH (0.9 M in DCM, 240 mL, 210 mmol, 1.05 eq.) is placed in the addition funnel and is subsequently added slowly to the reaction mixture over the course of 1 h while maintaining the internal temperature below -70 °C. The colorless mixture is stirred for an additional 30 min before 40 mL MeOH is added in two portions and stirred for another 30 min at below -70 °C. To the turbid mixture methyl 2-(triphenylphosphoranylidene)acetate (100.0 g, 300 mmol, 1.5 eq.) is added in one portion, the cooling bath is removed, and the resulting colorless suspension is allowed to reach room temperature while stirring for 1 h. After complete conversion the suspension is treated with a saturated K-Na-tartrate solution. After stirring for 30 min the solution is transferred into a separating funnel. The phases are separated and the aqueous phase is extracted 3x with 200 mL DCM. The combined organic phases are dried with $MgSO_4$ and filtrated and concentrated in vacuo affording 131 g (268% crude yield) of the title compound as a colorless solid with substantial amounts of triphenylphosphine oxide as the main contaminant.

To remove the triphenylphosphine oxide impurity the crude product is dissolved in 400 mL EtOH and treated with 100 mL of a freshly prepared 2.0 M solution of $ZnCl_2$ in EtOH. After stirring for 20 min the triphenylphosphine oxide completely precipitates out of solution and is filtered off. The filtrate is concentrated in vacuo and again dissolved in 300 mL Et_2O. The organic phase is washed twice with H_2O and dried with $MgSO_4$. Filtration and evaporation of the solvent affords pure methyl (S)-4-((*tert*-butyldimethylsilyl)oxy)pent-2-enoate **S2** (44.9 g, 184 mmol, 92%, separatable 1.5:1 Z:E mixture) as a colorless oil.

Z isomer:

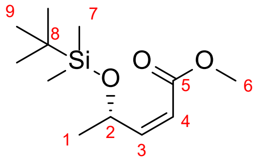

$C_{12}H_{24}O_3Si$
M = 244.41 g•mol^{-1}

TLC: R_f (pentane/Et_2O 15/1) = 0.42

^{1}H-NMR (400 MHz, $CDCl_3$, δ in ppm): 6.22 (dd, J = 11.7 Hz, 7.8 Hz, 1H, H3), 5.66 (dd, J = 11.7 Hz, 1.3 Hz, 1H, H4), 5.44 (dqd, J = 7.8 Hz, 6.3 Hz, 1.3 Hz, 1H, H2), 3.71 (s, 3H, H6), 1.25 (d, J = 6.3 Hz, 3H, H1), 0.88 (s, 9H, H9), 0.05 (s, 3H, H7), 0.03 (s, 3H, H7‘).

^{13}C-NMR (100 MHz, $CDCl_3$, δ in ppm): 166.4 (C5), 155.3 (C3), 116.5 (C3), 65.6 (C2), 51.4 (C6), 26.0 (C9), 23.7 (C1), 18.3 (C8), -4.6 (C7), -4.7 (C7‘).

E isomer:

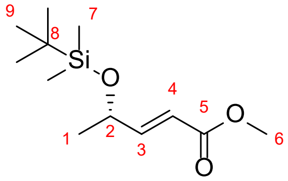

$C_{12}H_{24}O_3Si$
M = 244.41 g•mol^{-1}

TLC: R_f (pentane/Et_2O 15/1) = 0.25

^{1}H-NMR (400 MHz, $CDCl_3$, δ in ppm): 6.94 (dd, J = 15.5 Hz, 4.0 Hz, 1H, H3), 6.00 (dd, J = 15.5 Hz, 1.8 Hz, 1H, H4), 4.46 (qdd, J = 6.5 Hz, 4.0 Hz, 1.8 Hz, 1H, H2), 3.74 (s, 3H, H6), 1.26 (d, J = 6.5 Hz, 3H, H1), 0.91 (s, 9H, H9), 0.07 (s, 3H, H7), 0.06 (s, 3H, H7‘).

^{13}C-NMR (100 MHz, $CDCl_3$, δ in ppm): 167.5 (C5), 152.4 (C3), 118.7 (C4), 67.8 (C2), 51.7 (C6), 26.0 (C9), 23.7 (C1), 18.4 (C8), -4.7 (C7).

The spectral data are in agreement with literature reports.[1]

(S)-4-((*tert*-butyldimethylsilyl)oxy)pent-2-en-1-ol S3

In an oven dried 1 L three necked round bottom flask with an addition funnel Methyl (S)-4-((*tert*-butyldimethylsilyl)oxy)pent-2-enoate **S2** (44.9 g, 184 mmol, 1.0 eq.) is dissolved in 370 mL of anhydrous DCM and cooled to -40 °C with a dry ice bath. DIBALH (380 mL, 1.0 M in hexane, 376 mmol, 2.05 eq.) is added into the addition funnel and slowly added dropwise to the reaction mixture over the course of 2 h. After complete addition the dry ice bath is exchanged for an ice bath and the reaction is stirred for an additional hour. The reaction is quenched slowly by dropwise addition of NH_4Cl (Caution! gas evolution with an induction period) with ice bath cooling resulting in a colorless slurry. Then 300 mL of a saturated K-Na-tartrate solution is added and the colorless slurry is stirred for 2 h. The resulting colorless biphasic mixture is transferred into a separatory funnel, the phases are separated and the aqueous phase is extracted 3x with 200 mL Et_2O. The combined organic phases are dried with $MgSO_4$. Filtration over a short pad of celite and evaporation of the solvent under vacuum affords (S)-4-((*tert*-butyldimethylsilyl)oxy)pent-2-en-1-ol **S3** (36.7 g, 170 mmol, 92%) as a colorless oil, which was used without further need for purification.

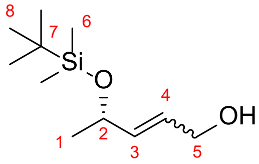

$C_{11}H_{24}O_2Si$
M = 216.40 g•mol^{-1}

TLC: R_f (pentane/Et_2O 3/1) = 0.24

Z isomer: ^{1}H-NMR (400 MHz, $CDCl_3$, δ in ppm): 5.60-5.48 (m, 2H, H3+H4), 5.48 (quin, J = 6.5 Hz, 1H, H2), 4.29-4.23 (m, 1H, H5), 4.18-4.10 (m, 1H, H5‘), 1.84 (brs, 1H, OH), 1.22 (d, J = 6.3 Hz, 3H, H1), 0.89 (s, 9H, H8), 0.07 (s, 3H, H6), 0.06 (s, 3H, H6‘).

^{13}**C-NMR** (100 MHz, $CDCl_3$, δ in ppm): 137.1 (C3), 127.2 (C4), 66.7 (C2), 59.1 (C5), 26.0 (C8), 25.0 (C1), 18.4 (C7), -4.5 (C6), -4.6 (C6‘).

E isomer: ^{1}**H-NMR** (400 MHz, $CDCl_3$, δ in ppm): 5.82-5.68 (m, 2H, H3+H4), 4.36-4.30 (m, 1H, H2), 4.29-4.23 (m, 2H, H5), 1.30 (brs, 1H, OH), 1.21 (d, J = 6.4 Hz, 3H, H1), 0.90 (s, 9H, H8), 0.06 (s, 3H, H6), 0.05 (s, 3H, H6‘).

^{13}**C-NMR** (100 MHz, $CDCl_3$, δ in ppm): 136.6 (C3), 127.4 (C4), 68.6 (C2), 63.4 (C5), 26.0 (C8), 24.5 (C1), 18.4 (C7), -4.5 (C6), -4.6 (C6‘).

The spectral data are in agreement with literature reports.[1]

(S,E)-4-((*tert*-butyldimethylsilyl)oxy)pent-2-enal 16

In a 500 mL Schlenk flask (S)-4-((*tert*-butyldimethylsilyl)oxy)pent-2-en-1-ol **S3** (8.66 g, 40 mmol, 1.0 eq.) is dissolved in 160 mL dry MeCN. Then $[Cu(MeCN)_4]OTf$ (151 mg, 0,4 mmol, 1 mol%), 4,4'-Dimethoxy-2,2'-bipyridyl (87 mg, 0,4 mmol, 1 mol%), TEMPO (63 mg, 0,4 mmol, 1 mol%) and DMAP (98 mg, 0,8 mmol, 2 mol%) are subsequently added, resulting in a dark brown solution. To the septum, an O_2 balloon fitted with a syringe reaching into the solution is connected. The mixture is then degassed and refilled with O_2 three times and stirred for 2 h while maintaining constant bubbling. The color changes from brownish red to green and finally to blue indicating complete oxidation. The reaction mixture is stirred overnight to ensure complete cis-trans isomerization. After complete reaction 200 mL H_2O are added and the mixture is transferred into a separatory funnel. The phases are separated and the aqueous phase is extracted 3x with 100 mL Et_2O. The combined organic phases are washed additionally with saturated NaCl solution and dried with $MgSO_4$. Filtration through a 2-3 cm pad of silica gel 60 and evaporation of the solvent under vacuum affords pure (S,E)-4-((*tert*-butyldimethylsilyl)oxy)pent-2-enal **16** (8.30 g, 38.7 mmol, 97% E:Z >50:1) as a colorless oil which was used without further need for purification.

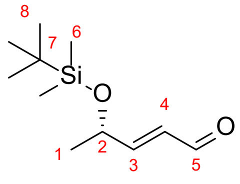

$C_{11}H_{22}O_2Si$
M = 214.38 g•mol^{-1}

TLC: R_f (pentane/Et_2O 2/1) = 0.55

$[\alpha]^{D}_{20}$ = +17.9 ° (c = 1.05, $CHCl_3$), Lit.: +17.9 (c = 1.07, $CHCl_3$)

^{1}**H-NMR** (400 MHz, $CDCl_3$, δ in ppm): 9.58 (d, J = 8.0 Hz, 1H, H5), 6.80 (dd, J = 15.5 Hz, 4.0 Hz, 1H, H3), 6.28 (ddd, J = 15.5 Hz, 8.0 Hz, 1.7 Hz, 1H, H4), 4.57 (qdd, J = 6.6 Hz, 4.0 Hz, 1.7 Hz, 1H, H2), 1.31 (d, J = 6.6 Hz, 3H, H1), 0.91 (s, 9H, H8), 0.08 (s, 3H, H6), 0.06 (s, 3H, H6')

^{13}**C-NMR** (100 MHz, $CDCl_3$, δ in ppm): 194.0 (C5), 161.1 (C3), 130.0 (C4), 67.9 (C2), 25.9 (C9), 23.5 (C1), 18.3 (C8), -4.7 (C6), -4.8 (C6')

The spectral data are in agreement with literature reports.

Trimethyl((2,2,5-trimethyl-4-methylen-4H-1,3-dioxin-6-yl)oxy)silane 18

In a dried 250 mL Schlenk flask freshly distilled DIPA (7.1 mL, 50 mmol, 1.2 eq.) is dissolved in dry THF (35 mL) and the solution is cooled to -78 °C with a dry ice bath. *n*BuLi (2.5 M in hexanes, 20 mL, 50 mmol, 1.2 eq.) is then added dropwise. The colorless solution is warmed to r.t. and stirred for 30 min before being cooled again to -78 °C. Then 2,2,6-trimethyl-4H-1,3-dioxin-4-one (5.94 g, 41.8 mmol, 1.0 eq.) dissolved in dry THF (15 mL) is

added to the solution over the course of 20 min and is stirred for 1 h at -78 °C. To the resulting yellow reaction mixture TMSCl (6.3 mL, 50 mmol, 1.2 eq.) is added dropwise and is stirred for another 3 h at -78 °C before warming to room temperature. The solvent is removed under reduced pressure and a heat dried micro distill (with a tared Schlenk tube as receiving flask) under constant N_2 stream is connected to the reaction flask. The receiving flask is connected to a high vacuum pump and cooled to 0 °C. Distillation of the crude product ($T_{b.p.}$ = 39-40 °C, 0.1 mbar, heating the product to above 50 °C leads to decomposition) affords ((2,2-dimethyl-4-methylen-4H-1,3-dioxin-6-yl)oxy)trimethylsilane **18** (7.92 g, 36.9 mmol, 88%) as a colorless oil.

The product can be stored for months at -20 °C under N_2 without decomposition.

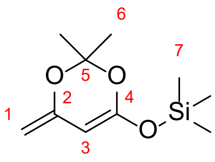

$C_{10}H_{18}O_3Si$
M = 214.34 g•mol^{-1}

^{1}H-NMR (400 MHz, $CDCl_3$, δ in ppm): 4.65 (s, 1H, H3), 4.07 (s, 1H, H1), 3.88 (s, 1H, H1'), 1.55 (s, 6H, H6), 0.27 (s, 9H, H7).

^{13}C-NMR (100 MHz, $CDCl_3$, δ in ppm): 153.5 (C4), 151.9 (C2), 102.7 (C5), 85.1 (C1), 76.8 (C3), 24.6 (C6), 0.4 (C7).

Trimethyl((2,2,5-trimethyl-4-methylen-4H-1,3-dioxin-6-yl)oxy)silane 17

The reaction is carried out as described for **18** using DIPA (16.9 mL, 120 mmol, 1.2 eq.), *n*BuLi in hexane (2.5 M, 48 mL, 120 mmol, 1.2 eq.), 2,2,5,6-Tetramethyl-4H-1,3-dioxin-4-one (15.6 g, 100 mmol, 1.0 eq.) in dry THF (70 mL), and TMSCl (14.0 mL, 110 mmol, 1.1 eq.). Distillation of the crude product ($T_{b.p.}$ = 59 °C, 0.6 mbar, heating the product to above 70 °C leads to decomposition) affords trimethyl((2,2,5-trimethyl-4-methylen-4H-1,3-dioxin-6-yl)oxy)silane **17** as a colorless liquid (17.73 g, 78.0 mmol, 78%). The product can be stored for months at -20 °C under N_2 atmosphere without decomposition.

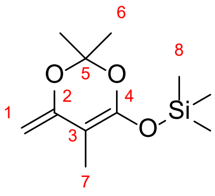

$C_{11}H_{20}O_3Si$
M = 228.36 g•mol^{-1}

^{1}H-NMR (400 MHz, $CDCl_3$, δ in ppm): 4.12 (d, J = 0.7 Hz, 1H, H1), 3.93 (d, J = 0.7 Hz, 1H, H1'), 1.66 (s, 3H, H7), 1.51 (s, 6H, H6), 0.24 (s, 9H, H8).

^{13}C-NMR (100 MHz, $CDCl_3$, δ in ppm): 154.4 (C2), 149.6 (C4), 101.5 (C5), 84.0 (C1), 81.9 (C3), 24.7 (C6), 9.5 (C7), 0.7 (C8).

6-((2R,5S,E)-5-((*tert*-Butyldimethylsilyl)oxy)-2-hydroxyhex-3-en-1-yl)-2,2-dimethyl-4H-1,3-dioxin-4-one 20

In an oven-dried Schlenk flask under an inert atmosphere, 400 mg CaH_2 and *R*-BINOL (2.78 g, 9.70 mmol, 0.5 eq.) are mixed and the flask is purged three times with inert gas. Dry THF (60 mL) is added and the suspension is stirred for 5 min before Ti(O*i*Pr)$_4$ (2.9 mL, 9.30 mmol, 0.5 eq.) is added dropwise, resulting in an orange suspension. The mixture is cooled to -60 °C using a cryostat and a solution of aldehyde **16** (4.13 g, 19.30 mmol, 1.0 eq.) in dry THF (20 mL) is added dropwise. After stirring for 30 min, a solution of silyl ketene acetal **18**

(6.21 g, 29.0 mmol, 1.5 eq.) in dry THF (20 mL) is added and the reaction is stirred at -60 °C for 3 d. The reaction is quenched by dropwise addition of saturated aqueous NH_4Cl (caution! vigorous gas evolution!) and stirred until a highly viscous yellow slurry forms. The suspension is filtered through a 2–3 cm pad of celite and washed twice with Et_2O (50 mL). The filtrate is transferred to a separatory funnel and the phases are separated. The aqueous phase is extracted three times with Et_2O (100 mL) and the combined organic phases are dried over $MgSO_4$. After filtration and removal of the solvent under reduced pressure the crude product is purified by flash chromatography (pentane/Et_2O 2:1 → 1:1 → 1:2 v:v) affording 6-((2R,5S,E)-5-((tert-Butyldimethylsilyl)oxy)-2-hydroxyhex-3-en-1-yl)-2,2-dimethyl-4H-1,3-dioxin-4-one **20** as colorless needle-shaped crystals (5.89 g, 16.50 mmol, 86%) with a diastereomeric ratio of 20.7:1.0.

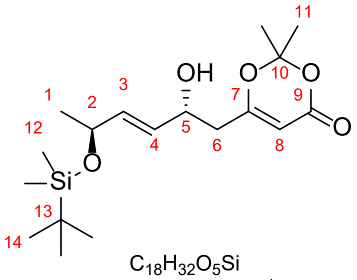

$C_{18}H_{32}O_5Si$
M = 356.53 g•mol^{-1}

TLC: R_f (pentane/Et_2O 1/1) = 0.20.

$[\mathbf{a}]^{D}_{20}$ = +16.1 (c = 1.08, $CHCl_3$).

$\mathbf{T}_{\mathbf{m.p.}}$ = 39-40 °C.

^{1}H-NMR (400 MHz, $CDCl_3$, δ in ppm): 5.77 (ddd, J = 15.5 Hz, 4.7 Hz, 0.5 Hz, 1H, H3), 5.66 (ddd, J = 15.4 Hz, 4.6 Hz, 1.2 Hz, 1H, H4), 5.32 (s, 1H, H8), 4.47-4.39 (m, 1H, H5), 4.35-4.27 (m, 1H, H2), 2.44 (d, J = 6.5 Hz, 2H, H6), 1.71 (brs, 1H, OH), 1.69 (s, 6H, H11), 1.20 (d, J = 6.4 Hz, 3H, H1), 0.89 (s, 9H, H14), 0.06 (s, 3H, H12), 0.04 (s, 3H, H12‘).

^{13}C-NMR (100 MHz, $CDCl_3$, δ in ppm): .168.5 (C7), 161.1 (C9), 137.0 (C3), 129.3 (C4), 106.8 (C10), 95.5 (C8), 69.3 (C5), 68.3 (C2), 41.7 (C6), 26.0 (C14), 25.6 (C11), 25.0 (C11‘), 24.4 (C1), 28.4 (C13), -4.5 (C12), -4.6 (C12‘).

HRMS: calculated for $C_{18}H_{31}O_5Si$ $[M-H]^-$: 355.19462, measured for $C_{18}H_{31}O_5Si$ $[M-H]^-$: 355.19664.

HPLC: Chiralcel OD-H, 1 mL/min, 25 °C, *n*Hex:*i*PrOH 95:5, 250 nm, t_{R1} = 12.82 min, t_{R2} = 17.25 min

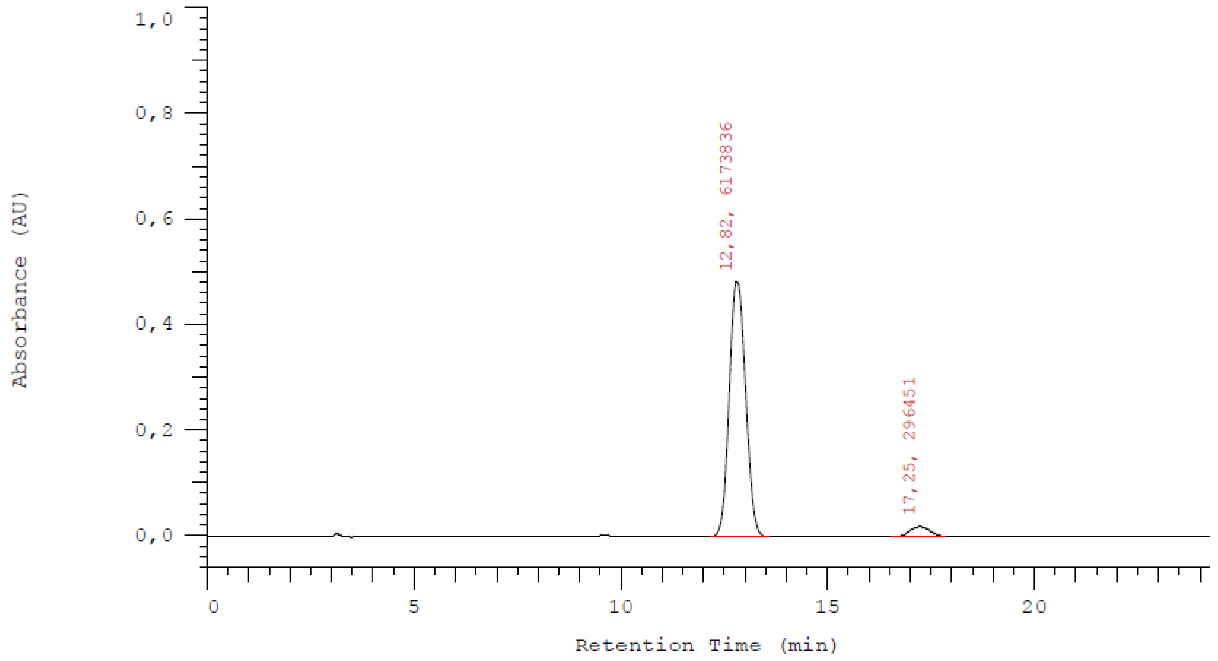

The absolute configuration of **20** was confirmed via X-ray crystal analysis.

peak	retention time [min]	area [AU•min]	area [%]	*dr*
1	12.82	6173836	95.4	20.7:1.0
2	17.25	296451	4.6	

6-((2R,5S,E)-5-((*tert*-Butyldimethylsilyl)oxy)-2-hydroxyhex-3-en-1-yl)-2,2,5-trimethyl-4H-1,3-dioxin-4-one 19

The reaction is carried out as described for **20** using CaH_2 (215 mg), (R)-BINOL (1.50 g, 5.25 mmol, 0.5 eq.) dissolved in dry THF (31 mL), Ti(O*i*Pr)$_4$ (1.55 mL, 5.25 mmol, 0.5 eq.), aldehyde **16** (2.24 g, 10.5 mmol, 1.0 eq.) and silyl ketene acetal **17** (3.60 g, 15.8 mmol, 1.5 eq.) dissolved in dry THF (11 mL). After workup the crude product is purified by flash chromatography (pentane:Et_2O 2:1 → 1:1 v:v) yielding 6-((2R,5S,E)-5-((*tert*-Butyl-dimethylsilyl)oxy)-2-hydroxyhex-3-en-1-yl)-2,2,5-trimethyl-4H-1,3-dioxin-4-one **19** (3.08 g, 8.32 mmol, 79%, 86% brsm) as a colorless oil with a diastereomeric ratio of 8.8:1.0.

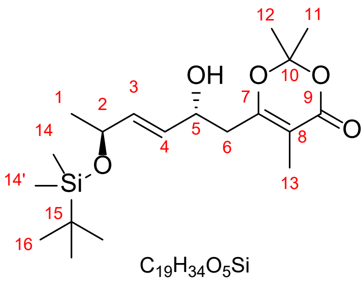

$C_{19}H_{34}O_5Si$
M = 370.56 g/mol

TLC: R_f (pentane/Et_2O 1/1) = 0.19

$[\alpha]^D_{20}$ = +15.3 (c = 0.98, $CHCl_3$)

^{1}H-NMR (400 MHz, $CDCl_3$, δ in ppm): 5.76 (ddd, J = 15.5 Hz, 4.5 Hz, 0.5 Hz, 1H, H3), 5.68 (ddd, J = 15.5 Hz, 6.2 Hz, 1.0 Hz, 1H, H4), 4.47-4.40 (m, 1H, H5), 4.36-4.28 (m, 1H, H2), 2.59 (dd, J = 14.1 Hz, 8.0 Hz, 1H, H6), 2.49 (dd, J = 14.1 Hz, 5.2 Hz, 1H, H6‘), 1.85 (s, 3H, H13), 1.66 (brs, 1H, OH), 1.67 (s, 3H, H12), 1.66 (s, 3H, H11), 1.20 (d, J = 6.4 Hz, 3H, H1), 0.89 (s, 9H, H16), 0.06 (s, 3H, H14), 0.05 (s, 3H, H14‘).

^{13}C-NMR (100 MHz, $CDCl_3$, δ in ppm): 162.7 (C9), 162.3 (C7), 136.9 (C3), 129.5 (C4), 105.1 (C10), 102.6 (C8), 70.0 (C5), 68.2 (C2), 38.9 (C6), 26.0 (C16), 25.7 (C12), 25.1 (C11), 24.4 (C1), 18.4 (C15), 10.6 (C13), -4.5 (C14), -4.6 (C14‘).

HRMS: calculated for $C_{19}H_{33}O_5Si$ $[M-H]^-$: 369.2103, measured for $C_{19}H_{33}O_5Si$ $[M-H]^-$: 369.2109.

HPLC: Chiralcel OD-H, 1 mL/min, 25 °C, *n*Hex:*i*PrOH 95:5, 250 nm, t_{R1} = 5.98 min, t_{R2} = 6.30 min.

The absolute configuration was determined via Mosher ester analysis in our previous work.[2]

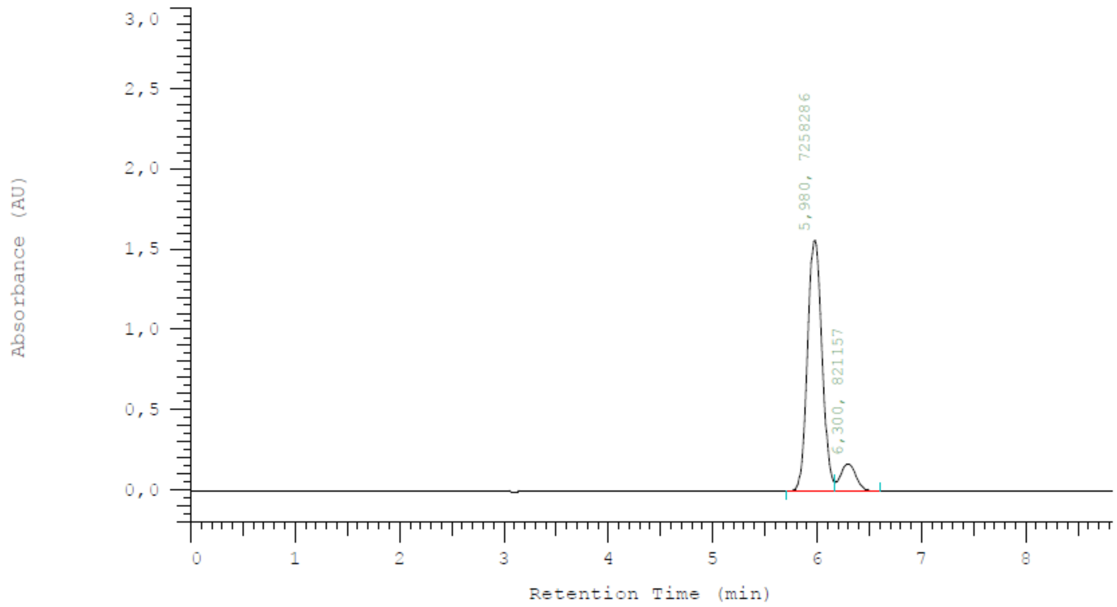

peak	retention time [min]	area [AU•min]	area [%]	*dr*
1	5.980	7258286	89.8	8.8:1.0
2	6.300	821157	10.2	

(R)-6-((S,E)-3-((*tert*-Butyldimethylsilyl)oxy)but-1-en-1-yl)-4-methoxy-5,6-dihydro-2H-pyran-2-one 21

In a 250 mL round bottom flask allyl alcohol **20** (10.34 g, 29.0 mmol, 1.0 eq.) is dissolved in MeOH (160 mL) and cooled to 0 °C with an ice bath. K_2CO_3 (8.02 g, 58.0 mmol, 2.0 eq.) is then added in one portion and the colorless suspension is stirred overnight while allowing it to warm to room temperature. The reaction mixture is concentrated under reduced pressure with a rotary evaporator until a highly viscous yellow resin is obtained. The resin is suspended in acetone (160 mL) and stirred vigorously before Me_2SO_4 (5.5 mL, 58.0 mmol, 2.0 eq.) is added dropwise to the suspension. After stirring for 2 h at room temperature, the orange suspension is quenched with a 1:9 mixture of NH_4OH/NH_4Cl (50 mL) and stirred for an additional 10 min. The biphasic mixture is transferred into a separatory funnel, the phases are separated and the aqueous phase is extracted three times with Et_2O (100 mL). The combined organic phases are washed once with saturated NH_4Cl solution and dried with $MgSO_4$. After filtration and removal of the solvent under reduced pressure, the crude product is purified by flash chromatography (pentane/Et_2O 1:1 v:v) affording (R)-6-((S,E)-3-((*tert*-Butyldimethylsilyl)oxy)but-1-en-1-yl)-4-methoxy-5,6-dihydro-2H-pyran-2-one **21** (8.23 g, 26.3 mmol, 91%) as a colorless oil, which slowly crystallizes at -20 °C. The compound slowly decomposes at room temperature.

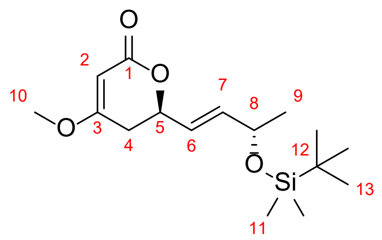

TLC: R_f (pentane/Et_2O 1/1) = 0.16.

$[\alpha]^D_{20}$ = +63.5 (c = 1.04, $CHCl_3$).

^{1}H-NMR (400 MHz, $CDCl_3$, δ in ppm): 5.86 (ddd, J = 15.5 Hz, 4.7 Hz, 0.8 Hz, 1H, H7), 5.73 (ddd, J = 15.4 Hz, 6.1 Hz, 1.3 Hz, 1H, H6), 5.15 (d, J = 1.2 Hz, 1H, H2), 4.91-4.84 (m, 1H, H5), 4.37-4.30 (m, 1H, H8), 3.74 (s, 3H, H10), 2.56 (ddd, J = 17.1 Hz, 10.8 Hz, 1.2 Hz, 1H, $H4_{ax}$), 2.44 (dd, J = 17.1 Hz, 4.4 Hz, 1H, $H4_{eq}$), 1.21 (d, J = 6.4 Hz, 3H, H9), 0.89 (s, 9H, H13), 0.05 (s, 3H, H11), 0.04 (s, 3H, H11').

^{13}C-NMR (100 MHz, $CDCl_3$, δ in ppm): 172.5 (C3), 167.1 (C1), 139.0 (C7), 124.8 (C6), 90.6 (C2), 75.7 (C5), 68.3 (C8), 56.2 (C10), 33.4 (C4), 26.0 (C13), 24.3 (C9), 18.4 (C12), -4.6 (C11), -4.7 (C11').

HRMS: calculated for $C_{16}H_{27}O_4Si$ $[M-H]^-$: 311.16841, measured for $C_{16}H_{27}O_4Si$ $[M-H]^-$: 311.16971.

(R)-6-((S,E)-3-((*tert*-Butyldimethylsilyl)oxy)but-1-en-1-yl)-3-iodo-4-methoxy-5,6-dihydro-2H-pyran-2-one 22

In an oven-dried round bottom flask under an inert atmosphere lactone **21** (1.56 g, 5.0 mmol, 1.0 eq.) is dissolved in dry DMF (50 mL) and successively treated with freshly distilled pyridine (1.60 mL, 20.0 mmol, 4.0 eq.) and DMAP (122 mg, 1.0 mmol, 0.2 eq.). After cooling the reaction mixture to 0 °C, iodine (5.08 g, 20.0 mmol, 4.0 eq.) is added in one portion. The flask is then protected from light with aluminum foil and the red-brown solution is stirred at r.t. for 2 d. The reaction is quenched by addition of saturated aqueous $NaHCO_3$ (20 mL) and saturated aqueous $Na_2S_2O_3$ (20 mL), and the aqueous phase is extracted three times with Et_2O. The combined organic phases are dried over $MgSO_4$, filtered, and the solvent is removed under reduced pressure. Purification of the crude product by column chromatography (pentane/Et_2O 1:1 v:v) affords (R)-6-((S,E)-3-((*tert*-Butyldimethylsilyl)oxy)but-1-en-1-yl)-3-iodo-4-methoxy-5,6-dihydro-2H-pyran-2-one **22** as a yellow wax (1.62 g, 3.71 mmol, 74%).

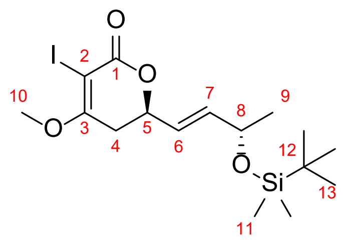

TLC: R_f (pentane/Et_2O 1:1) = 0.23.

$[\alpha]^D_{20}$ = +41.9 (c = 1.48, $CHCl_3$).

$T_{m.p.}$ = 49-51°C.

^{1}H-NMR (400 MHz, $CDCl_3$, δ in ppm): 5.92 (ddd, J = 15.3 Hz, 4.3 Hz, 0.8 Hz, 1H, H7), 5.77 (ddd, J = 15.3 Hz, 6.4 Hz, 1.6 Hz, 1H, H6), 4.90 (dddtr, J = 10.5 Hz, 6.4 Hz, 4.5 Hz, 0.8 Hz, 1H, H5), 4.36 (qddd, J = 6.4 Hz, 4.3 Hz, 1.6 Hz, 0.7 Hz, 1H, H8), 3.93 (s, 3H, H10), 2.77 (dd, J = 17.0 Hz, 4.5 Hz, 1H, $H4_{eq}$), 2.69 (dd, J = 17.0 Hz, 10.5 Hz, 1H, $H4_{ax}$), 1.22 (d, J = 6.4 Hz, 3H, H9), 0.07 (s, 3H, H11), 0.06 (s, 3H, H11').

^{13}C-NMR (100 MHz, $CDCl_3$, δ in ppm): 171.4 (C3), 163.3 (C1), 140.1 (C7), 123.8 (C6), 75.3 (C5), 68.0 (C8), 65.1 (C2), 56.9 (C10), 31.6 (C4), 26.0 (C13), 24.3 (C9), 18.4 (C12), -4.5 (C11), -4.6 (C11').

HRMS: calculated for $C_{16}H_{26}IO_4Si$ $[M-H]^-$: 437.06506, measured for $C_{16}H_{26}IO_4Si$ $[M-H]^-$: 437.06424.

(R)-6-((S,E)-3-((*tert*-Butyldimethylsilyl)oxy)but-1-en-1-yl)-3-(hydroxymethyl)-4-methoxy-5,6-dihydro-2H-pyran-2-one 23

A solution of monomeric formaldehyde was prepared following the procedure by Schlosser *et al.*:[3] paraformaldehyde ($(CH_2O)_n$, 3.0 g, 100 mmol), $(pTsO)_2$ (500 mg), and dry THF (40 mL) are placed in a three neck round bottom flask under inert atmosphere equipped with an distillation apparatus with a tared Schlenk flask as a receiving flask, which is cooled to -78 °C. Under a stream of inert gas, the monomeric formaldehyde:THF azeotrope is distilled, affording a 0.33 M solution of formaldehyde in THF as determined by ^{1}H NMR spectroscopy (13.2 mmol, 3.5 eq.).

In a separate oven-dried Schlenk flask under an inert atmosphere, vinyliodide **22** (1.62 g, 3.72 mmol, 1.0 eq.) is dissolved in dry THF (20 mL) and cooled to -78 °C. A solution of *i*PrMgCl in THF (2.0 M, 2.1 mL, 4.1 mmol, 1.1 eq.) is added dropwise and the yellow solution is stirred for 10 min. The resulting vinyl Grignard reagent is then transferred via cannula at -78 °C into the formaldehyde solution and the mixture is stirred for 2 h. The reaction is quenched by addition of saturated aqueous NH_4Cl, the phases are separated and the aqueous phase is extracted three times with Et_2O. The combined organic phases are dried over $MgSO_4$, filtered and the solvent is removed under reduced pressure. Purification of the crude product by column chromatography (pentane/acetone 2:1 v:v) affords (R)-6-((S,E)-3-((*tert*-Butyldimethylsilyl)oxy)but-1-en-1-yl)-3-(hydroxymethyl)-4-methoxy-5,6-dihydro-2H-pyran-2-one **23** (260 mg, 759 µmol, 20%) as well as the protodeiodination product **21** (787 mg, 2.52 mmol, 68%).

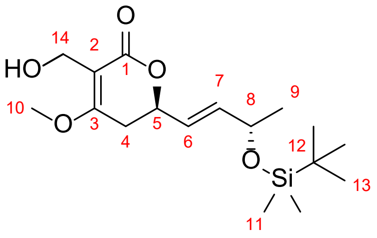

$C_{17}H_{30}O_5Si$
M = 342.51 g•mol^{-1}

TLC: R_f (pentane:acetone 3:1) = 0.18.

$[\mathbf{a}]^{\mathbf{D}}_{\mathbf{20}}$ = +86.6 (c = 0.67, $CHCl_3$).

$\mathbf{T_{m.p.}}$ = 55-56 °C.

^{1}**H-NMR** (400 MHz, $CDCl_3$, δ in ppm): 5.89 (ddd, J = 15.4 Hz, 4.4 Hz, 0.8 Hz, 1H, H7), 5.76 (ddd, J = 15.4 Hz, 6.4 Hz, 1.4 Hz, 1H, H6), 4.89-4.83 (m, 1H, H5), 4.43 (dd, J = 12.2 Hz, 7.3 Hz, 1H, H14), 4.39-4.32 (m, 2H, H14‘+H8), 3.84 (s, 3H, H10), 2.77 (tr, J = 6.8 Hz, 1H, OH), 2.69 (dd, J = 17.3 Hz, 4.6 Hz, 1H, $H4_{eq}$), 2.61 (dd, J = 17.3 Hz, 11.0 Hz, 1H, $H4_{ax}$), 1.21 (d, J = 6.4 Hz, 3H, H9), 0.90 (s, 9H, H13), 0.07 (s, 3H, H11), 0.06 (s, 3H, H11‘).

^{13}**C-NMR** (100 MHz, $CDCl_3$, δ in ppm): 168.4 (C1), 167.4 (C3), 139.7 (C7), 124.4 (C6), 107.6 (C2), 75.0 (C5), 68.1 (C8), 56.1 (C10), 54.9 (C14), 29.8 (C4), 26.0 (C13), 24.3 (C9), 18.4 (C12), -4.6 (C11), -4.7 (C11‘).

HRMS: calculated for $C_{17}H_{29}O_5Si$ $[M-H]^-$: 341.17897, measured for $C_{17}H_{29}O_5Si$ $[M-H]^-$: 341.17920.

(R)-6-((S,E)-3-((*tert*-Butyldimethylsilyl)oxy)but-1-en-1-yl)-4-methoxy-3-(((4-methoxybenzyl)oxy)methyl)-5,6-dihydro-2H-pyran-2-one 24

An oven-dried microwave vessel equipped with a magnetic stir bar is charged with vinyl iodide **22** (438 mg, 1.00 mmol, 1.0 eq.), stannatrane **28** (450 mg, 1.10 mmol, 1.1 eq.), and Ad_2nBuP (143 mg, 0.40 mmol, 0.40 eq.) and is purged three times with inert gas. Dry toluene (4 mL) is added to dissolve the mixture, followed by addition of $[PdCl(C_3H_5)]_2$ (18.0 mg, 50 µmol, 0.05 eq.). The resulting yellow solution is heated in a microwave reactor at 30 W for two periods of 10 min under air cooling (T_{max} = 65 °C) with a 2 min pause in between. After complete

conversion is achieved, the reaction mixture is cooled to 0 °C, upon which 5-Iodo-1-aza-5-stannabicyclo[3.3.3]undecane crystallizes as a colorless solid. The supernatant solution is transferred to a single-neck round-bottom flask, and the solid is washed three times with pentane (5 mL) at 0 °C. The combined organic phases are dried over $MgSO_4$, filtered, and the solvent is removed under reduced pressure. The procedure is repeated to convert a total of 2.5 mmol of vinyl iodide **22**. Purification of the crude product by column chromatography (pentane/acetone 5:1 v:v containing 1 vol% Et_3N) affords the title compound **24** as a yellow oil (915 mg, 2.04 mmol, 82%).

The recovered stannatrane is converted into the corresponding water-soluble hydroxide by treatment with 3 M NaOH and transferred to a separatory funnel. The aqueous phase is washed twice with Et_2O and subsequently acidified to pH 1 with 6 N HCl, resulting in precipitation of chlorostannatrane **26** as a colorless solid. The aqueous phase is extracted twice with DCM, the combined organic phases are dried with $MgSO_4$, filtered and the solvent is removed under reduced pressure to recover approximately 80% of chlorostannatrane **28**.

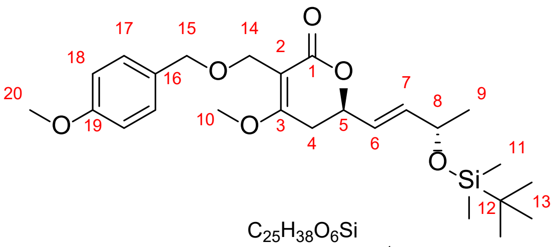

$C_{25}H_{38}O_6Si$
M = 462.66 g•mol^{-1}

TLC: R_f (pentane:acetone 5:1) = 0.24.

TLC: R_f (pentane:Et_2O 1:1) = 0.06.

[**a**]$^{D}_{20}$ = +24.2 (c = 1.20, $CHCl_3$).

^{1}H-NMR (400 MHz, $CDCl_3$, δ in ppm): 7.31-7.27 (m, 2H, H17), 6.88-6.84 (m, 2H, H18), 5.87 (ddd, J = 15.4 Hz, 4.5 Hz, 0.7 Hz, 1H, H7), 5.74 (ddd, J = 15.4 Hz, 6.4 Hz, 1.4 Hz, 1H, H6), 4.82-4.75 (m, 1H, H5), 4.49 (s, 2H, H15), 4.38-4.31 (m, 1H, H8), 4.30 (s, 2H, H14), 3.83 (s, 3H, H10), 3.79 (s, 3H, H20), 2.64 (dd, J = 17.2 Hz, 4.9 Hz, 1H, $H4_{eq}$), 2.58 (dd, J = 17.2 Hz, 10.7 Hz, 1H, $H4_{ax}$), 1.21 (d, J = 6.4 Hz, 3H, H9), 0.90 (s, 9H, H13), 0.06 (s, 3H, H11), 0.05 (s, 3H, H11')

^{13}C-NMR (100 MHz, $CDCl_3$, δ in ppm): 169.5 (C3), 167.3 (C1), 159.2 (C19), 139.3 (C7), 131.1 (C16), 129.5 (C17), 124.7 (C6), 113.7 (C18), 105.6 (C2), 74.4 (C5), 72.4 (C15), 68.1 (C8), 61.0 (C14), 56.1 (C10), 55.4 (C20), 30.1 (C4), 26.0 (C13), 24.3 (C9), 18.4 (C12), -4.5 (C11), -4.6 (C11').

HRMS: calculated for $C_{25}H_{37}O_6Si$ [M-H]$^-$: 461.23649, measured for $C_{25}H_{37}O_6Si$ [M-H]$^-$: 461.24064.

(R)-6-((S,E)-3-Hydroxybut-1-en-1-yl)-4-methoxy-3-(((4-methoxybenzyl)oxy)methyl)-5,6-dihydro-2H-pyran-2-one S4

In a reaction vessel made of plastic equipped with a magnetic stir bar lactone **24** (915 mg, 2.04 mmol, 1.0 eq.) is dissolved in dry THF (20 mL), treated with Pyr•HF (70 wt% HF, 340 µL, 13.8 mmol, 6.7 eq) and stirred overnight. After complete conversion, the reaction is quenched by addition of saturated aqueous $NaHCO_3$ solution. The phases are separated and the aqueous phase is extracted three times with Et_2O and the combined organic phases are dried with $MgSO_4$. After filtration and removal of the solvent under reduced pressure the crude product is purified by flash chromatography (pentane/acetone 10:1 v:v containing 1.5 vol% NEt_3) affording allyl alcohol **S4** (647 mg, 1.86 mmol, 91%) as a colorless oil.

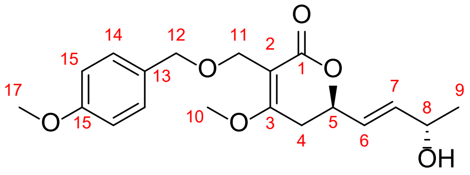

$C_{25}H_{38}O_6Si$
M = 348.40 g•mol^{-1}

TLC: R_f (pentane:acetone 1:1) = 0.39.

$[\mathbf{a}]_{20}^{D}$ = +33.9 (c = 1.18, $CHCl_3$)

^{1}H-NMR (400 MHz, $CDCl_3$, δ in ppm): 7.31-7.27 (m, 2H, H14), 6.88-6.84 (m, 2H, H15), 5.94 (ddd, J = 15.6 Hz, 5.5 Hz, 1.0 Hz, 1H, H7), 5.79 (ddd, J = 15.6 Hz, 6.3 Hz, 1.3 Hz, 1H, H6), 4.83-4.76 (m, 1H, H5), 4.49 (s, 2H, H12), 4.40-4.33 (m, 1H, H8), 4.29 (s, 2H, H11), 3.82 (s, 3H, H10), 3.79 (s, 3H, H17), 2.68 (dd, J = 17.2 Hz, 4.4 Hz, 1H, $H4_{eq}$), 2.59 (dd, J = 17.2 Hz, 11.1 Hz, 1H, $H4_{ax}$), 1.64 (d, J = 4.4 Hz, 1H, OH), 1.29 (d, J = 6.5 Hz, 3H, H9).

^{13}C-NMR (100 MHz, $CDCl_3$, δ in ppm): 169.5 (C3), 167.1 (C1), 159.2 (C16), 138.6 (C7), 131.0 (C13), 129.6 (C14), 125.9 (C6), 113.8 (C15), 105.6 (C2), 74.1 (C5), 72.5 (C12), 67.8 (C8), 61.0 (C11), 56.2 (C10), 55.4 (C17), 30.1 (C4), 23.4 (C9).

HRMS: calculated for $C_{19}H_{23}O_6$ $[M-H]^-$: 347.15001, measured for $C_{19}H_{23}O_6$ $[M-H]^-$: 347.15026.

(S,E)-4-((R)-4-Methoxy-5-(((4-methoxybenzyl)oxy)methyl)-6-oxo-3,6-dihydro-2H-pyran-2-yl)but-3-en-2-yl 2,2,2-trifluoroacetate 25

In an oven-dried round bottom flask under an inert atmosphere, allyl alcohol **S4** (704 mg, 2.02 mmol, 1.0 eq.) is dissolved in dry DCM (20 mL) and cooled to 0 °C. DMAP (24.4 mg, 202 µmol, 0.1 eq.), freshly distilled NEt_3 (560 µL, 4.04 mmol, 2.0 eq.), and TFAA (560 µL, 4.04 mmol, 2.0 eq.) are then successively added dropwise. After stirring for 20 min the reaction is quenched by addition of H_2O (20 mL). The phases are separated, the aqueous phase is extracted three times with DCM (30 mL) and the combined organic phases are dried with $MgSO_4$. After filtration through a 2-3 cm pad of silica and removal of the solvent under reduced pressure the title compound **25** is obtained as a colorless to pale yellow oil (725 mg, 1.63 mmol, 81%). The compound is stable under an inert atmosphere at -20 °C and can be stored for several months.

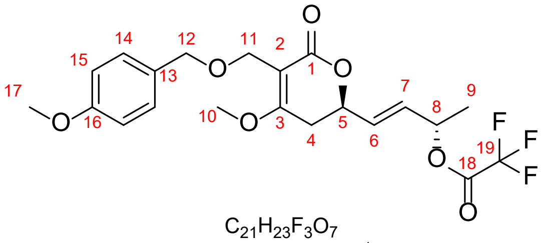

$C_{21}H_{23}F_3O_7$
M = 444.40 g•mol^{-1}

TLC: R_f (pentane:acetone 3:1) = 0.31.

$[\mathbf{a}]_{20}^{D}$ = +0.4 (c = 1.08, $CHCl_3$).

^{1}H-NMR (400 MHz, $CDCl_3$, δ in ppm): 7.28 (m, 2H, H14), 6.86 (m, 2H, H15), 5.98-5.83 (m, 2H, H6+H7), 5.53 (quint, J = 6.2 Hz, 1H, H8), 4.80 (dtr, J = 11.5 Hz, 4.4 Hz, 1H, H5), 4.49 (s, 2H, H12), 4.31 (d, J = 10.9 Hz, 1H, H11), 4.28 (d, J = 10.9 Hz, 1H, H11‘), 3.84 (s, 3H, H10), 3.79 (s, 3H, H17), 2.68 (dd, J = 17.1 Hz, 4.3 Hz, 1H, H4), 2.57 (dd, J = 17.1 Hz, 11.4 Hz, 1H, H4‘), 1.47 (d, J = 6.5 Hz, 3H, H9).

^{13}C-NMR (100 MHz, $CDCl_3$, δ in ppm): 169.2 (C3), 166.7 (C1), 159.2 (C16), 156.8 (q, J = 42 Hz, C18), 131.2 (C7), 130.9 (C13), 130.4 (C6), 129.6 (C14), 114.6 (q, J = 286 Hz, C19), 113.8 (C15), 105.7 (C2), 75.0 (C8), 73.2 (C5), 72.5 (C12), 61.0 (C11), 56.3 (C10), 55.3 (C17), 29.9 (C4), 20.0 (C9).

HRMS: calculated for $C_{21}H_{22}F_3O_7$ $[M-H]^-$: 443.13231, measured for $C_{21}H_{22}F_3O_7$ $[M-H]^-$: 443.13636.

5-(Iodomethyl)-1-aza-5-stannabicyclo[3.3.3]undecane 27[4]

In an oven-dried round-bottom flask under an inert atmosphere, Et_2Zn (1.0 M in hexane, 12.0 mL, 12.0 mmol, 2.5 eq.) is dissolved in dry THF (40 mL) at -60 °C. CH_2I_2 (1.93 mL, 24.0 mmol, 5.0 eq.) is added dropwise and the reaction mixture is stirred for 1 h at -40 °C. Stannatrane chloride **26** (1.41 g, 4.79 mmol, 1.0 eq.) is then added in one portion, the cooling bath is replaced with an ice bath and the mixture is stirred at 0 °C for 3 h. During this time, the initially colorless suspension turns into a pale yellow solution. After additional stirring for 30 min at room temperature, the reaction mixture is transferred into a separatory funnel containing pentane (100 mL) and 3 N HCl (8 mL). The phases are separated and the organic phase is washed twice with distilled H_2O, once with saturated aqueous $NaHCO_3$ and dried with $MgSO_4$. After filtration and removal of the solvent under reduced pressure the title compound **27** (1.87 g, 4.67 mmol, 98%) is obtained as colorless crystals.

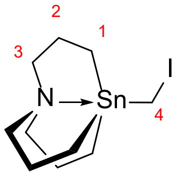

$C_{10}H_{20}INSn$
M = 399.89 g•mol^{-1}

TLC: R_f (pentane:ethyl acetate 10:1) = 0.77.

$T_{m.p.}$ = 39-41 °C.

^{1}H-NMR (400 MHz, $CDCl_3$, δ in ppm): 2.37 (tr, J = 5.6 Hz, 6H, H3), 1.74-1.66 (m, J(^{119}Sn-^{1}H) = 88.5 Hz, 6H, H2), 1.67 (s, 2H, H4), 0.83 (tr, J = 6.8 Hz, J(^{119}Sn-^{1}H) = 54.4 Hz, 6H, H1).

^{13}C-NMR (100 MHz, $CDCl_3$, δ in ppm): 54.8 (C3), 23.4 (C2), 7.3 (C1).

The spectral data are in agreement with literature reports.[4]

5-(((4-Methoxybenzyl)oxy)methyl)-1-aza-5-stannabicyclo[3.3.3]undecane 28

An oven-dried Schlenk flask under an inert atmosphere is charged with KH (1.28 g, 30 wt%, 9.58 mmol, 2.0 eq.), washed twice with dry THF (10 mL) and suspended in dry THF (20 mL). *p*-Methoxybenzyl alcohol (890 µL, 7.19 mmol, 1.5 eq.) is then added dropwise at room temperature, resulting in a highly viscous colorless suspension towards the end of the addition. After stirring for 1 h at room temperature, a solution of stannatrane **27** (1.92 g, 4.79 mmol, 1.0 eq.) in dry THF (10 mL) is added dropwise and the cannula is rinsed with additional dry THF (10 mL). After stirring for 2 h at room temperature, the reaction is quenched by addition of saturated aqueous NH_4Cl and the phases are separated. The aqueous phase is extracted three times with Et_2O and the combined organic phases are dried with $MgSO_4$. After filtration and removal of the solvent under reduced pressure the crude product is purified by flash chromatography (pentane/ethyl acetate 10:1 v:v) to afford PMBM stannatrane **28** (1.47 g, 3.59 mmol, 75%).as colorless crystals.

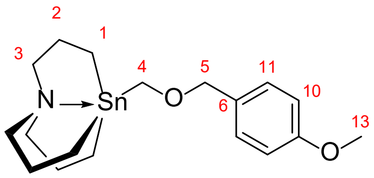

$C_{18}H_{29}NO_2Sn$
M = 410.15 g•mol^{-1}

TLC: R_f (pentane:ethyl acetate 10:1) = 0.29.

$T_{m.p.}$ = 20-22 °C.

^{1}H-NMR (400 MHz, $CDCl_3$, δ in ppm): 7.25-7.21 (m, 2H, H7), 6.88-6.84 (m, 2H, H8), 4.31 (s, 2H, H5), 3.80 (s, 3H, H10), 3.34 (s, 2H, H4), 2.38 (tr, J = 5.8 Hz, 6H, H1), 1.70-1.61 (m, J(^{119}Sn-^{1}H) = 82.3 Hz, 6H, H2), 0.73 (tr, J = 6.8 Hz, J(^{119}Sn-^{1}H) = 49.8 Hz, 6H, H3).

^{13}C-NMR (100 MHz, $CDCl_3$, δ in ppm): 158.9 (C9), 131.8 (C6), 129.1 (C7), 113.7 (C8), 76.8 (C5), 68.1 (C4), 55.4 (C10), 54.9 (J^{119}Sn-^{13}C = 27.9 Hz, C1), 23.3 (J^{119}Sn-^{13}C = 25.4 Hz, C2), 6.4 (J^{119}Sn-^{13}C = 392.0 Hz, C3).

HRMS: measured for $C_{18}H_{30}NO_2Sn$ $[M+H]^+$: 412.12931, calculated for $C_{18}H_{30}NO_2Sn$ $[M+H]^+$: 412.12948.

3-((2S,3R)-3-ethyl-3-(trimethylsilyl)oxiran-2-yl)propan-1-ol 30

In a 1L three necked round bottom flask with two identical dropping funnels the alkene **29** (85 w% prepared according to literature procedure, 2.26g, 10.3 mmol, 1.0 eq.) is dissolved in MeCN (175 mL) and cooled to 0°C. To this solution the L-Shi catalyst **31** (prepared from L-sorbose according to Shi *et al.*,[5] 2.20 g, 8.52 mmol, 0.7 eq.), *n*Bu_4NHSO_4 (496 mg, 1.46 mmol, 0.12 eq) and a solution of Na_2EDTA (10^{-4} M, 125 mL) are added in succession and the solution is stirred vigorously. Then a freshly prepared solution of K_2CO_3 (11.27 g, 81.5 mmol, 6.7 eq.) in H_2O (85 mL) and oxone (11.97 g, 19.5 mmol, 1.6 eq.) in Na_2EDTA (10^{-4} M, 85 mL) are filled into the respective dropping funnels. The solutions are simultaneously added dropwise to the reaction mixture (caution! a too one-sided addition of either solution reduces the yield dramatically) over the course of 1 h. After complete addition the mixture is stirred for another hour before being quenched with 100 mL pentane. The triphasic mixture is transferred into a separatory funnel and the lower aqueous phase is separated. The aqueous phase is extracted 3x with 100 mL pentane and once with 100 mL ethyl acetate. The combined organic phases are dried with $MgSO_4$, filtrated and concentrated under reduced pressure. The crude product is purified by flash chromatography (pentane:Et_2O 5:1 → 2:1 →1:1 v:v) affording 3-((2S,3R)-3-ethyl-3-(trimethylsilyl)oxiran-2-yl)propan-1-ol **30** (1.56 g, 7.1 mmol, 75%).

Derivatization to the PNB ester and HPLC analysis (Chiralcel OD-H, 1 mL/min, 25 °C, *n*Hex:*i*PrOH 95:5, 254 nm, t_{R1} = 7.03 min, t_{R2} = 8.25 min) indicated 92 % ee.

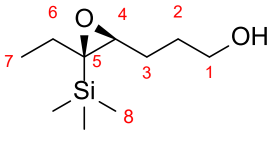

$C_{10}H_{22}O_2Si$
M = 202.37 g•mol^{-1}

The enantiomer is synthesized with identical yield and ee using D-Shi catalyst instead.

TLC: R_f (pentane/Et_2O 1/1) = 0.16

^{1}H-NMR (400 MHz, $CDCl_3$, δ in ppm): 3.77-3.66 (m, 2H, H1), 2.73 (dd, J = 8.5 Hz, 3.7 Hz, 1H, H4), 1.94 (dq, J = 13.3 Hz, 7.3 Hz, 1H, H3), 1.87-1.72 (m, 4H, H3'+H2+OH), 1.53-1.43 (m, 1H, H6), 1.16-1.06 (m, 1H, H6'), 0.91 (tr, J = Hz, 3H, H7), 0.15 (s, 9H, H8).

^{13}C-NMR (100 MHz, $CDCl_3$, δ in ppm): 63.3 (C1), 62.9 (C4), 59.2 (C5), 30.4 (C6+C3), 27.7 (C2), 10.2 (C7), -1.0 (C8).

The spectral data are in agreement with literature reports.[2]

3-((2S,3R)-3-ethyl-3-(trimethylsilyl)oxiran-2-yl)propanal 32

In a 50 mL round bottom flask epoxy alcohol **30** (1.20 g, 5.95 mmol, 1.0 eq.) is dissolved in 30 mL of dry DCM and cooled to 0 °C. Pyridine (575 µL, 7.14 mmol, 1.2 eq.) and DMP (3.03 g, 7.14 mmol, 1.2 eq.) are sequentially added and the yellow solution is stirred for 2 h at room temperature. The reaction is quenched by addition of both a saturated $Na_2S_2O_3$ and a $NaHCO_3$ solution and the biphasic mixture is transferred into a separatory funnel. The phases are separated and the aqueous phase is extracted 3x with 10 mL DCM. The combined organic phases are dried with $MgSO_4$. After filtration and removal of the solvent under reduced pressure the crude product is purified by flash chromatography (pentane:Et_2O 5:1 v:v) affording 3-((2S,3R)-3-ethyl-3-(trimethylsilyl)oxiran-2-yl)propanal **32** (1.01 g, 5.00 mmol, 83%) as a colorless liquid with fruity odor.

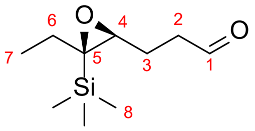

$C_{10}H_{20}O_2Si$
M = 200.35 g•mol^{-1}

TLC: R_f (pentane/Et_2O 5/1) = 0.15

^{1}H-NMR (400 MHz, $CDCl_3$, δ in ppm):9.83 (tr, J = 1.3 Hz, 1H, H1), 2.72 (dd, J = 8.5 Hz, 4.3 Hz, 1H, H4), 2.69-2.57 (m, 2H, H2), 2.04 (dddd, J = 14.6 Hz, 8.1 Hz, 6.8 Hz, 4.3 Hz, 1H, H3), 1.91 (dq, J = 13.6 Hz, 7.3 Hz, 1H, H3‘), 1.69 (dddd, J = 14.3 Hz, 8.5 Hz, 7.8 Hz, 6.6 Hz, 1H, H6), 1.15-1.05 (m, 1H, H6‘), 0.90 (tr, J = 7.5 Hz, 3H, H7), 0.14 (s, 9H, H8).

^{13}C-NMR (100 MHz, $CDCl_3$, δ in ppm): 201.4 (C1), 62.3 (C4), 59.2 (C5), 41.6 (C2), 30.3 (C6), 23.7 (C3), 10.2 (C7), -1.1 (C8).

(2S,3S,6R)-2-ethyl-2-(trimethylsilyl)-6-((trimethylsilyl)ethynyl)tetrahydro-2H-pyran-3-ol 33

A 100 mL round bottom flask with a small reflux condenser and a three-way stopcock is charged with $Zn(OTf)_2$ (2.00 g, 5.5 mmol, 1.1 eq.) under N_2. The $Zn(OTf)_2$ is dried with a heat gun under vacuum for 10 min and the flask is purged 3 x with N_2. After cooling to room temperature (+)-NME (1.08 g, 6.0 mmol, 1.2 eq.) is added and the mixture is dissolved with dry toluene (25 mL). Then freshly distilled NEt_3 (830 µL, 6.0 mmol, 1.2 eq.) is added in one portion and the resulting biphasic colorless mixture is stirred vigorously for 2 h. Afterwards tms acetylene (3.6 mL, 25.0 mmol, 5.0 eq.) is added at once and the mixture is stirred for 15 min at room temperature before being heated to 60 °C. The aldehyde **32** (1.01 g, 5.00 mmol, 1.0 eq.) dissolved in dry toluene (25 mL) is then added via a syringe pump over the course of 10 h (shorter addition times result in major aldol side reaction!). After the addition is complete the mixture is stirred for an additional 4 h before being quenched with saturated NH_4Cl solution. The biphasic mixture is separated and the aqueous phase is extracted three times with Et_2O (50 mL). The combined organic phases are dried with $MgSO_4$, filtrated and concentrated under reduced pressure yielding the open chained product (2.39 g, 160 %) as a yellow oil with ~10:1 dr (separatable but not done at this point).

In a dried round bottom flask under N_2 the crude product is dissolved in dry DCM (50 mL) and cooled to 0 °C with stirring. Then $BF_3•OEt_2$ (634 µL 1.0 eq, 5.00 mmol) is added in one portion. After stirring for 10 min the mixture is immediately quenched with saturated $NaHCO_3$ solution and the biphasic mixture is separated. The aqueous phase is extracted three times with DCM (20 mL) and the combined organic phases are dried with $MgSO_4$. After filtration and removal of the solvent under reduced pressure the crude product is purified via flash chromatography (pentane:Et_2O 10:1 v:v) affording pure (2S,3S,6R)-2-ethyl-2-(trimethylsilyl)-6-((trimethylsilyl)ethynyl)tetrahydro-2H-pyran-3-ol **33** (952 mg, 3.18 mmol, 64%).

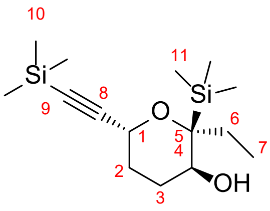

$C_{15}H_{30}O_2Si_2$
M = 298.57 g•mol^{-1}

TLC: R_f (pentane/Et_2O 10/1) = 0.18 (main product), 0.25 (epimer)

^{1}H-NMR (400 MHz, $CDCl_3$, δ in ppm): 4.24 (dd, J = 10.4 Hz, 2.3 Hz, 1H, H1), 3.70 (dtr, J = 10.2 Hz, 4.4 Hz, 1H, H4), 1.99-1.82 (m, 1H, H3+H2+H6), 1.82-1.60 (m, 3H, H2'+H3'+H6'), 1.48 (brd, J = 4.3 Hz, 1H, OH), 0.97 (tr, J = 7.4 Hz, H7), 0.20 (s, 9H, H11), 0.16 (s, 9H, H10).

^{13}C-NMR (100 MHz, $CDCl_3$, δ in ppm): 105.2 (C8), 88.5 (C9), 78.0 (C5), 71.8 (C4), 64.5 (C1), 32.0 (C2), 29.7 (C3), 29.6 (C6), 7.6 (C7), 1.0 (C11), 0.1 (C10).

HRMS: calculated for $C_{15}H_{31}O_2Si_2$ $[M+H]^+$: 299.18571, measured for $C_{15}H_{31}O_2Si_2$ $[M+H]^+$:299.18444.

(2R,3S,6R)-2-Ethyl-6-ethynyltetrahydro-2H-pyran-3-ol 34

In a round bottom flask the alcohol **32** (1.05 g, 3.52 mmol, 1.0 eq.) is dissolved in THF (35 mL) at room temperature. AcOH (200 µL, 3.52 mmol, 1.0 eq) and TBAF (1.0 M in THF, 3.5 mL 3.52 mmol, 1.0 eq.) are then added sequentially and the mixture is stirred for 10 min before being quenched with saturated $NaHCO_3$ solution. The phases are separated and the aqueous phase is extracted twice with Et_2O. The organic phases are combined and dried with $MgSO_4$. After filtration and evaporation of the solvent the crude mono deprotected alcohol is transferred into a round bottom flask with a small reflux condenser and cycled with inert gas before being dissolved in dry THF (35 mL). TBAF (1.0 M in THF, 14.0 mL, 14 mmol, 4.0 eq.) is then added resulting in a slightly red solution which is stirred at 45 °C for 18 h. The wine-red mixture is quenched with saturated NH_4Cl solution and the phases are separated. The aqueous phase is extracted three times with Et_2O (20 mL). The organic phases are combined and dried with $MgSO_4$. After filtration and removal of the solvent under reduced pressure the crude product is purified via flash chromatography (pentane:acetone 10:1 →5:1 v:v) affording (2R,3S,6R)-2-Ethyl-6-ethynyltetrahydro-2H-pyran-3-ol **34** (474 mg, 3.07 mmol, 87%) as colorless crystals.

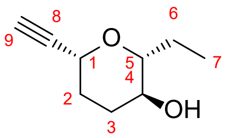

$C_9H_{14}O_2$
M = 154.21 g•mol^{-1}

TLC: R_f (pentane/acetone 5/1) = 0.18

$T_{m.p.}$ = 50-52 °C

$[\alpha]^D_{20}$ = +106.4° (c = 1.10, $CHCl_3$)

^{1}H-NMR (400 MHz, $CDCl_3$, δ in ppm): 4.08 (dtr, J = 11.4 Hz, 2.3 Hz, 1H, H1), 3.41-3.31 (m, 1H, H4), 3.00 (trd, J = 8.7 Hz, 2.6 Hz, 1H, H5), 2.45 (d, J = 2.1 Hz, 1H, H9), 2.14-2.07 (m, 1H, H3'), 1.97 (ddtr, J = 13.8 Hz, 4.9 Hz, 2.7 Hz, 1H, H2'), 1.89 (dqd, J = 14.6 Hz, 7.6 Hz, 2.8 Hz, 1H, H6'), 1.87-1.76 (m, 1H, H2), 1.56-1.43 (m, 1H, H6), 1.50-1.41 (m, 1H, H3), 1.41 (brd, J = 5.2 Hz, 1H, OH), 1.01 (tr, J = 7.5 Hz, 3H, H7).

^{13}C-NMR (100 MHz, $CDCl_3$, δ in ppm): 83.9 (C5), 82.8 (C8), 72.7 (C9), 69.5 (C4), 67.5 (C1), 32.9 (C3), 32.4 (C2), 24.9 (C6), 9.9 (C7).

HRMS: calculated for $C_9H_{15}O_2$ $[M+H]^+$: 155.10666, measured for $C_9H_{15}O_2$ $[M+H]^+$: 155.10679.

(2R,3S,6R)-2-Ethyl-6-((trimethylsilyl)ethynyl)tetrahydro-2H-pyran-3-ol 35

In an oven-dried round bottom flask under inert atmosphere pyran **34** (972 mg, 6.30 mmol, 1.0 eq.) is dissolved in dry THF (30 mL) and cooled to -78 °C. *n*BuLi (5.8 mL, 2.5 M in hexane, 14.5 mmol, 2.3 eq.) is added dropwise, resulting in a colorless, highly viscous suspension toward the end of the addition. After stirring for 20 min, TMSCl (2.0 mL, 15.8 mmol, 2.5 eq.) is added dropwise, during which the suspension dissolves. The cooling bath is removed and the colorless reaction mixture is allowed to warm to room temperature. After complete double silylation (approximately 20 min), 2 N HCl (17 mL) is added in one portion under stirring. After an additional 30 min, the reaction is quenched by careful addition of saturated $NaHCO_3$ solution and the phases are separated. The aqueous phase is extracted three times with Et_2O (20 mL) and the combined organic phases are dried with

$MgSO_4$. After filtration and removal of the solvent under reduced pressure the crude product is purified via flash chromatography (pentane:acetone 10:1 v:v) affording (2R,3S,6R)-2-Ethyl-6-((trimethylsilyl)ethynyl)tetrahydro-2H-pyran-3-ol **35** (1.40 g, 6.18 mmol, 98%) as a colorless oil.

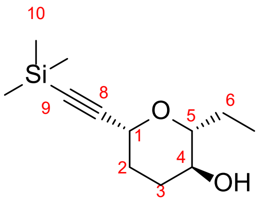

$C_{12}H_{20}O_2Si$
M = 226.39 g•mol^{-1}

TLC: R_f (pentane/acetone 10/1) = 0.18 (major product), 0.77 (double tms protected intermediate)

$[\alpha]^{D}_{20}$ = +87.6 (c = 1.05, $CHCl_3$).

^{1}H-NMR (400 MHz, $CDCl_3$, δ in ppm): 4.08 (dd, J = 11.4 Hz, 2.4 Hz, 1H, H1), 3.40-3.31 (m, 1H, H4), 2.98 (trd, J = 8.7 Hz, 2.8 Hz, 1H, H5), 2.12-2.05 (m, 1H, H3), 1.96 (ddtr, J = 13.7 Hz, 4.4 Hz, 2.7 Hz, 1H, H2), 1.91-1.83 (m, 1H, H6), 1.79 (trdd, J = 13.6 Hz, 11.5 Hz, 3.9 Hz, 1H, H2‘), 1.56-1.38 (m, 3H, OH+H3‘+H6‘), 1.01 (tr, J = 7.5 Hz, 3H, H7), 0.16 (s, 9H, H10).

^{13}C-NMR (100 MHz, $CDCl_3$, δ in ppm): 104.3 (C8), 89.2 (C9), 83.9 (C5), 69.6 (C4), 68.2 (C1), 33.0 (C3), 32.6 (C2), 25.0 (C6), 10.0 (C7), 0.0 (C10).

HRMS: calculated for $C_{12}H_{21}O_2Si$ $[M-H]^-$: 225.131630, measured for $C_{12}H_{21}O_2Si$ $[M-H]^-$: 225.131988.

(2R,6R)-2-Ethyl-6-((trimethylsilyl)ethynyl)dihydro-2H-pyran-3(4H)-one S5

In an oven-dried round bottom flask under inert atmosphere alcohol **35** (909 mg, 3.81 mmol, 1.0 eq.) is dissolved in dry DCM (20 mL) and cooled to 0 °C. Afterwards dry pyridine (330 µL, 4.00 mmol, 1.05 eq.) is added, followed by addition of Dess-Martin periodinane (1.70 g, 4.00 mmol, 1.05 eq.) in one portion. After 5 min, the ice bath is removed and the reaction mixture is stirred at room temperature for 2 h. Upon complete conversion, the reaction is quenched by successive addition of saturated $NaHCO_3$ solution and saturated $Na_2S_2O_3$ solution and the phases are separated. The aqueous phase is extracted three times with Et_2O and the combined organic phases are dried with $MgSO_4$. After filtration and removal of the solvent under reduced pressure the crude product is purified via flash chromatography (pentane:acetone 10:1 v:v) affording (2R,6R)-2-Ethyl-6-((trimethylsilyl)ethynyl)dihydro-2H-pyran-3(4H)-on **S5** (791 mg, 3.53 mmol, 93%) as a slightly yellow oil.

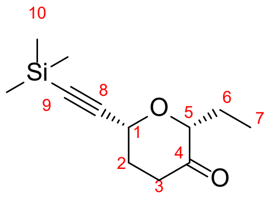

$C_{12}H_{20}O_2Si$
M = 224.38 g•mol^{-1}

TLC: R_f (pentane/acetone 10/1) = 0.50.

$[\alpha]^{D}_{20}$ = +127.2 (c = 1.05, $CHCl_3$).

^{1}H-NMR (400 MHz, $CDCl_3$, δ in ppm): 4.51 (dd, J = 8.4 Hz, 5.4 Hz, 1H, H1), 3.78 (dd, J = 7.3 Hz, 4.7 Hz, 1H, H5), 2.61 (ddd, J = 16.1 Hz, 5.5 Hz, 5.0 Hz, 1H, H3), 2.50-2.39 (m, 1H, H3‘), 2.33-2.25 (m, 2H, H2), 1.87 (dqd, J = 14.6 Hz, 7.5 Hz, 4.7 Hz, 1H, H6), 1.70 (dquint, J = 14.6 Hz, 7.3 Hz, 1H, H6‘), 0.98 (tr, J = 7.5 Hz, 3H, H7), 0.18 (s, 9H, H10).

^{13}C-NMR (100 MHz, $CDCl_3$, δ in ppm): 207.9 (C4), 103.2 (C8), 90.5 (C9), 84.6 (C5), 66.6 (C1), 37.2 (C3), 32.5 (C2), 23.4 (C6), 10.0 (C7), -0.1 (C10).

HRMS: calculated for $C_{12}H_{21}O_2Si$ $[M+H]^+$: 225.13053, measured for $C_{12}H_{21}O_2Si$ $[M+H]^+$: 225.13152.

(2R,6R)-2-Ethyl-6-((trimethylsilyl)ethynyl)-5,6-dihydro-2H-pyran-3-yl trifluoromethanesulfonate 36

In an oven-dried Schlenk flask under inert atmosphere ketone **S5** (790 mg, 3.53 mmol, 1.0 eq.) is dissolved in dry THF (35 mL) and cooled to -78 °C. KHMDS (5.3 mL, 1.0 M in THF, 5.30 mmol, 1.5 eq.) is added dropwise along the flask wall and the reaction mixture is stirred for 1 h. Meanwhile, $PhNTf_2$ (1.51 g, 4.24 mmol, 1.2 eq.) is dissolved in dry THF (10 mL) in an oven-dried pear flask under inert atmosphere and added dropwise to the reaction mixture over 10 min, during which the solution gradually turns deep black. After complete addition, the reaction mixture is allowed to slowly warm to room temperature overnight. Upon complete conversion, the reaction is quenched by addition of saturated aqueous NH_4Cl and the phases are separated. The aqueous phase is extracted three times with Et_2O and the combined organic phases are dried with $MgSO_4$. After filtration through a pad of Celite and removal of the solvent under reduced pressure the crude product is purified via flash chromatography (pentane/Et_2O 100:1 v:v) affording (2R,6R)-2-Ethyl-6-((trimethylsilyl)ethynyl)-5,6-dihydro-2H-pyran-3-yl trifluoromethanesulfonate **36** (854 mg 2.40 mmol, 68%) as a yellow oil.

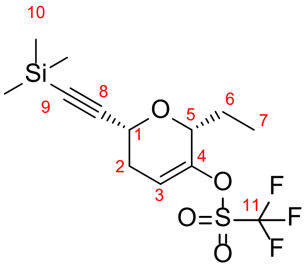

$C_{13}H_{19}F_3O_4SSi$
M = 356.43 g•mol^{-1}

TLC: R_f (pentane/Et_2O 100/1) = 0.11.

$[\alpha]^{D}_{20}$ = +50.1 (c = 1.08, $CHCl_3$).

^{1}H-NMR (500 MHz, $CDCl_3$, δ in ppm): 5.88 (dtr, J = 6.3 Hz, 2.0 Hz, 1H, H3), 4.36 (ddd, J = 10.6 Hz, 3.3 Hz, 0.6 Hz, 1H, H1), 4.31-4.27 (m, 1H, H5), 2.62 (dddd, J = 17.3 Hz, 10.6 Hz, 3.9 Hz, 2.2 Hz, 1H, H2), 2.43 (ddtr, J = 17.4 Hz, 6.3 Hz, 3.2 Hz, 1H, H2'), 1.87 (dqd, J = 14.6 Hz, 7.4 Hz, 3.4 Hz, 1H, H6), 1.67 (dqd, J = 14.5 Hz, 7.3 Hz, 6.7 Hz, 1H, H6'), 0.99 (tr, J = 7.4 Hz, 3H, H7), 0.18 (s, 9H, H10).

^{13}C-NMR (125 MHz, $CDCl_3$, δ in ppm): 147.9 (C4), 116.5 (C3), 102.4 (C8), 91.0 (C9), 75.3 (C5), 64.4 (C1), 32.0 (C2), 24.4 (C6), 8.5 (C7), -0.1 (C10), C11 (not detected).

HRMS: calculated for $C_{13}H_{20}F_3O_4SSi$ $[M+H]^+$: 357.07981, measured for $C_{13}H_{20}F_3O_4SSi$ $[M+H]^+$: 357.08282.

(((2R,6R)-6-Ethyl-5-methyl-3,6-dihydro-2H-pyran-2-yl)ethynyl)trimethylsilane 37

In an oven-dried Schlenk flask under inert atmosphere vinyl triflate **36** (877 mg, 2.46 mmol, 1.0 eq.) is dissolved in dry THF (7 mL) and dry NMP (19 mL) at room temperature. $Fe(acac)_3$ (956 mg, 2.70 mmol, 1.1 eq.) is added and the resulting red suspension is cooled to -35 °C. MeMgBr (8.2 mL, 3.0 M in Et2O, 24.6 mmol, 10.0 eq.) is then added dropwise along the flask wall over 15 min, during which vigorous gas evolution occurs and after initial formation of a yellow precipitate, a viscous black suspension forms. After stirring for 2 h, the reaction is quenched by addition of saturated NH_4Cl solution and the phases are separated. The aqueous phase is extracted three times with Et_2O and the combined organic phases are dried with $MgSO_4$. After filtration through a pad of Celite and removal of the solvent under reduced pressure the crude product is purified via flash chromatography (pentane/Et_2O 150:1 v:v) affording (((2R,6R)-6-ethyl-5-methyl-3,6-dihydro-2H-pyran-2-yl)ethynyl)trimethylsilane **37** (484 mg, 2.18 mmol, 89%) as a colorless oil.

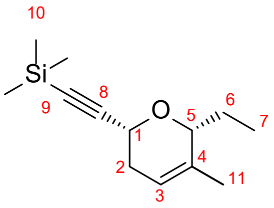

$C_{13}H_{22}OSi$
M = 222.40 g•mol^{-1}

TLC: R_f (pentane/Et_2O 100/1) = 0.31.

$[\alpha]^{D}_{20}$ = +128.1 (c = 1.28, $CHCl_3$).

^{1}H-NMR (400 MHz, $CDCl_3$, δ in ppm): 5.53-5.49 (m, 1H, H3), 4.24 (dd, J = 10.8 Hz, 3.3 Hz, 1H, H1), 4.10-4.04 (m, 1H, H5), 2.40-2.30 (m, 1H, H2), 2.16-2.07 (m, 1H, H2‘), 1.79 (dqd, J = 14.7 Hz, 7.4 Hz, 3.5 Hz, 1H, H6), 1.57 (dd, J = 2.5 Hz, 1.2 Hz, 3H, H11), 1.55 (dqd, J = 14.7 Hz, 7.2 Hz, 6.3 Hz, 1H, H6‘), 0.91 (tr, J = 7.3 Hz, 3H, H7), 0.17 (s, 9H, H10).

^{13}C-NMR (100 MHz, $CDCl_3$, δ in ppm): 135.4 (C4), 120.3 (C3), 105.1 (C8), 89.1 (C9), 78.7 (C5), 64.6 (C1), 32.5 (C2), 25.5 (C6), 19.1 (C11), 8.5 (C7), 0.06 (C10).

HRMS: calculated for $C_{13}H_{21}OSi$ [M-H]$^-$: 221.13672, measured for $C_{13}H_{21}OSi$ [M-H]$^-$: 221.12952.

(2R,6R)-6-Ethyl-2-ethynyl-5-methyl-3,6-dihydro-2H-pyran S6

In an round bottom flask alkene **37** (370 mg, 1.66 mmol, 1.0 eq.) is dissolved in Et_2O (5 mL) and successively treated with AcOH (100 µL, 1.75 mmol, 1.05 eq.) and TBAF•3H_2O (552 mg, 1.75 mmol, 1.05 eq.). After stirring for 30 min, the reaction is quenched by addition of saturated $NaHCO_3$ solution and the phases are separated. The aqueous phase is extracted three times with Et_2O and the combined organic phases are dried with $MgSO_4$. After filtration, the solvent is carefully removed under reduced pressure (>300 mbar). Purification of the crude product by flash chromatography (pentane/Et_2O 100:1 v:v) affords (2R,6R)-6-ethyl-2-ethynyl-5-methyl-3,6-dihydro-2H-pyran **S6** (226 mg, 1.50 mmol, 91%) as a colorless, highly volatile oil.

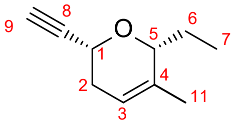

$C_{13}H_{22}OSi$
M = 150.22 g•mol^{-1}

TLC: R_f (pentane/Et_2O 100/1) = 0.33.

^{1}H-NMR (400 MHz, $CDCl_3$, δ in ppm): 5.53 (dquint, J = 6.1 Hz, 1.5 Hz, 1H, H3), 4.25 (dddd, J = 10.8 Hz, 3.2 Hz, 2.2 Hz, 0.5 Hz, 1H, H1), 4.13-4.07 (m, 1H, H5), 2.47 (d, J = 2.2 Hz, 1H, H9), 2.43-2.33 (m, 1H, H2), 2.17-2.09 (m, 1H, H2‘), 1.79 (dqd, J = 14.6 Hz, 7.4 Hz, 3.6 Hz, 1H, H6), 1.58 (dd, J = 2.5 Hz, 1.2 Hz, 3H, H10), 1.55 (dqd, J = 14.5 Hz, 7.2 Hz, 6.1 Hz, 1H, H6‘), 0.90 (tr, J = 7.3 Hz, 3H, H7).

^{13}C-NMR (100 MHz, $CDCl_3$, δ in ppm): 135.4 (C4), 120.1 (C3), 83.5 (C8), 78.7 (C5), 72.8 (C9), 63.9 (C1), 32.3 (C2), 25.5 (C6), 19.1 (C10), 8.3 (C7).

The spectral data are in agreement with literature reports.[6]

Tributyl((E)-2-((2R,6R)-6-ethyl-5-methyl-3,6-dihydro-2H-pyran-2-yl)prop-1-en-1-yl)stannane 38

In an oven-dried 10 mL Schlenk flask CuCN (155 mg, 1.73 mmol, 1.2 eq.) is dried under vacuum using a heat gun and the flask is purged three times with inert gas. The pale beige powder cooled to room temperature, suspended in dry THF (20 mL), and cooled to -78 °C. *n*BuLi (1.4 mL, 2.5 M in hexane, 3.46 mmol, 2.4 eq.) is added dropwise and the cooling bath is removed for 2 min. The suspension is allowed to warm slowly under stirring until a colorless to pale yellow solution forms. The solution is then recooled to -90 °C and *n*Bu_3SnH (930 µL, 3.46 mmol, 2.4 eq.) is added dropwise, during which an intense yellow coloration accompanied by gas

evolution is observed. After stirring for 10 min, a solution of pyran **S6** (217 mg, 1.44 mmol, 1.0 eq.) in dry THF (8 mL) is added slowly along the flask wall and the syringe is rinsed with additional dry THF (2 mL). After 15 min, MeI (1.8 mL, 28.8 mmol, 20 eq.) and dry DMPU (850 µL) are sequentially added dropwise. The cooling bath is removed and the wine red reaction mixture is allowed to warm to room temperature. After stirring for 1 h, the reaction mixture becomes colorless. The reaction is then quenched by addition of 3 mL of a 9:1 mixture of saturated aqueous NH_4Cl and NH_4OH. The phases are separated and the aqueous phase is extracted three times with Et_2O (10 mL). The combined organic phases are dried with $MgSO_4$, filtered, and the solvent is removed under reduced pressure. Purification of the crude product by flash chromatography (puriFlash XS520Plus chromatography system using a Büchi FlashPure Select C18 30 µm cartridge, MeCN → MeCN/DCM 60:40 v:v) affords tributyl((E)-2-((2R,6R)-6-ethyl-5-methyl-3,6-dihydro-2H-pyran-2-yl)prop-1-en-1-yl)stannane **38** (618 mg, 1.38 mmol, 94%) as a colorless oil.

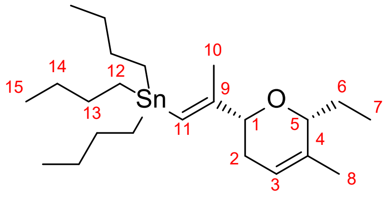

$C_{23}H_{44}OSn$
M = 455.31 g•mol^{-1}

TLC: R_f (MeCN:DCM 60:40, C18-TLC plate) = 0.43.

$[\alpha]^{D}_{20}$ = +45.9 (c = 1.19, $CHCl_3$).

^{1}H-NMR (400 MHz, $CDCl_3$, δ in ppm): 5.84 (s, 1H, H10), 5.59-5.53 (m, 1H, H3), 4.13-4.09 (m, 1H, H5), 3.92 (dd, J = 10.0 Hz, 3.3 Hz, 1H, H1), 2.13-1.94 (m, 2H, H2), 1.85-1.73 (m, 1H, H6), 1.79 (s, 3H, H11), 1.61-1.58 (m, 3H, H8), 1.57-1.40 (m, 7H, H6'+H13), 1.35-1.24 (m, 6H, H14), 0.94-0.85 (m, 15H, H7+H12+H15).

^{13}C-NMR (100 MHz, $CDCl_3$, δ in ppm): 154.7 (C9), 135.5 (C4), 121.7 (C10), 121.0 (C3), 78.7 (C1), 78.3 (C5), 31.0 (C2), 29.43 (1C, C13), 29.36 (2C, C13), 27.57 (1C, C14), 27.48 (2C, C14), 25.9 (C6), 21.0 (C11), 19.1 (C8), 13.9 (C15), 10.2 (C12), 8.9 (C7).

HRMS: calculated for $C_{23}H_{43}OSn$ $[M-H]^-$: 455.23414, measured for $C_{23}H_{43}OSn$ $[M-H]^-$: 455.22110.

(2R,6R)-2-ethyl-6-ethynyldihydro-2H-pyran-3(4H)-one 39

In a round bottom flask alcohol **34** (93 mg, 600 µmol, 1.0 eq.) is dissolved in dry DCM (10 mL) and cooled to 0 °C. To this are sequentially added at once pyridine (52 µL, 630 µmol, 1.05 eq.) and DMP (267 mg, 630 µmol, 1.05 eq.) with stirring. The ice bath is removed and the reaction is stirred for an additional 2 h at room temperature. The reaction is then quenched by sequential addition of a saturated $NaHCO_3$ and a saturated $Na_2S_2O_3$ solution. The phases are separated and the aqueous phase is extracted three times with DCM (10 mL). The combined organic phases are dried with $MgSO_4$. After filtration through a 2 cm pad of silical gel 60 and removal of the solvent under reduced pressure the crude product is purified by flash chromatography (pentane:acetone 5:1 v:v) affording (2R,6R)-2-ethyl-6-ethynyldihydro-2H-pyran-3(4H)-one **39** (87.5 mg, 575 µmol, 96%) as a colorless oil, which crystallizes in the refrigerator.

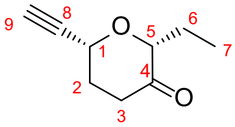

$C_9H_{12}O_2$
M = 152.19 g•mol^{-1}

TLC: R_f (pentane/acetone 5/1) = 0.36

$[\alpha]^{D}_{20}$ = +149.2 (c = 1.20, $CHCl_3$)

^{1}H-NMR (400 MHz, $CDCl_3$, δ in ppm): 4.52 (ddd, J = 8.0 Hz, 5.8 Hz, 2.1 Hz, 1H, H1), 3.80 (dd, J = 7.3 Hz, 4.5 Hz, 1H, H5), 2.62 (dtr, J = 16.2 Hz, 5.3 Hz, 1H, H3), 2.54 (d, J = 2.1 Hz, 1H, H9), 2.51-2.42 (m, 1H, H3‘), 2.35-2.27 (m, 2H, H2), 1.89 (dqd, J = 14.9 Hz, 7.5 Hz, 4.5 Hz, 1H, H6), 1.69 (dquint, J = 14.8 Hz, 7.4 Hz, 1H, H6‘), 0.98 (tr, J = 7.5 Hz, 3H, H7).

^{13}C-NMR (100 MHz, $CDCl_3$, δ in ppm): 207.5 (C4), 84.5 (C5), 81.9 (C8), 73.8 (C9), 66.0 (C1), 37.1 (C3), 32.3 (C2), 23.3 (C6), 9.9 (C7).

HRMS: calculated for $C_9H_{13}O_2$ $[M+H]^+$: 153.09101, measured for $C_9H_{13}O_2$ $[M+H]^+$ 153.09105.

(2R,6R)-2-ethyl-6-ethynyl-3-methyltetrahydro-2H-pyran-3-ol 40

In a 50 mL Schlenk flask Li Cl (116 mg, 2.78 mmol, 1.1 eq.) and $ZnCl_2$ (507 mg, 3.72 mmol, 1.5 eq.) are dried with a heat gun under high vacuum (p < 10^{-2} mbar) and the flask is flushed three times with inert gas. After cooling to room temperature, dry THF (4 mL) is added and the resulting colorless solution is stirred for 5 min before being cooled to 0 °C. Then $TMSCH_2MgBr$ (1.0 M in THF, 7.4 mL, 7.4 mmol, 3.0 eq.) is added, the cooling bath is removed and the colorless suspension is stirred at room temperature for 20 min. Afterwards the suspension is cooled to -78 °C using a dry ice bath and MeLi (1.6 M in Et_2O, 2.3 mL, 3.72 mmol, 1.5 eq.) is added dropwise. The dry ice bath is exchanged for an ice bath and the reaction mixture is stirred for an additional 20 min at 0 °C, resulting in a colorless solution. Ketone **39** (378 mg, 2.48 mmol, 1.0 eq.), dissolved in dry THF (2 mL), is then added dropwise and the syringe is rinsed with additional dry THF (1 mL). After stirring for 30 min at 0 °C the reaction is quenched by addition of saturated NH_4Cl solution and the phases are separated. The aqueous phase is extracted three times with Et_2O (10 mL) and the combined organic phases are dried with $MgSO_4$. After filtration and removal of the solvent under reduced pressure (2R,6R)-2-ethyl-6-ethynyl-3-methyltetrahydro-2H-pyran-3-ol **40** (432 mg, 2.48 mmol, quantitative) is obtained as a separable 1:1.6 epimeric mixture which was used without further need for purification.

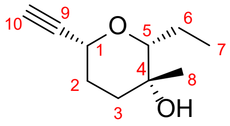

$C_{10}H_{16}O_2$
M = 168.24 g•mol^{-1}

TLC: R_f (pentane/acetone 5/1) = 0.35

$[a]^D_{20}$ = +104.5 (c = 1.26, $CHCl_3$)

^{1}H-NMR (500 MHz, $CDCl_3$, δ in ppm): 4.09 (dtr, J = 11.9 Hz, 2.3 Hz, 1H, H1), 3.05 (dd, J = 9.8 Hz, 2.7 Hz, 1H, H5), 2.49 (d, J = 2.2 Hz, 1H, H10), 2.32 (s, 1H, OH), 1.97 (dddd, J = 14.1 Hz, 13.7 Hz, 11.9 Hz, 4.3 Hz, 1H, $H2_{ax}$), 1.82-1.73 (m, 2H, $H2_{eq}$+H3), 1.62 (dqd, J = 15.0 Hz, 7.5 Hz, 2.7 Hz, 1H, H6), 1.57-1.47 (m, 2H, H3'+H6'), 1.10 (s, 3H, H8), 0.99 (tr, J = 7.5 Hz, 3H, H7).

^{13}C-NMR (125 MHz, $CDCl_3$, δ in ppm): 86.4 (C5), 82.9 (C9), 73.0 (C10), 68.3 (C1), 68.2 (C4), 37.5 (C3), 29.2 (C2), 24.5 (C8), 21.7 (C6), 11.0 (C7)

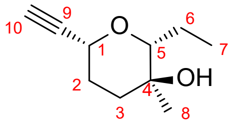

$C_{10}H_{16}O_2$
M = 168.24 g•mol^{-1}

TLC: R_f (pentane/acetone 5/1) = 0.31

$T_{m.p.}$ = 62-63 °C

$[\alpha]^D_{20}$ = +109.5 (c = 0.95, $CHCl_3$)

^{1}H-NMR (400 MHz, $CDCl_3$, δ in ppm): 4.09 (ddd, J = 10.4 Hz, 3.4 Hz, 2.1 Hz, 1H, H1), 2.99 (dd, J = 10.1 Hz, 2.0 Hz, 1H, H5), 2.46 (d, J = 2.1 Hz, 1H, H10), 1.93-1.77 (m, 3H, H2+H2'+H3), 1.69 (dqd, J = 14.1 Hz, 7.6 Hz, 2.0 Hz, 1H, H6), 1.62-1.53 (m, 1H, H3'), 1.36 (ddq, J = 14.1 Hz, 10.1 Hz, 7.2 Hz, 1H, H6'), 1.44-1.31 (brs, 1H, OH), 1.21 (s, 3H, H8), 1.01 (tr, J = 7.4 Hz, 3H, H7).

^{13}C-NMR (100 MHz, $CDCl_3$, δ in ppm): 86.9 (C5), 82.9 (C9), 72.7 (C10), 69.5 (C4), 68.2 (C1), 39.8 (C3), 31.8 (C2), 21.8 (C6), 20.4 (C8), 11.4 (C7).

(2R,3R,6R)-2-Ethyl-6-ethynyl-3-methyltetrahydro-2H-pyran 43

In an oven-dried round-bottom flask equipped with a reflux condenser the epimeric mixture of pyran **40** (466 mg, 2.77 mmol, 1.0 eq.) is dissolved in dry DCM (28 mL) and treated with Et_3SiH (2.7 mL, 16.6 mmol, 6.0 eq.), followed by dropwise addition of BF_3•OEt_2 (1.8 mL, 13.9 mmol, 5.0 eq.). The yellow reaction mixture is stirred at 40 °C for 2 d. After complete conversion of the starting material, the reaction mixture is quenched by addition of saturated $NaHCO_3$ solution and the phases are separated. The aqueous phase is extracted three times with Et_2O and the combined organic phases are dried over $MgSO_4$. After filtration, the solvent is carefully removed under reduced pressure (p>200 mbar). Purification of the crude product by flash chromatography (pentane/Et_2O 100:1 v:v) affords (2R,3R,6R)-2-Ethyl-6-ethynyl-3-methyltetrahydro-2H-pyran **43** (253 mg, 1.66 mmol, 60%) as a volatile, colorless liquid with a fruity odor. Absolute configuration of **43** was confirmed by 2D-NOESY experiments.

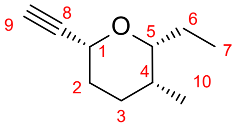

$C_{10}H_{16}O$
M = 152.24 g•mol^{-1}

TLC: R_f (pentane/Et_2O 100/1) = 0.25.

$[\alpha]^D_{20}$ = -48.2 (c = 1.14, $CHCl_3$).

^{1}H-NMR (400 MHz, $CDCl_3$, δ in ppm): 4.78-4.73 (m, 1H, H1), 3.42 (ddd, J = 9.4 Hz, 8.2 Hz, 2.8 Hz, 1H, H5), 2.43 (d, J = 2.3 Hz, H9), 1.92-1.81 (m, 1H, H2), 1.75-1.65 (m, 2H, H2'+H6), 1.65-1.53 (m, 2H, H3), 1.44-1.33 (m, 2H, H6'+H4), 0.93 (tr, J = 7.4 Hz, 3H, H7), 0.85 (d, J = 6.6 Hz, 3H, H10).

^{13}C-NMR (100 MHz, $CDCl_3$, δ in ppm): 82.8 (C8), 78.6 (C5), 74.1 (C9), 64.8 (C1), 34.8 (C4), 31.0 (C2), 28.4 (C3), 25.7 (C6), 18.1 (C10), 9.6 (C7).

HRMS: calculated for $C_{10}H_{17}O$ $[M+H]^+$: 153.12739, measured for $C_{10}H_{17}O$ $[M+H]^+$: 153.12744.

Additionally, 44.4 mg (292 µmol, 11%) of (2S,3R,6R)-2-ethyl-6-ethynyl-3-methyltetrahydro-2H-pyran **44** are obtained as a side fraction, containing trace amounts of **45**.

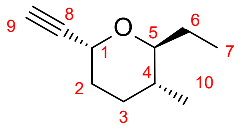

$C_{10}H_{16}O$
M = 152.24 g•mol^{-1}

TLC: R_f (pentane/Et_2O 100/1) = 0.24.

^{1}H-NMR (400 MHz, $CDCl_3$, δ in ppm): 4.09 (dtr, J = 11.7 Hz, 2.0 Hz, 1H, H1), 3.31-3.26 (m, 1H, H5), 2.45 (d, J = 2.0 Hz, H9), 1.92-1.81 (m, 1H, $H2_a$), 1.77-1.53 (m, 4H, $H2_b$+H3+$H6_a$), 1.46-1.31 (m, 1H, $H6_b$), 0.95 (d, J = 6.9 Hz, 3H, H10), 0.85 (tr, J = 7.5 Hz, 3H, H7).

^{13}C-NMR (100 MHz, $CDCl_3$, δ in ppm): 83.8 (C8), 82.6 (C5), 72.3 (C9), 68.9 (C1), 30.9 (C4), 29.6 (C3), 27.7 (C2), 26.2 (C6), 11.4 (C10), 10.4 (C7).

Tributyl((E)-2-((2R,5R,6R)-6-ethyl-5-methyltetrahydro-2H-pyran-2-yl)prop-1-en-1-yl)stannane 46

The reaction is carried out as described for stannane **38** using pyran **43** (107 mg, 700 µmol, 1.0 eq.), CuCN (75 mg, 840 µmol, 1.2 eq.), *n*BuLi (2.5 M in hexane, 670 µL, 1.68 mmol, 2.4 eq.), *n*Bu_3SnH (452 µL, 1.68 mmol, 2.4 eq.), MeI (870 µL, 14.0 mmol, 20 eq.), and dry DMPU (400 µL) in dry THF (8 mL). The pyrane, diluted in dry THF (2 mL), is added slowly along the flask wall and the syringe is rinsed with additional dry THF (1 mL) at -90 °C. Workup and purification by flash chromatography (puriFlash XS520Plus chromatography system using a Büchi FlashPure Select C18 30 µm cartridge, MeCN → MeCN/DCM 60:40 v:v) affords Tributyl((E)-2-((2R,5R,6R)-6-ethyl-5-methyltetrahydro-2H-pyran-2-yl)prop-1-en-1-yl)stannane **46** (308 mg, 673 µmol, 96%) as a colorless oil.

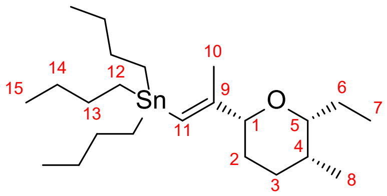

$C_{23}H_{46}OSn$
M = 457.33 g•mol^{-1}

$[\alpha]_{20}^{D}$ = -11.0 (c = 1.31, $CHCl_3$)

^{1}H-NMR (400 MHz, $CDCl_3$, δ in ppm): 5.75 (s, 1H, H10), 4.24 (quint, J = 3.5 Hz, 1H, H1), 3.02 (trd, J = 8.4 Hz, 3.2 Hz, 1H, H5), 2.02-1.93 (m, 1H, $H2_a$), 1.78 (s, 3H, H11), 1.74-1.71 (m, 1H, $H2_b$), 1.69-1.55 (m, 4H, H6+H3), 1.53-1.42 (m, 6H, H13), 1.35-1.22 (m, 6H, H14), 0.96 (tr, J = 7.4 Hz, 3H, H7), 0.91-0.86 (m, 15H, H15+H12), 0.85 (d, J = 6.4 Hz, 3H, H8).

^{13}C-NMR (100 MHz, $CDCl_3$, δ in ppm): 153.1 (C9), 124.1 (C10), 78.1 (C5), 76.4 (C1), 34.2 (C4), 29.4 (C13), 27.9 (C3), 27.5 (C14), 26.2 (C2), 25.9 (C6), 22.5 (C11), 18.4 (C8), 13.9 (C15), 10.4 (C7), 10.3 (C12).

HRMS: calculated for $C_{23}H_{47}OSn$ $[M+H]^+$: 459.26434, measured for $C_{23}H_{47}OSn$ $[M+H]^+$: 459.25617.

(((1S,2R,4R,6S)-2-Ethyl-1-methyl-3,7-dioxabicyclo[4.1.0]heptan-4-yl)ethynyl)trimethylsilane 47

In a 100 mL three necked round bottom flask alkene **37** (342 mg, 1.54 mmol, 1.0 eq.) is dissolved in MeCN (23 mL) and cooled to 0 °C. To this solution are successively added L-shi catalyst **31** (280 mg, 1.08 mmol, 0.7 eq.), *n*Bu_4NHSO_4 (65 mg, 0.19 mmol, 0.12 eq.), and a 10^{-4} M Na_2EDTA solution (15 mL). Afterwards, a K_2CO_3 solution (1.42 g, 10.3 mmol, 6.7 eq.) in water (85 mL) and a solution of oxone^{TM} (1.51 g, 2.46 mmol, 1.6 eq.) in 10^{-4} M Na_2EDTA (85 mL) are added simultaneously over 1 h using two syringe pumps under vigorous stirring. After complete addition, the colorless suspension is stirred for an additional hour at 0 °C before being quenched by addition of pentane (25 mL). The phases are separated and the aqueous phase is extracted three times with pentane (20 mL) and once with Et_2O (20 mL). The combined organic phases are dried with $MgSO_4$. After filtration and removal of the solvent under reduced pressure the crude product is purified by flash chromatography (pentane:Et_2O 10:1 v:v) affording the (((1S,2R,4R,6S)-2-Ethyl-1-methyl-3,7-dioxabicyclo[4.1.0]heptan-4-yl)ethynyl)trimethylsilane **47** (213 mg, 893 µmol, 58%) as well as (((1R,2R,4R,6R)-2-ethyl-1-methyl-3,7-dioxabicyclo[4.1.0]heptan-4-yl)ethynyl)trimethylsilane (21.0 mg, 89 µmol, 6%) as colorless oils.

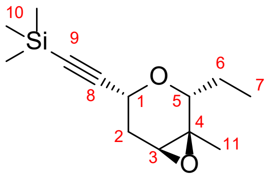

$C_{13}H_{22}O_2Si$
M = 238.40 g•mol^{-1}

TLC: R_f (pentane/Et_2O 10/1) = 0.33.

^{1}H-NMR (400 MHz, $CDCl_3$, δ in ppm): 4.20 (dd, J = 11.2 Hz, 3.1 Hz, 1H, H1), 3.70 (dd, J = 9.0 Hz, 3.3 Hz, 1H, H5), 3.11 (brs, 1H, H3), 2.24 (brdtr, J = 14.6 Hz, 2.6 Hz, 1H, $H2_a$), 2.09 (ddd, J = 14.6 Hz, 11.2 Hz, 1.5 Hz, 1H, $H2_b$), 1.74 (dqd, J = 14.4 Hz, 7.4 Hz, 3.5 Hz, 1H, $H6_a$), 1.61 (ddq, J = 14.4 Hz, 8.8 Hz, 7.3 Hz, 1H, $H6_b$), 1.28 (s, 3H, H11), 1.04 (tr, J = 7.4 Hz, 3H, H7), 0.16 (s, 9H, H10).

^{13}C-NMR (100 MHz, $CDCl_3$, δ in ppm): 104.3 (C8), 89.6 (C9), 80.0 (C5), 61.3 (C1), 59.0 (C3+C4), 33.1 (C2), 25.9 (C6), 20.3 (C11), 10.6 (C7), 0.0 (C10).

HRMS: calculated for $C_{13}H_{23}O_2Si$ $[M+H]^+$: 239.14618, measured for $C_{13}H_{23}O_2Si$ $[M+H]^+$: 239.14730.

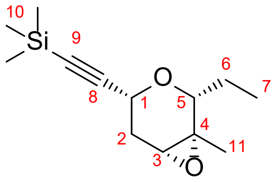

$C_{13}H_{22}O_2Si$
M = 238.40 g•mol^{-1}

TLC: R_f (pentane/Et_2O 10/1) = 0.19.

^{1}H-NMR (400 MHz, $CDCl_3$, δ in ppm): 3.97 (dd, J = 10.2 Hz, 5.8 Hz, 1H, H1), 3.51 (dd, J = 8.9 Hz, 3.0 Hz, 1H, H5), 3.03 (brd, J = 4.6 Hz, 1H, H3), 2.19-2.07 (m, 2H, H2), 1.79 (dqd, J = 14.8 Hz, 7.5 Hz, 2.9 Hz, 1H, $H6_a$), 1.65 (dquint, J = 14.8 Hz, 7.3 Hz, 1H, $H6_b$), 1.27 (s, 3H, H11), 1.05 (tr, J = 7.4 Hz, 3H, H7), 0.15 (s, 9H, H10).

^{13}C-NMR (100 MHz, $CDCl_3$, δ in ppm): 103.7 (C8), 90.3 (C9), 78.7 (C5), 64.5 (C1), 57.3 (C3), 55.6 (C4), 31.5 (C2), 23.9 (C6), 18.8 (C11), 10.5 (C7), -0.1 (C10).

HRMS: calculated for $C_{13}H_{23}O_2Si$ $[M+H]^+$: 239.14618, measured for $C_{13}H_{23}O_2Si$ $[M+H]^+$: 239.14673.

(2R,3R,4S)-2-Ethyl-3-methyl-3-((trimethylsilyl)ethynyl)tetrahydro-2H-pyran-4-ol 49

In a 10 mL round-bottom flask epoxide **47** (24 mg, 100 µmol, 1.0 eq.) is dissolved in HFIP (1 mL) and cooled to 0 °C. After sequential addition of Et_3SiH (32 µL, 200 µmol, 2.0 eq.) followed by $Bi(OTf)_3$ (3.0 mg, 5 µmol, 5 mol%) the colorless mixture is stirred for 20 min. The reaction is quenched by addition of saturated $NaHCO_3$ solution and the phases are separated. The aqueous phase is extracted three times with Et_2O and the combined organic phases are dried over $MgSO_4$. After filtration and removal of the solvents under reduced pressure, the crude product is purified via flash chromatography (pentane:acetone 15:1 v:v) affording (2R,3R,4S)-2-ethyl-3-methyl-3-((trimethylsilyl)ethynyl)tetrahydro-2H-pyran-4-ol **49** (6.1 mg, 25 µmol, 25%) as colorless needle shaped crystals. The structure of **49** was confirmed by 2D-NMR experiments and single crystal x-ray crystallography.

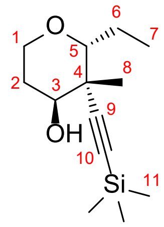

$C_{13}H_{24}O_2Si$
M = 240.42 g•mol^{-1}

TLC: R_f (pentane/acetone 10/1) = 0.28.

$T_{m.p.}$ = 78-80 °C.

$[\alpha]^{D}_{20}$ = +255.4 (c = 0.98, $CHCl_3$)

^{1}H-NMR (400 MHz, $CDCl_3$, δ in ppm): 3.89 (dd, J = 6.5 Hz, 3.4 Hz, 1H, H3), 3.80 (tr, J = 1.9 Hz, 1H, H1), 3.78 (dd, J = 6.3 Hz, 2.2 Hz, 1H, H1‘), 3.29 (dd, J = 10.0 Hz, 2.7 Hz, 1H, H5), 2.37 (dddd, J = 13.6 Hz, 10.6 Hz, 6.4 Hz, 2.6 Hz, 1H, H2), 1.68-1.50 (m, 4H, H2‘+H6+OH), 1.18 (s, 3H, H8), 0.98 (tr, J = 7.5 Hz, 3H, H7), 0.14 (s, 9H, H11).

^{13}C-NMR (100 MHz, $CDCl_3$, δ in ppm): 109.3 (C9), 88.0 (C10), 79.6 (C5), 72.0 (C3), 62.1 (C1), 41.5 (C4), 31.0 (C2), 23.5 (C6), 22.1 (C8), 11.1 (C7), 0.3 (C11).

Additionally, 8.2 mg (34 µmol, 34%) of compound **48** is obtained as a separable epimeric mixture.

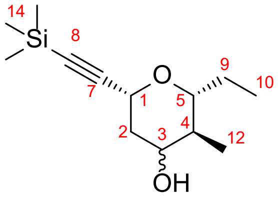

$C_{13}H_{24}O_2Si$
M = 240.42 g•mol^{-1}

diastereomer 1:

TLC: R_f (pentane/acetone 10/1) = 0.22.

^{1}H-NMR (400 MHz, $CDCl_3$, δ in ppm): 4.56 (ddd, J = 12.1 Hz, 2.5 Hz, 0.6 Hz, 1H, H1), 3.94 (quint, J = 2.6 Hz, 1H, H3), 3.72 (ddd, J = 7.6 Hz, 6.6 Hz, 2.5 Hz, 1H, H5), 2.03 (ddd, J = 14.4 Hz, 12.1 Hz, 2.8 Hz, 1H, $H2_a$), 1.71 (dtrd, J = 14.3 Hz, 2.6 Hz, 1.4 Hz, 1H, $H2_b$), 1.67-1.56 (m, 2H, H4+$H6_a$), 1.42-1.31 (m, 2H, $H6_b$+OH), 0.93 (d, J = 7.2 Hz, 3H, H11), 0.91 (tr, J = 7.5 Hz, 3H, H7), 0.16 (s, 9H, H10).

^{13}C-NMR (100 MHz, $CDCl_3$, δ in ppm): 105.1 (C8), 89.0 (C9), 76.3 (C5), 70.4 (C3), 63.8 (C1), 37.4 (C4), 35.5 (C2), 25.5 (C6), 10.8 (C11), 10.4 (C7), 0.1 (C10).

HRMS: calculated for $C_{13}H_{25}O_2Si$ $[M+H]^+$: 241.16183, measured for $C_{13}H_{25}O_2Si$ $[M+H]^+$: 241.16230.

diastereomer 2:

TLC: R_f (pentane/acetone 10/1) = 0.22.

^{1}H-NMR (400 MHz, $CDCl_3$, δ in ppm): 4.79 (dd, J = 5.6 Hz, 1.6 Hz, 1H, H1), 3.73-3.63 (m, 1H, H3), 3.50 (ddd, J = 10.3 Hz, 7.8 Hz, 2.8 Hz, 1H, H5), 2.05 (ddd, J = 12.4 Hz, 4.5 Hz, 1.6 Hz, 1H, $H2_a$), 1.78-1.67 (m, 2H, $H2_b$+$H6_a$), 1.49-1.39 (m, 2H, $H6_b$+OH), 1.30 (trq, J = 10.0 Hz, 6.6 Hz, 1H, H4), 0.99 (d, J = 6.5 Hz, 3H, H11), 0.94 (tr, J = 7.4 Hz, 3H, H7), 0.17 (s, 9H, H10).

^{13}C-NMR (100 MHz, $CDCl_3$, δ in ppm): 103.9 (C8), 91.8 (C9), 76.8 (C5), 70.6 (C3), 64.7 (C1), 43.5 (C4), 39.6 (C2), 25.5 (C6), 12.9 (C11), 9.5 (C7), 0.1 (C10).

HRMS: calculated for $C_{13}H_{25}O_2Si$ $[M+H]^+$: 241.16183, measured for $C_{13}H_{25}O_2Si$ $[M+H]^+$: 241.16220.

(2R,3R,6R)-2-ethyl-2-(trimethylsilyl)-6-((trimethylsilyl)ethynyl)tetrahydro-2H-pyran-3-ol S7

Synthesis is carried as described for pyran **33**, starting from (2S,3R)-aldehyde *ent*-**32** (1.19 g, 5.9 mmol, 1.0 eq.), $Zn(OTf)_2$ (2.36 g, 6.5 mmol, 1.1 eq.), (+)-NME (1.27 g, 7.1 mmol, 1.2 eq.), NEt_3 (980 µL, 7.1 mmol, 1.2 eq.) and TMS acetylene (4.2 mL, 29.5 mmol, 5.0 eq.) in a total of 60 mL dry toluene at 60 °C. The crude intermediate product is dissolved in dry DCM (60 mL) at 0 °C, treated dropwise with BF_3•OEt_2 (750 µL, 5.9 mmol, 1.0 eq.) and stirred for 10 min. Workup and purification via flash chromatography (pentane:Et_2O 5:1 v:v) affords (2R,3R,6R)-2-ethyl-2-(trimethylsilyl)-6-((trimethylsilyl)ethynyl)tetrahydro-2H-pyran-3-ol **S7** (1.13 g, 3.8 mmol, 64%) as a colorless oil.

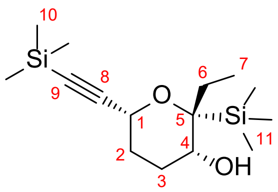

$C_{15}H_{30}O_2Si_2$
M = 298.57 g•mol^{-1}

TLC: R_f (pentane/acetone 10/1) = 0.34.

^{1}H-NMR (400 MHz, $CDCl_3$, δ in ppm): 4.49-4.39 (m, 1H, H1), 3.70 (dtr, J = 10.5 Hz, 2.4 Hz, 1H, H4), 2.27 (d, J = 10.7 Hz, 1H, OH), 2.00 (dq, J = 14.0 Hz, 7.6 Hz, 1H, H6), 1.95-1.87 (m, 2H, H2+H3), 1.71-1.65 (m, 2H, H2'+H3'), 1.61 (dq, J = 13.9 Hz, 7.7 Hz, 1H, H6'), 0.87 (tr, J = 7.6 Hz, 3H, H7), 0.16 (s, 9H, H10), 0.13 (s, 9H, H11).

^{13}C-NMR (100 MHz, $CDCl_3$, δ in ppm): 106.5 (C8), 88.1 (C9), 77.1 (C5), 67.0 (C4), 59.0 (C1), 26.8 (C2), 24.6 (C3), 24.1 (C6), 9.3 (C7), 0.1 (C10), -1.3 (C11).

HRMS: calculated for $C_{15}H_{31}O_2Si_2$ $[M+H]^+$: 299.18571, measured for $C_{15}H_{31}O_2Si_2$ $[M+H]^+$: 299.18444.

(2S,3R,6R)-2-Ethyl-6-ethynyltetrahydro-2H-pyran-3-ol S8

Stepwise deprotection is carried as described for pyran **34** using pyran **S7** (3.00 mmol, 1.0 eq.) with AcOH (180 µL, 3.15 mmol, 1.0 eq.) and TBAF (3.2 mL, 1.0 M in THF, 1.05 eq.) in 30 mL THF. After workup the monodesilylated intermediate is dissolved in dry THF (30 mL), treated with TBAF (1.0 M in THF, 12.0 mL,12.0 mmol, 4.0 eq.) and stirred at 45 °C for 18 h under inert atmosphere. After workup the crude product is purified by flash chromatography (pentane:acetone 5:1 v:v) affording (2S,3R,6R)-2-Ethyl-6-ethynyltetrahydro-2H-pyran-3-ol **S8** (428 mg, 2.77 mmol, 92%) as colorless crystals.

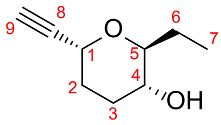

$C_9H_{14}O_2$
M = 154.21 g•mol^{-1}

TLC: R_f (pentane/acetone 5/1) = 0.14.

^{1}H-NMR (400 MHz, $CDCl_3$, δ in ppm): 4.70-4.64 (m, 1H, H1), 3.55 (trd, J = 8.7 Hz, 2.9 Hz, 1H, H5), 3.35-3.26 (m, 1H, H4), 2.46 (d, J = 2.2 Hz, 1H, H9), 2.00-1.93 (m, 1H, H3), 1.92-1.78 (m, 4H, H2+H3'+H6), 1.55-1.38 (m, 2H, H6'+OH), 0.98 (tr, J = 7.4 Hz, 3H, H7).

^{13}C-NMR (100 MHz, $CDCl_3$, δ in ppm): 81.9 (C8), 77.7 (C5), 74.7 (C9), 70.1 (C4), 64.1 (C1), 30.1 (C2), 29.0 (C3), 24.6 (C6), 9.7(C7).

HRMS: calculated for $C_9H_{13}O_2$ $[M-H]^-$: 153.09210, measured for $C_9H_{13}O_2$ $[M-H]^-$: 153.09217.

(2S,6R)-2-Ethyl-6-ethynyldihydro-2H-pyran-3(4H)-one S9

Oxidation is carried as described for pyran **39** using pyran **S8** (580 mg, 3.76 mmol, 1.0 eq.), pyridine (330 µL, 3.95 mmol, 1.05 eq.) and Dess-Martin periodinane (1.67 g, 3.95 mmol, 1.05 eq.) in dry DCM (40 mL) for 2 h at room temperature. After workup the crude product is purified by flash chromatography (pentane:acetone 10:1 v:v) affording (2S,6R)-2-ethyl-6-ethynyldihydro-2H-pyran-3(4H)-one **S9** (492 mg, 86%) as a colorless oil.

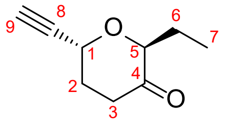

$C_9H_{12}O_2$
M = 152.19 g•mol^{-1}

TLC: R_f (pentane/acetone 10/1) = 0.33.

$[\alpha]_{20}^{D}$ = -122.3° (c = 1.03, $CHCl_3$).

^{1}H-NMR (400 MHz, $CDCl_3$, δ in ppm): 4.84 (ddd, J = 5.7 Hz, 3.6 Hz, 2.2 Hz, 1H, H1), 4.22 (dd, J = 7.5 Hz, 4.5 Hz, 1H, H5), 2.70 (dddtr, J = 16.1 Hz, 10.7 Hz, 6.2 Hz, 0.5 Hz, 1H, H3), 2.55 (d, J = 2.2 Hz, 1H, H9), 2.49 (dddtr, J = 16.1 Hz, 5.8 Hz, 4.8 Hz, 0.6 Hz, 1H, H3‘), 2.37 (ddtr, J = 13.6 Hz, 10.8 Hz, 5.6 Hz, 1H, H2), 2.14 (dddd, J = 13.6 Hz, 6.2 Hz, 5.0 Hz, 3.6 Hz, 1H, H2‘), 1.87 (dqd, J = 14.5 Hz, 7.5 Hz, 4.4 Hz, 1H, H6), 1.64 (dquint, J = 14.5 Hz, 7.4 Hz, 1H, H6‘), 0.95 (tr, J = 7.4 Hz, 1H, H7)

^{13}C-NMR (100 MHz, $CDCl_3$, δ in ppm): 208.3 (C4), 80.8 (C8), 79.4 (C5), 75.5 (C9), 63.2 (C1), 35.3 (C3), 31.0 (C2), 22.8 (C6), 9.7 (C7).

HRMS: calculated for $C_9H_{13}O_2$ $[M+H]^+$: 153.09101, measured for $C_9H_{13}O_2$ $[M+H]^+$: 153.09108.

(2S,6R)-2-Ethyl-6-ethynyl-3-methyltetrahydro-2H-pyran-3-ol 52

In a Schlenk flask LiCl (151 mg, 3.55 mmol, 1.1 eq.) and $ZnCl_2$ (661 mg, 4.85 mmol, 1.5 eq.) are dried with a heat gun under high vacuum (p < 10^{-2} mbar) and the flask is flushed three times with N_2. After cooling to room temperature, dry THF (10 mL) is added and the resulting colorless solution is stirred for 5 min before being cooled to 0 °C. Then $TMSCH_2MgCl$ (1.0 M in Et_2O, 9.7 mL, 9.7 mmol, 3.0 eq.) is added, the cooling bath is removed and the colorless suspension is stirred at room temperature for 20 min. Afterwards the suspension is cooled to 0 °C and MeLi (1.6 M in Et_2O, 3.0 mL, 4.85 mmol, 1.5 eq.) is added dropwise. The reaction mixture is stirred for 1 h at 0 °C, resulting in a colorless suspension. Ketone **S9** (492 mg, 3.29 mmol, 1.0 eq.), dissolved in dry THF (3 mL), is then added dropwise. After stirring for 1 h at 0 °C the reaction is quenched by addition of saturated NH_4Cl solution and the phases are separated. The aqueous phase is extracted three times with Et_2O and the combined organic phases are dried with $MgSO_4$. After filtration through a 3 cm pad of silica gel 60 and removal of the solvent under reduced pressure (2S,6R)-2-Ethyl-6-ethynyl-3-methyltetrahydro-2H-pyran-3-ol **52** (543 mg, 3.23 mmol, quantitative) is obtained as a barely separable 1:2.6 diastereomeric mixture which is used without further purification.

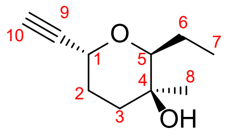

major diastereomer
$C_{10}H_{16}O_2$
M = 168.24 g•mol^{-1}

TLC: R_f (pentane/acetone 10/1) = 0.21.

^{1}H-NMR (400 MHz, $CDCl_3$, δ in ppm): 4.77 (brd, J = 5.4 Hz, 1H, H1), 3.64 (dd, J = 10.1 Hz, 2.6 Hz, 1H, H5), 2.43 (d, J = 2.2 Hz, 1H, H10), 2.19-1.85 (m, 2H, H2+OH), 1.93-1.84 (m, 1H,

H3), 1.67-1.56 (m, 3H, H2‘+H3‘+H6), 1.45 (ddq, J = 14.4 Hz, 10.1 Hz, 7.3 Hz, 1H, H6‘), 1.14 (s, 3H, H8) 0.96 (tr, J = 7.4 Hz, 3H, H7).

^{13}C-NMR (100 MHz, $CDCl_3$, δ in ppm): 81.7 (C9), 79.5 (C5), 74.3 (C10), 68.9 (C4), 64.9 (C1), 33.6 (C3), 26.8 (C2), 25.1 (C8), 21.3 (C6), 10.8 (C7).

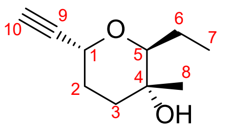

minor diastereomer
$C_{10}H_{16}O_2$
M = 168.24 g•mol^{-1}

HRMS: calculated for $C_{10}H_{15}O_2$ $[M-H]^-$: 167.10775, measured for $C_{10}H_{15}O_2$ $[M-H]^-$: 167.10748.

TLC: R_f (pentane/acetone 10/1) = 0.19.

^{1}H-NMR (400 MHz, $CDCl_3$, δ in ppm): 4.67-4.62 (m, 1H, H1), 3.55 (dd, J = 10.5 Hz, 2.4 Hz, 1H, H5), 2.45 (d, J = 2.2 Hz, 1H, H10), 1.98-1.85 (m, 2H, H3+H2), 1.82-1.63 (m, 3H, H2‘+H3‘+H6), 1.56 (s, 1H, OH), 1.34 (ddq, J = 14.2 Hz, 10.4 Hz, 7.2 Hz, 1H, H6‘), 1.15 (s, 3H, H8) 0.99 (tr, J = 7.4 Hz, 3H, H7).

^{13}C-NMR (100 MHz, $CDCl_3$, δ in ppm): 82.1 (C9), 81.0 (C5), 74.1 (C10), 69.9 (C4), 63.7 (C1), 35.4 (C3), 29.4 (C2), 21.3 (C6), 20.7 (C8), 11.0 (C7).

(2R,3S,6R)-2-Ethyl-6-ethynyl-3-methyltetrahydro-2H-pyran 45

In an oven dried round bottom flask equipped with a reflux condenser the epimeric mixture of pyran **52** (543 mg, 3.23 mmol, 1.0 eq.) is dissolved in dry DCM (25 mL) and treated with Et_3SiH (3.1 mL, 19.4 mmol, 6.0 eq.), followed by dropwise addition of BF_3•OEt_2 (1.8 mL, 16.2 mmol, 5.0 eq.). The yellow reaction mixture is stirred at 45 °C for 2 d. After complete conversion of the starting material, the reaction mixture is quenched by addition of saturated $NaHCO_3$ solution and the phases are separated. The aqueous phase is extracted three times with Et_2O and the combined organic phases are dried over $MgSO_4$. After filtration the solvent is carefully removed under reduced pressure (p>200 mbar). Purification of the crude product by flash chromatography (pentane/Et_2O 150:1 v:v) affords pure (2R,3S,6R)-2-Ethyl-6-ethynyl-3-methyltetrahydro-2H-pyran **45** (394 mg, 2.41 mmol, 75%) as a volatile, colorless liquid with a fruity odor.

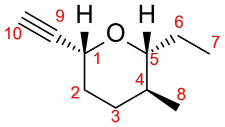

$C_{10}H_{16}O$
M = 152.24 g•mol^{-1}

TLC: R_f (pentane/Et_2O 100/1) = 0.09.

$[\alpha]^{D}_{20}$ = +88.0 (c = 1.02, $CHCl_3$)

^{1}H-NMR (500 MHz, $CDCl_3$, δ in ppm): 4.05 (dtr, J = 11.4 Hz, 2.3 Hz, 1H, H1), 2.86 (ddd, J = 9.6 Hz, 8.1 Hz, 2.7 Hz, 1H, H5), 2.44 (d, J = 2.3 Hz, 1H, H10), 1.86-1.81 (m, 1H, H2), 1.81-1.75 (m, 1H, H3), 1.75-1.67 (m, 2H, H2‘+H6), 1.68-1.35 (m, 2H, H4+H6‘), 1.23-1.14 (m, 1H, H3‘), 0.96 (tr, J = 7.4 Hz, 3H, H7), 0.81 (d, J = 6.7 Hz, 3H, H8).

^{13}C-NMR (125 MHz, $CDCl_3$, δ in ppm): 85.3 (C5), 83.8 (C9), 72.2 (C10), 67.9 (C1), 34.1 (C4), 33.3 (C2), 32.8 (C3), 25.9 (C6), 17.8 (C8), 9.8 (C7).

HRMS: calculated for $C_{10}H_{17}O_2$ $[M+H]^+$: 153.12739, measured for $[M+H]^+$: 153.12732.

Tributyl((E)-2-((2R,5S,6R)-6-ethyl-5-methyltetrahydro-2H-pyran-2-yl)prop-1-en-1-yl)stannane 53

The reaction is carried out as described for stannane **38** using pyran **45** (122 mg, 800 µmol, 1.0 eq.), CuCN (86 mg, 960 µmol, 1.2 eq.), *n*BuLi (2.5 M in hexane, 770 µL, 1.92 mmol, 2.4 eq.), nBu_3SnH (520 µL, 1.92 mmol, 2.4 eq.), MeI (1.0 mL, 16.0 mmol, 20 eq.), and dry DMPU (460 µL) in dry THF (11 mL). The pyrane, diluted in dry THF (3 mL), is added slowly along the flask wall and the syringe is rinsed with additional dry THF (2 mL) at -90 °C. Workup and purification by flash chromatography (puriFlash XS520Plus chromatography system using a Büchi FlashPure Select C18 30 µm cartridge, MeCN → MeCN/DCM 60:40 v:v) affords tributyl((E)-2-((2R,5S,6R)-6-ethyl-5-methyltetrahydro-2H-pyran-2-yl)prop-1-en-1-yl)stannane **53** (320 mg, 701 µmol, 88%) as a colorless oil.

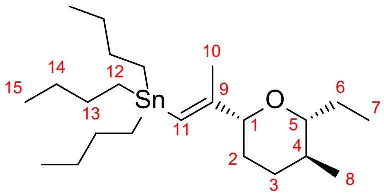

$C_{23}H_{46}OSn$
M = 457.33 g•mol^{-1}

$[\alpha]_{20}^{D}$ = +25.0 (c = 1.00, $CHCl_3$)

^{1}H-NMR (500 MHz, $CDCl_3$, δ in ppm): 5.79 (s, 1H, H10), 3.68 (brd, J = 11.1 Hz, 1H, H1), 2.92 (ddd, J = 9.5 Hz, 7.9 Hz, 2.9 Hz, 1H, H5), 1.83-1.76 (m, 1H, $H3_a$), 1.78 (s, 3H, H11), 1.76-1.67 (m, 2H, $H2_a$+$H6_a$), 1.53-1.41 (m, 6H, H13), 1.36-1.27 (m, 9H, $H2_b$+H4+$H6_b$+H14), 1.26-1.17 (m, 1H, $H3_b$), 0.97 (tr, J = 7.4 Hz, 3H, H7), 0.91-0.86 (m, 12H, H12+H15), 0.82 (d, J = 6.5 Hz, 3H, H8).

^{13}C-NMR (125 MHz, $CDCl_3$, δ in ppm): 155.1 (C9), 121.2 (C10), 84.5 (C5), 82.8 (C1), 34.5 (C4), 33.5 (C3), 31.4 (C2), 29.4 (C13), 27.5 (C14), 26.1 (C6), 21.6 (C11), 17.9 (C8), 13.9 (C15), 10.2 (C12), 9.7 (C7)

HRMS: calculated for $C_{23}H_{47}OSn$ $[M+H]^+$: 459.26434, measured for $C_{23}H_{47}OSn$ $[M+H]^+$: 459.26324.

General procedure for the microwave-assisted π-allyl Stille coupling

An oven dried microwave vial equipped with a magnetic stirring bar is charged with the corresponding lactone (1.0 eq.), stannane (1.05 eq.) and finely ground molecular sieves 4 Å and the vessel is flushed three times with N_2. Subsequently, the mixture is suspended in a 2:1 mixture of dry THF and dry DMF (0.05-0.1 M) and Pd_2dba_3 (5-10 mol%) is added. The resulting dark red to violet suspension is irradiated in a microwave reactor at 40 W for 1 min per cycle for a total of up to 10 cycles with air cooling (T_{max} = 40–50 °C). Between each cycle the reaction mixture is cooled to room temperature for 1-2 min. After the first cycle, the suspension typically turns yellow and Pd^0 precipitates as a black solid after several cycles. After complete conversion of the starting material the reaction mixture is filtered and the solvent is evaporated under vacuum. Purification via flash chromatography (pentane:acetone with 1-2 vol% NEt_3) affords the desired jerangolid in 30-34% yield.

14-*epi* jerangolid E 55

Synthesis is performed according to the general procedure using lactone **54** (62.0 mg, 200 µmol, 1.0 eq.), stannane **46** (113 mg, 210 µmol, 1.05 eq.), finely ground molecular sieves 4 Å (40 mg), and Pd_2dba_3 (18.0 mg, 20 µmol, 10 mol%) in a 2:1 mixture of dry THF and dry DMF (3 mL). Microwave irradiation is carried out at 40 W for 10 cycles of 1 min each under air cooling (T_{max} = 48-51 °C). After filtration and removal of the solvent in vacuo the crude product is purified via flash chromatography (pentane/acetone 15:1 v:v with 1 vol% NEt_3) affording 14-*epi* jerangolid E **55** (22.1 mg, 61.0 µmol, 31%) as a colorless resin.

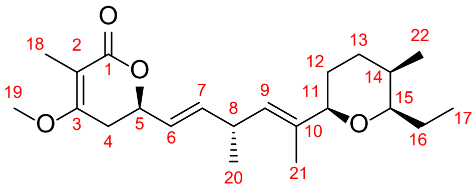

$C_{22}H_{34}O_4$
M = 362.51 g•mol^{-1}

TLC: R_f (pentane/acetone 10/1) = 0.11.

^{1}H-NMR (400 MHz, CD_3OD, δ in ppm): 5.87 (ddd, J = 15.6 Hz, 6.2 Hz, 1.0 Hz, 1H, H7), 5.62 (ddd, J = 15.6 Hz, 6.6 Hz, 1.4 Hz, 1H, H6), 5.27 (dquint, J = 9.0 Hz, 1.4 Hz, 1H, H9), 4.83 (brddd, J = 11.0 Hz, 6.6 Hz, 4.4 Hz, 1H, H5), 4.11 (tr, J = 4.9 Hz, 1H, H11), 3.84 (s, 3H, H19), 3.27-3.16 (m, 1H, H8), 3.11 (ddd, J = 8.8 Hz, 6.6 Hz, 4.1 Hz, 1H, H15), 2.86 (ddq, J = 17.4 Hz, 4.3 Hz, 1.1 Hz, 1H, $H4_a$), 2.63 (ddq, J = 17.3 Hz, 11.1 Hz, 2.0 Hz, 1H, $H4_b$), 1.98-1.89 (m, 1H, $H12_a$), 1.74-1.68 (m, 2H, $H12_b$+$H13_a$), 1.71 (dd, J = 1.8 Hz, 1.0 Hz, 3H, H18), 1.68-1.54 (m, 2H, H16), 1.67 (dd, J = 1.3 Hz, 0.6 Hz, 3H, H21), 1.49-1.42 (m, 1H, H14), 1.42-1.34 (m, 1H, $H13_b$), 1.09 (d, J = 6.9 Hz, 3H, H20), 0.95 (tr, J = 7.5 Hz, 3H, H17), 0.93 (d, J = 6.7 Hz, 3H, H22).

^{13}C-NMR (100 MHz, CD_3OD, δ in ppm): 17.1 (C1), 169.2 (C3), 140.2 (C7), 135.9 (C10), 130.9 (C9), 126.7 (C6), 102.7 (C2), 79.9 (C15), 76.9 (C5), 76.2 (C11), 56.3 (C19), 36.2 (C8), 34.5 (C14), 30.4 (C4), 28.1 (C13), 26.4 (C12), 26.2 (C16), 21.0 (C20), 18.7 (C22), 13.8 (C21), 10.7 (C17), 8.8 (C18).

HRMS: calculated for $C_{22}H_{35}O_4$ $[M+H]^+$: 363.25299, measured for $C_{22}H_{35}O_4$ $[M+H]^+$: 363.25654.

Table S1. Comparison of the ^{1}H-NMR spectra of synthetic 14-epi jerangolid E **55**, the natural product isolated by Höfle *et al.*,[7,8] and the compound synthesized by Hahn *et al.*[9]

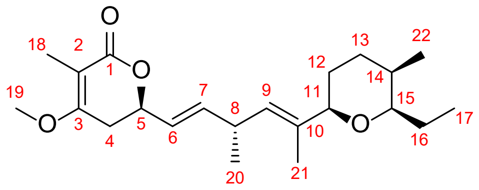

55

Pos.	isolated jerangolid E **2**[8] ^{1}H-NMR (400 MHz, CD_3OD, δ in ppm, J [Hz])	synthetic jerangolid E **2** (Hahn *et al.*[9]) ^{1}H-NMR (500 MHz, CD_3OD, δ in ppm, J [Hz])	synthetic 14-*epi* jerangolid E **55** ^{1}H-NMR (400 MHz, CD_3OD, δ in ppm, J [Hz])
$H4_a$	2.88 (ddq, 17.3, 4.1, 0.8)	2.84 (ddq, 17.6, 4.3, 1.1)	2.86 (ddq, 17.4, 4.3, 1.1)
$H4_b$	2.67 (ddq, 17.3, 11.3, 2.0)	2.67 (ddq, 17.4, 11.5, 2.0)	2.63 (ddq, 17.3, 11.1, 2.0)
H5	4.83 (brdd, 6.7, 4.4)	4.80 (ddd, 11.2, 6.6, 4.4)	4.83 (brddd, 11.0, 6.6, 4.4)
H6	5.63 (ddd, 15.6, 6.6, 1.4)	5.59 (ddd, 15.5, 6.6, 1.4)	5.62 (ddd, 15.6, 6.6, 1.4)
H7	5.87 (ddd, 15.6, 6.2, 0.8)	5.83 (ddd, 15.6, 6.4, 1.0)	5.87 (ddd, 15.6, 6.2, 1.0)
H8	3.21 (m)	3.17 (m)	3.27-3.16 (m)
H9	5.29 (ddq, 9.1, 1.3, 1.2)	5.25 (ddq, 9.1, 1.1, 1.2)	5.27 (dquint, 9.0, 1.4)
H11	3.67 (brd, 10.8)	3.63 (brd, 10.8)	4.11 (tr, 4.9)
$H12_a$	1.68 (m)	1.64 (m)	1.98-1.89 (m)
$H12_b$	1.48 (m)	1.44 (m)	1.74-1.68 (m)
$H13_a$	1.86 (m)	1.82 (dddd, 12.5, 3.5, 3.4, 3.1)	1.74-1.68 (m)
$H13_b$	1.30 (m)	1.26 (m)	1.42-1.34 (m)
H14	1.35 (m)	1.31 (m)	1.49-1.42 (m)
H15	2.96 (ddd, 9.2, 7.8, 2.8)	2.92 (ddd, 9.1, 8.0, 2.7)	3.11 (ddd, 8.8, 6.6, 4.1)
$H16_a$	1.75 (m)	1.71 (m)	1.68-1.54
$H16_b$	1.44 (m)	1.40 (m)	1.68-1.54
H17	0.99 (dd, 7.5, 7.3)	0.95 (tr, 7.4)	0.95 (tr, 7.5)
H18	1.74 (dd, 1.7, 1.0)	1.70 (dd, 1.7, 1.2)	1.71 (dd, 1.8, 1.0)
H19	3.88 (s)	3.84 (s)	3.84 (s)
H20	1.13 (d, 6.9)	1.09 (d, 6.9)	1.09 (d, 6.9)
H21	1.69 (d, 1.3)	1.65 (d, 1.4)	1.67 (dd, 1.3, 0.6)
H22	0.87 (d, 6.4)	0.83 (d, 6.4)	0.93 (d, 6.7)

Table S2. Comparison of the ^{13}C-NMR spectra of synthetic 14-epi jerangolid E **55**, the natural product isolated by Höfle *et al.*,[7,8] and the compound synthesized by Hahn *et al.*[9]

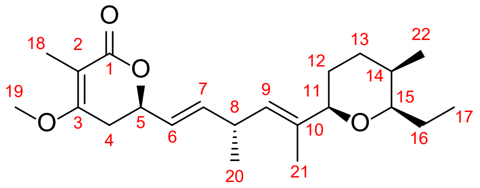

55

Pos.	isolated jerangolid E **2**[8] ^{13}C-NMR (100 MHz, CD_3OD, δ in ppm)	synthetic jerangolid E **2** (Hahn *et al.*[9]) ^{13}C-NMR (125 MHz, CD_3OD, δ in ppm)	synthetic 14-*epi* jerangolid E **55** ^{13}C-NMR (100 MHz, CD_3OD, δ in ppm)	Δδ
1	171.4	171.4	171.3	-0.1
2	102.7	102.6	102.7	0.0
3	169.3	169.4	169.2	-0.1
4	30.3	30.3	30.4	+0.1
5	76.9	77.0	76.9	0.0
6	126.4	126.4	126.7	+0.3
7	140.3	140.3	140.2	-0.1
8	35.8	35.8	36.2	+0.4
9	128.9	128.9	130.9	+2.0
10	137.8	137.8	135.9	-1.9
11	83.5	83.5	76.2	-7.3
12	32.0	32.0	26.4	-5.6
13	34.3	34.2	28.1	-6.2
14	35.6	35.6	34.5	-0.9
15	85.9	85.9	79.9	-6.0
16	26.9	26.9	26.2	-0.7
17	9.9	9.9	10.7	+0.8
18	8.8	8.8	8.8	0.0
19	56.3	56.3	56.3	0.0
20	21.0	21.0	21.0	0.0
21	13.4	13.4	13.8	+0.4
22	18.0	18.0	18.7	+0.7

(R)-6-((R,1E,4E)-5-((2R,5R,6R)-6-Ethyl-5-methyltetrahydro-2H-pyran-2-yl)-3-methylhexa-1,4-dien-1-yl)-4-methoxy-3-(((4-methoxybenzyl)oxy)methyl)-5,6-dihydro-2H-pyran-2-one 56

Synthesis is performed according to the general procedure using lactone **25** (88.9 mg, 200 µmol, 1.0 eq.), stannane **46** (113 mg, 210 µmol, 1.05 eq.), finely ground molecular sieves 4 Å (40 mg), and Pd_2dba_3 (18.0 mg, 20 µmol, 10 mol%) in a 2:1 mixture of dry THF and dry DMF (3 mL). Microwave irradiation is carried out at 40 W for 10 cycles of 1 min each under air cooling. After filtration and removal of the solvent in vacuo the crude product is purified via flash chromatography (pentane/acetone10:1 v:v with 1 vol% NEt_3) affording the title compound **56** (30.4 mg, 60.9 µmol, 31%) as a yellow resin.

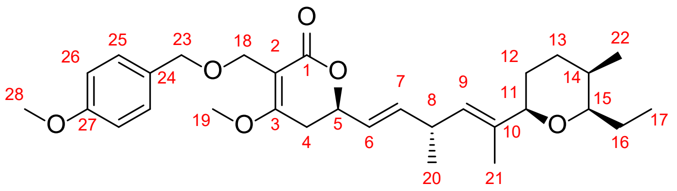

$C_{30}H_{42}O_6$
M = 498.66 g•mol^{-1}

TLC: R_f (pentane/acetone 5/1) = 0.17.

^{1}H-NMR (400 MHz, $CDCl_3$, δ in ppm): 7.28 (m, 2H, H25), 6.85 (m, 2H, H26), 5.81 (ddd, J = 15.6 Hz, 5.9 Hz, 0.8 Hz, 1H, H7), 5.52 (ddd, J = 15.6 Hz, 6.8 Hz, 1.5 Hz, 1H, H6), 5.18 (dquint, J = 9.0 Hz, 1.3 Hz, 1H, H9), 4.73 (brddd, J = 11.0 Hz, 6.8 Hz, 4.8 Hz, 1H, H5), 4.48 (s, 2H, H23), 4.30 (d, J = 10.7 Hz, 1H, $H18_a$), 4.27 (d, J = 10.7 Hz, 1H, $H18_b$), 4.08 (brtr, J = 4.7 Hz, 1H, H11), 3.81 (s, 3H, H19), 3.78 (s, 3H, H28), 3.20-3.11 (m, 1H, H8), 3.08 (ddd, J = 8.4 Hz, 6.4 Hz, 4.6 Hz, 1H, H15), 2.62 (dd, J = 17.2 Hz, 4.6 Hz, 1H, $H4_a$), 2.54 (dd, J = 17.2 Hz, 11.0 Hz, 1H, $H4_b$), 1.92-1.82 (m, 1H, $H12_a$), 1.71-1.66 (m, 1H, $H13_a$), 1.64 (dd, J = 1.2 Hz, 0.6 Hz, 3H, H21), 1.66-1.60 (m, 1H, $H12_b$), 1.60-1.51 (m, 2H, H16), 1.50-1.43 (m, 1H, H14), 1.36-1.28 (m, 1H, $H13_b$), 1.07 (d, J = 6.9 Hz, 3H, H20), 0.94 (tr, J = 7.4 Hz, 3H, H17), 0.91 (d, J = 6.8 Hz, 3H, H22).

^{13}C-NMR (100 MHz, $CDCl_3$, δ in ppm): 169.6 (C3), 167.4 (C1), 159.2 (C27), 139.9 (C7), 135.3 (C10), 131.0 (C24), 129.5 (C25), 129.0 (C9), 124.7 (C6), 113.7 (C26), 105.6 (C2), 78.6 (C15), 75.0 (C5), 74.8 (C11), 72.4 (C23), 61.0 (C18), 56.1 (C19), 55.4 (C28), 34.9 (C8), 33.1 (C14), 30.3 (C4), 27.2 (C13), 25.4 (C12+C16), 20.6 (C20), 18.5 (C22), 13.6 (C21), 10.4 (C17).

HRMS: calculated for $C_{30}H_{43}O_6$ $[M+H]^+$: 499.30542, measured for $C_{30}H_{43}O_6$ $[M+H]^+$: 499.30723.

14-*epi* jerangolid H 57

PMB ether **56** (28.0 mg, 56 µmol, 1.0 eq.) is dissolved in DCM (2 mL) and treated with H_2O (100 µL). Afterwards DDQ (15.3 mg, 67 µmol, 1.2 eq.) is added in one portion at 0 °C and the resulting dark green biphasic mixture is stirred vigorously turning into a red-brown suspension after approximately 3 min. The reaction is stirred for 2 h at 0 °C, then quenched by addition of saturated $NaHCO_3$ solution. The phases are separated and the aqueous phase is extracted three times with ethyl acetate. The combined organic phases are dried with $MgSO_4$, filtered through a pad of celite and the solvent is removed under reduced pressure. Purification via flash chromatography (pentane:acetone 2:1 v:v) affords 14-*epi* jerangolid H **57** (18.1 mg, 48.0 µmol, 85%) as a pale yellow resin.

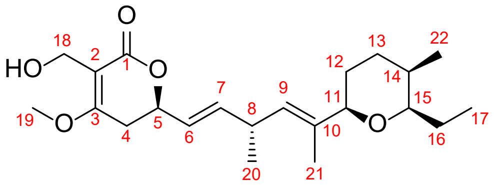

$C_{22}H_{34}O_5$
M = 378.51 g•mol^{-1}

TLC: R_f (pentane/acetone 2/1) = 0.39.

^{1}H-NMR (400 MHz, CD_3OD, δ in ppm): 5.89 (ddd, J = 15.5 Hz, 6.2 Hz, 0.9 Hz, 1H, H7), 5.63 (ddd, J = 15.5 Hz, 6.8 Hz, 1.3 Hz, 1H, H6), 5.28 (dquint, J = 9.0 Hz, 1.3 Hz, 1H, H9), 4.86 (brddd, J = 10.7 Hz, 6.2 Hz, 3.7 Hz, 1H, H5), 4.30 (s, 2H, H18), 4.12 (brtr, J = 4.7 Hz, 1H, H11), 3.89 (s, 3H, H19), 3.28-3.17 (m, 1H, H8), 3.12 (ddd, J = 8.6 Hz, 6.5 Hz, 4.0 Hz, 1H, H15), 2.92 (dd, J = 17.5 Hz, 4.1 Hz, 1H, $H4_a$), 2.69 (dd, J = 17.5 Hz, 11.4 Hz, 1H, $H4_b$), 1.99-1.89 (m, 1H, $H12_a$), 1.76-1.70 (m, 1H, $H13_a$), 1.69-1.63 (m, 1H, $H12_b$), 1.67 (dd, J = 1.3 Hz, 0.6 Hz, 3H, H21), 1.62-1.53 (m, 2H, H16), 1.53-1.44 (m, 1H, H14), 1.44-1.35 (m, 1H, $H13_b$), 1.11 (d, J = 6.8 Hz, 3H, H20), 0.95 (tr, J = 7.4 Hz, 3H, H17), 0.94 (d, J = 6.6 Hz, 3H, H22).

^{13}C-NMR (100 MHz, CD_3OD, δ in ppm): 172.6 (C3), 170.3 (C1), 140.5 (C7), 135.9 (C10), 130.9 (C9), 126.4 (C6), 107.3 (C2), 80.0 (C15), 76.8 (C5), 76.2 (C11), 56.8 (C19), 54.0 (C18), 36.2 (C8), 34.5 (C14), 30.6 (C4), 28.1 (C13), 26.4 (C12), 26.2 (C16), 21.0 (C20), 18.7 (C22), 13.8 (C21), 10.7 (C17).

HRMS: calculated for $C_{22}H_{35}O_5$ $[M+H]^+$: 379.24791, measured for $C_{22}H_{35}O_5$ $[M+H]^+$: 379.25034.

Table S3. Comparison of the ^{1}H-NMR spectra of synthetic 14-*epi* jerangolid H **57** and natural jerangolid H **3** isolated by Höfle *et al.*[7,8]

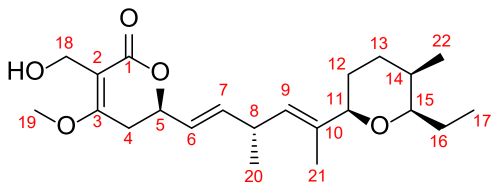

57

Pos.	isolated jerangolid H **3**[8]<br>^{1}H-NMR (400 MHz, CD_3OD, δ in ppm, J [Hz])	synthetic 14-*epi* jerangolid H **57**<br>^{1}H-NMR (400 MHz, CD_3OD, δ in ppm, J [Hz])
4_a	2.95 (dd, 17.5, 4.2)	2.86 (dd, 17.5 ,4.1)
4_b	2.73 (dd, 17.5, 11.3)	2.63 (dd, 17.5,11.4)
5	4.88 (m)	4.83 (brddd,10.7, 6.2, 3.7)
6	5.64 (ddd, 15.5, 6.7, 1.4)	5.63 (ddd, 15.5, 6.8, 1.3)
7	5.89 (ddd, 15.5, 6.4, 1.0)	5.89 (ddd, 15.5, 6.2, 0.9)
8	3.22 (m)	3.28-3.17 (m)
9	5.30 (ddq, 9.0, 1.4, 1.3)	5.28 (dquint, 9.0, 1.3)
11	3.68 (brd, 10.1)	4.12 (tr, 4.7)
12_a	1.68 (m)	1.99-1.89 (m)
12_b	1.49 (m)	1.69-1.63 (m)
13_a	1.85 (m)	1.76-1.70 (m)
13_b	1.28 (m)	1.44-1.35 (m)
14	1.34 (m)	1.53-1.44 (m)
15	2.96 (m)	3.12 (ddd, 8.6 Hz, 6.5, 4.0)
16_a	1.76 (dq, 7.4, 2.9)	1.62-1.53 (m)
16_b	1.44 (m)	1.62-1.53 (m)
17	0.99 (dd, 7.4, 7.3)	0.95 (tr, 7.4)
18	4.34 (s)	4.30 (s)
19	3.93 (s)	3.89 (s)
20	1.13 (d, 6.8)	1.11 (d, 6.8)
21	1.70 (d, 1.4)	1.67 (dd, 1.3 , 0.6)
22	0.87 (d, 6.2)	0.94 (d, 6.6)
OH	2.87 (s)[a]	-

[a] measured in DMSO-d_6

Table S4. Comparison of the ^{13}C-NMR spectra of synthetic 14-*epi* jerangolid H **57** and natural jerangolid H **3** isolated by Höfle *et al.*[7,8]

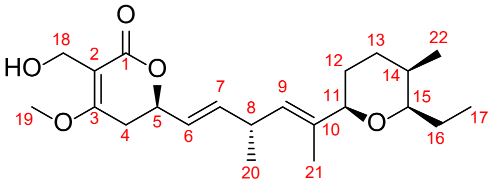

57

Pos.	isolated jerangolid H **3**[8] ^{13}C-NMR (100 MHz, CD_3OD, δ in ppm)	synthetic 14-*epi* jerangolid H **57** ^{13}C-NMR (100 MHz, CD_3OD, δ in ppm)	Δδ
1	170.3	170.3	0.0
2	107.3	107.3	0.0
3	172.6	172.6	0.0
4	30.5	30.6	+0.1
5	76.9	76.8	-0.1
6	126.2	126.4	+0.2
7	140.5	140.5	0.0
8	35.8[a)]	36.2	+0.4
9	128.9	130.9	+2.0
10	137.9	135.9	-2.0
11	83.5	76.2	-7.3
12	32.0	26.4	-5.6
13	34.3	28.1	-6.2
14	35.6[a)]	34.5	-1.1
15	85.9	80.0	-5.9
16	26.9	26.2	-0.7
17	9.9	10.7	+0.8
18	54.0	54.0	0.0
19	56.8	56.8	0.0
20	21.0	21.0	0.0
21	13.4	13.8	+0.4
22	18.0	18.7	+0.7

[a)]assignment ambiguous.

Jerangolid E 2

Synthesis is performed according to the general procedure using lactone **54** (62.0 mg, 200 µmol, 1.0 eq.), stannane **53** (101 mg, 210 µmol, 1.05 eq.), finely ground molecular sieves 4 Å (40 mg) and Pd_2dba_3 (18.2 mg, 20 µmol, 10 mol%) in a 2:1 mixture of dry THF and dry DMF (3 mL). Microwave irradiation is carried out at 40 W for 10 cycles of 1 min each under air cooling (T_{max} = 44-47 °C). After filtration and removal of the solvent in vacuo the crude product is purified via flash chromatography (pentane/acetone 20:1 v:v with 1 vol% NEt_3) affording jerangolid E **2** (23.8 mg, 65.7 µmol, 33%) as a yellow resin.

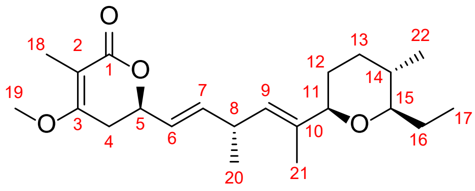

$C_{22}H_{34}O_4$
M = 362.51 g•mol^{-1}

TLC: R_f (pentane/acetone 10/1) = 0.18.

^{1}H-NMR (500 MHz, CD_3OD, δ in ppm): 5.83 (ddd, J = 15.5 Hz, 6.4 Hz, 1.0 Hz, 1H, H7), 5.59 (ddd, J = 15.6 Hz, 6.5 Hz, 1.4 Hz, 1H, H6), 5.25 (dquint, J = 9.0 Hz, 1.2 Hz, 1H, H9), 4.83 (brddd, J = 11.1 Hz, 6.5 Hz, 4.4 Hz, 1H, H5), 3.83 (s, 3H, H19), 3.63 (brd, J = 11.0 Hz, 1H, H11), 3.23-3.13 (m, 1H, H8), 2.91 (ddd, J = 9.4 Hz, 8.0 Hz, 2.7 Hz, 1H, H15), 2.84 (ddq, J = 17.4 Hz, 4.1 Hz, 1.0 Hz, 1H, $H4_a$), 2.62 (ddq, J = 17.4 Hz, 11.5 Hz, 2.0 Hz, 1H, $H4_b$), 1.84-1.78 (m, 1H, $H13_a$), 1.75-1.68 (m, 1H, $H16_a$), 1.71 (dd, J = 1.7 Hz, 0.9 Hz, 3H, H18), 1.68-1.61 (m, 1H, $H12_a$), 1.65 (dd, J = 1.3 Hz, 3H, H21), 1.48-1.36 (m, 2H, $H12_b$+$H16_b$), 1.35-1.22 (m, 2H, $H13_b$+H14), 1.09 (d, J = 6.9 Hz, 3H, H20), 0.95 (tr, J = 7.4 Hz, 3H, H17), 0.83 (d, J = 6.5 Hz, 3H, H22).

^{13}C-NMR (125 MHz, CD_3OD, δ in ppm): 171.4 (C1), 169.4 (C3), 140.3 (C7), 137.8 (C10), 128.9 (C9), 126.4 (C6), 102.7 (C2), 85.9 (C15), 83.5 (C11), 76.9 (C5), 56.3 (C19), 35.8 (C8), 35.6 (C14), 34.2 (C13), 32.0 (C12), 30.3 (C4), 26.9 (C16), 21.0 (C20), 18.0 (C22), 13.4 (C21), 9.9 (C17), 8.8 (C18).

HRMS: calculated for $C_{22}H_{35}O_4$ $[M+H]^+$: 363.25299, measured for $C_{22}H_{35}O_4$ $[M+H]^+$: 363.25250.

Table S5. Comparison of the ^{1}H-NMR spectra of synthetic jerangolid E **2**, the natural product isolated by Höfle *et al.*,[7,8] and the compound synthesized by Hahn *et al.*[9]

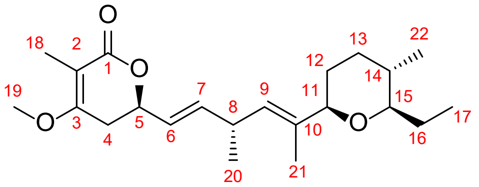

2

Pos.	isolated jerangolid E **2**[8] ^{1}H-NMR (400 MHz, CD_3OD, δ in ppm, J [Hz])	synthetic jerangolid E **2** (Hahn *et al.*[9]) ^{1}H-NMR (500 MHz, CD_3OD, δ in ppm, J [Hz])	synthetic jerangolid E **2** (this work) ^{1}H-NMR (500 MHz, CD_3OD, δ in ppm, J [Hz])
$H4_a$	2.88 (ddq, 17.3, 4.1, 0.8)	2.84 (ddq, 17.6, 4.3, 1.1)	2.84 (ddq, 17.4, 4.1, 1.0)
$H4_b$	2.67 (ddq, 17.3, 11.3, 2.0)	2.67 (ddq, 17.4, 11.5, 2.0)	2.62 (ddq, 17.4, 11.5, 2.0)
H5	4.83 (brdd, 6.7, 4.4)	4.80 (ddd, 11.2, 6.6, 4.4)	4.83 (brddd,11.1, 6.5, 4.4)
H6	5.63 (ddd, 15.6, 6.6, 1.4)	5.59 (ddd, 15.5, 6.6, 1.4)	5.59 (ddd, 15.6, 6.5, 1.4)
H7	5.87 (ddd, 15.6, 6.2, 0.8)	5.83 (ddd, 15.6, 6.4, 1.0)	5.83 (ddd, 15.5, 6.4, 1.0)
H8	3.21 (m)	3.17 (m)	3.23-3.13 (m)
H9	5.29 (ddq, 9.1, 1.3, 1.2)	5.25 (ddq, 9.1, 1.1, 1.2)	5.25 (dquint, 9.0, 1.2)
H11	3.67 (brd, 10.8)	3.63 (brd, 10.8)	3.63 (brd, 11.0)
$H12_a$	1.68 (m)	1.64 (m)	1.68-1.61 (m)
$H12_b$	1.48 (m)	1.44 (m)	1.48-1.36 (m)
$H13_a$	1.86 (m)	1.82 (dddd, 12.5, 3.5, 3.4, 3.1)	1.84-1.78 (m)
$H13_b$	1.30 (m)	1.26 (m)	1.35-1.22 (m)
H14	1.35 (m)	1.31 (m)	1.35-1.22 (m)
H15	2.96 (ddd, 9.2, 7.8, 2.8)	2.92 (ddd, 9.1, 8.0, 2.7)	2.91 (ddd, 9.4, 8.0, 2.7)
$H16_a$	1.75 (m)	1.71 (m)	1.75-1.68
$H16_b$	1.44 (m)	1.40 (m)	1.48-1.36
H17	0.99 (dd, 7.5, 7.3)	0.95 (tr, 7.4)	0.95 (tr, 7.4)
H18	1.74 (dd, 1.7, 1.0)	1.70 (dd, 1.7, 1.2)	1.71 (dd, 1.7, 0.9)
H19	3.88 (s)	3.84 (s)	3.83 (s)
H20	1.13 (d, 6.9)	1.09 (d, 6.9)	1.09 (d, 6.9)
H21	1.69 (d, 1.3)	1.65 (d, 1.4)	1.65 (d, 1.3)
H22	0.87 (d, 6.4)	0.83 (d, 6.4)	0.83 (d, 6.5)

Table S6. Comparison of the ^{13}C-NMR spectra of synthetic jerangolid E **2**, the natural product isolated by Höfle *et al.*,[7,8] and the compound synthesized by Hahn *et al.*[9]

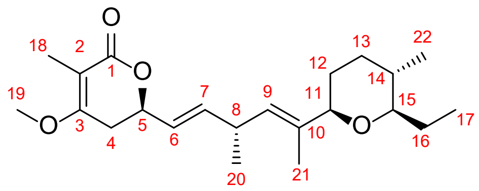

2

Pos.	isolated jerangolid E **2**[8] ^{13}C-NMR (100 MHz, CD_3OD, δ in ppm)	synthetic jerangolid E **2** (Hahn *et al.*[9]) ^{13}C-NMR (125 MHz, CD_3OD, δ in ppm)	synthetic jerangolid E **2** (this work) ^{13}C-NMR (125 MHz, CD_3OD, δ in ppm)	Δδ
1	171.4	171.4	171.4	0.0
2	102.7	102.6	102.7	0.0
3	169.3	169.4	169.4	+0.1
4	30.3	30.3	30.3	0.0
5	76.9	77.0	76.9	0.0
6	126.4	126.4	126.4	0.0
7	140.3	140.3	140.3	0.0
8	35.8	35.8	35.8	0.0
9	128.9	128.9	128.9	0.0
10	137.8	137.8	137.8	0.0
11	83.5	83.5	83.5	0.0
12	32.0	32.0	32.0	0.0
13	34.3	34.2	34.2	-0.1
14	35.6	35.6	35.6	0.0
15	85.9	85.9	85.9	0.0
16	26.9	26.9	26.9	0.0
17	9.9	9.9	9.9	0.0
18	8.8	8.8	8.8	0.0
19	56.3	56.3	56.3	0.0
20	21.0	21.0	21.0	0.0
21	13.4	13.4	13.4	0.0
22	18.0	18.0	18.0	0.0

(R)-6-((R,1E,4E)-5-((2R,5S,6R)-6-Ethyl-5-methyltetrahydro-2H-pyran-2-yl)-3-methylhexa-1,4-dien-1-yl)-4-methoxy-3-(((4-methoxybenzyl)oxy)methyl)-5,6-dihydro-2H-pyran-2-one 58

Synthesis is performed according to the general procedure using lactone **25** (88.9 mg, 200 µmol, 1.0 eq.), stannane **53** (101 mg, 210 µmol, 1.05 eq.), finely ground molecular sieves 4 Å (40 mg) and Pd_2dba_3 (18.2 mg, 20 µmol, 10 mol%) in a 2:1 mixture of dry THF and dry DMF (3 mL). Microwave irradiation is carried out at 40 W for 10 cycles of 1 min each under air cooling (T_{max} = 48-51 °C). After filtration and removal of the solvent in vacuo the crude product is purified via flash chromatography (pentane/acetone 8:1 v:v with 1 vol% NEt_3) affording the title compound **58** (31.4 mg, 63.0 µmol, 32%) as a yellow resin.

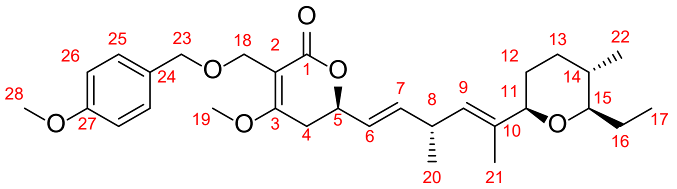

$C_{30}H_{42}O_6$
M = 498.66 g•mol^{-1}

TLC: R_f (pentane/acetone 5/1) = 0.14.

^{1}H-NMR (500 MHz, CD_3OD, δ in ppm): 7.26 (m, 2H, H25), 6.88 (m, 2H, H26), 5.83 (ddd, J = 15.6 Hz, 6.4 Hz, 0.9 Hz, 1H, H7), 5.58 (ddd, J = 15.6 Hz, 6.7 Hz, 1.3 Hz, 1H, H6), 5.26 (dquint, J = 9.0 Hz, 1.2 Hz, 1H, H9), 4.75 (brddd, J = 11.1 Hz, 6.8 Hz, 4.2 Hz, 1H, H5), 4.43 (s, 2H, H23), 4.27 (s, 2H, H18), 3.88 (s, 3H, H19), 3.78 (s, 3H, H28), 3.63 (brd, J = 11.0 Hz, 1H, H11), 3.23-3.14 (m, 1H, H8), 2.88 (ddd, J = 10.5 Hz, 8.1 Hz, 2.7 Hz, 1H, H15), 2.68 (dd, J = 17.5 Hz, 4.1 Hz, 1H, $H4_a$), 2.54 (dd, J = 17.5 Hz, 11.4 Hz, 1H, $H4_b$), 1.84-1.76 (m, 1H, $H13_a$), 1.75-1.69 (m, 1H, $H16_a$), 1.68-1.61 (m, 1H, $H12_a$), 1.65 (d, J = 1.3 Hz, 3H, H21), 1.48-1.37 (m, 2H, $H12_b$+$H16_b$), 1.36-1.22 (m, 2H, $H13_b$+H14), 1.09 (d, J = 6.9 Hz, 3H, H20), 0.95 (tr, J = 7.4 Hz, 3H, H17), 0.84 (d, J = 6.5 Hz, 3H, H22).

^{13}C-NMR (125 MHz, CD_3OD, δ in ppm): 173.8 (C3), 170.2 (C1), 160.8 (C27), 140.6 (C7), 137.8 (C10), 131.9 (C24), 130.6 (C25), 128.8 (C9), 126.1 (C6), 114.6 (C26), 104.7 (C2), 85.9 (C15), 83.4 (C11), 76.7 (C5), 73.0 (C23), 61.8 (C18), 56.9 (C19), 55.7 (C28), 35.8 (C8), 35.6 (C14), 34.2 (C13), 31.9 (C12), 30.5 (C4), 26.9 (C16), 21.0 (C20), 18.0 (C22), 13.4 (C21), 9.9 (C17).

HRMS: calculated for $C_{30}H_{43}O_6$ $[M+H]^+$: 499.30542, measured for $C_{30}H_{43}O_6$ $[M+H]^+$: 499.30437.

Jerangolid H 3

PMB ether **58** (29.8 mg, 59.8 µmol, 1.0 eq.) is dissolved in DCM (3 mL) and treated with H_2O (150 µL). Afterwards DDQ (16.3 mg, 71.8 µmol, 1.2 eq.) is added in one portion at 0 °C and the resulting dark green biphasic mixture is stirred vigorously turning into a red-brown suspension after approximately 3 min. The reaction is stirred for 2 h at 0 °C, then quenched by addition of saturated $NaHCO_3$ solution. The phases are separated and the aqueous phase is extracted three times with ethyl acetate. The combined organic phases are dried with $MgSO_4$, filtered through a pad of celite and the solvent is removed under reduced pressure. Purification via flash chromatography (pentane:acetone 3:1 v:v) affords jerangolid H **3** (22.1 mg, 58.4 µmol, 98%) as a pale yellow resin.

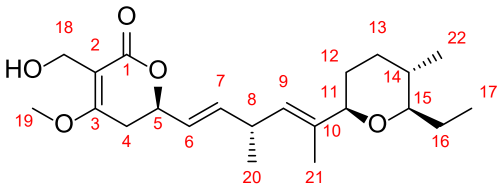

$C_{22}H_{34}O_5$
M = 378.51 g•mol^{-1}

TLC: R_f (pentane/acetone 3/1) = 0.22.

^{1}H-NMR (500 MHz, CD_3OD, δ in ppm): 5.85 (ddd, J = 15.5 Hz, 6.5 Hz, 1.1 Hz, 1H, H7), 5.60 (ddd, J = 15.5 Hz, 6.7 Hz, 1.4 Hz, 1H, H6), 5.26 (dquint, J = 9.0 Hz, 1.3 Hz, 1H, H9), 4.84 (brddd, J = 11.1 Hz, 6.7 Hz, 4.0 Hz, 1H, H5), 4.31 (d, J = 11.5 Hz, 1H, H18$_a$), 4.29 (d, J = 11.5 Hz, 1H), 3.89 (s, 3H, H19), 3.63 (brd, J = 11.1 Hz, 1H, H11), 3.22-3.14 (m, 1H, H8), 2.92 (ddd, J = 9.4 Hz, 8.0 Hz, 2.8 Hz, 1H, H15), 2.91 (dd, J = 17.6 Hz, 4.1 Hz, 1H, H4$_a$), 2.70 (dd, J = 17.6 Hz, 11.5 Hz, 1H, H4$_b$), 1.84-1.79 (m, 1H, H13$_a$), 1.76-1.69 (dqd, J = 14.2 Hz, 7.5 Hz, 2.8 Hz, 1H, H16$_a$), 1.66 (d, J = 1.4 Hz, 1H, H21), 1.66-1.62 (m, 1H, H12$_a$), 1.49-1.38 (m, 2H, H12$_b$+H16$_b$), 1.37-1.30 (m, 1H, H14), 1.29-1.21 (m, 1H, H13$_b$), 1.10 (d, J = 6.8 Hz, 3H, H20), 0.95 (tr, J = 7.4 Hz, 3H, H17), 0.84 (d, J = 6.4 Hz, 3H, H22).

^{13}C-NMR (125 MHz, CD_3OD, δ in ppm): 172.7 (C3), 170.3 (C1), 140.5 (C7), 137.8 (C10), 128.8 (C9), 126.2 (C6), 107.3 (C2), 85.9 (C15), 83.5 (C11), 76.9 (C5), 56.8 (C19), 54.0 (C18), 35.8 (C8), 35.6 (C14), 34.2 (C13), 31.9 (C12), 30.5 (C4), 26.9 (C16), 21.0 (C20), 18.0 (C22), 13.4 (C21), 9.9 (C17).

HRMS: calculated for $C_{22}H_{35}O_5$ $[M+H]^+$: 379.24791, measured for $C_{22}H_{35}O_5$ $[M+H]^+$: 379.24737.

Table S7. Comparison of the ^{1}H-NMR spectra of synthetic jerangolid H **3** and natural jerangolid H 3 isolated by Höfle *et al.*[7,8]

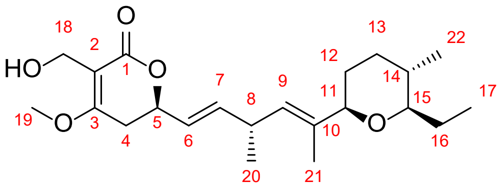

3

Pos.	isolated jerangolid H **3**[8] ^{1}H-NMR (400 MHz, CD_3OD, δ in ppm, J [Hz])	jerangolid H **3** (this work) ^{1}H-NMR (500 MHz, CD_3OD, δ in ppm, J [Hz])
4_a	2.95 (dd, 17.5, 4.2)	2.91 (dd, 17.6, 4.1)
4_b	2.73 (dd, 17.5, 11.3)	2.70 (dd, 17.6, 11.5)
5	4.88 (m)	4.84 (brddd,11.1, 6.7, 4.0)
6	5.64 (ddd, 15.5, 6.7, 1.4)	5.60 (ddd, 15.5, 6.7, 1.4)
7	5.89 (ddd, 15.5, 6.4, 1.0)	5.85 (ddd, 15.5, 6.5, 1.1)
8	3.22 (m)	3.22-3.14 (m)
9	5.30 (ddq, 9.0, 1.4, 1.3)	5.26 (dquint, 9.0, 1.3)
11	3.68 (brd, 10.1)	3.63 (brd, 11.1)
12_a	1.68 (m)	1.66-1.62 (m)
12_b	1.49 (m)	1.49-1.38 (m)
13_a	1.85 (m)	1.84-1.79 (m)
13_b	1.28 (m)	1.29-1.21 (m)
14	1.34 (m)	1.37-1.30 (m)
15	2.96 (m)	2.92 (ddd, 9.4, 8.0, 2.8)
16_a	1.76 (dq, 7.4, 2.9)	1.72 (dqd, 14.2, 7.5, 2.8)
16_b	1.44 (m)	1.49-1.38
17	0.99 (dd, 7.4, 7.3)	0.95 (tr, 7.4)
18_a	4.34 (s)	4.31 (d, 11.5)
18_b	4.34 (s)	4.29 (d, 11.5)
19	3.93 (s)	3.89 (s)
20	1.13 (d, 6.8)	1.10 (d, 6.8)
21	1.70 (d, 1.4)	1.66 (d, 1.4)
22	0.87 (d, 6.2)	0.84 (d, 6.4)
OH	2.87 (s)[a)]	-

[a)]measured in DMSO-d_6

Table S8. Comparison of the ^{13}C-NMR spectra of synthetic jerangolid H **3** and natural jerangolid H 3 isolated by Höfle *et al.*[7,8]

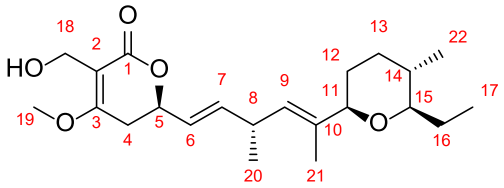

3

Pos.	isolated jerangolid H **3**[8] ^{13}C-NMR (100 MHz, CD_3OD, δ in ppm)	synthetic jerangolid H **3** (this work) ^{13}C-NMR (100 MHz, CD_3OD, δ in ppm)	Δδ
1	170.3	170.3	0.0
2	107.3	107.3	0.0
3	172.6	172.7	+0.1
4	30.5	30.5	0.0
5	76.9	76.9	0.0
6	126.2	126.2	0.0
7	140.5	140.5	0.0
8	35.8a)	35.8	0.0
9	128.9	128.8	-0.1
10	137.9	137.8	-0.1
11	83.5	83.5	0.0
12	32.0	31.9	-0.1
13	34.3	34.2	-0.1
14	35.6a)	35.6	0.0
15	85.9	85.9	0.0
16	26.9	26.9	0.0
17	9.9	9.9	0.0
18	54.0	54.0	0.0
19	56.8	56.8	0.0
20	21.0	21.0	0.0
21	13.4	13.4	0.0
22	18.0	18.0	0.0

a)assignment ambiguous.

Jerangolid D 4

Synthesis is performed according to the general procedure using lactone **54** (81 mg, 263 µmol, 1.0 eq.), stannane **38** (80 w%, 157 mg, 275 µmol, 1.05 eq.), finely ground molecular sieves 4 Å (80 mg) and Pd_2dba_3 (24.0 mg, 26 µmol, 10 mol%) in a 2:1 mixture of dry THF and dry DMF (3 mL). Microwave irradiation is carried out at 40 W for 7 cycles of 1 min each under air cooling (T_{max} = 44-46 °C). After filtration and removal of the solvent in vacuo the crude product is purified via flash chromatography (pentane/acetone 20:1 v:v with 1 vol% NEt_3) affording jerangolid D **4** (28.5 mg, 79.1 µmol, 30%) as a yellow resin.

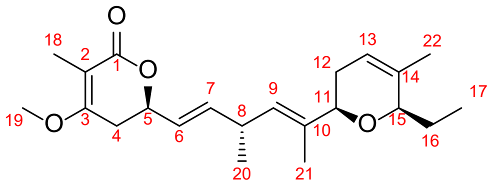

$C_{22}H_{32}O_4$
M = 360.49 g•mol^{-1}

TLC: R_f (pentane/acetone 10/1) = 0.20.

^{1}H-NMR (500 MHz, CD_3OD, δ in ppm): 5.83 (ddd, J = 15.5 Hz, 6.5 Hz, 1.1 Hz, 1H, H7), 5.59 (ddd, J = 15.5 Hz, 6.6 Hz, 1.4 Hz, 1H, H6), 5.60-5.55 (m, 1H, H13), 5.29 (dquint, J = 9.1 Hz, 1.3 Hz, 1H, H9), 4.81 (brddd, J = 11.5 Hz, 6.6 Hz, 4.1 Hz, 1H, H5), 3.84 (dd, J = 10.2 Hz, 3.0 Hz, 1H, H11), 3.84 (s, 3H, H19), 3.23-3.14 (m, 1H, H8), 2.85 (ddq, J = 17.4 Hz, 4.2 Hz, 1.0 Hz, 1H, $H4_a$), 2.63 (ddq, J = 17.4 Hz, 11.5 Hz, 1.9 Hz, 1H, $H4_b$), 2.14-2.06 (m, 1H, $H12_a$), 1.94 (m, 1H, $H12_b$), 1.77 (dqd, J = 14.2 Hz, 7.4 Hz, 3.6 Hz, 1H, $H16_a$), 1.71 (dd, J = 1.9 Hz, 1.0 Hz, 3H, H18), 1.67 (d, J = 1.3 Hz, 3H, H21), 1.61-1.59 (m, 3H, H22), 1.54 (dqd, J = 14.4 Hz, 7.3 Hz, 6.2 Hz, $H16_b$), 1.10 (d, J = 6.8 Hz, 3H, H20), 0.90 (tr, J = 7.4 Hz, 3H, H17).

^{13}C-NMR (125 MHz, CD_3OD, δ in ppm): 171.4 (C1), 169.4 (C3), 140.2 (C7), 137.2 (C10), 136.3 (C14), 129.4 (C9), 126.4 (C6), 122.0 (C13), 102.6 (C2), 79.5 (C15), 79.3 (C11), 76.9 (C5), 56.4 (C19), 35.8 (C8), 31.1 (C12), 30.3 (C4), 26.6 (C16), 21.0 (C20), 19.1 (C22), 12.8 (C21), 8.8 (C18), 8.7 (C17)

HRMS: calculated for $C_{22}H_{33}O_4$ $[M+H]^+$: 361.23734, measured for $C_{22}H_{33}O_4$ $[M+H]^+$: 361.23727.

Table S9. Comparison of the ^{1}H-NMR spectra of synthetic jerangolid D **4**, the natural product isolated by Höfle *et al.*,[7,8] and the compound synthesized by Markó *et al.*[6]

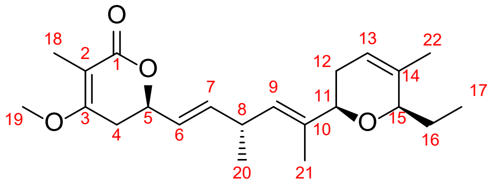

4

Pos.	isolated jerangolid D **4**[8] ^{1}H-NMR (400 MHz, CD_3OD, δ in ppm, J [Hz])	synthetic jerangolid D **4** (Markó *et al.*[6]) ^{1}H-NMR (500 MHz, CD_3OD, δ in ppm, J [Hz])	synthetic jerangolid D **4** (this work) ^{1}H-NMR (500 MHz, CD_3OD, δ in ppm, J [Hz])
$H4_a$	2.88 (ddd, 17.3, 4.0, 0.7)	2.86 (ddq, 17.4, 4.0, 0.8)	2.85 (ddq, 17.4, 4.2, 1.0)
$H4_b$	2.67 (ddq, 17.4, 11.4, 2.0)	2.66 (ddq, 17.4, 2.1)	2.63 (ddq, 17.4, 11.5, 1.9)
H5	4.85 (m)	4.85-4.81 (m)	4.81 (brddd, 11.5, 6.6, 4.1)
H6	5.63 (ddd, 15.3, 6.6, 1.3)	5.62 (dd, 15.4, 1.4)	5.59 (ddd, 15.5, 6.6, 1.4)
H7	5.87 (ddd, 15.3, 6.5, 0.8)	5.85 (dd, 15.3, 6.5)	5.83 (ddd, 15.5, 6.5, 1.1)
H8	3.23 (m)	3.23-3.19 (m)	3.23-3.14 (m)
H9	5.33 (ddq, 9.1, 1.2, 1.1)	5.31 (dq, 9.2, 1.1)	5.29 (dquint, 9.1, 1.3)
H11	3.88 (dd, 10.3, 3.2)	3.87 (dd, 10.4, 2.8)	3.84 (dd, 10.2, 3.0)
$H12_a$	2.14 (m)	2.18-2.09 (m)	2.14-2.06 (m)
$H12_b$	1.95 (m)	1.97-1.91 (m)	1.94-1.86 (m)
H13	5.63 (m)	5.64-5.58 (m)	5.60-5.55 (m)
H15	4.13 (brs)	4.11 (brs)	4.11-4.07 (m)
$H16_a$	1.81 (m)	1.81-1.77 (m)	1.77 (dqd, 14.2, 7.4, 3.6)
$H16_b$	1.58 (m)	1.62-1.52 (m)	1.54 (dqd, 14.4, 7.3, 6.2)
H17	0.94 (dd, 7.4, 7.3)	0.93 (tr, 7.3)	0.90 (tr, 7.4)
H18	1.75 (-)[a)]	1.73 (brs)	1.71 (dd, 1.9, 1.0)
H19	3.88 (s)	3.87 (s)	3.84 (s)
H20	1.14 (d, 6.8)	1.12 (d, 6.8)	1.10 (d, 6.8)
H21	1.71 (d, 1.1)	1.69 (brd, 1.2)	1.67 (d, 1.3)
H22	1.64 (m)	1.62 (m)	1.61-1.59 (m)

[a)]multiplet not specified

Table S10. Comparison of the ^{13}C-NMR spectra of synthetic jerangolid D **4**, the natural product isolated by Höfle *et al.*,[7,8] and the compound synthesized by Markó *et al.*[6]

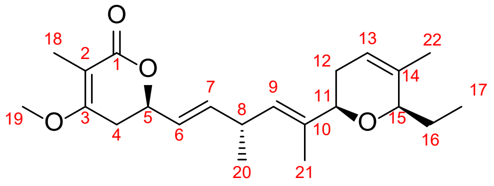

4

Pos.	isolated jerangolid D **4**[8] ^{13}C-NMR (100 MHz, CD_3OD, δ in ppm)	synthetic jerangolid D **4** (Markó *et al.*[6]) ^{13}C-NMR (125 MHz, CD_3OD, δ in ppm)	synthetic jerangolid D **4** (this work) ^{13}C-NMR (125 MHz, CD_3OD, δ in ppm)	Δδ
1	171.4	171.4	171.4	0.0
2	102.7	102.7	102.6	-0.1
3	169.3	169.4	169.4	+0.1
4	30.3	30.3	30.3	0.0
5	76.9	76.9	76.9	0.0
6	126.4	126.4	126.4	0.0
7	140.2	140.2	140.2	0.0
8	35.8	35.8	35.8	0.0
9	129.4	129.4	129.4	0.0
10	137.3	137.3	137.2	-0.1
11	79.2	79.3	79.3	+0.1
12	31.2	31.1	31.1	-0.1
13	122.0	122.0	122.0	0.0
14	136.3	136.3	136.3	0.0
15	79.5	79.5	79.5	0.0
16	26.6	26.6	26.6	0.0
17	8.7a)	8.7	8.7b)	0.0
18	8.8a)	8.8	8.8b)	0.0
19	56.3	56.3	56.4	+0.1
20	21.0	21.0	21.0	0.0
21	12.8	12.8	12.8	0.0
22	19.0	19.0	19.1	+0.1

a)assignment ambiguous; b)assigned based on ^{1}J-coupling observed in the HMBC spectrum.

(R)-6-((R,1E,4E)-5-((2R,6R)-6-Ethyl-5-methyl-3,6-dihydro-2H-pyran-2-yl)-3-methylhexa-1,4-dien-1-yl)-4-methoxy-3-(((4-methoxybenzyl)oxy)methyl)-5,6-dihydro-2H-pyran-2-one 59

Synthesis is performed according to the general procedure using lactone **25** (133 mg, 300 µmol, 1.0 eq.), stannane **38** (143 mg, 315 µmol, 1.05 eq.), finely ground molecular sieves 4 Å (80 mg) and Pd_2dba_3 (28.0 mg, 30 µmol, 10 mol%) in a 2:1 mixture of dry THF and dry DMF (3 mL). Microwave irradiation is carried out at 40 W for 10 cycles of 1 min each under air cooling (T_{max} = 46-48 °C). After filtration and removal of the solvent in vacuo the crude product is purified via flash chromatography (pentane/acetone 10:1 v:v with 1 vol% NEt_3) affording the title compound **59** (51.1 mg, 103 µmol, 34%) as a yellow resin.

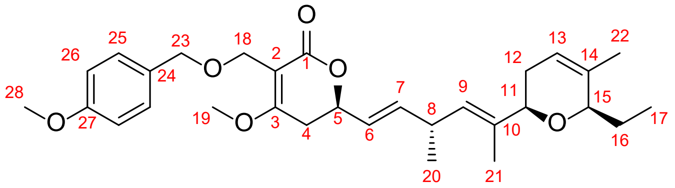

$C_{30}H_{40}O_6$
M = 496.64 g•mol^{-1}

TLC: R_f (pentane/acetone 5/1) = 0.19.

^{1}H-NMR (400 MHz, $CDCl_3$, δ in ppm): 7.30-7.26 (m, 2H, H25), 6.88-6.83 (m, 2H, H26), 5.78 (ddd, J = 15.6 Hz, 6.2 Hz, 0.9 Hz, 1H, H7), 5.57 (dq, J = 6.2 Hz, 1.7 Hz, 1H, H13), 5.51 (ddd, J = 15.5 Hz, 6.8 Hz, 1.4 Hz, 1H, H6), 5.25 (dquint, J = 9.0 Hz, 1.3 Hz, 1H, H9), 4.76-4.69 (m, 1H, H5), 4.49 (s, 2H, H23), 4.31 (d, J = 10.9 Hz, 1H, $H18_a$), 4.28 (d, J = 10.9 Hz, 1H, $H18_b$), 4.12-4.06 (m, 1H, H15), 3.83 (brdd, J = 10.7 Hz, 2.9 Hz, 1H, H11), 3.82 (s, 3H, H19), 3.79 (s, 3H, H28), 3.19-3.08 (m, 1H, H8), 2.62 (dd, J = 17.1 Hz, 5.0 Hz, 1H, $H4_a$), 2.56 (dd, J = 17.1 Hz, 10.8 Hz, 1H, $H4_b$), 2.15-2.04 (m, 1H, $H12_a$), 1.94-1.84 (m, 1H, $H12_b$), 1.78 (dqd, J = 14.6 Hz, 7.3 Hz, 3.6 Hz, 1H, $H16_a$), 1.65 (d, J = 1.4 Hz, 3H, H21), 1.59 (dd, J = 2.2 Hz, 1.0 Hz, 3H, H22), 1.53 (dqd, J = 14.5 Hz, 7.3 Hz, 6.1 Hz, 1H, $H16_b$), 1.08 (d, J = 6.9 Hz, 3H, H20), 0.90 (tr, J = 7.3 Hz, 3H, H17).

^{13}C-NMR (100 MHz, $CDCl_3$, δ in ppm): 169.7 (C3), 167.4 (C1), 159.2 (C27), 139.8 (C7), 136.5 (C10), 135.3 (C14), 131.1 (C24), 129.6 (C25), 127.8 (C9), 124.6 (C6), 120.9 (C13), 113.8 (C26), 105.6 (C2), 78.1 (C15), 77.8 (C11), 75.0 (C5), 72.4 (C23), 61.0 (C18), 56.1 (C19), 55.4 (C28), 34.6 (C8), 30.33 (C12), 30.26 (C4), 25.8 (C16), 20.6 (C20), 19.1 (C22), 12.7 (C21), 8.5 (C17).

HRMS: calculated for $C_{30}H_{41}O_6$ $[M+H]^+$: 497.28977, measured for $C_{30}H_{41}O_6$ $[M+H]^+$: 497.29156.

Jerangolid A 5

PMB ether **59** (24.2 mg, 48.7 µmol, 1.0 eq.) is dissolved in DCM (2 mL) and treated with H_2O (100 µL). Afterwards DDQ (13.3 mg, 59.0 µmol, 1.2 eq.) is added in one portion at 0 °C and the resulting dark green biphasic mixture is stirred vigorously turning into a red-brown suspension after approximately 3 min. The reaction is stirred for 2 h at 0 °C, then quenched by addition of saturated $NaHCO_3$ solution. The phases are separated and the aqueous phase is extracted three times with ethyl acetate. The combined organic phases are dried with $MgSO_4$, filtered through a pad of celite and the solvent is removed under reduced pressure. Purification via flash chromatography (pentane:acetone 2:1 v:v) affords jerangolid A **5** (18.3 mg, 48.6 µmol, quantitative) as a colorless resin.

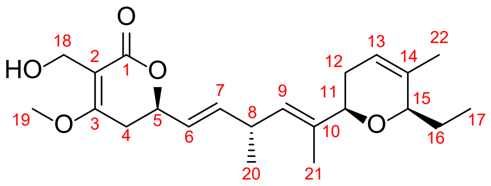

$C_{22}H_{32}O_5$
M = 376.49 g•mol^{-1}

TLC: R_f (pentane/acetone 3/1) = 0.20.

^{1}H-NMR (500 MHz, CD_3OD, δ in ppm): 5.85 (ddd, J = 15.6 Hz, 6.5 Hz, 1.0 Hz, 1H, H7), 5.60 (ddd, J = 15.6 Hz, 6.7 Hz, 1.3 Hz, 1H, H6), 5.60-5.57 (m, 1H, H13), 5.29 (dquint, J = 8.8 Hz, 1.2 Hz, 1H, H9), 4.83 (brddd, J = 11.0 Hz, 6.6 Hz, 4.0 Hz, 1H, H5), 4.31 (d, J = 11.4 Hz, 1H, $H18_a$), 4.29 (d, J = 11.4 Hz, 1H, $H18_b$), 4.11-4.06 (m, 1H, H15), 3.89 (s, 3H, H19), 3.84 (brdd, J = 10.7 Hz, 2.7 Hz, 1H, H11), 3.24-3.15 (m, 1H, H8), 2.91 (dd, J = 17.5 Hz, 4.1 Hz, 1H, $H4_a$), 2.70 (dd, J = 17.5 Hz, 11.5 Hz, 1H, $H4_b$), 2.15-2.06 (m, 1H, $H12_a$), 1.95-1.88 (m, 1H, $H12_b$), 1.77 (dqd, J = 14.6 Hz, 7.4 Hz, 3.6 Hz, 1H, $H16_a$), 1.67 (d, J = 1.4 Hz, 3H, H21), 1.60 (dq, J = 2.4 Hz, 1.2 Hz, 3H, H22), 1.54 (dqd, J = 14.4 Hz, 7.2 Hz, 6.3 Hz, 1H, $H16_b$), 1.10 (d, J = 6.8 Hz, 3H, H20), 0.90 (tr, J = 7.3 Hz, 3H, H17).

^{13}C-NMR (125 MHz, CD_3OD, δ in ppm): 127.7 (C3), 170.3 (C1), 140.5 (C7), 137.3 (C10), 136.3 (C14), 129.3 (C9), 126.2 (C6), 122.0 (C13), 107.3 (C2), 79.5 (C15), 79.3 (C11), 76.9 (C5), 56.8 (C19), 54.0 (C18), 35.9 (C8), 31.2 (C12), 30.5 (C4), 26.6 (C16), 21.0 (C20), 19.1 (C22), 12.8 (C21), 8.7 (C17).

HRMS: calculated for $C_{22}H_{33}O_5$ $[M+H]^+$: 377.23225, measured for $C_{22}H_{33}O_5$ $[M+H]^+$: 377.23400.

Table S11. Comparison of the ^{1}H-NMR spectra of synthetic jerangolid A **5**, the natural product isolated by Höfle *et al.*,[7,8] and the compound synthesized by Hanessian *et al.*[10]

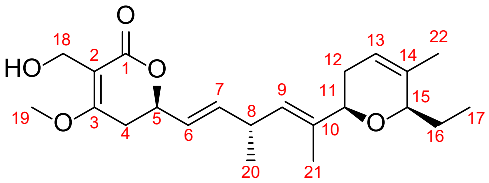

5

Pos.	isolated jerangolid A **5**[8] ^{1}H-NMR (400 MHz, CD_3OD, δ in ppm, J [Hz])	synthetic jerangolid A **5** (Hanessian *et al.*[10]) ^{1}H-NMR (500 MHz, CD_3OD, δ in ppm, J [Hz])	synthetic jerangolid A **5** (this work) ^{1}H-NMR (500 MHz, CD_3OD, δ in ppm, J [Hz])
H4$_a$	2.90 (dd, 17.2, 4.7)a)	2.86 (dd, 17.5, 4.1)	2.91 (dd, 17.5, 4.1)
H4$_b$	2.72 (dd, 17.3, 10.9)a)	2.65 (dd, 17.5, 11.5)	2.70 (dd, 17.5, 11.5)
H5	4.80 (dddd, 10.9, 6.8, 4.7, 1.0)	4.79 (ddd, 11.1, 6.8, 4.0)	4.83 (brddd, 11.0, 6.6, 4.0)
H6	5.53 (ddd, 15.5, 6.8, 1.5)	5.56 (ddd, 15.5, 6.8, 1.4)	5.60 (ddd, 15.6, 6.7, 1.3)
H7	5.81 (ddd, 15.4, 6.2, 1.0)	5.80 (ddd, 15.5, 6.5, 1.0)	5.85 (ddd, 15.6, 6.5, 1.0)
H8	3.14 (dddq, 8.6, 6.9, 6.9, 1.5)	3.17-3.13 (m)	3.24-3.15 (m)
H9	5.26 (ddq, 9.0, 1.4, 1.3)	5.24 (dt, 9.1, 1.3)	5.29 (dquint, 8.8,1.2)
H11	3.84 (dd, 10.9, 2.8)	3.79 (dd, 10.9, 2.8)	3.84 (brdd, 10.7, 2.7)
H12$_a$	2.13 (m)	2.08-2.02 (m)	2.15-2.06 (m)
H12$_b$	1.95 (m)	1.88-1.83 (m)	1.95-1.88 (m)
H13	5.57 (dq, 6.3, 1.5)	5.56-5.53 (m)	5.60-5.57 (m)
H15	4.10 (m)	4.04 (brs)	4.11-4.06 (m)
H16$_a$	1.78 (dqd, 14.4, 7.3, 3.6)	1.74-1.69 (m)	1.77 (dqd, 14.6, 7.4, 3.6)
H16$_b$	1.54 (dqd, 14.3, 7.2, 0.9)	1.52-1.46 (m)	1.54 (dqd, 14.4, 7.2, 6.3)
H17	0.91 (dd, 7.3, 7.2)	0.84 (tr, 7.3)	0.90 (tr, 7.3)
H18$_a$	4.35 (d, 12.4)a)	4.25 (d, 11.3)	4.31 (d, 11.4)
H18$_b$	4.32 (d, 12.4)a)	4.24 (d, 11.4)	4.29 (d, 11.4)
H19	3.84 (s)	3.84 (s)	3.89 (s)
H20	1.11 (d, 6.8)	1.05 (d, 6.8)	1.10 (d, 6.8)
H21	1.66 (d, 1.5)	1.62 (d, 1.4)	1.67 (d, 1.4)
H22	1.60 (dd, 2.4, 1.1)	1.55 (dd, 2.3, 1.1)	1.60 (dq, 2.4, 1.2)
OH	2.80b)	-	-

a)corrected chemical shifts;[10] b) measured in $CDCl_3$

Table S12. Comparison of the ^{13}C-NMR spectra of synthetic jerangolid A **5**, the natural product isolated by Höfle *et al.*,[7,8] and the compound synthesized by Hanessian *et al.*[10]

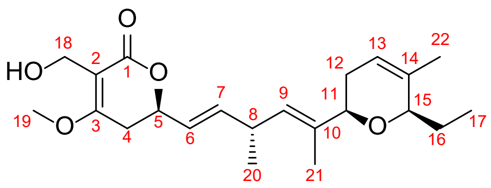

5

Pos.	isolated jerangolid A **5**[8] ^{13}C-NMR (100 MHz, CD_3OD, δ in ppm)	synthetic jerangolid A **5** (Hanessian *et al.*[10]) ^{13}C-NMR (125 MHz, CD_3OD, δ in ppm)	synthetic jerangolid A **5** (this work) ^{13}C-NMR (125 MHz, CD_3OD, δ in ppm)	Δδ
1	170.3	170.4	170.3	0.0
2	107.4	102.2	107.3	-0.1
3	172.6	172.8	172.7	+0.1
4	30.5	30.5	30.5	0.0
5	76.8	76.9	76.9	+0.1
6	126.2	126.2	126.2	0.0
7	140.4	140.5	140.5	+0.1
8	35.8	35.9	35.9	+0.1
9	129.3	129.4	129.3	0.0
10	137.3	137.3	137.3	0.0
11	79.2	79.3	79.3	+0.1
12	31.2	31.1	31.2	0.0
13	122.0	122.0	122.0	0.0
14	136.3	136.3	136.3	0.0
15	79.5	79.5	79.5	0.0
16	26.6	26.6	26.6	0.0
17	8.7	8.7	8.7	0.0
18	54.0	53.9	54.0	0.0
19	56.8	56.8	56.8	0.0
20	21.0	20.9	21.0	0.0
21	12.8	12.8	12.8	0.0
22	19.1	19.1	19.1	0.0

NMR spectra

Methyl (S)-2-((*tert*-butyldimethylsilyl)oxy)propanoate S1

^{1}H-NMR (400 MHz, $CDCl_3$)

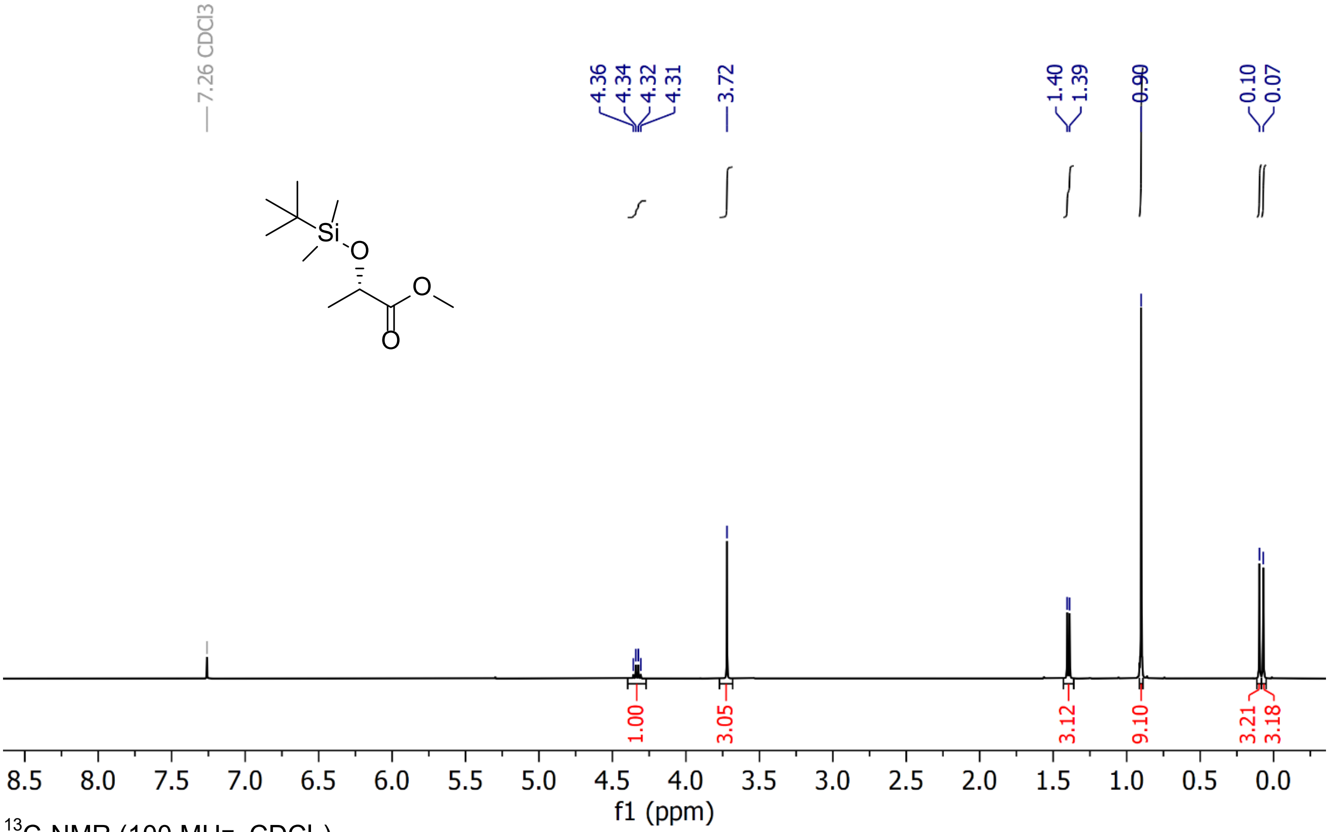

^{13}C-NMR (100 MHz, $CDCl_3$)

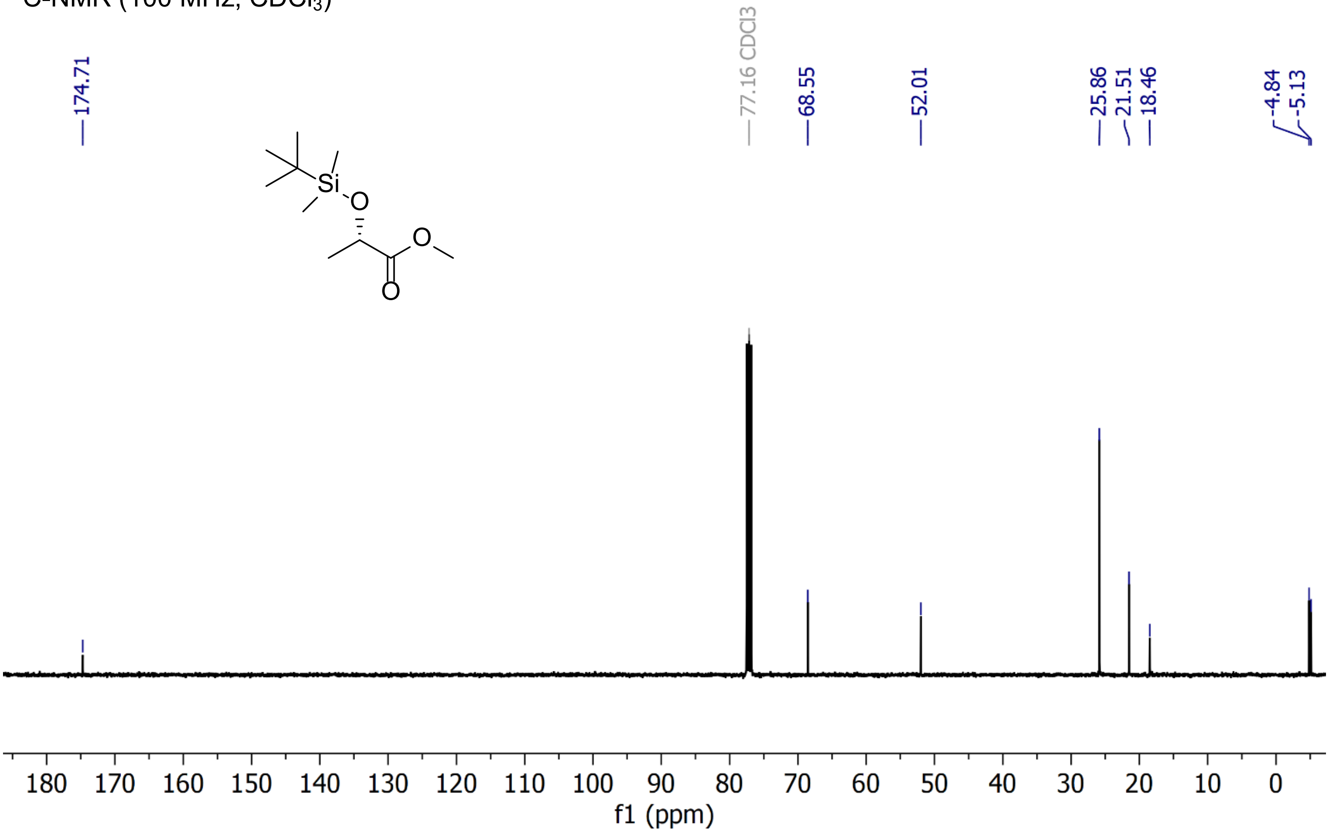

Methyl (S)-4-((*tert*-butyldimethylsilyl)oxy)pent-2-enoate S2

^{1}H-NMR (400 MHz, $CDCl_3$)

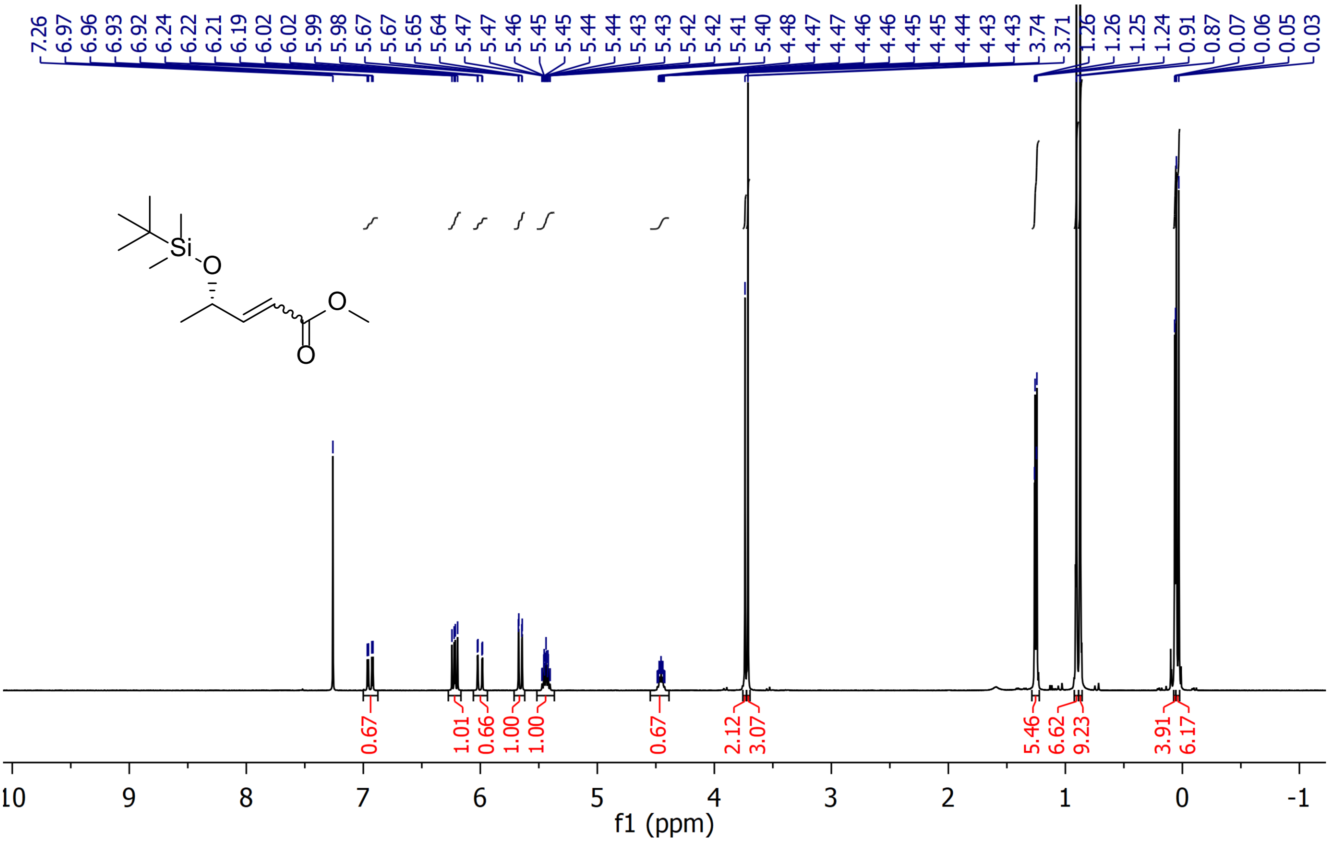

^{13}C-NMR (100 MHz, $CDCl_3$)

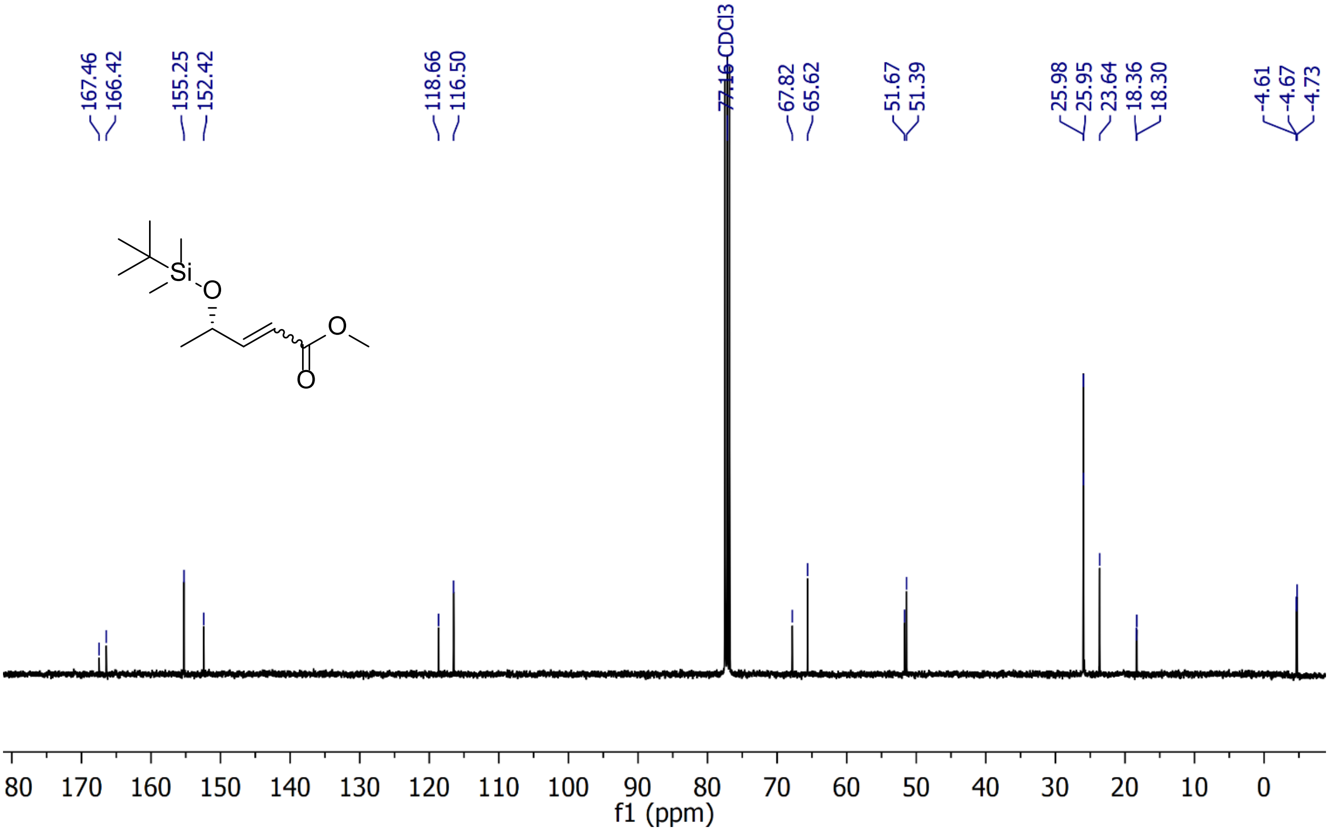

(S)-4-((*tert*-butyldimethylsilyl)oxy)pent-2-en-1-ol S3

^{1}H-NMR (400 MHz, $CDCl_3$)

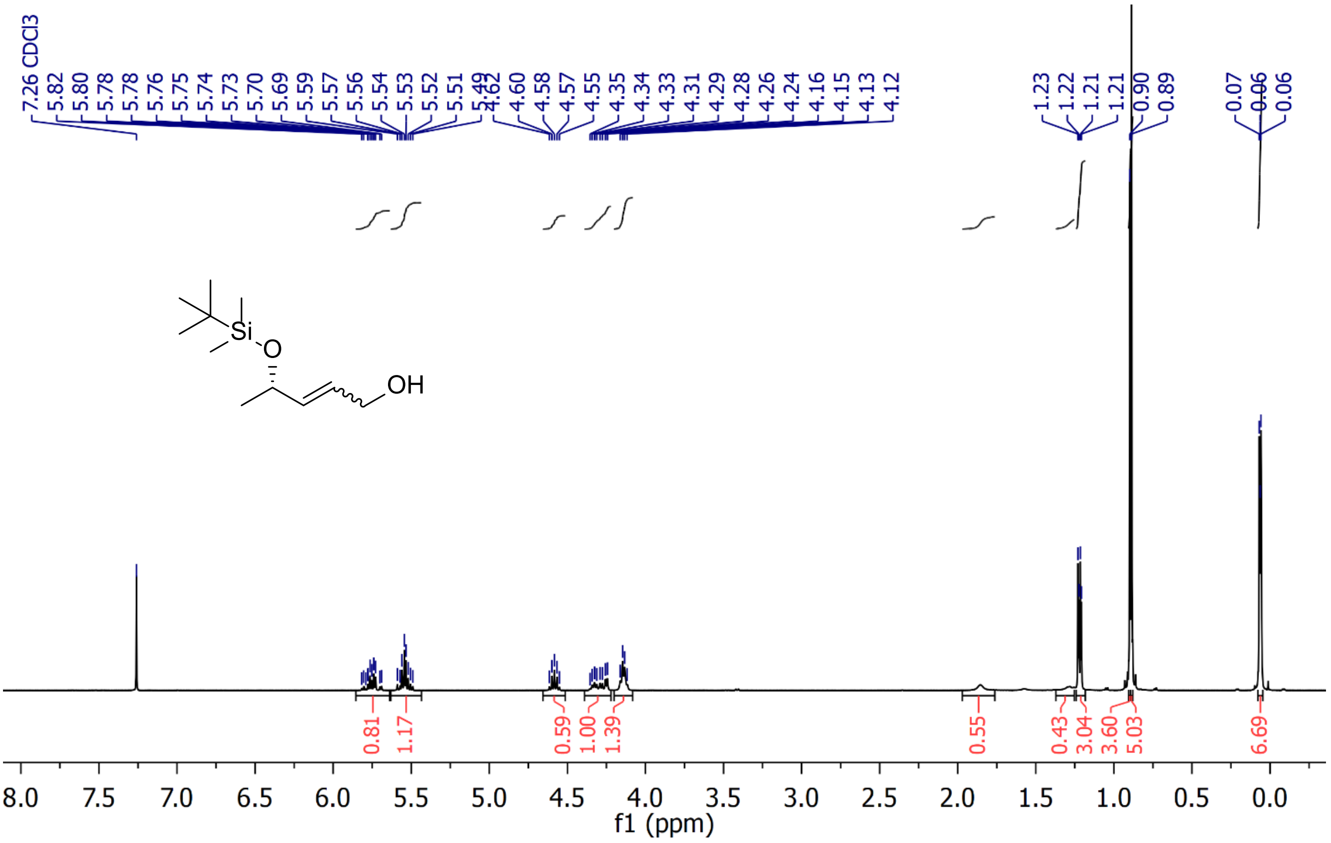

^{13}C-NMR (100 MHz, $CDCl_3$)

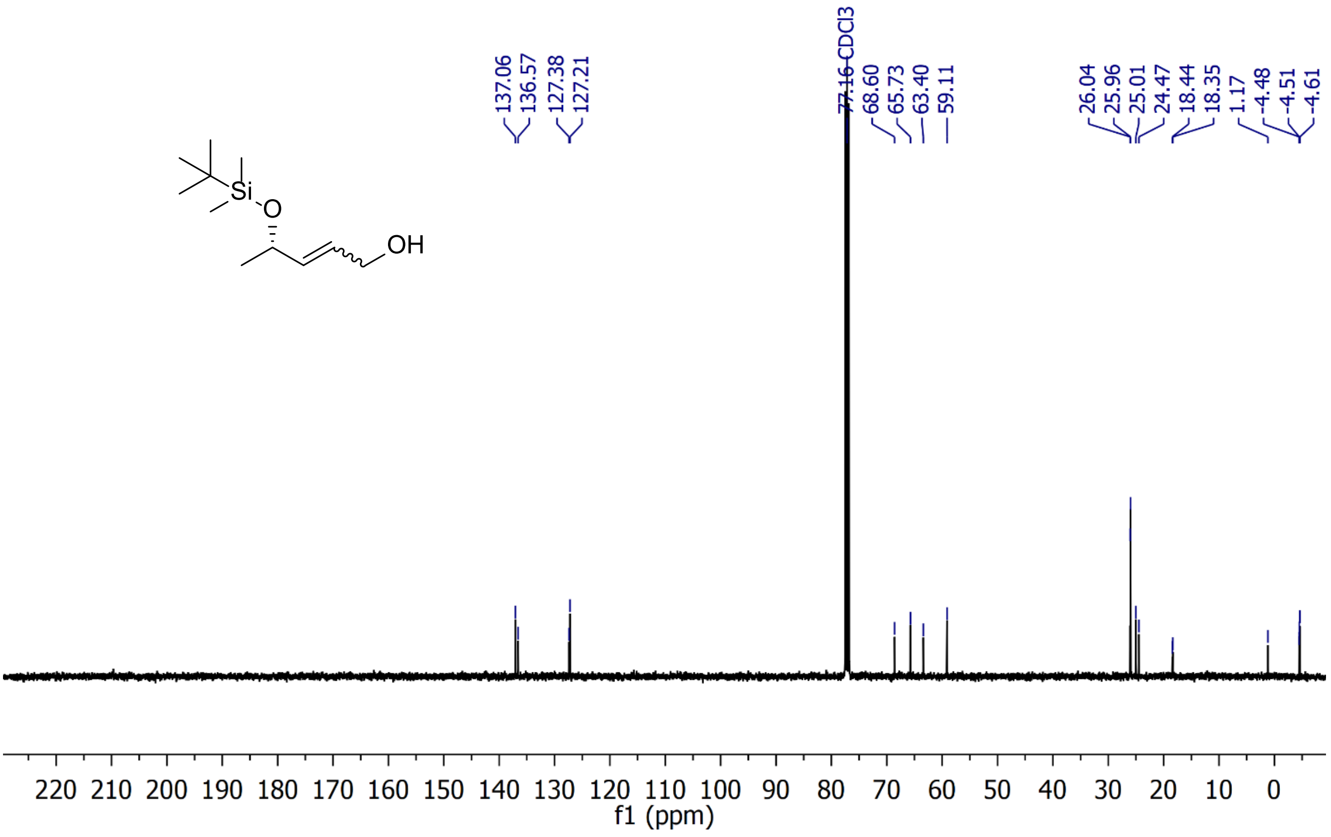

(S,E)-4-((*tert*-butyldimethylsilyl)oxy)pent-2-enal 16

^{1}H-NMR (400 MHz, $CDCl_3$)

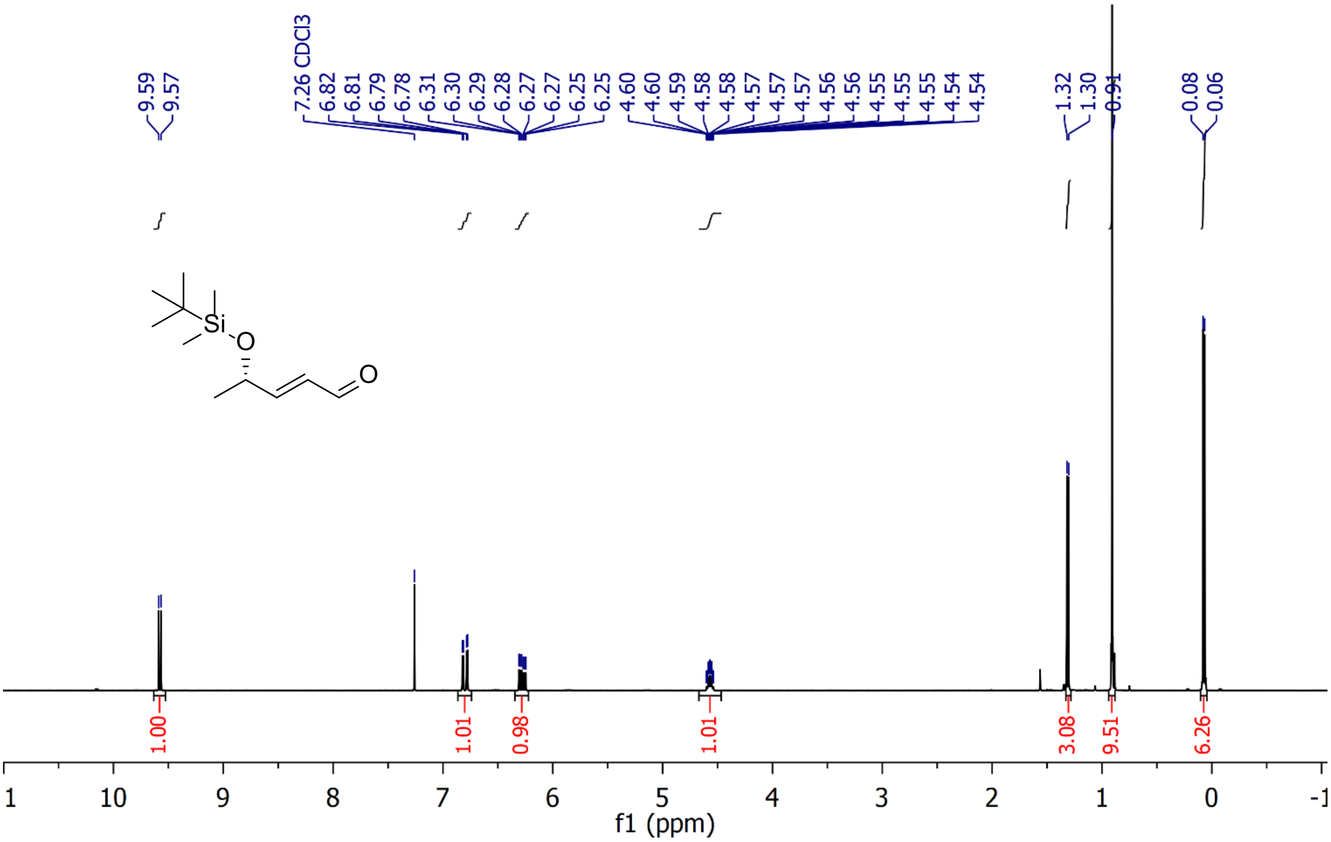

^{13}C-NMR (100 MHz, $CDCl_3$)

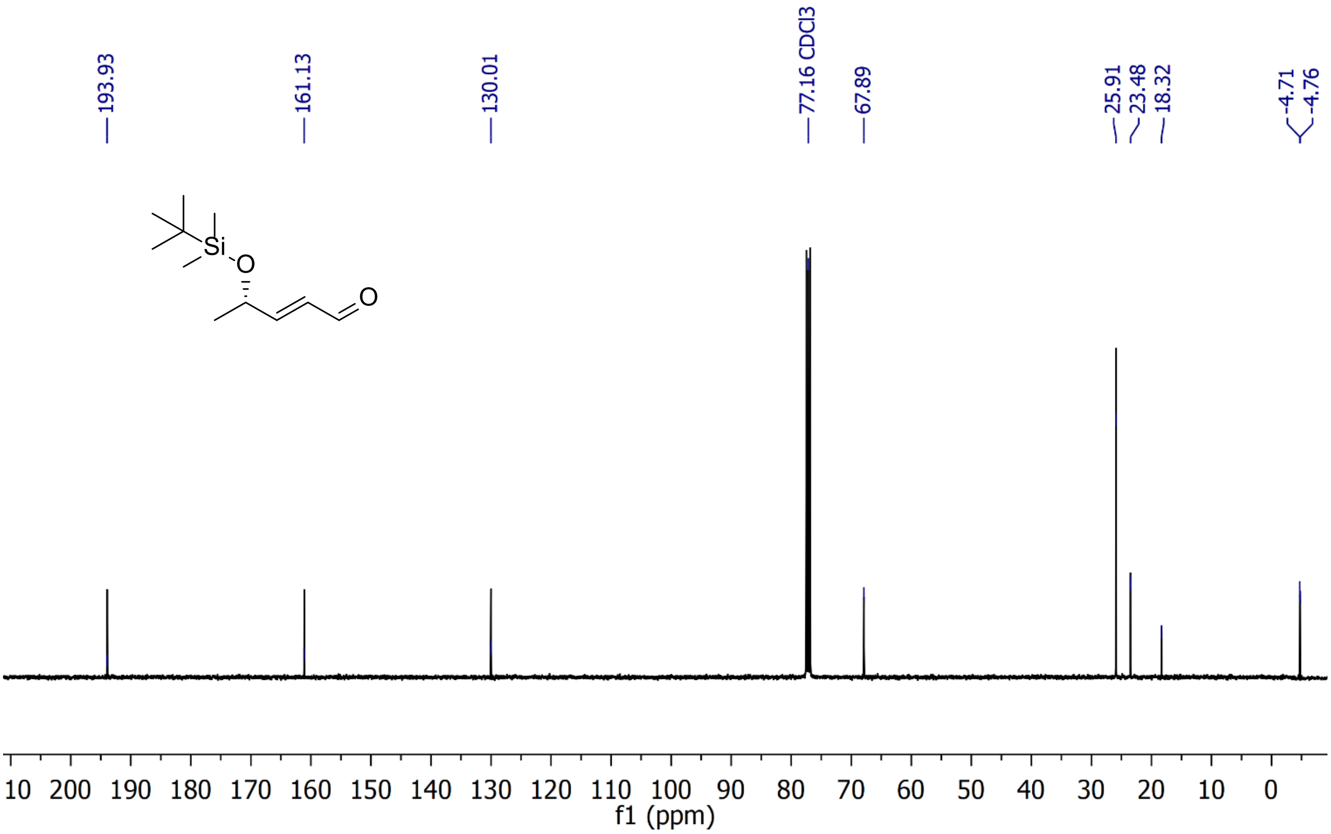

Trimethyl((2,2,5-trimethyl-4-methylen-4H-1,3-dioxin-6-yl)oxy)silane 18

^{1}H-NMR (400 MHz, $CDCl_3$)

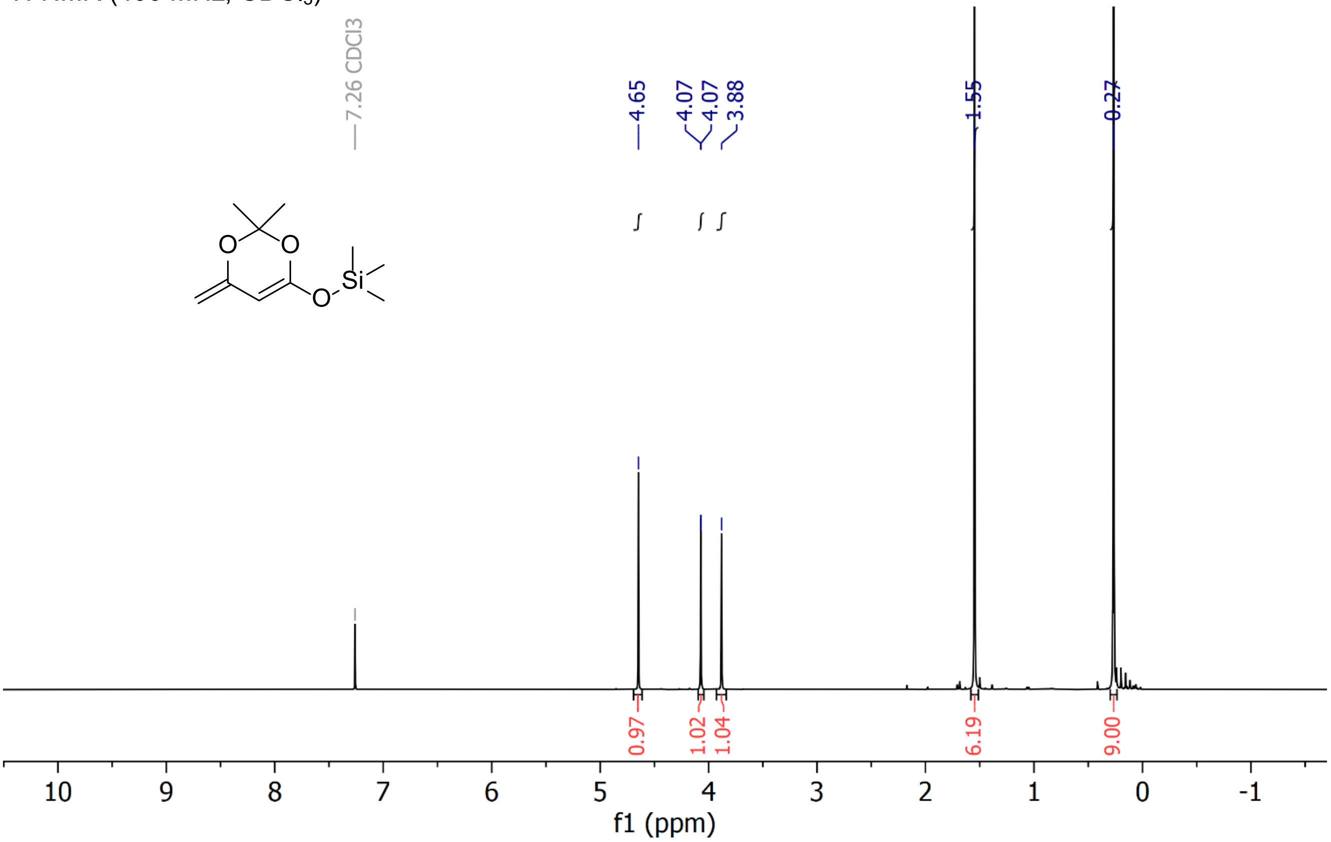

^{13}C-NMR (100 MHz, $CDCl_3$)

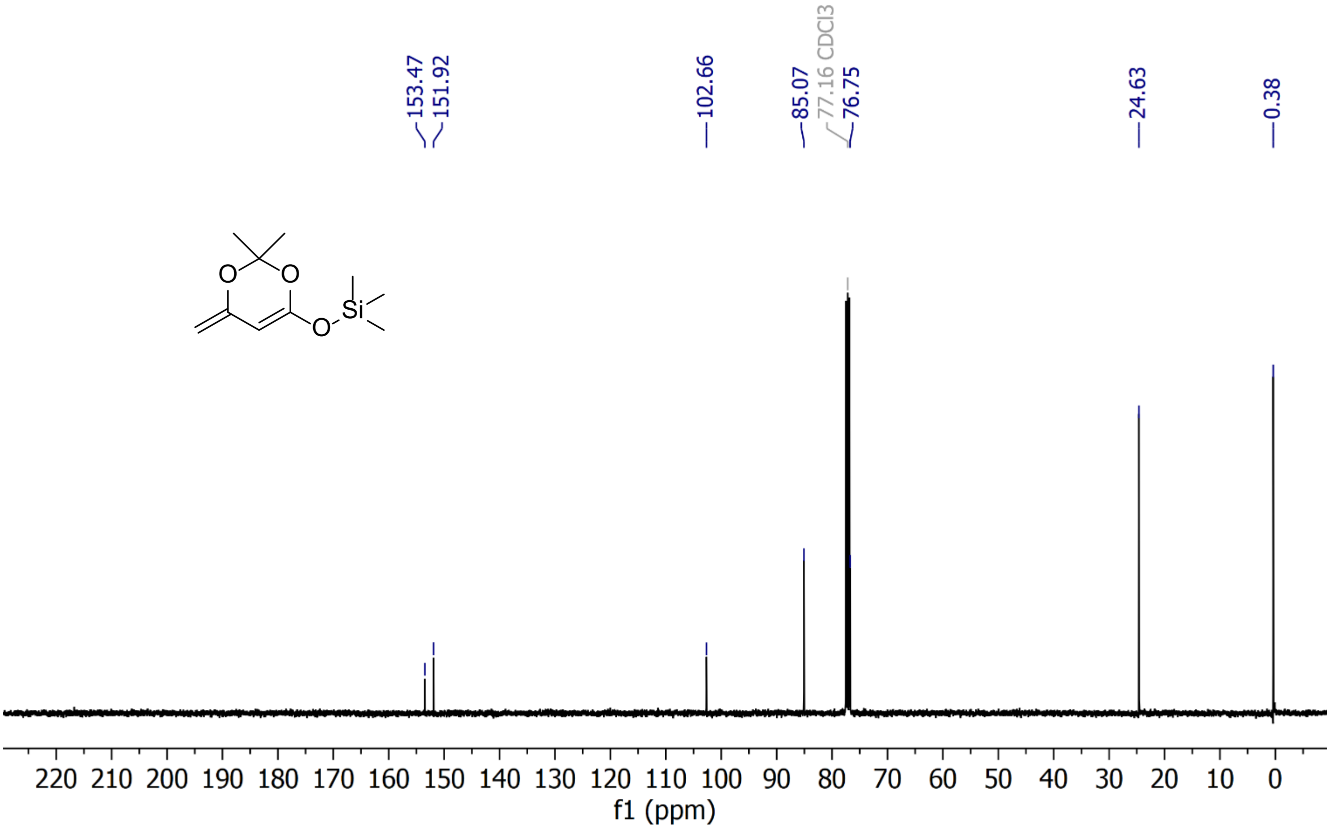

Trimethyl((2,2,5-trimethyl-4-methylen-4H-1,3-dioxin-6-yl)oxy)silane 17

^{1}H-NMR (400 MHz, $CDCl_3$)

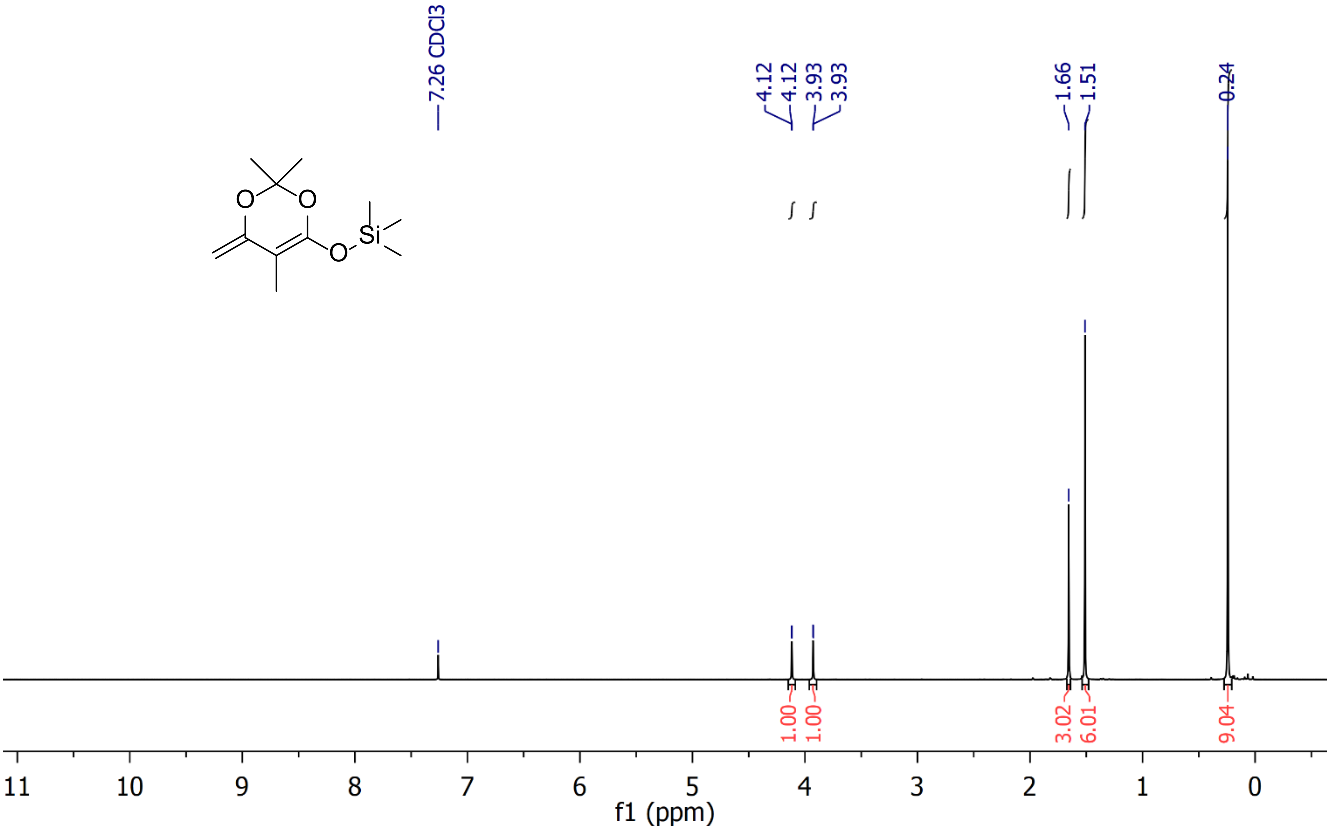

^{13}C-NMR (100 MHz, $CDCl_3$)

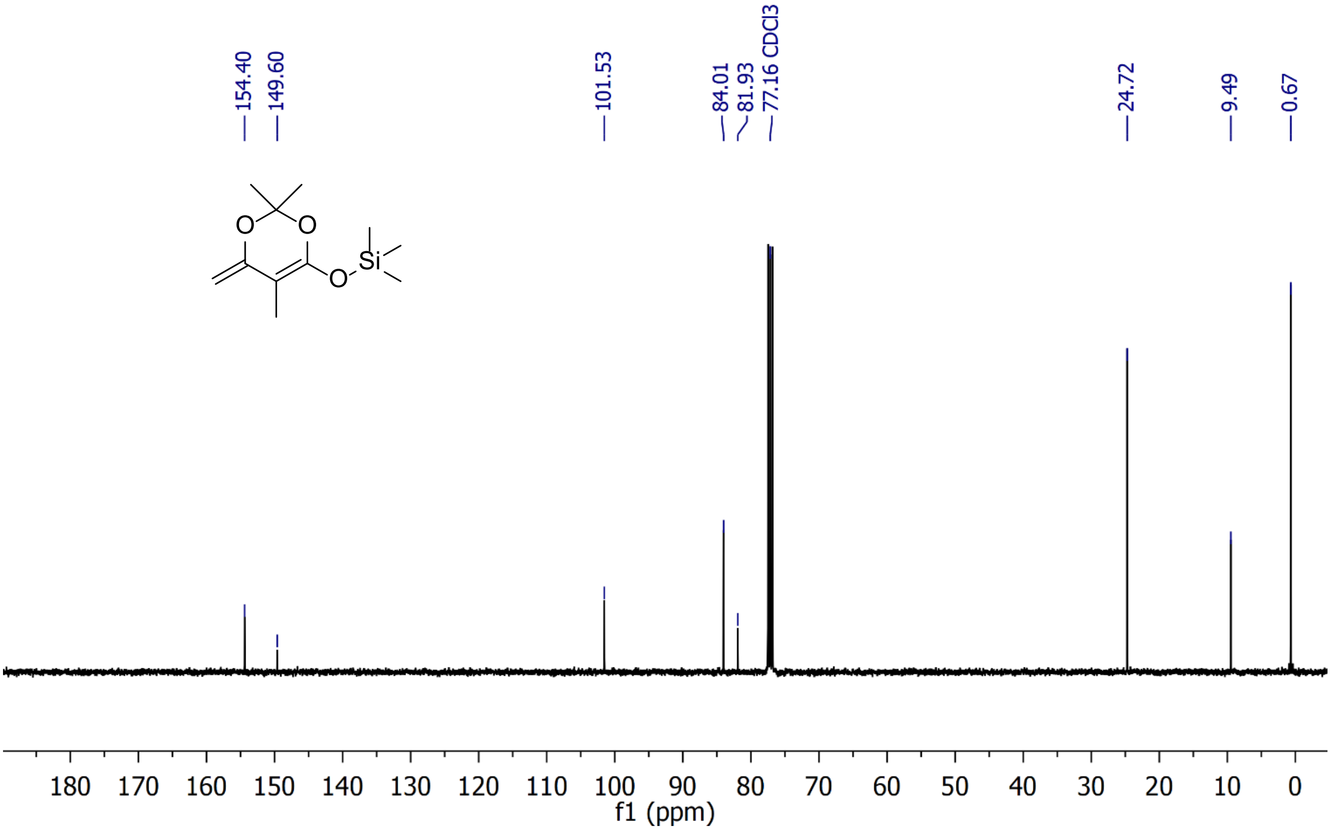

6-((2R,5S,E)-5-((*tert*-Butyldimethylsilyl)oxy)-2-hydroxyhex-3-en-1-yl)-2,2-dimethyl-4H-1,3-dioxin-4-one 20

^{1}H-NMR (400 MHz, $CDCl_3$)

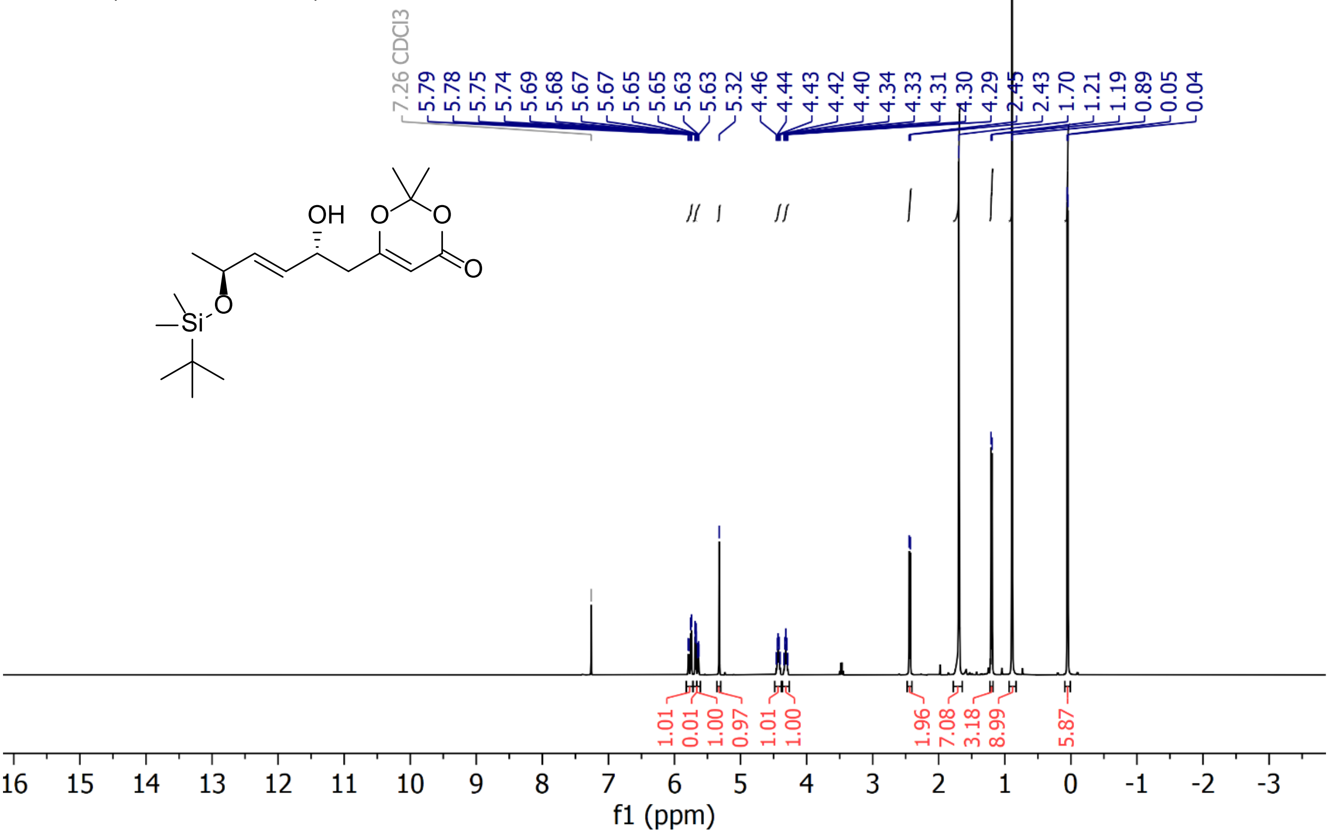

^{13}C-NMR (100 MHz, $CDCl_3$)

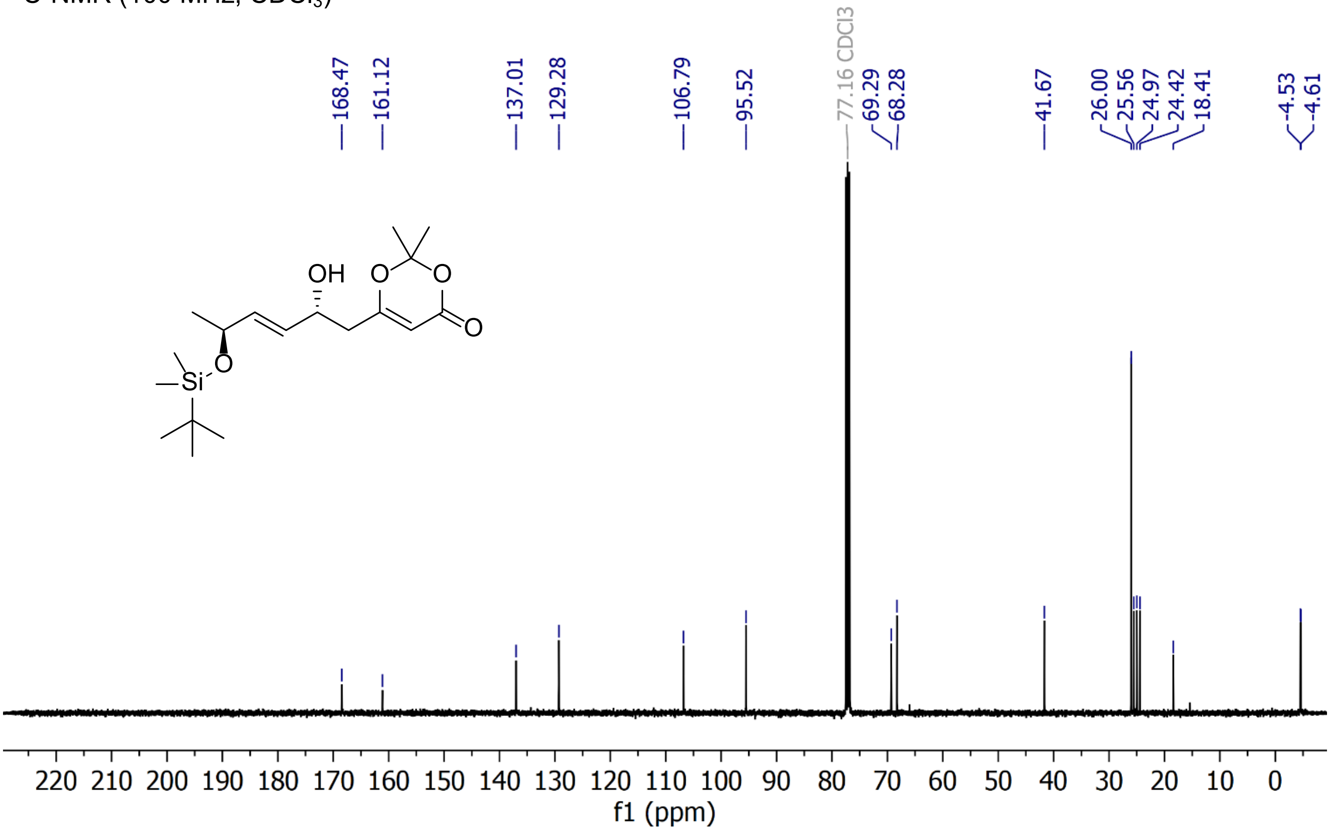

6-((2R,5S,E)-5-((*tert*-Butyldimethylsilyl)oxy)-2-hydroxyhex-3-en-1-yl)-2,2,5-trimethyl-4H-1,3-dioxin-4-one 19

^{1}H-NMR (400 MHz, $CDCl_3$)

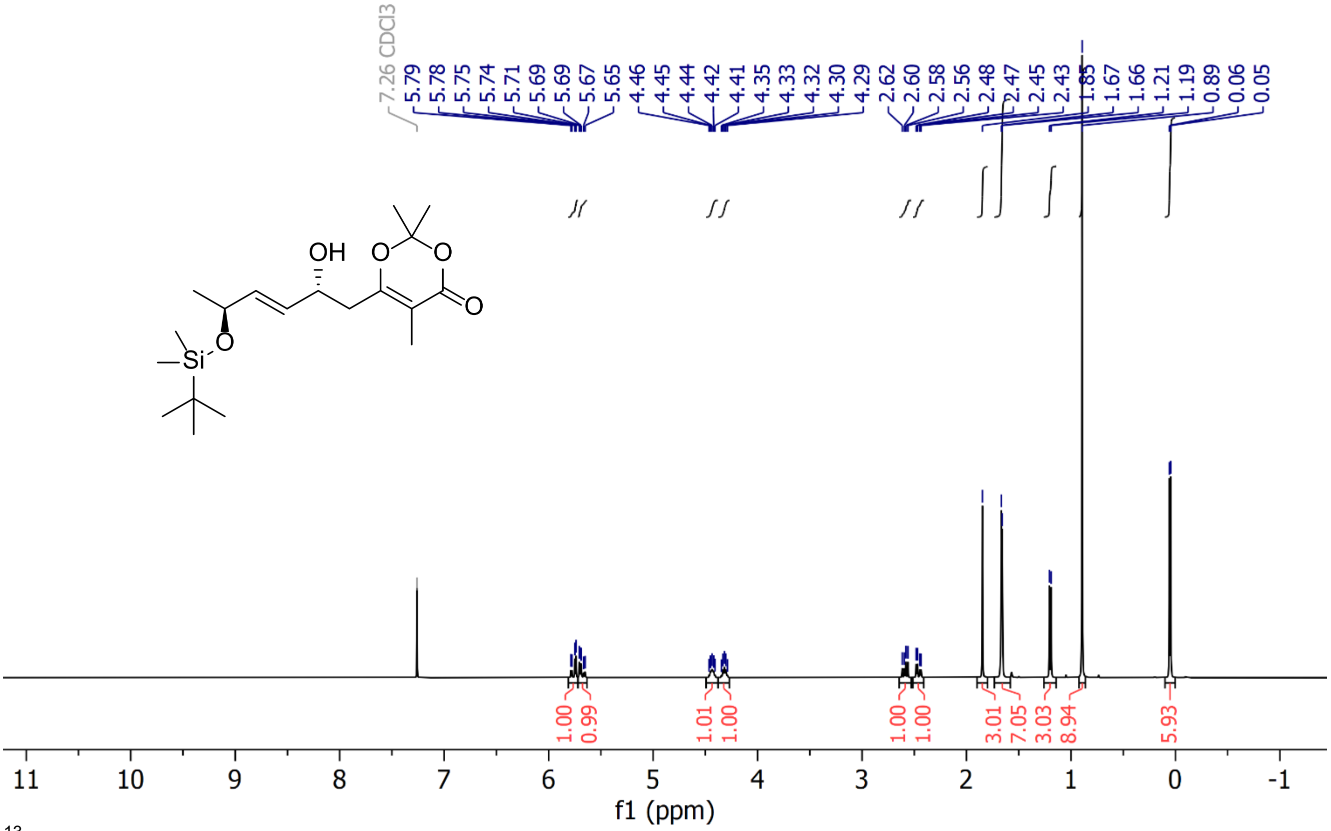

^{13}C-NMR (100 MHz, $CDCl_3$)

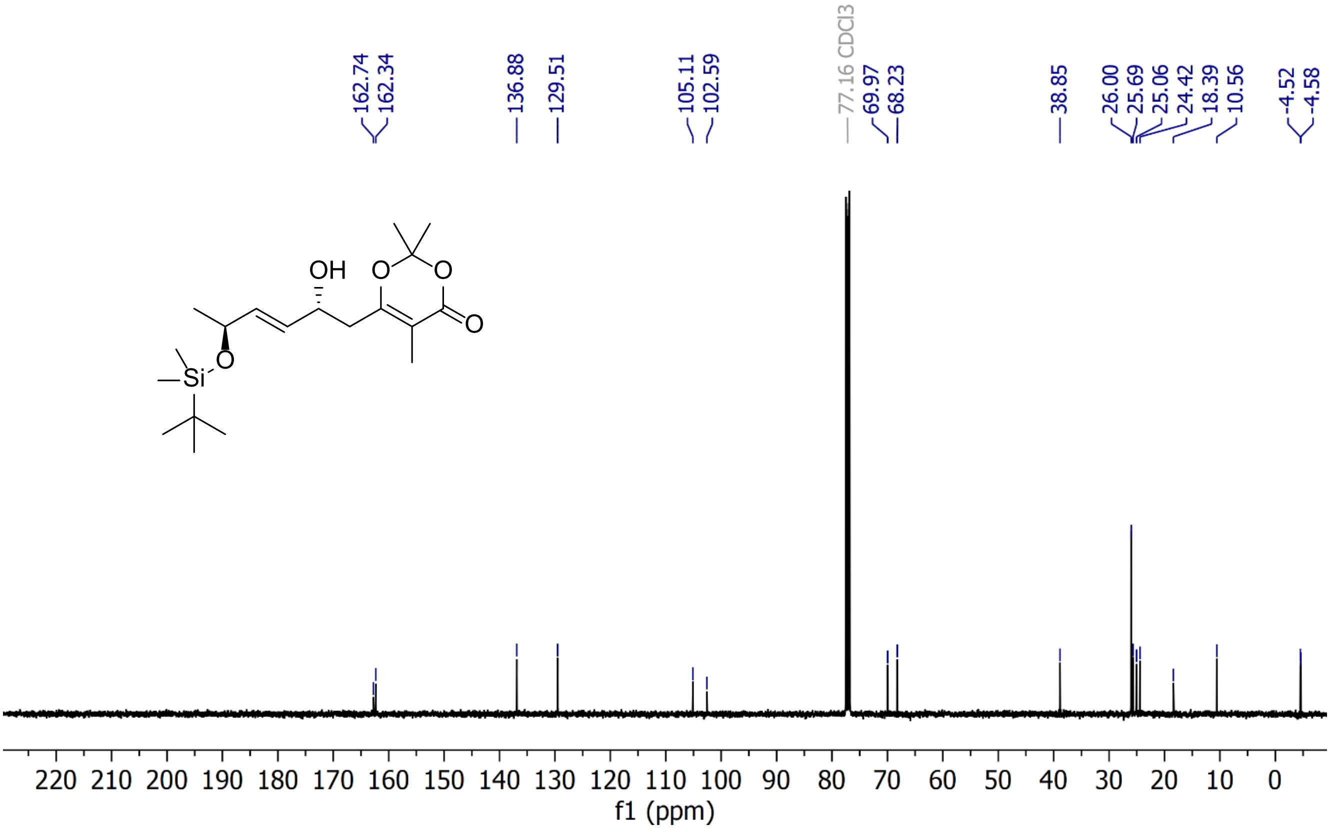

(R)-6-((S,E)-3-((*tert*-Butyldimethylsilyl)oxy)but-1-en-1-yl)-4-methoxy-5,6-dihydro-2H-pyran-2-one 21

^{1}H-NMR (400 MHz, $CDCl_3$)

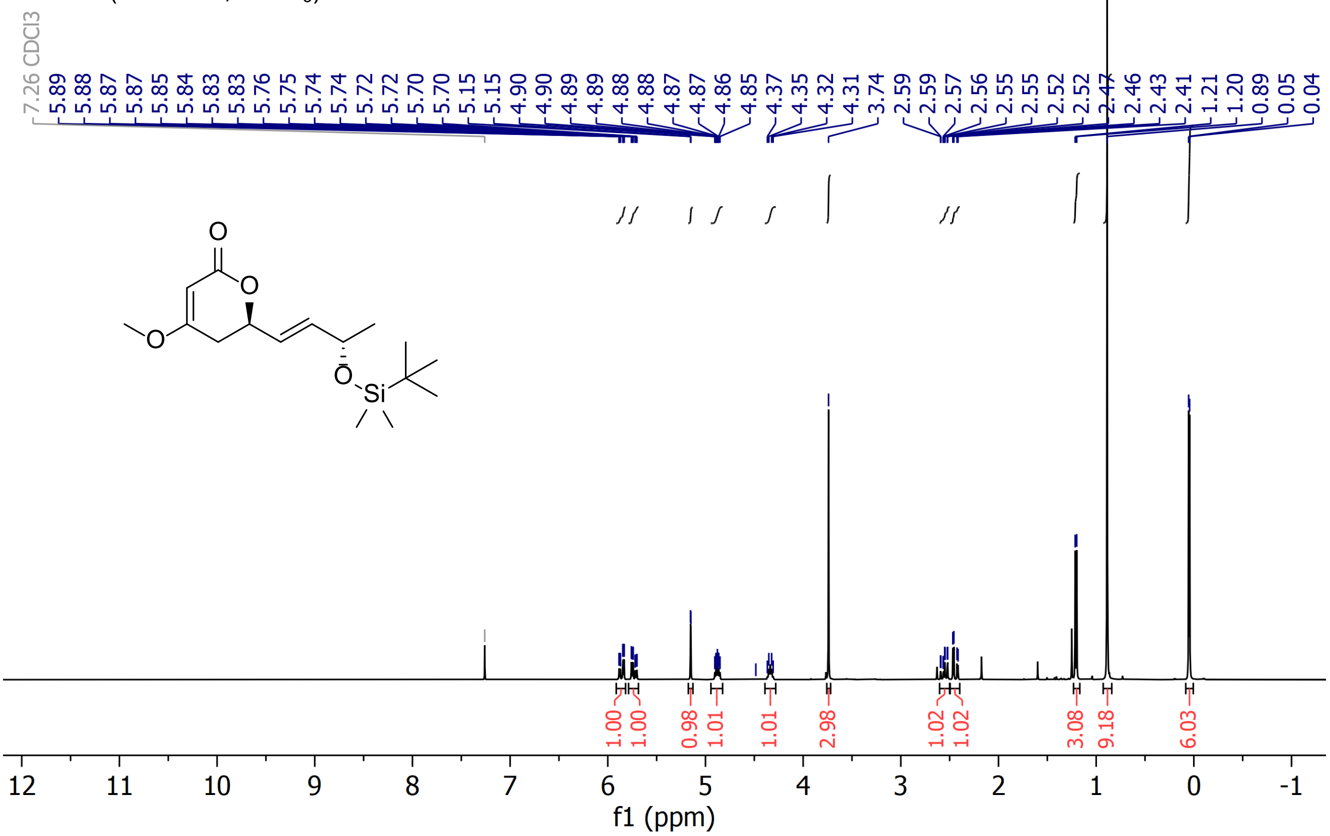

^{13}C-NMR (100 MHz, $CDCl_3$)

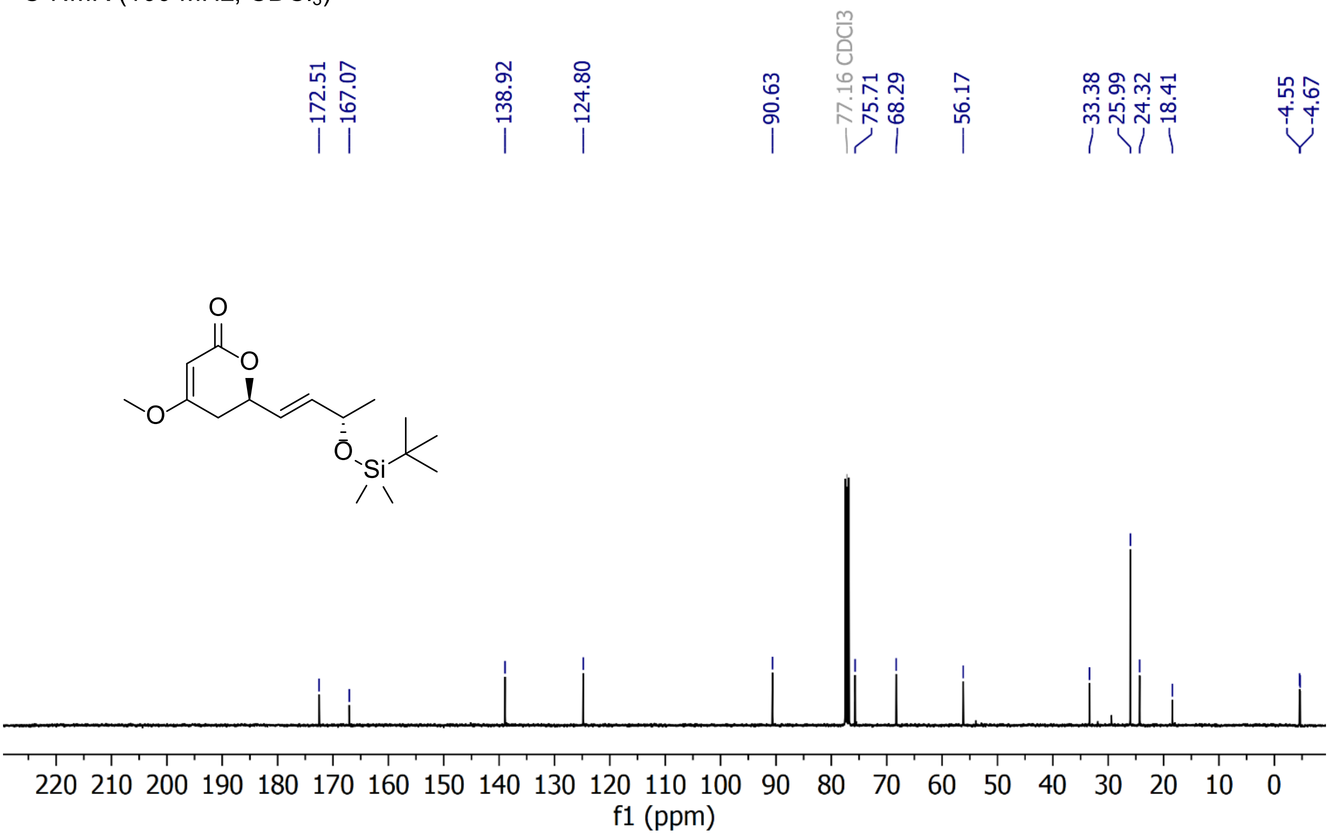

(R)-6-((S,E)-3-((*tert*-Butyldimethylsilyl)oxy)but-1-en-1-yl)-3-iodo-4-methoxy-5,6-dihydro-2H-pyran-2-one 22

^{1}H-NMR (400 MHz, $CDCl_3$)

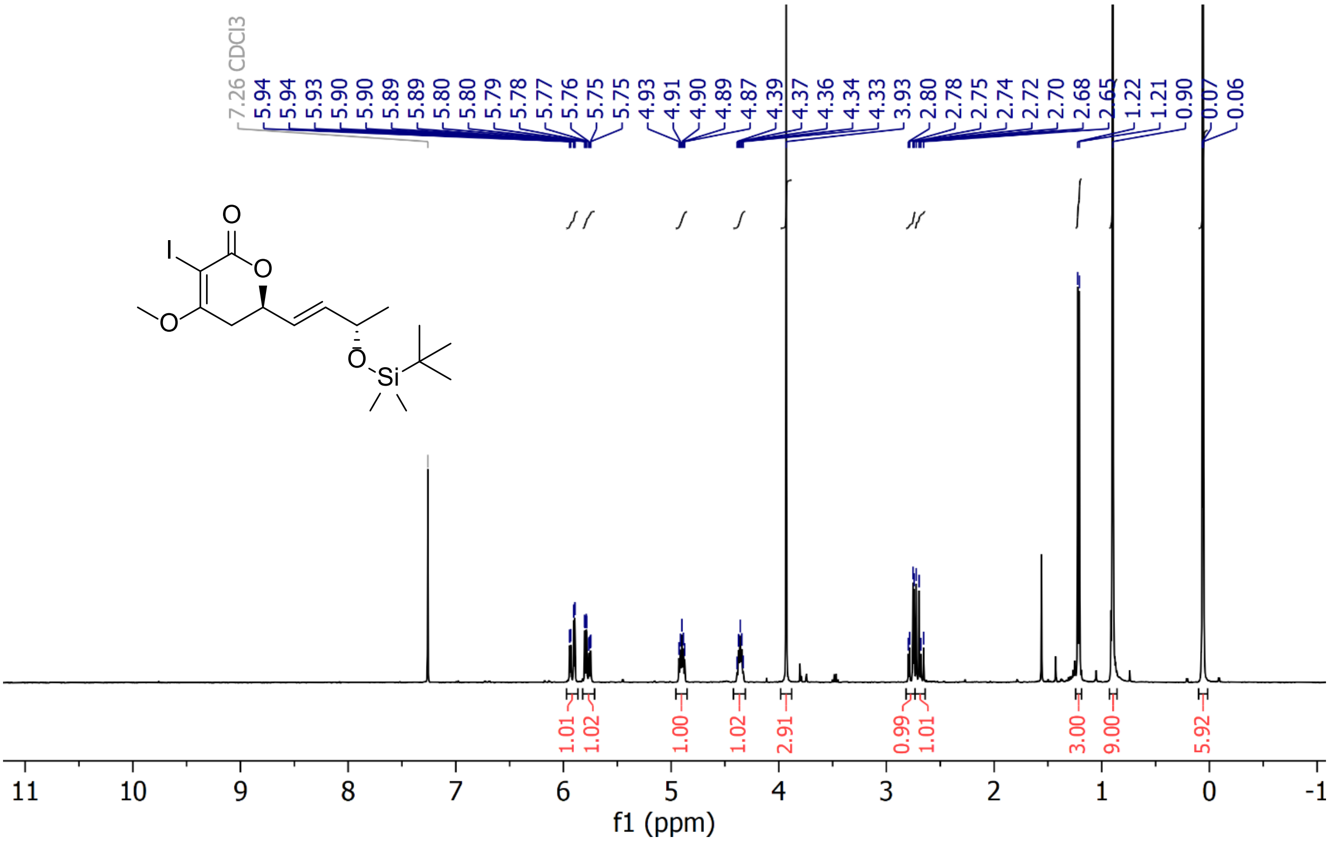

^{13}C-NMR (100 MHz, $CDCl_3$)

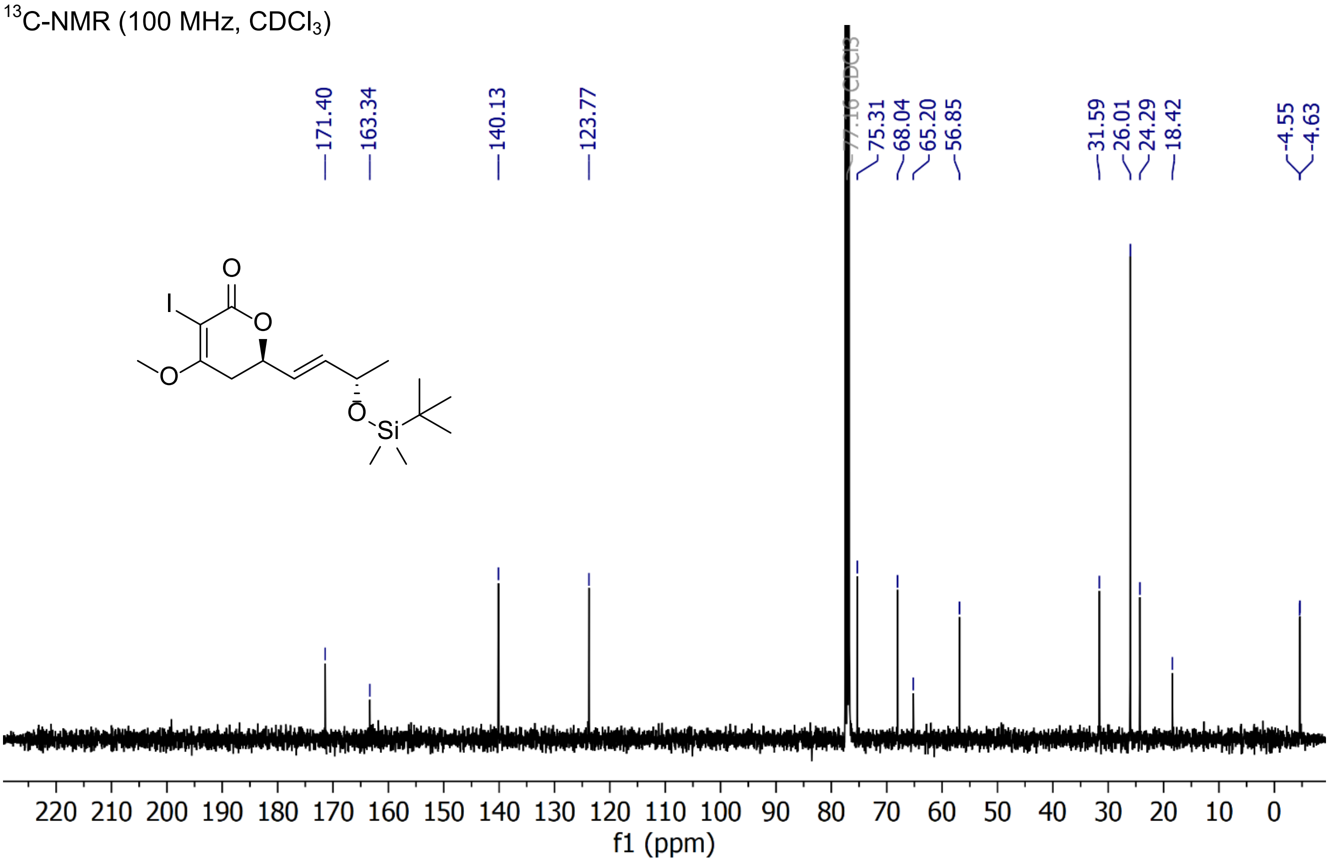

(R)-6-((S,E)-3-((*tert*-Butyldimethylsilyl)oxy)but-1-en-1-yl)-3-(hydroxymethyl)-4-methoxy-5,6-dihydro-2H-pyran-2-one 23

^{1}H-NMR (400 MHz, $CDCl_3$)

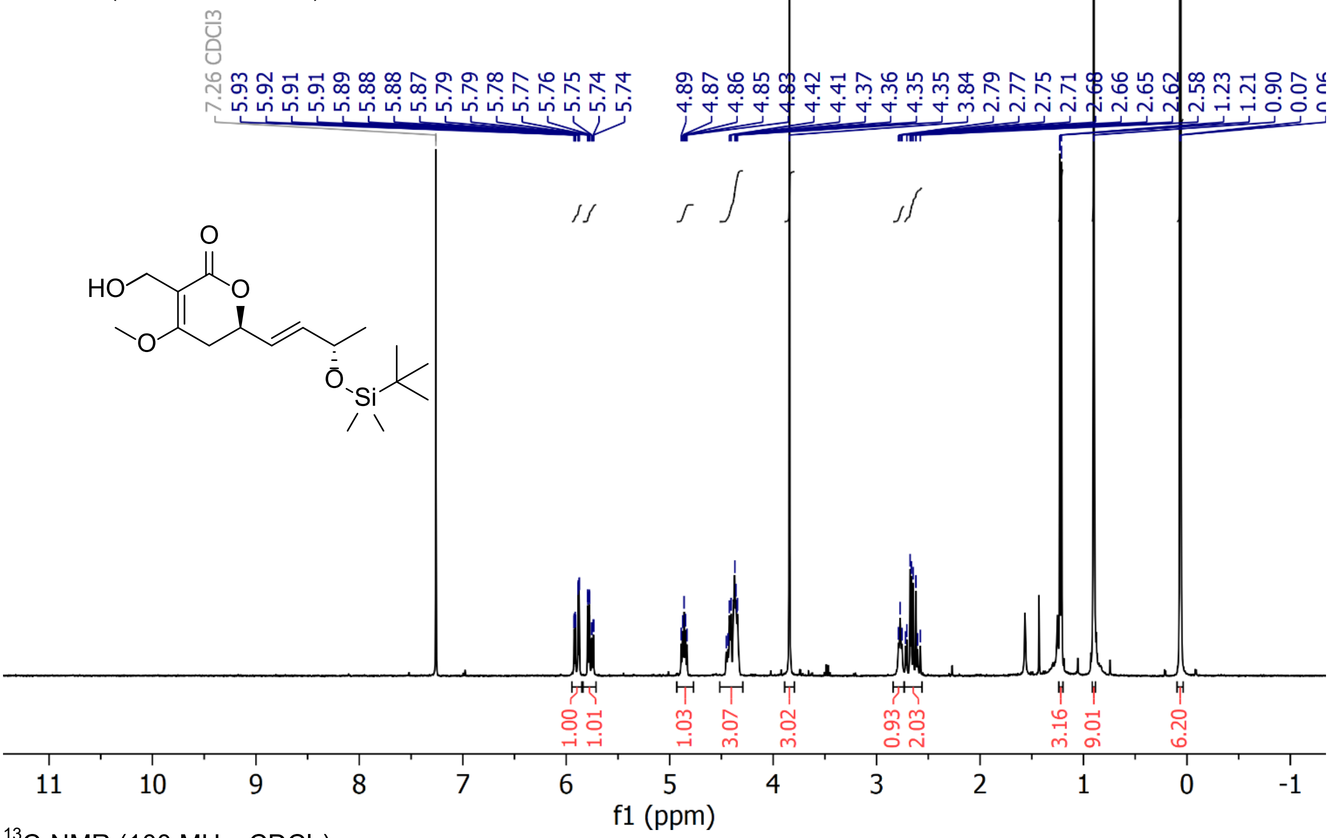

^{13}C-NMR (100 MHz, $CDCl_3$)

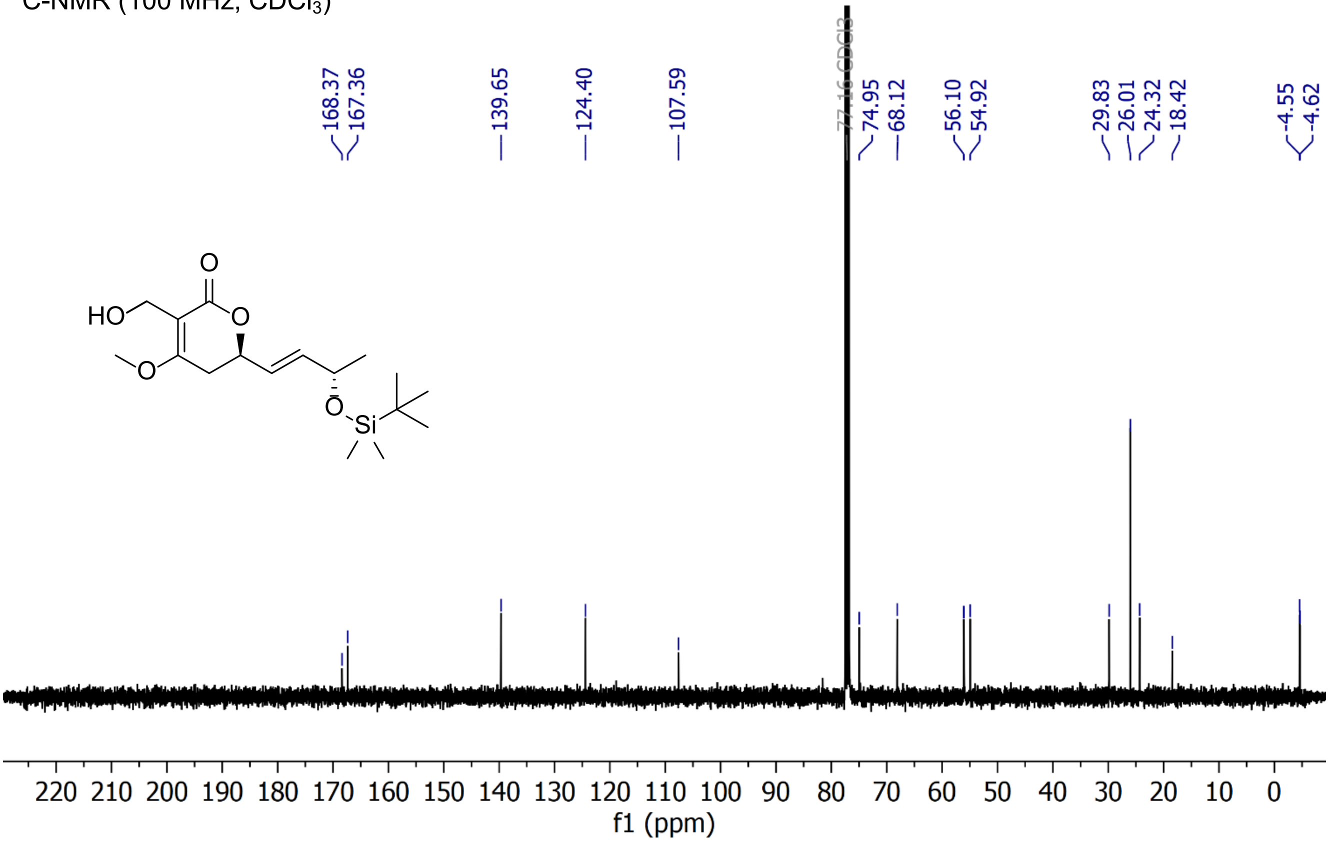

(R)-6-((S,E)-3-((*tert*-Butyldimethylsilyl)oxy)but-1-en-1-yl)-4-methoxy-3-(((4-methoxybenzyl)oxy)methyl)-5,6-dihydro-2H-pyran-2-one 24

^{1}H-NMR (400 MHz, $CDCl_3$)

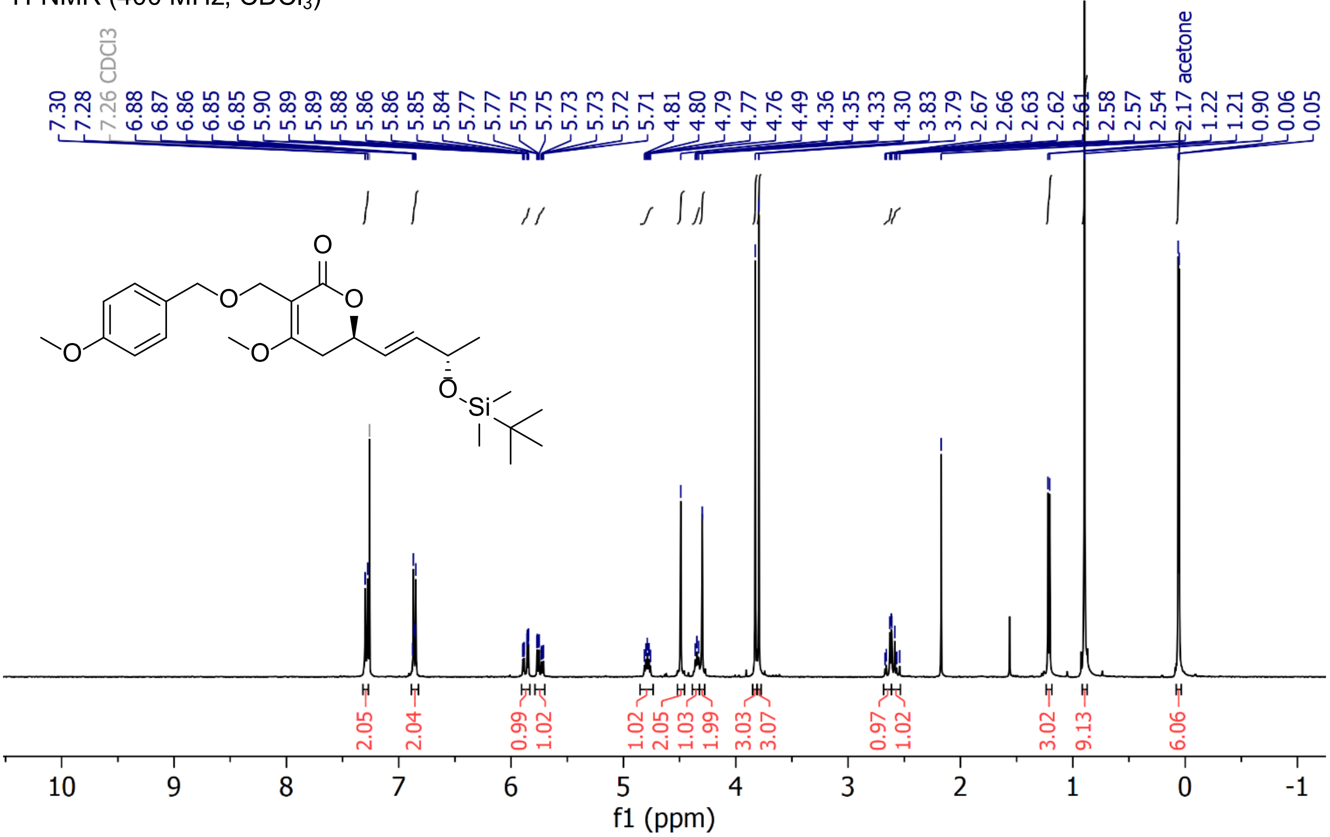

^{13}C-NMR (100 MHz, $CDCl_3$)

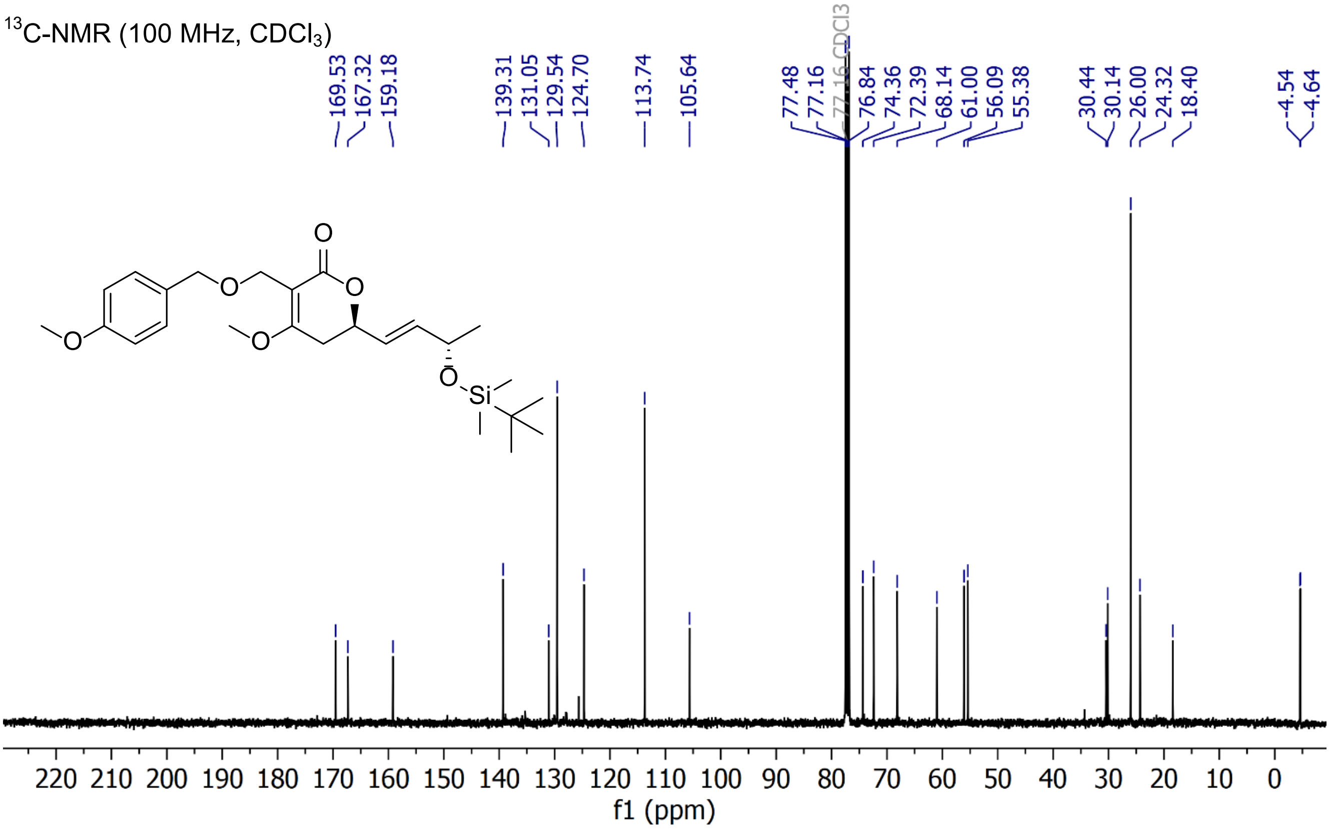

(R)-6-((S,E)-3-Hydroxybut-1-en-1-yl)-4-methoxy-3-(((4-methoxybenzyl)oxy)methyl)-5,6-dihydro-2H-pyran-2-one S4

^{1}H-NMR (400 MHz, $CDCl_3$)

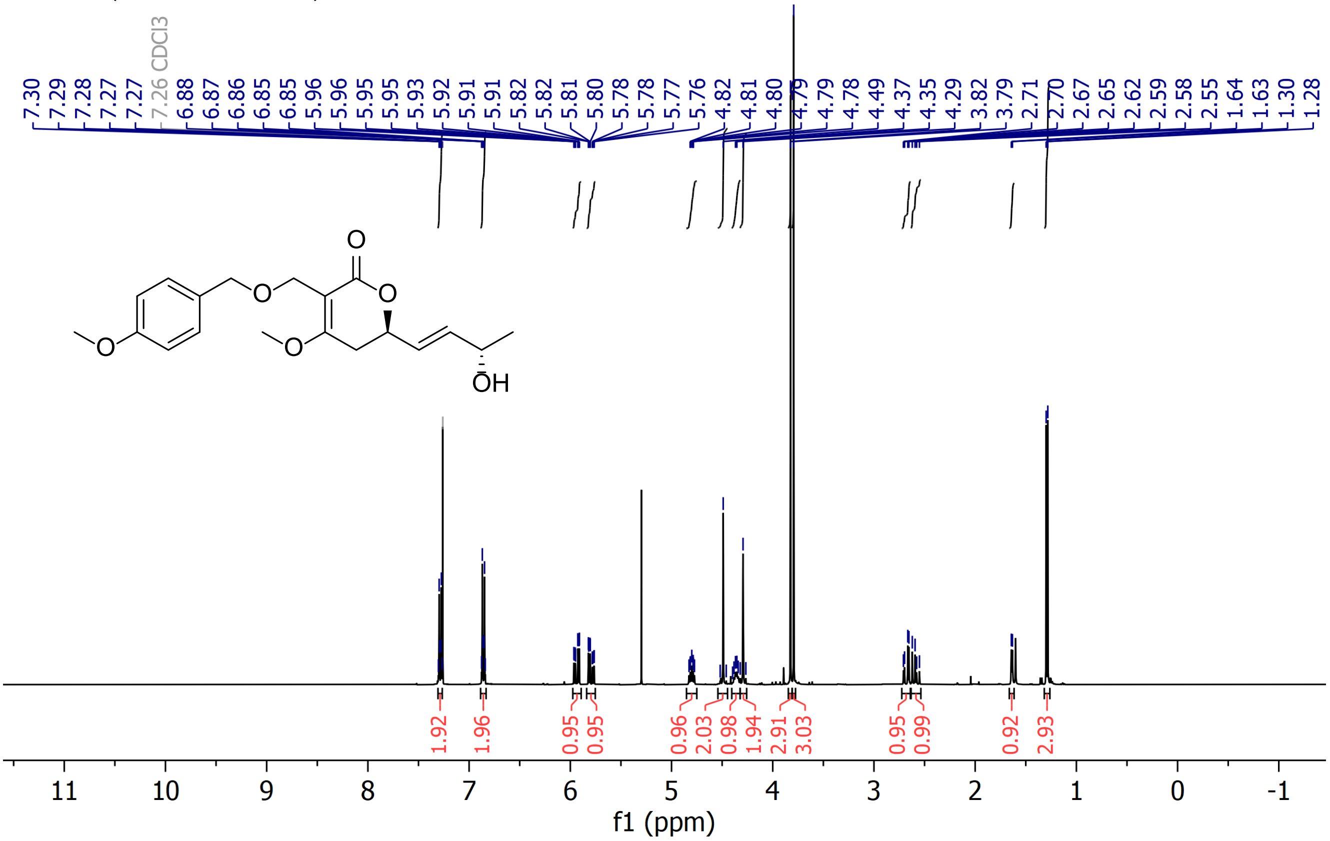

^{13}C-NMR (100 MHz, $CDCl_3$)

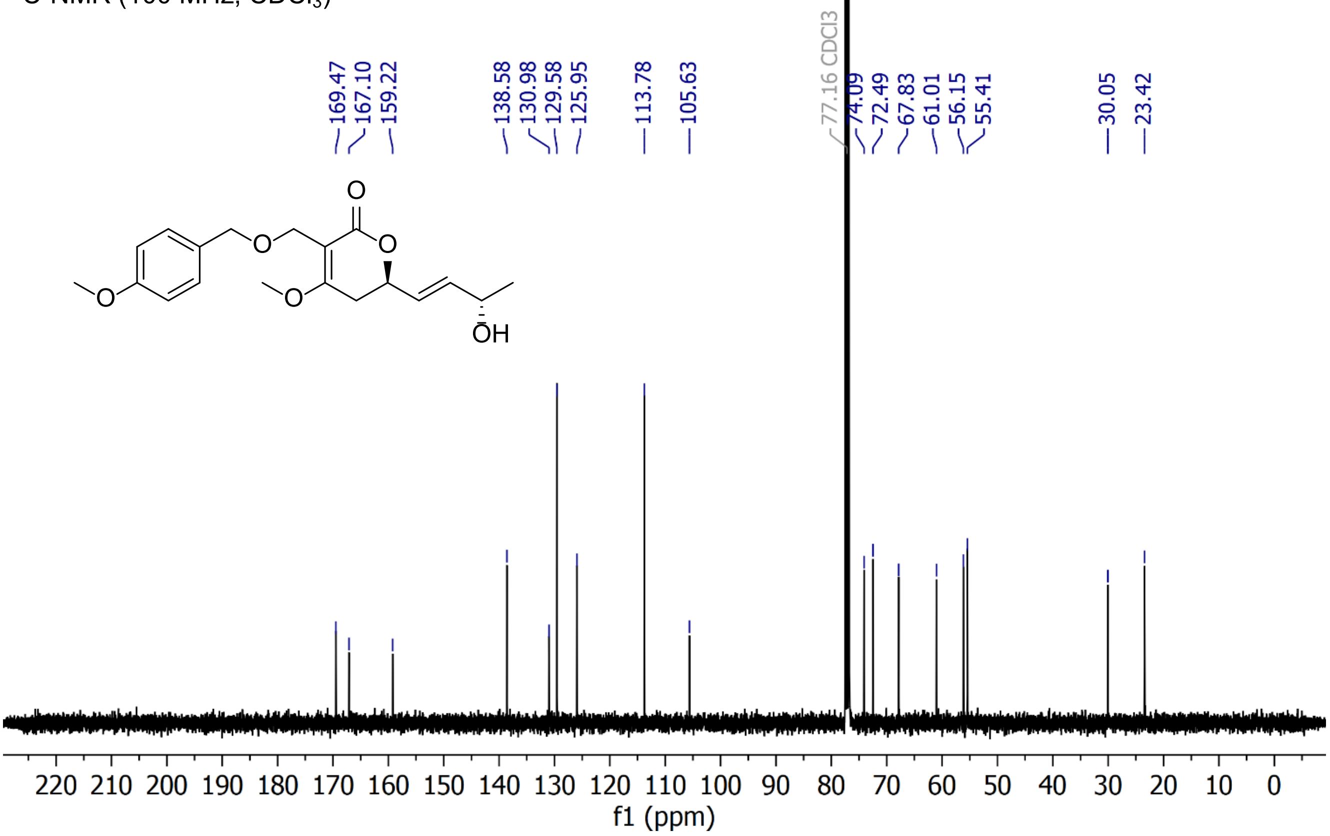

(S,E)-4-((R)-4-Methoxy-5-(((4-methoxybenzyl)oxy)methyl)-6-oxo-3,6-dihydro-2H-pyran-2-yl)but-3-en-2-yl 2,2,2-trifluoroacetate 25

^{1}H-NMR (400 MHz, $CDCl_3$)

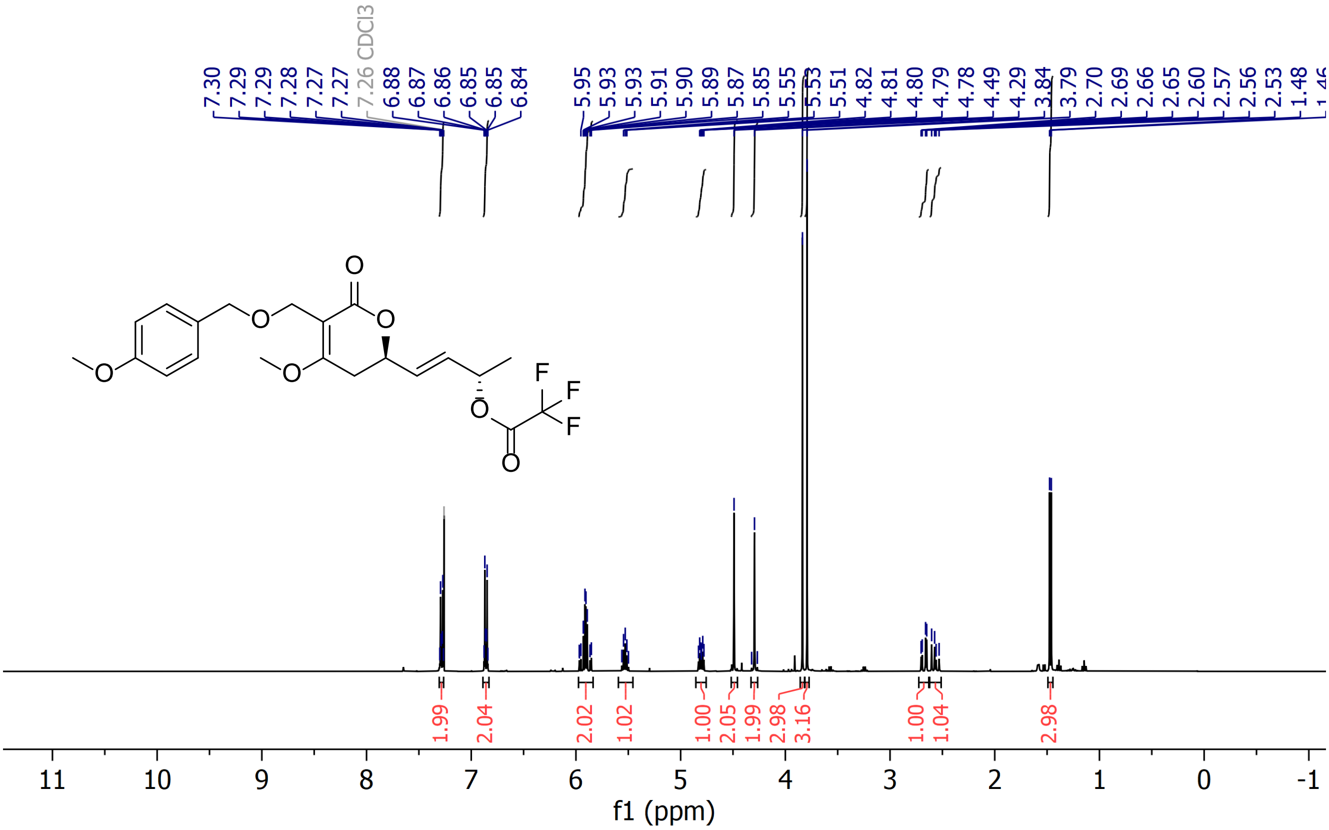

^{13}C-NMR (100 MHz, $CDCl_3$)

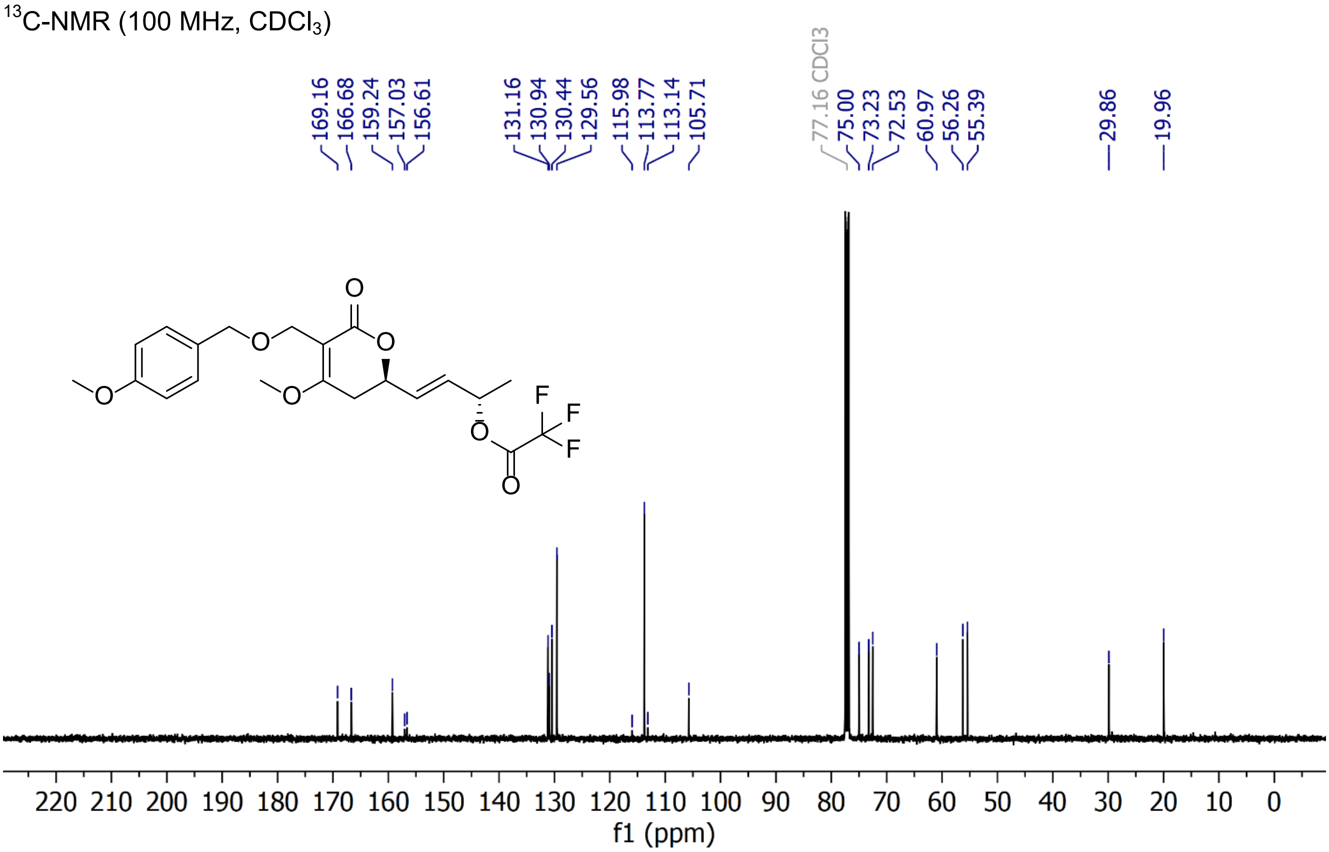

5-(Iodomethyl)-1-aza-5-stannabicyclo[3.3.3]undecane 27

^{1}H-NMR (400 MHz, $CDCl_3$)

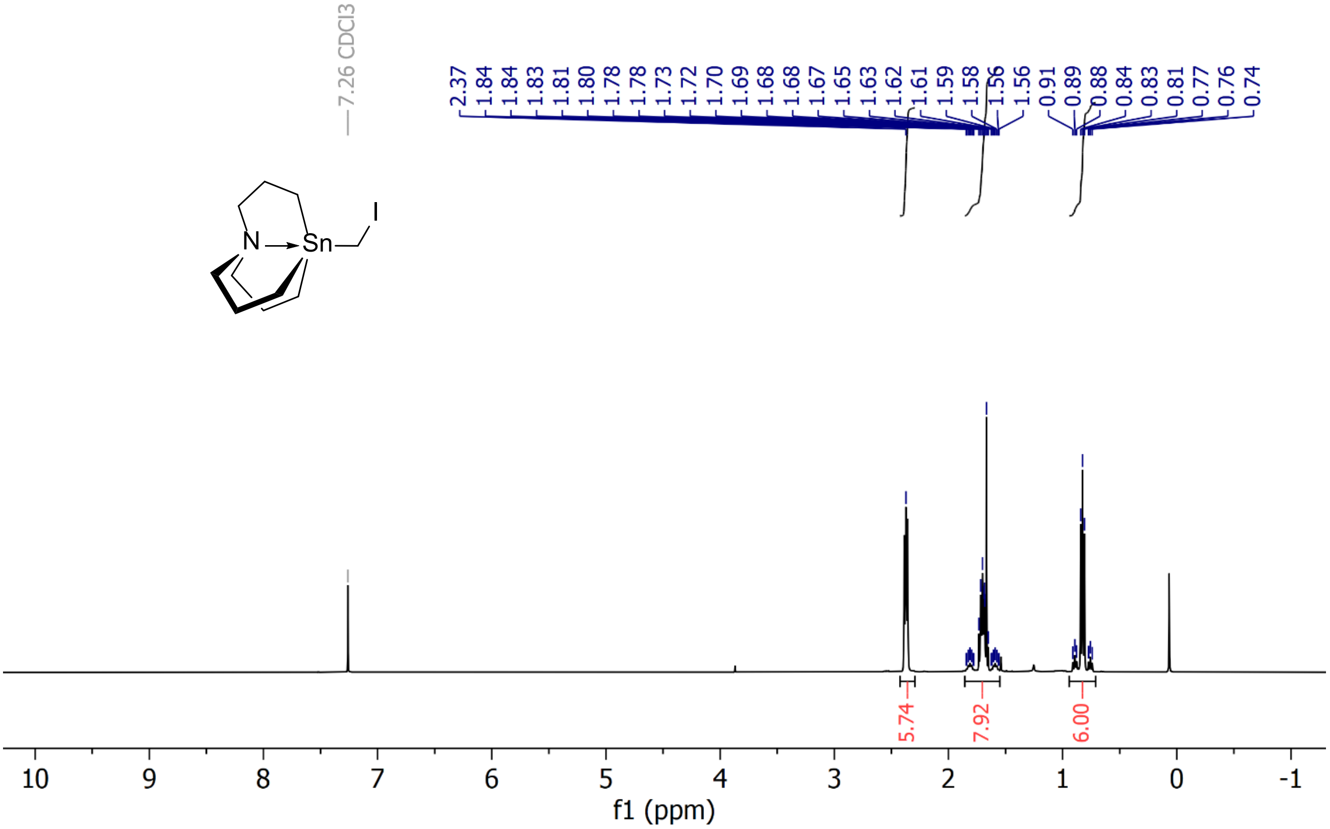

^{13}C-NMR (100 MHz, $CDCl_3$)

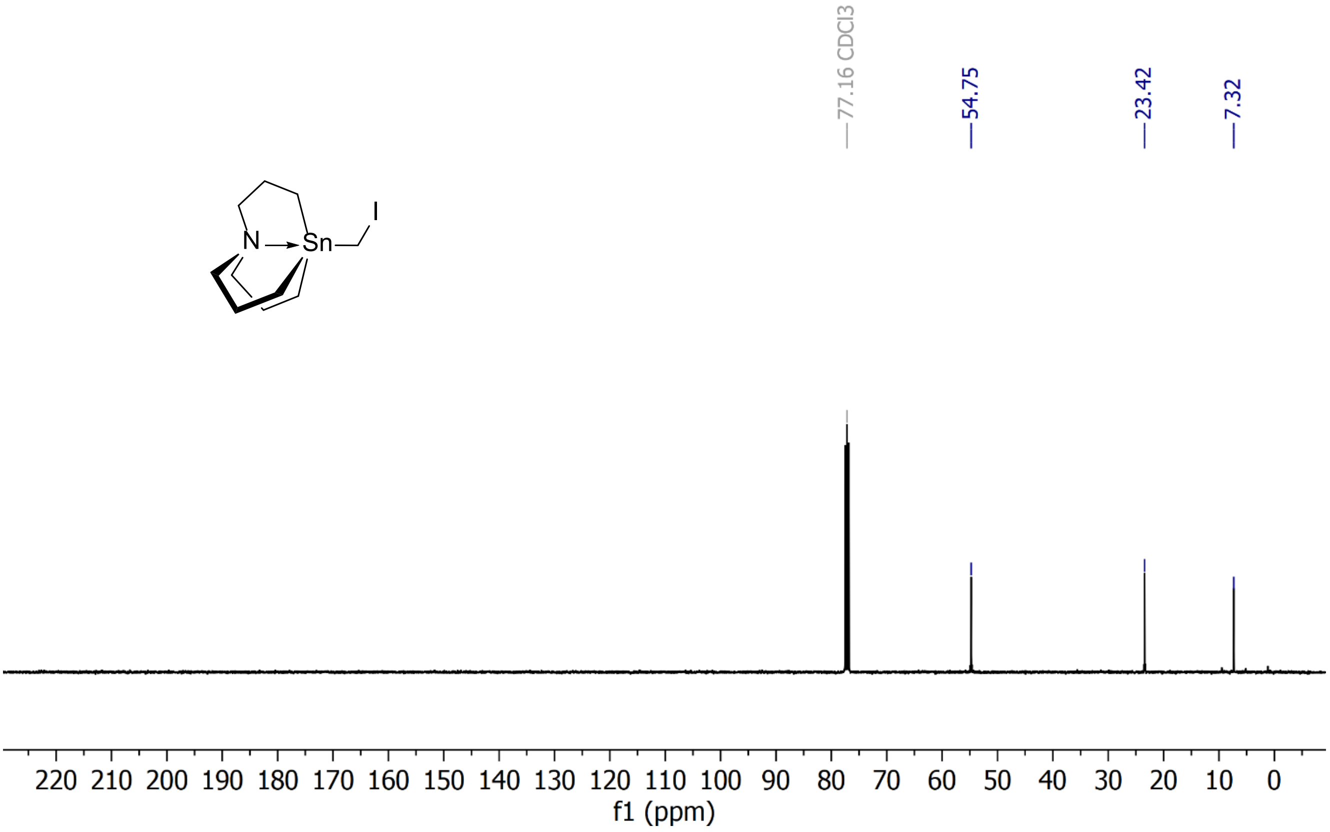

5-(((4-Methoxybenzyl)oxy)methyl)-1-aza-5-stannabicyclo[3.3.3]undecane 28

^{1}H-NMR (400 MHz, $CDCl_3$)

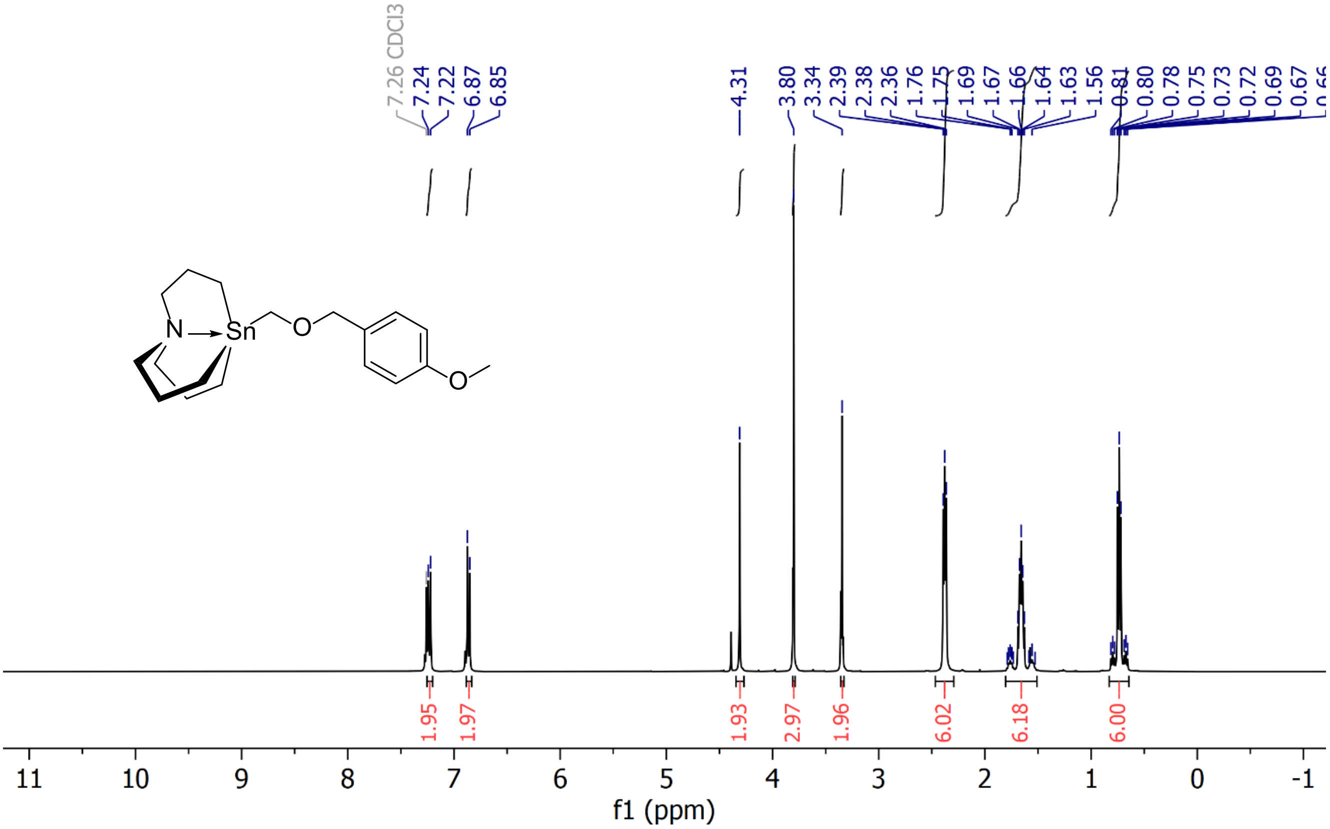

^{13}C-NMR (100 MHz, $CDCl_3$)

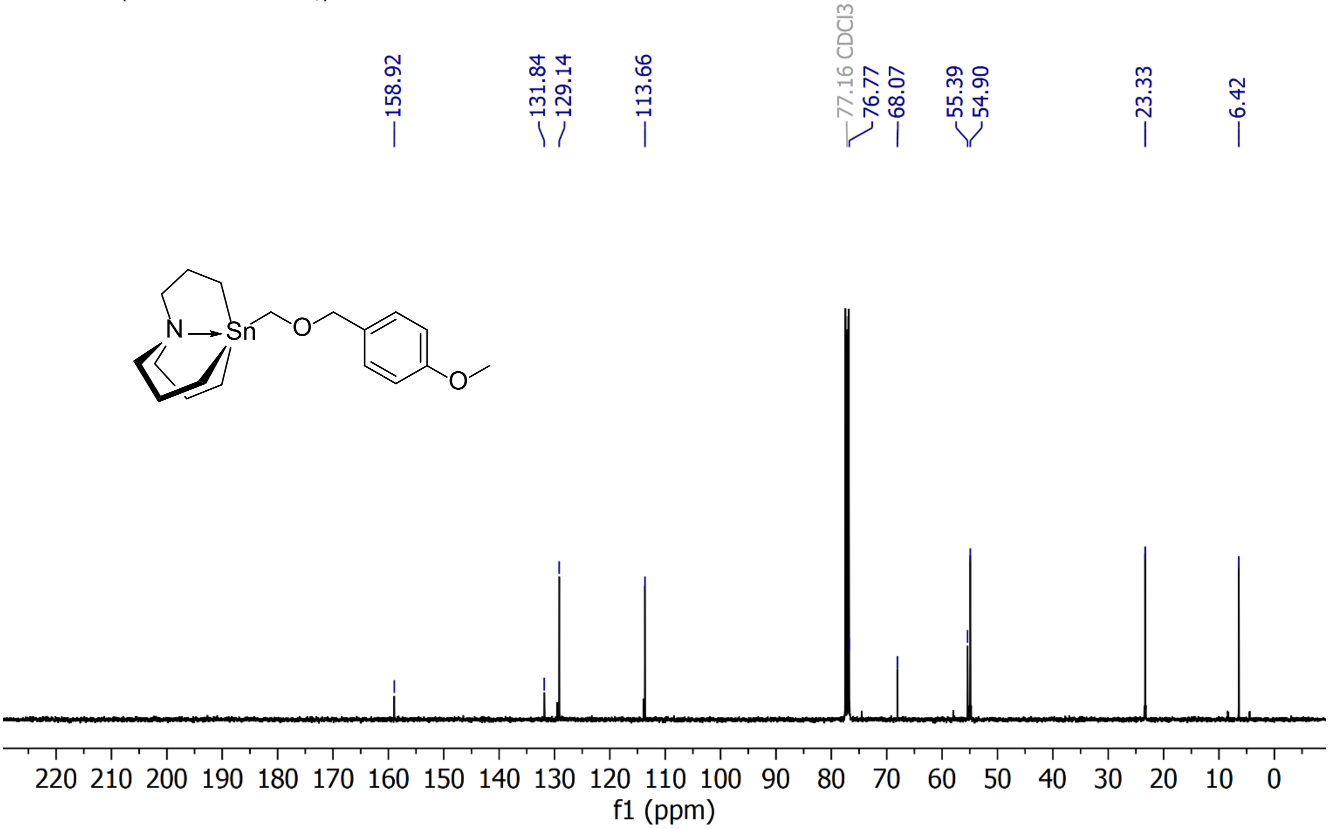

3-((2S,3R)-3-ethyl-3-(trimethylsilyl)oxiran-2-yl)propan-1-ol 30

^{1}H-NMR (400 MHz, $CDCl_3$)

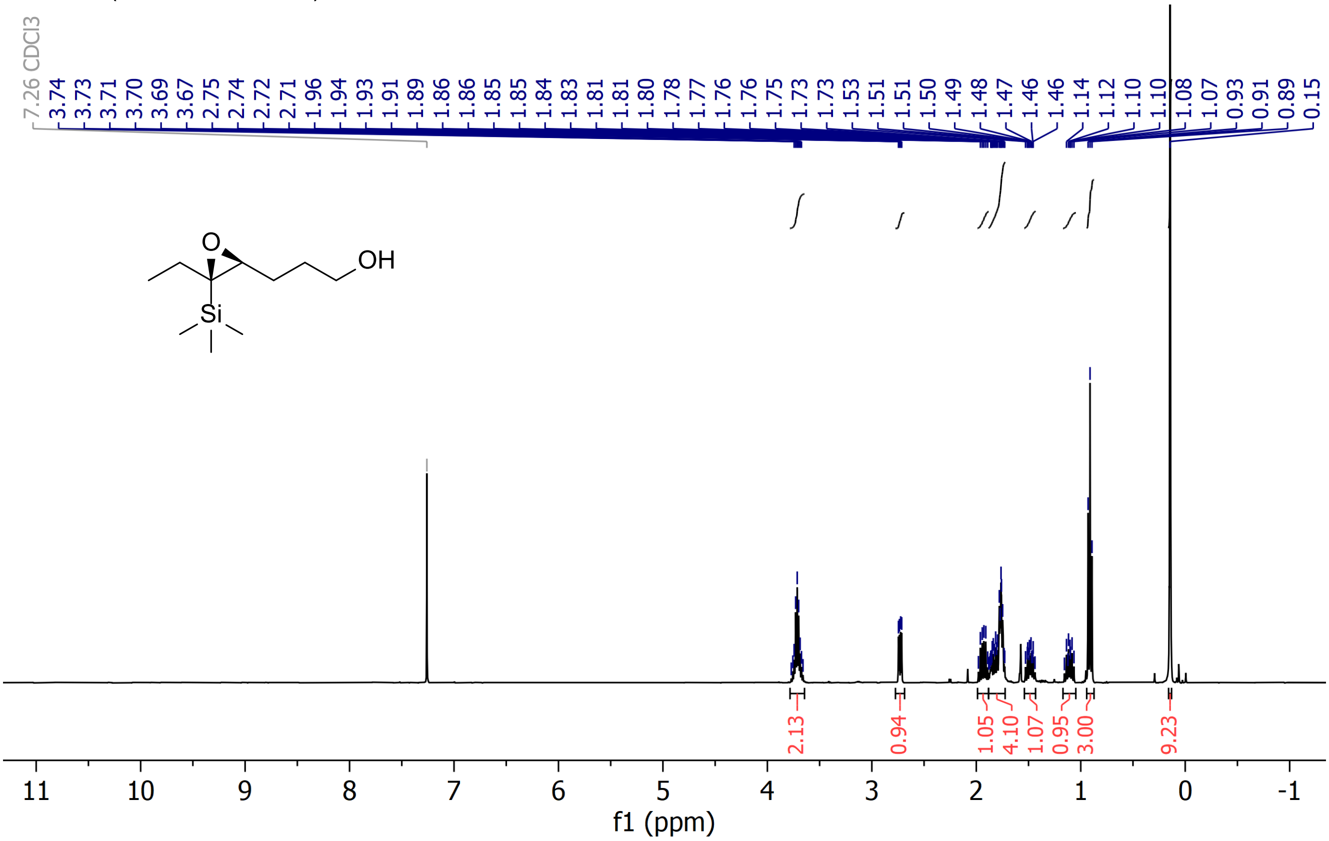

^{13}C-NMR (100 MHz, $CDCl_3$)

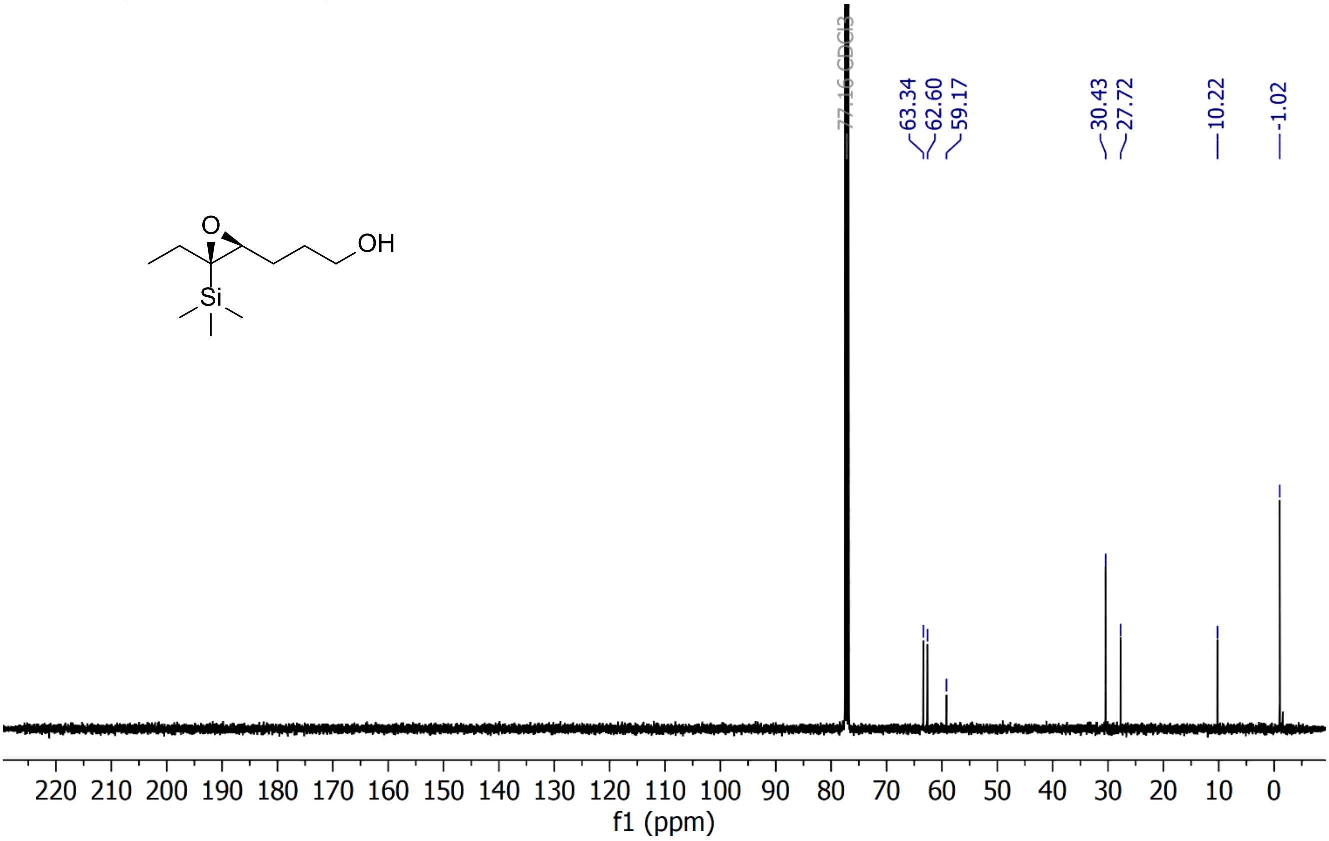

3-((2S,3R)-3-ethyl-3-(trimethylsilyl)oxiran-2-yl)propanal 32

^{1}H-NMR (400 MHz, $CDCl_3$)

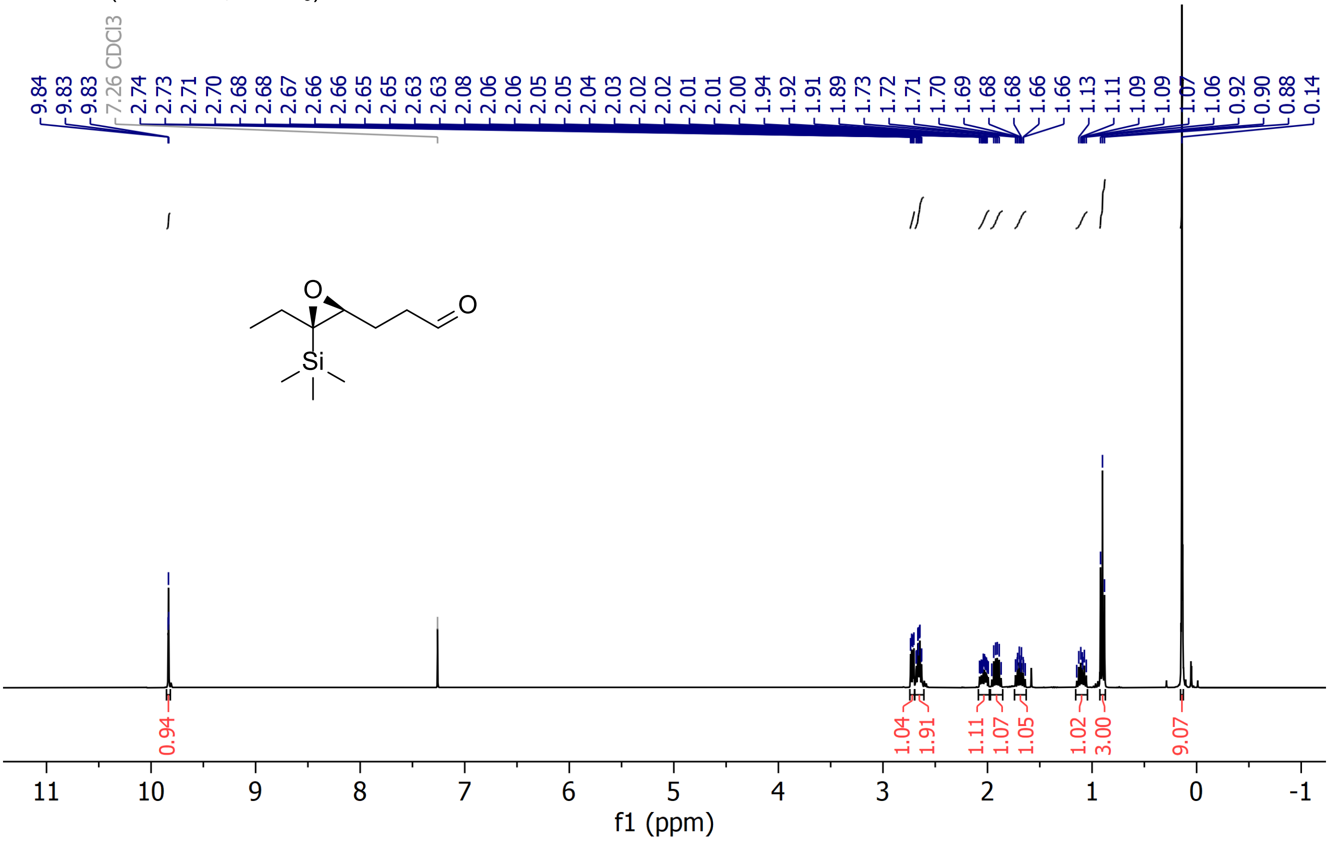

^{13}C-NMR (100 MHz, $CDCl_3$)

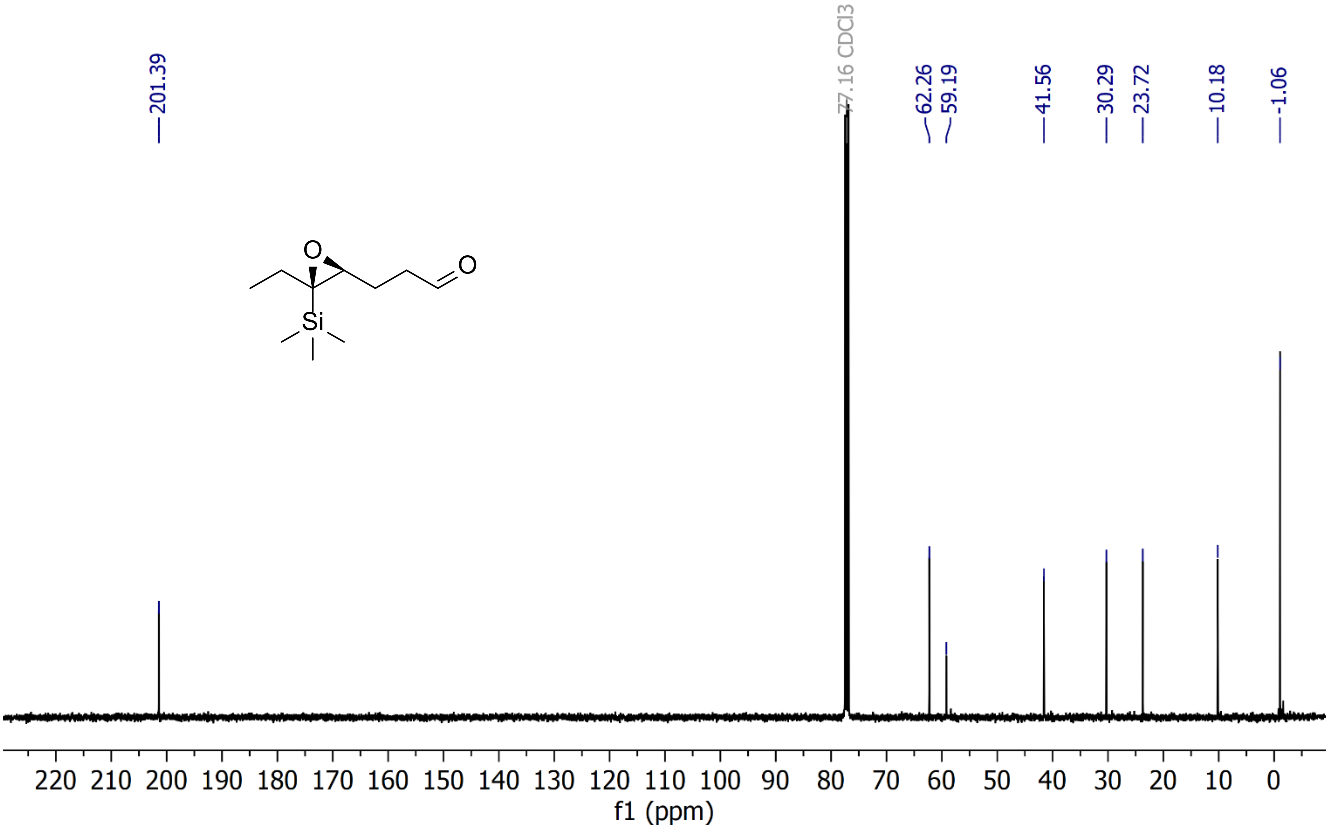

(2S,3S,6R)-2-ethyl-2-(trimethylsilyl)-6-((trimethylsilyl)ethynyl)tetrahydro-2H-pyran-3-ol 33

^{1}H-NMR (400 MHz, $CDCl_3$)

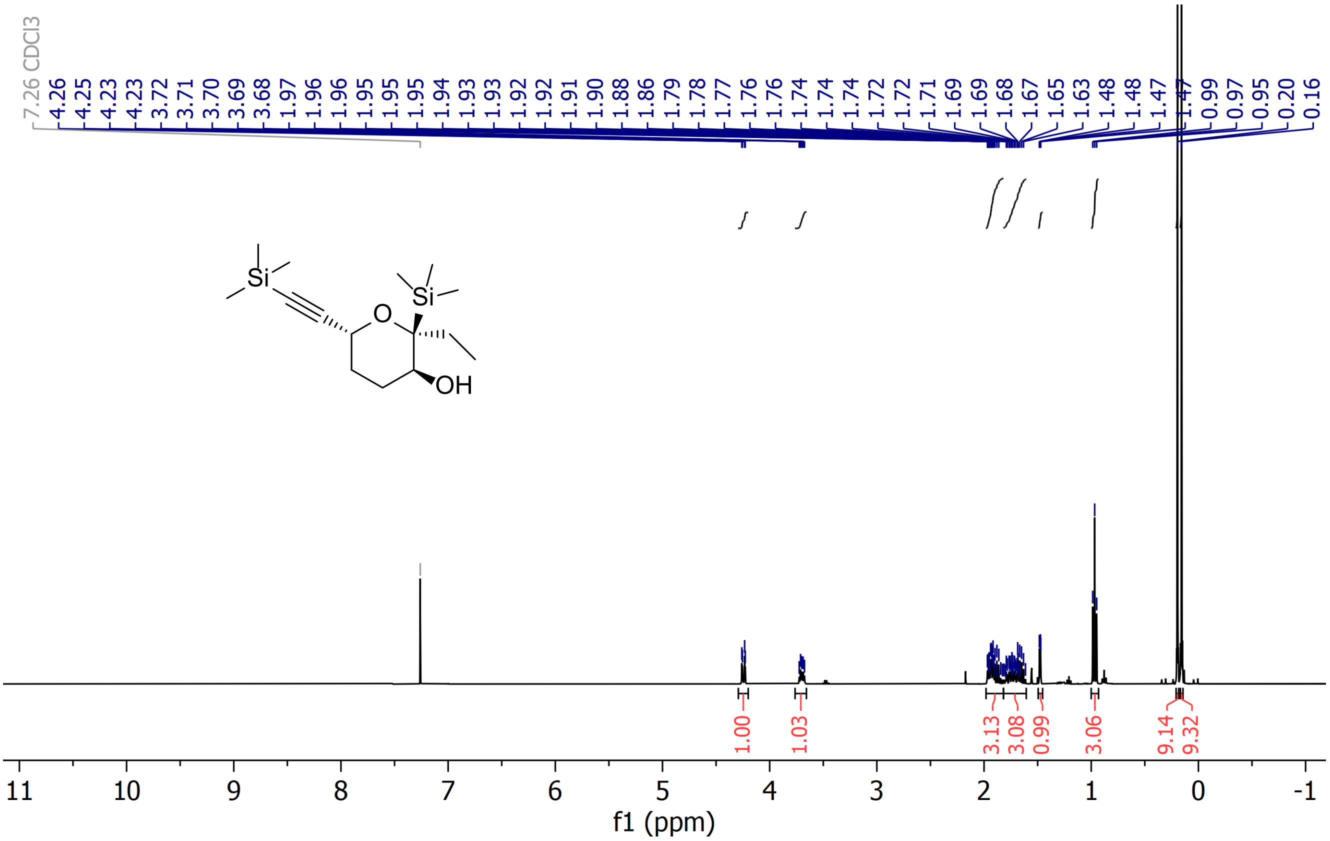

^{13}C-NMR (100 MHz, $CDCl_3$)

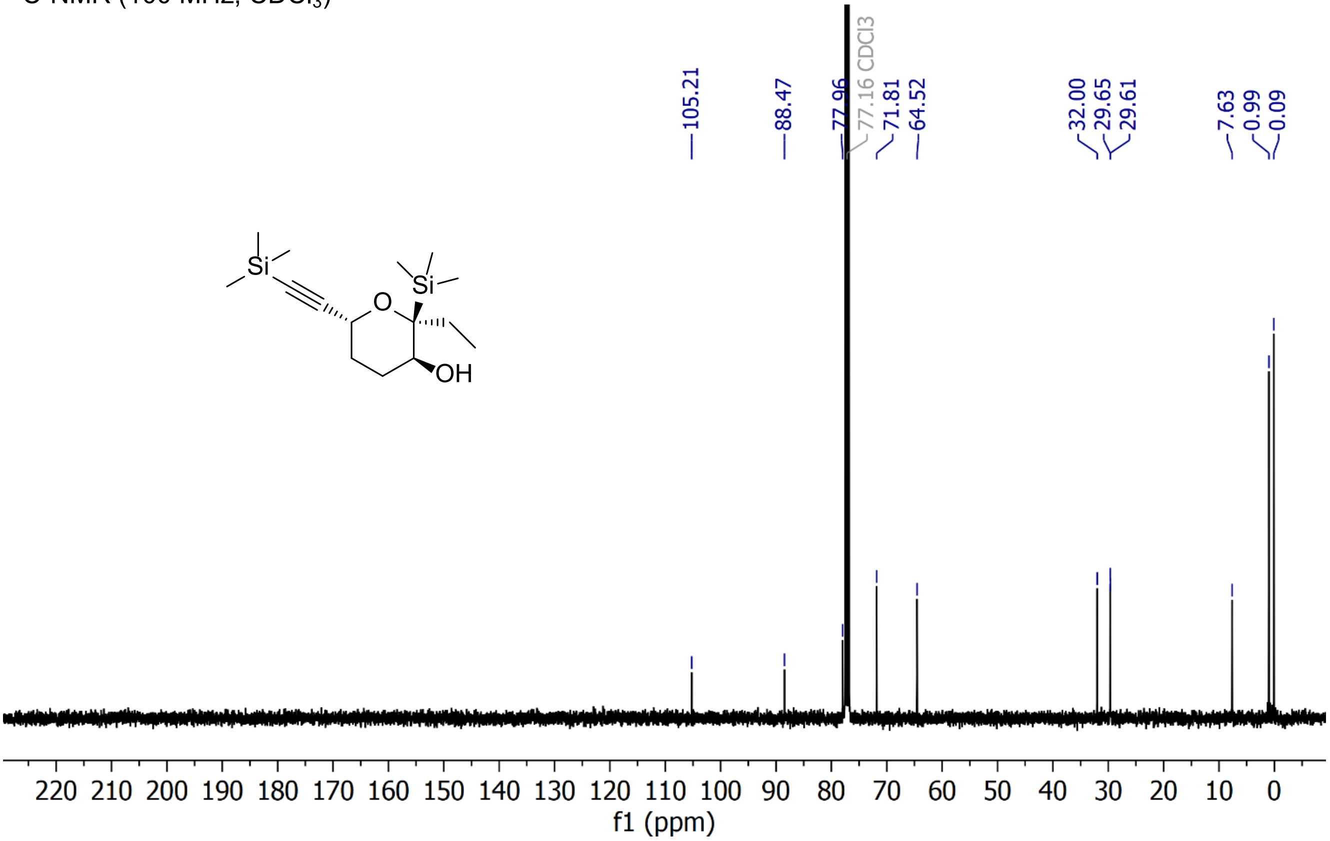

(2R,3S,6R)-2-Ethyl-6-ethynyltetrahydro-2H-pyran-3-ol 34

^{1}H-NMR (400 MHz, $CDCl_3$)

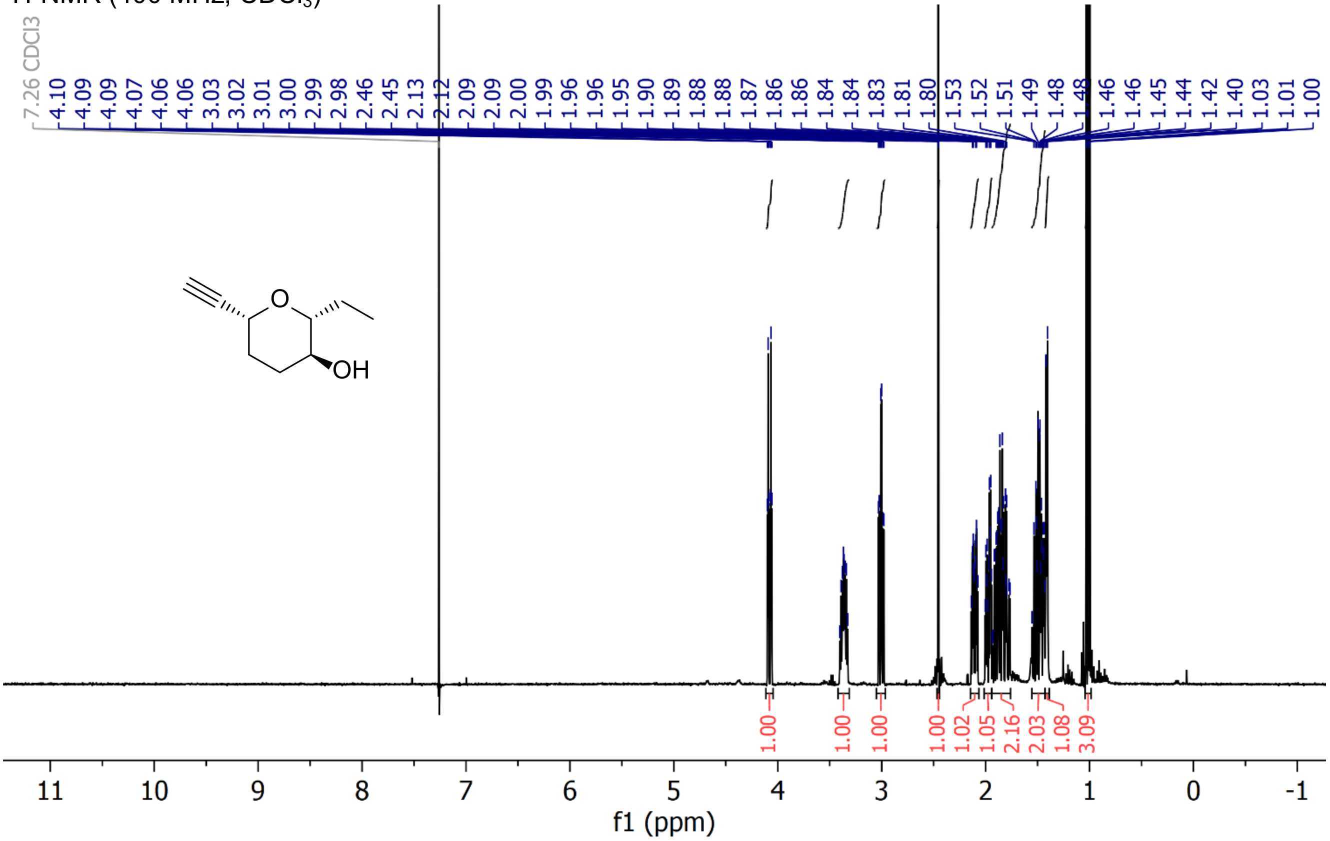

^{13}C-NMR (100 MHz, $CDCl_3$)

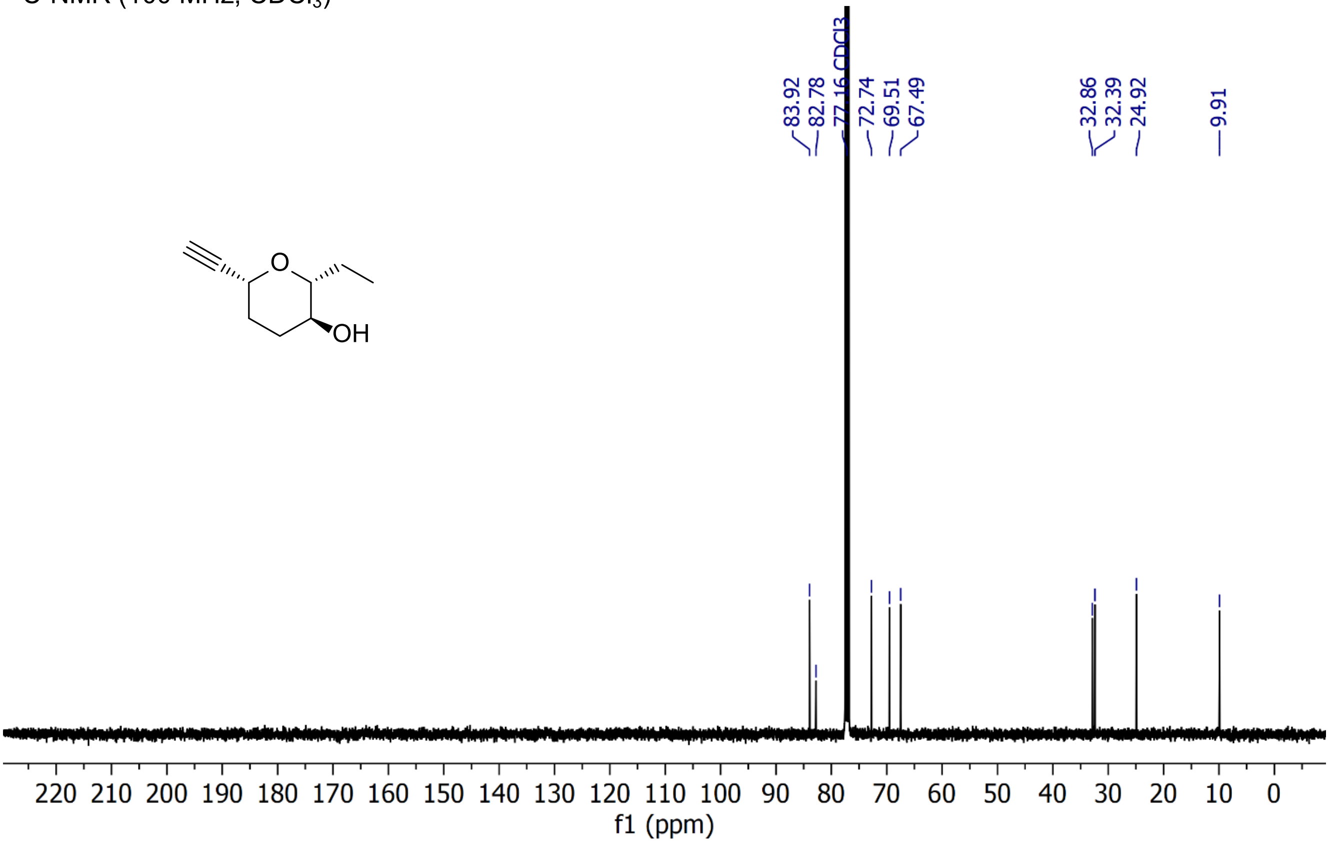

(2R,3S,6R)-2-Ethyl-6-((trimethylsilyl)ethynyl)tetrahydro-2H-pyran-3-ol 35

^{1}H-NMR (400 MHz, $CDCl_3$)

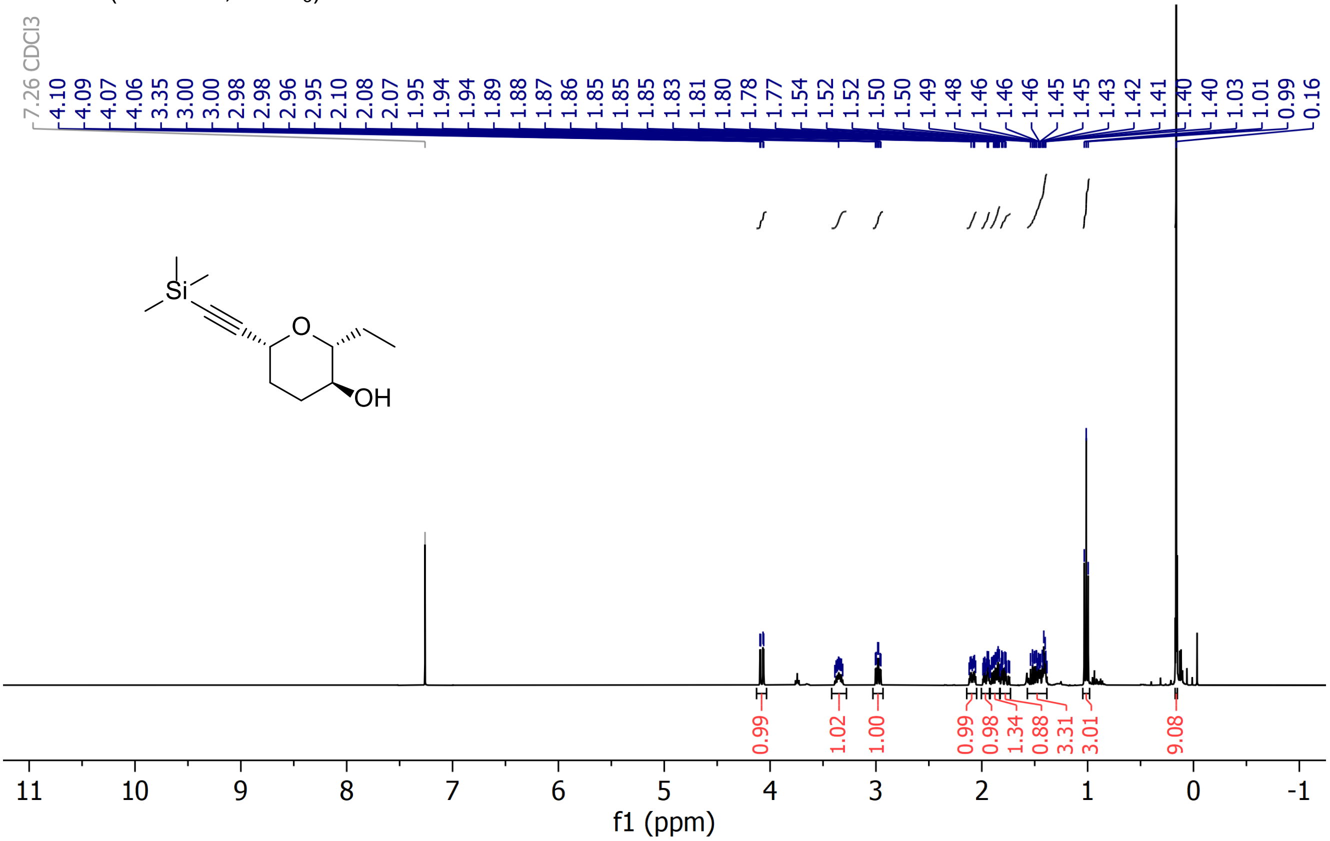

^{13}C-NMR (100 MHz, $CDCl_3$)

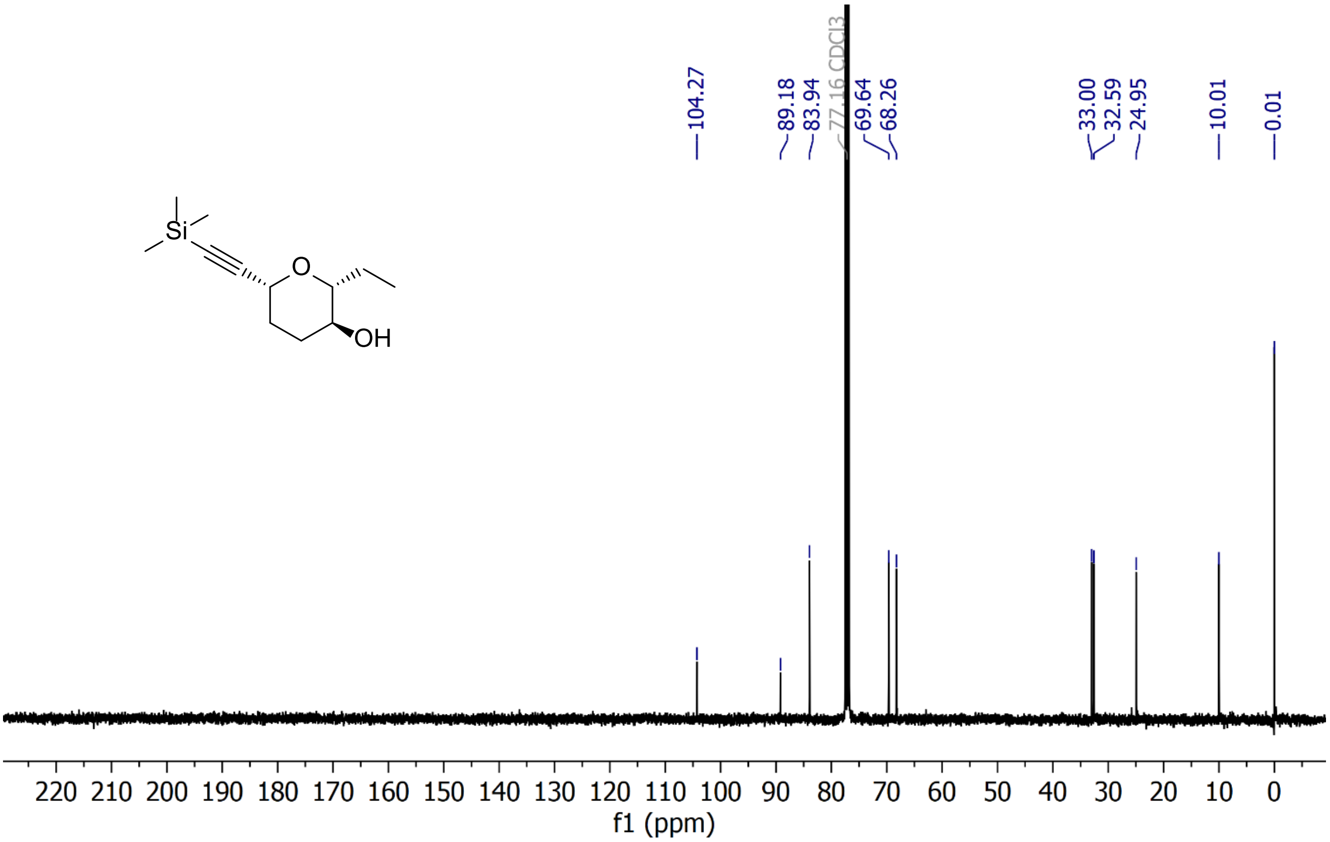

(2R,6R)-2-Ethyl-6-((trimethylsilyl)ethynyl)dihydro-2H-pyran-3(4H)-one S5

^{1}H-NMR (400 MHz, $CDCl_3$)

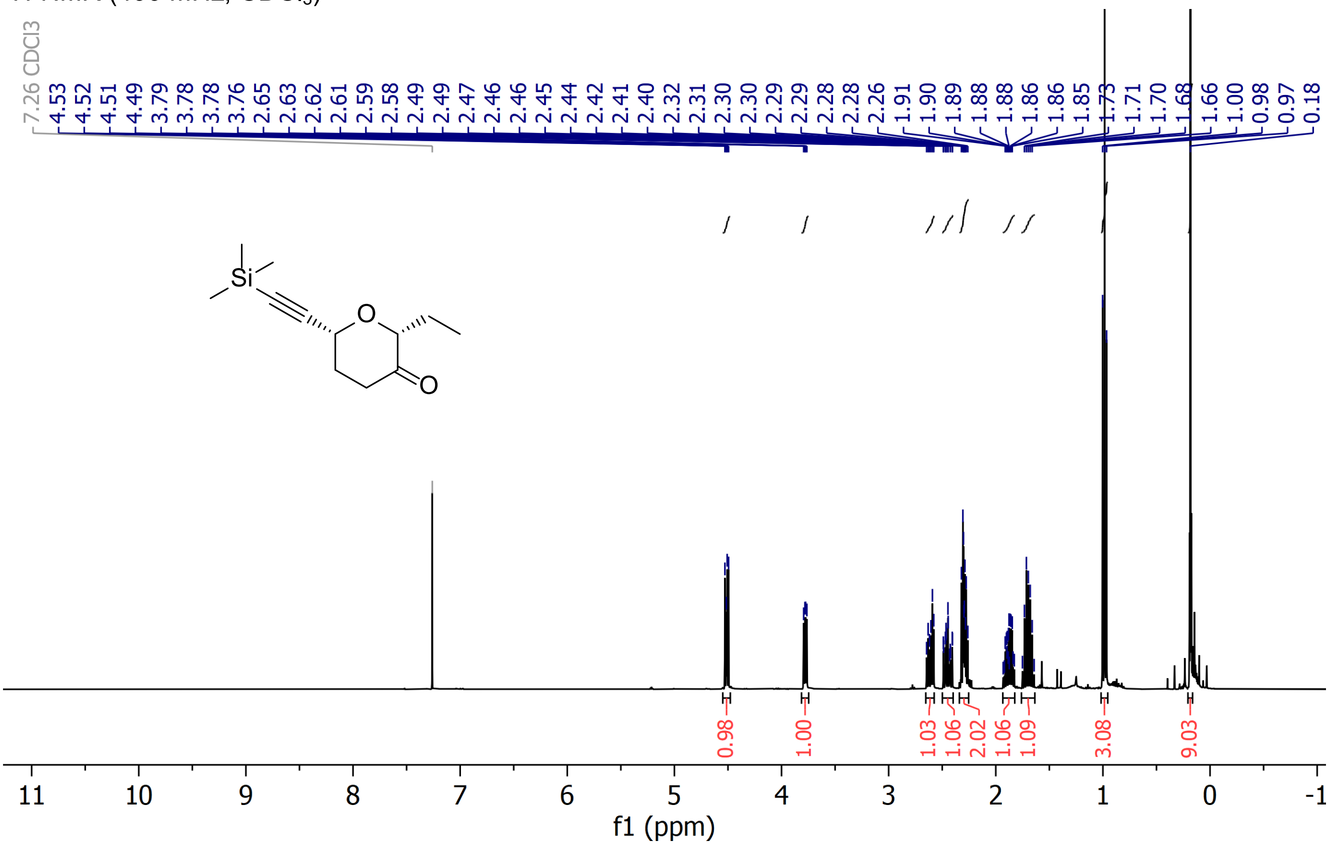

^{13}C-NMR (100 MHz, $CDCl_3$)

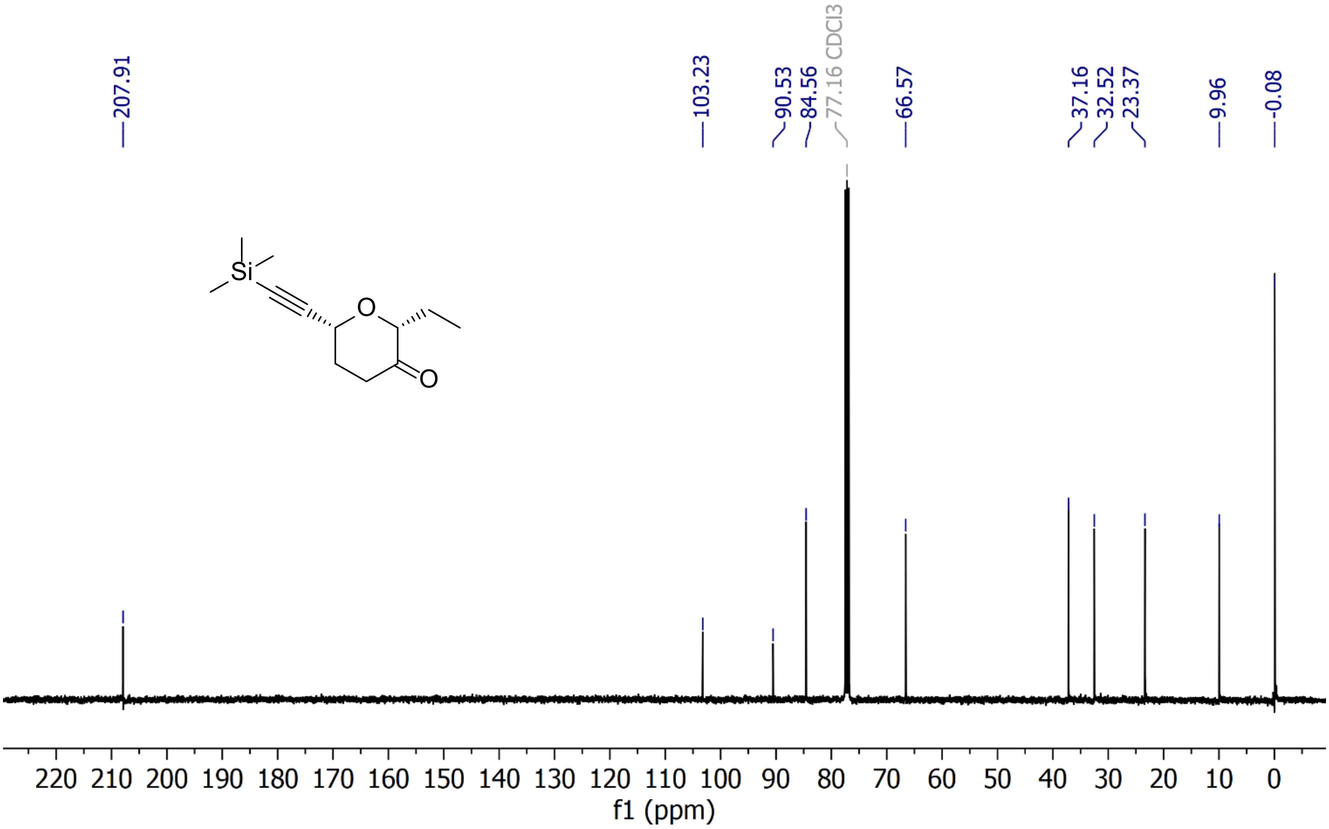

(2R,6R)-2-Ethyl-6-((trimethylsilyl)ethynyl)-5,6-dihydro-2H-pyran-3-yl trifluoromethanesulfonate 36

^{1}H-NMR (500 MHz, $CDCl_3$)

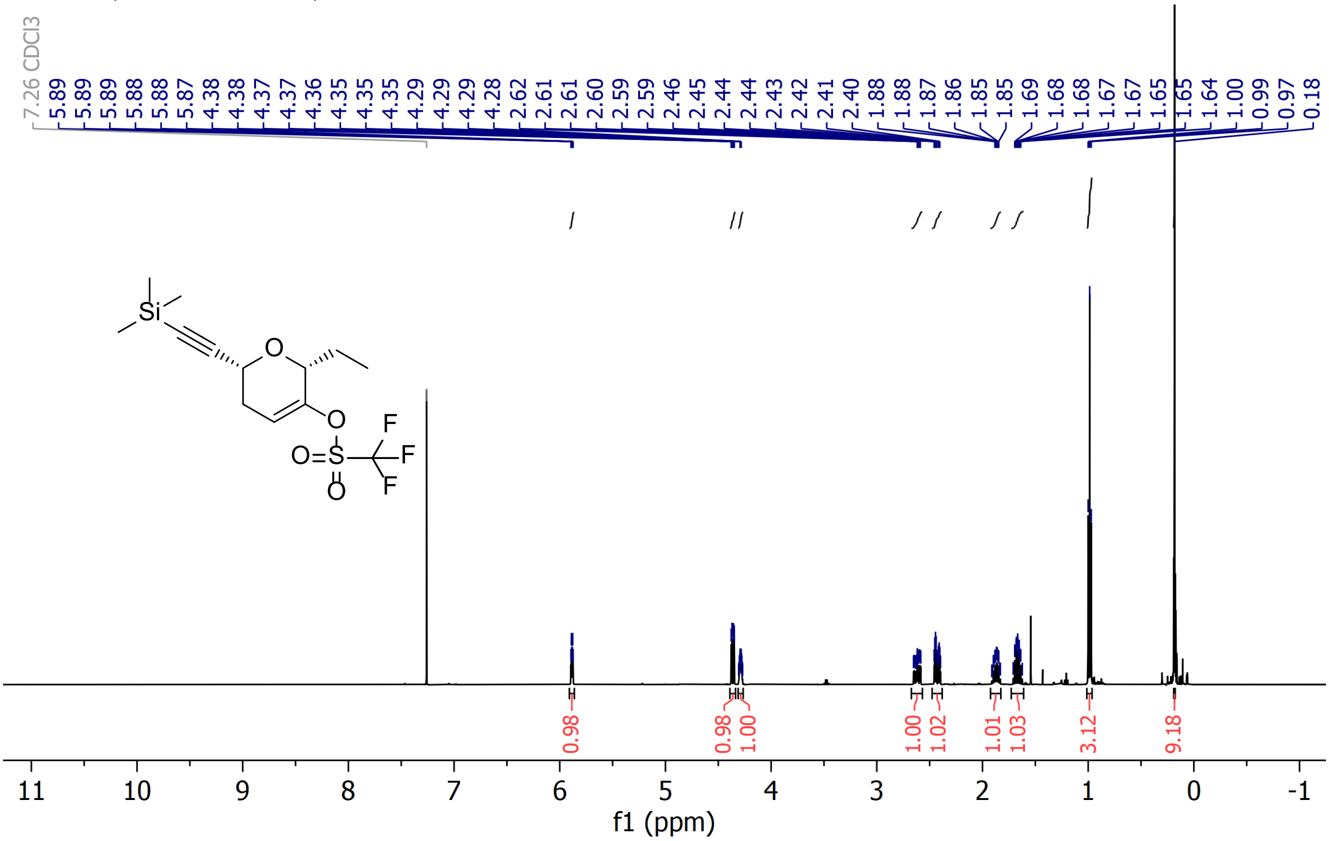

^{13}C-NMR (125 MHz, $CDCl_3$)

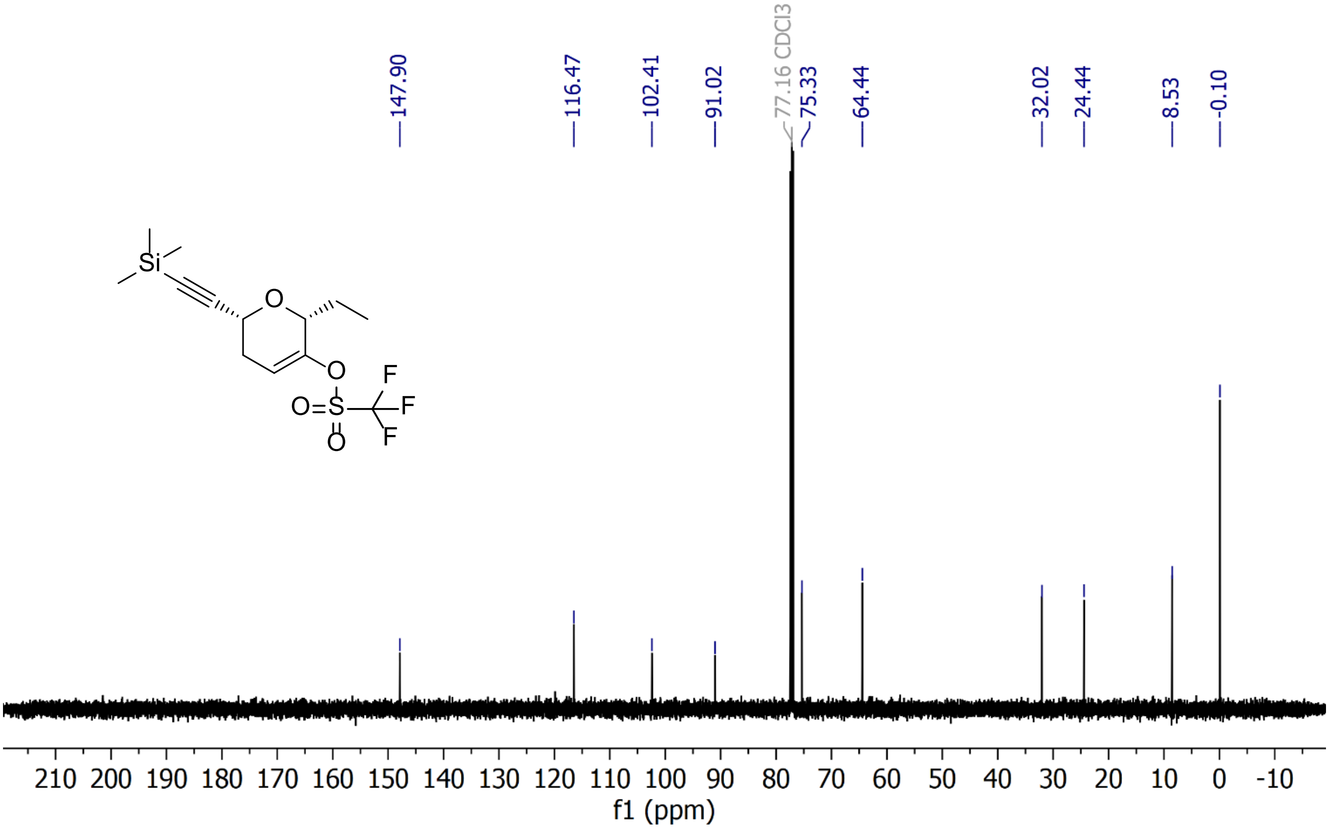

(((2R,6R)-6-Ethyl-5-methyl-3,6-dihydro-2H-pyran-2-yl)ethynyl)trimethylsilane 37

^{1}H-NMR (400 MHz, $CDCl_3$)

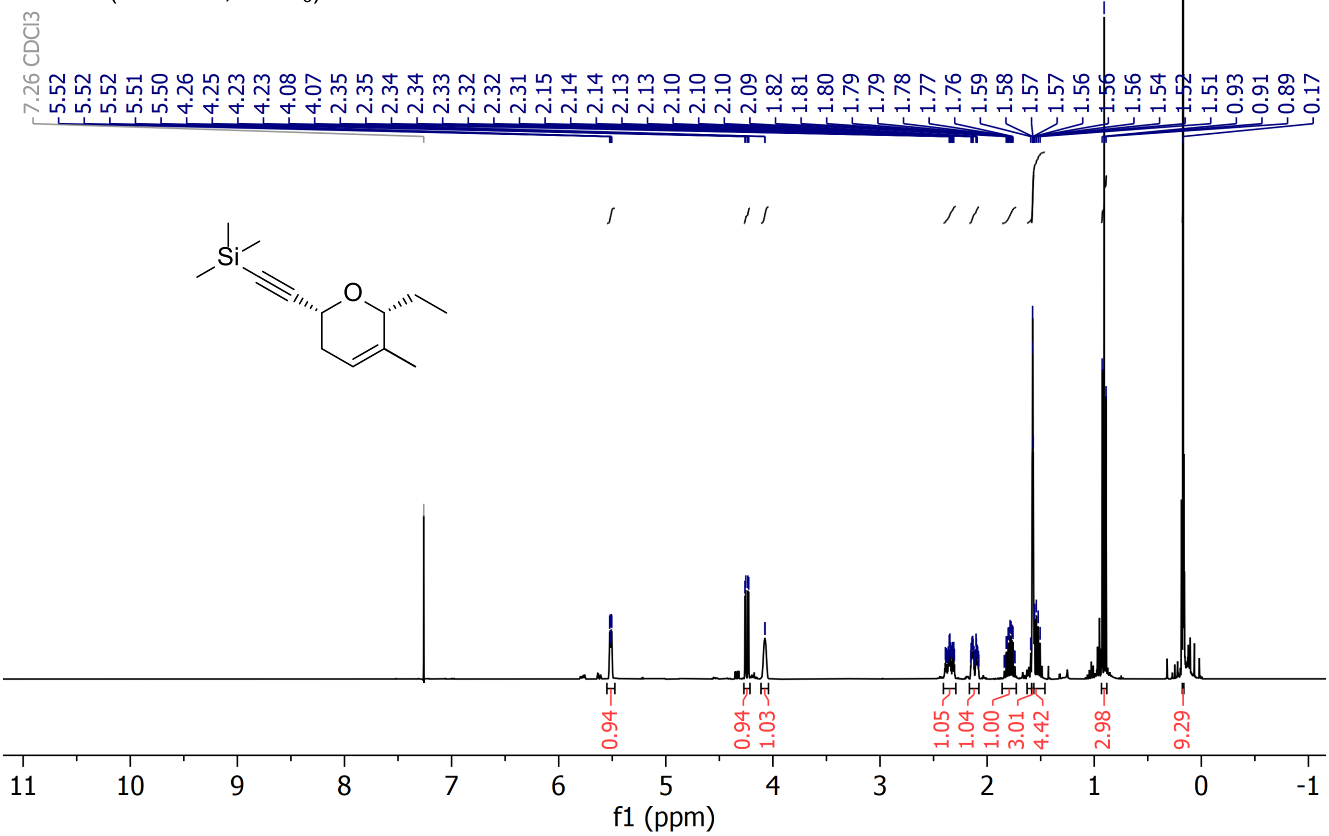

^{13}C-NMR (100 MHz, $CDCl_3$)

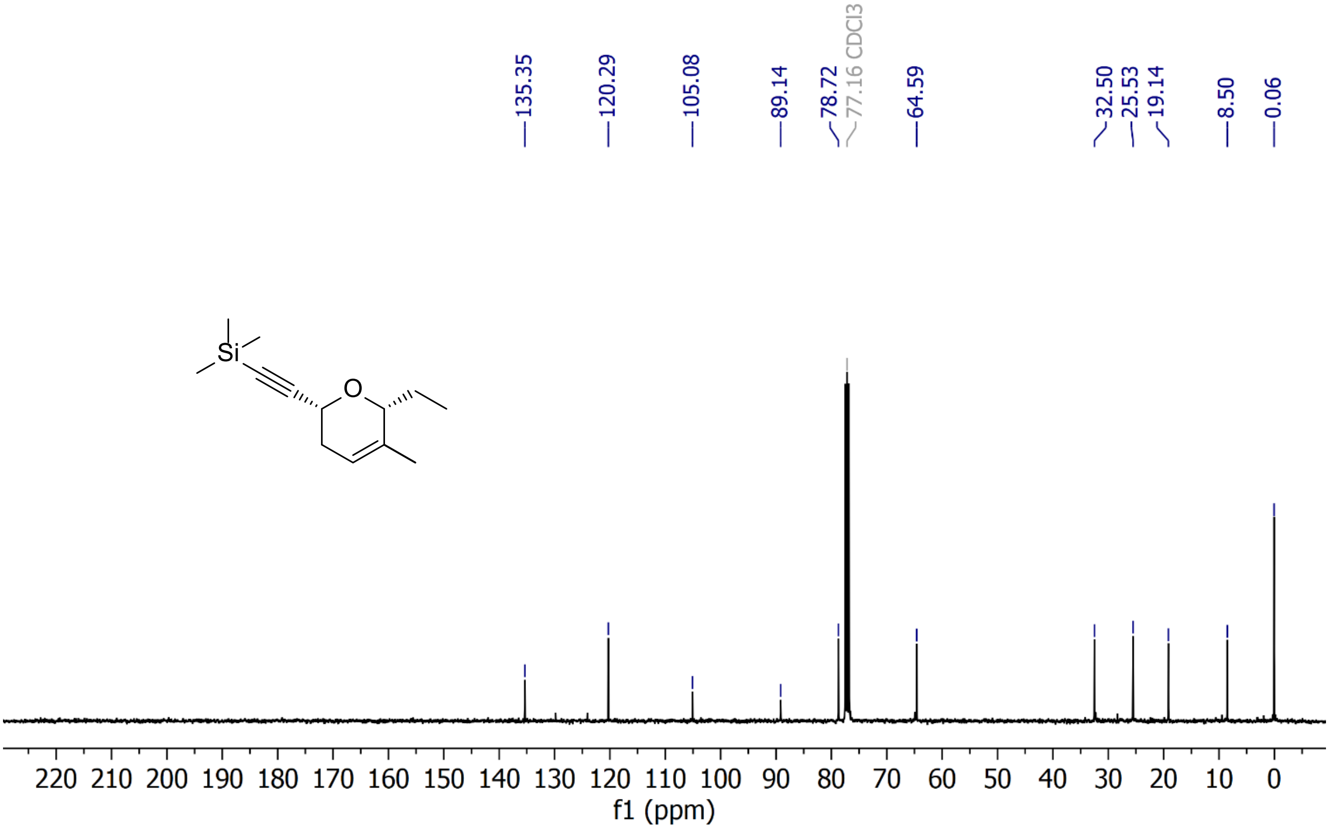

(2R,6R)-6-Ethyl-2-ethynyl-5-methyl-3,6-dihydro-2H-pyran S6

^{1}H-NMR (400 MHz, $CDCl_3$)

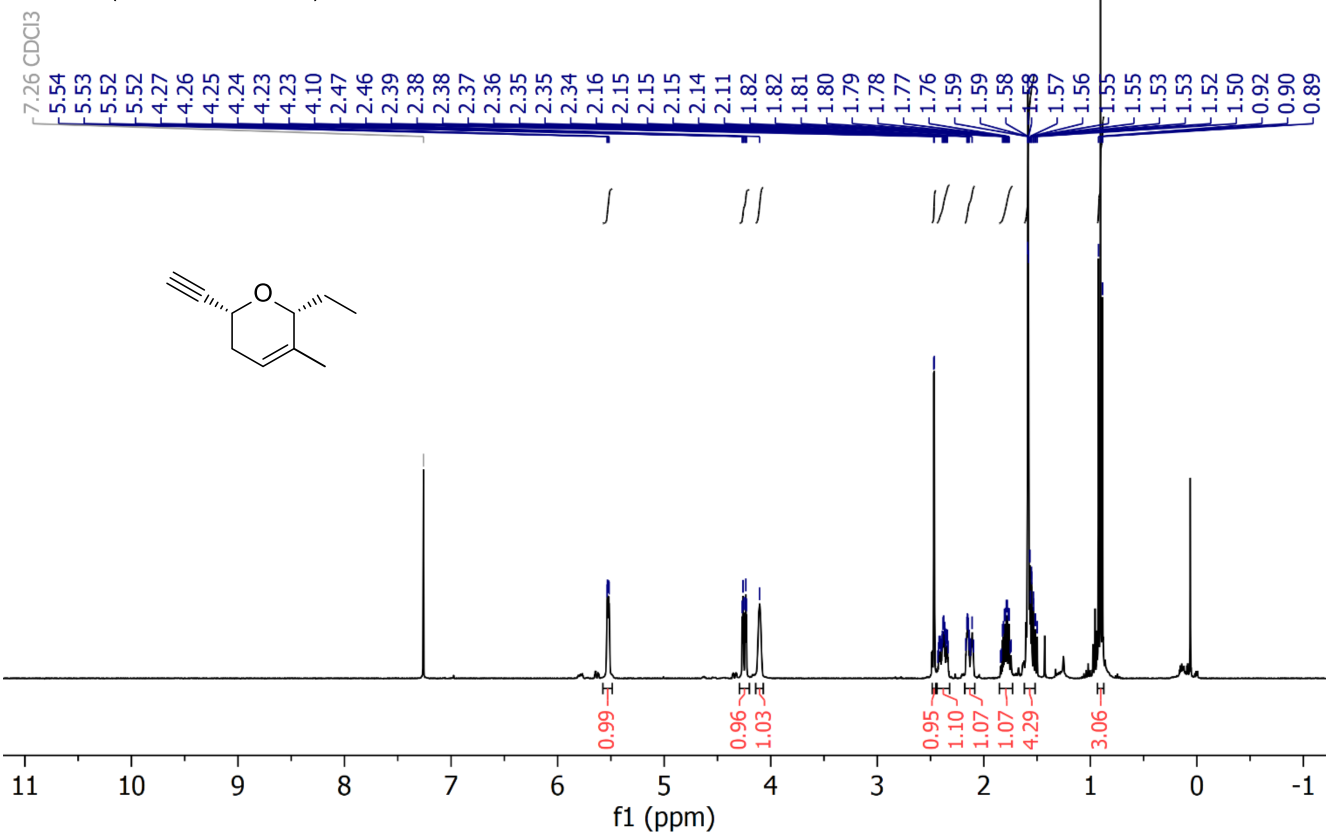

^{13}C-NMR (100 MHz, $CDCl_3$)

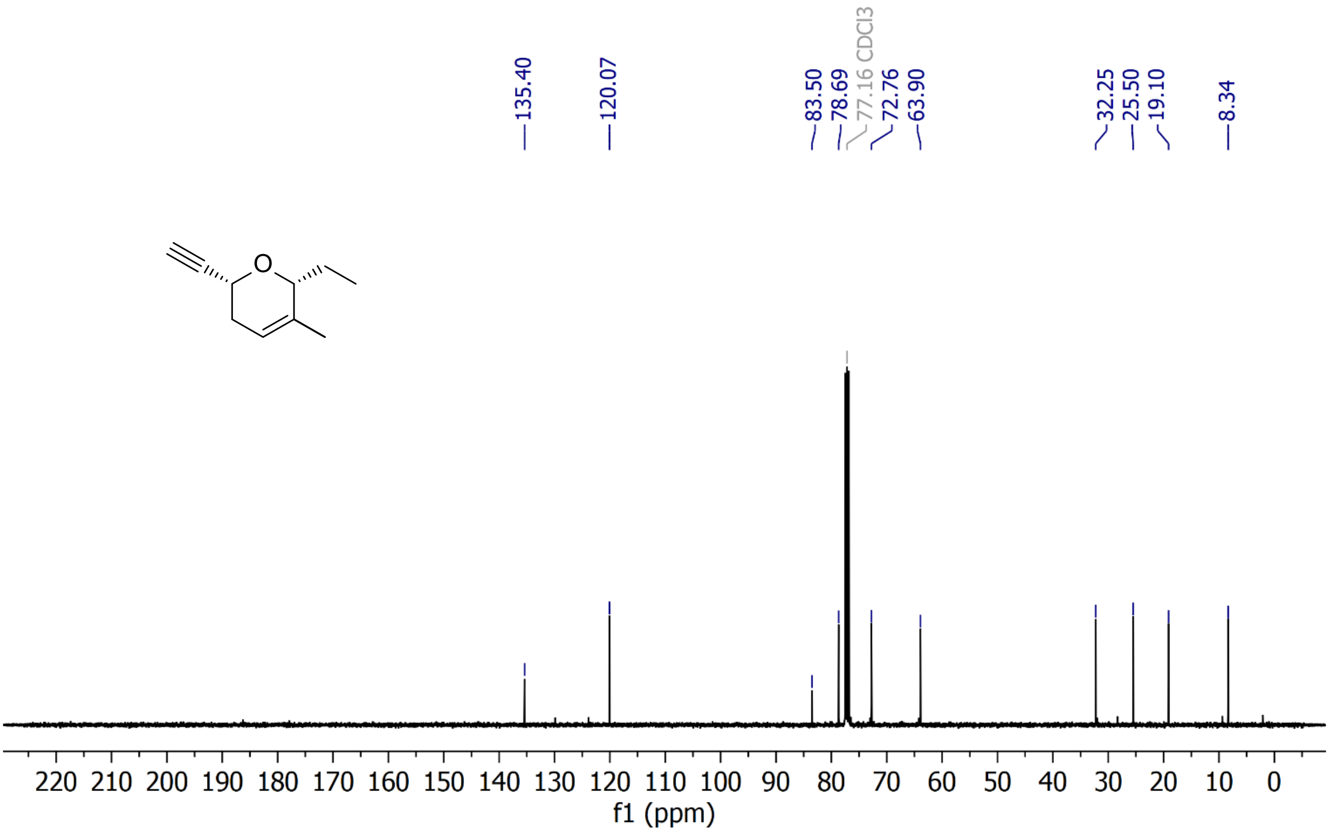

Tributyl((E)-2-((2R,6R)-6-ethyl-5-methyl-3,6-dihydro-2H-pyran-2-yl)prop-1-en-1-yl)stannane 38

^{1}H-NMR (400 MHz, $CDCl_3$)

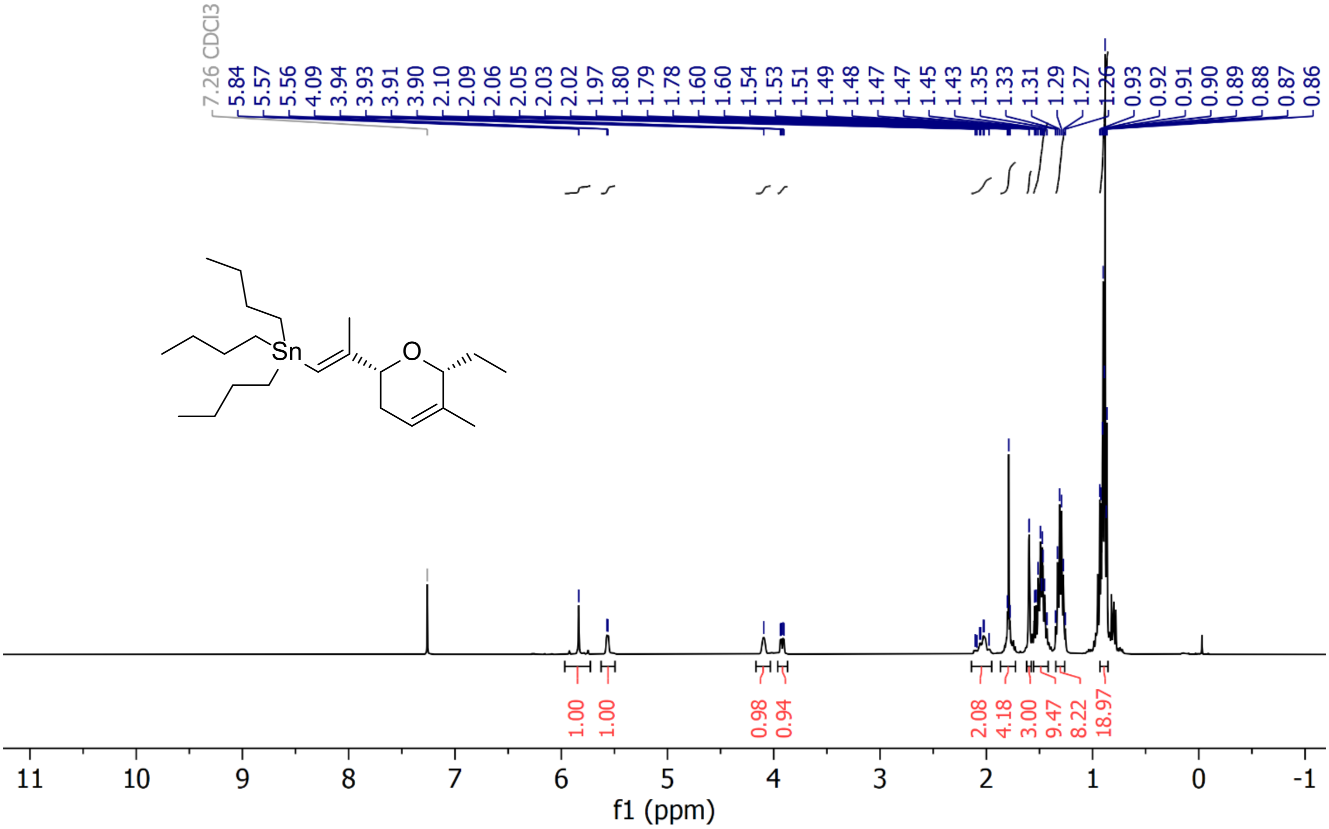

^{13}C-NMR (100 MHz, $CDCl_3$)

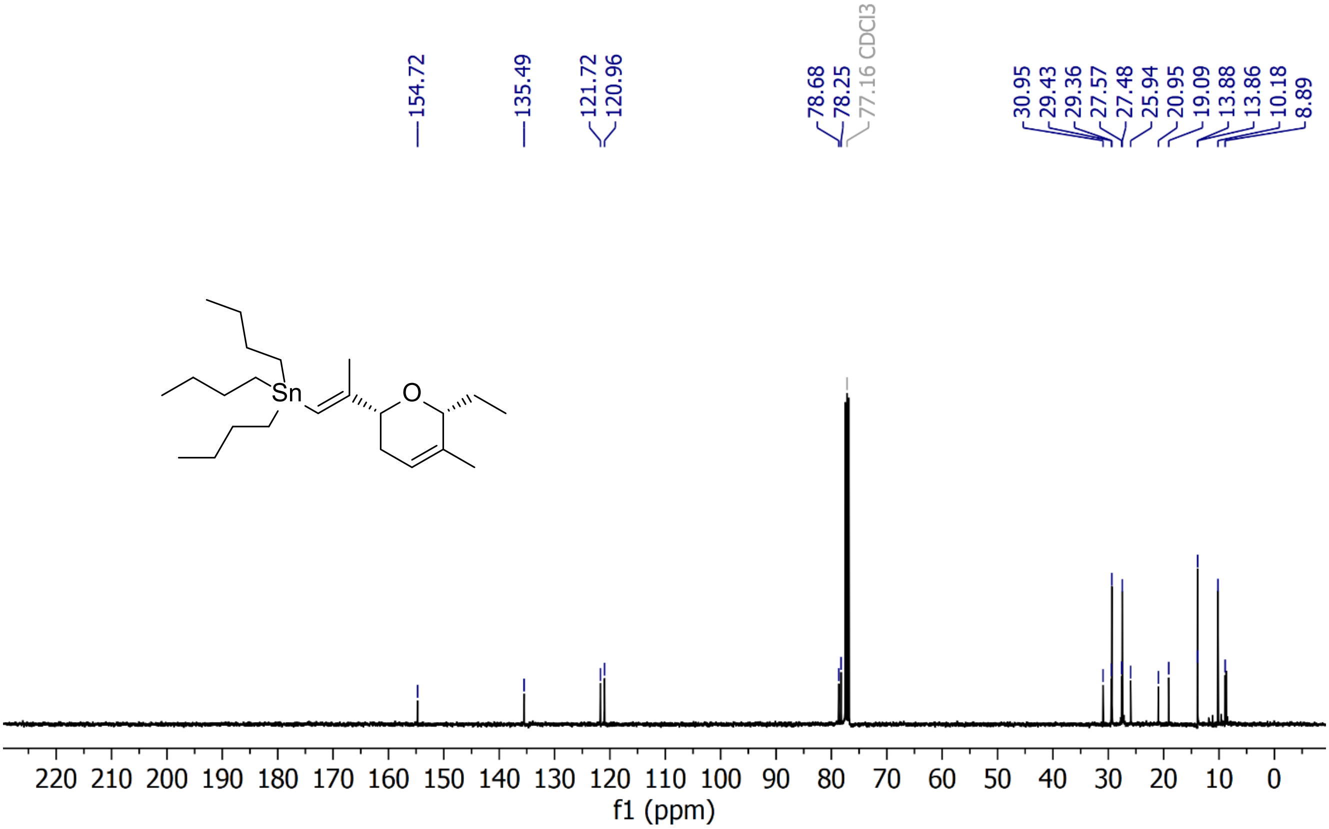

(2R,6R)-2-ethyl-6-ethynyldihydro-2H-pyran-3(4H)-one 39

^{1}H-NMR (400 MHz, $CDCl_3$)

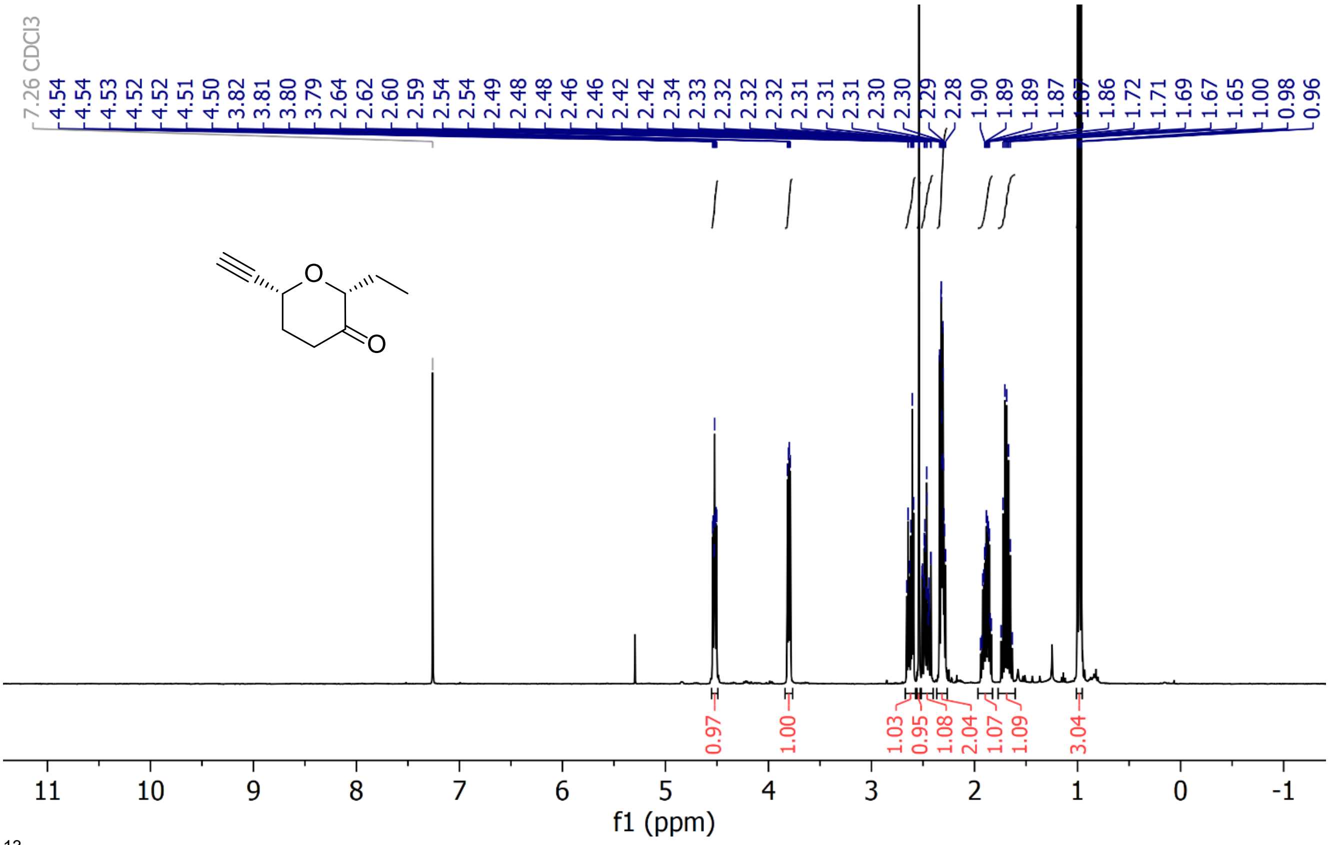

^{13}C-NMR (100 MHz, $CDCl_3$)

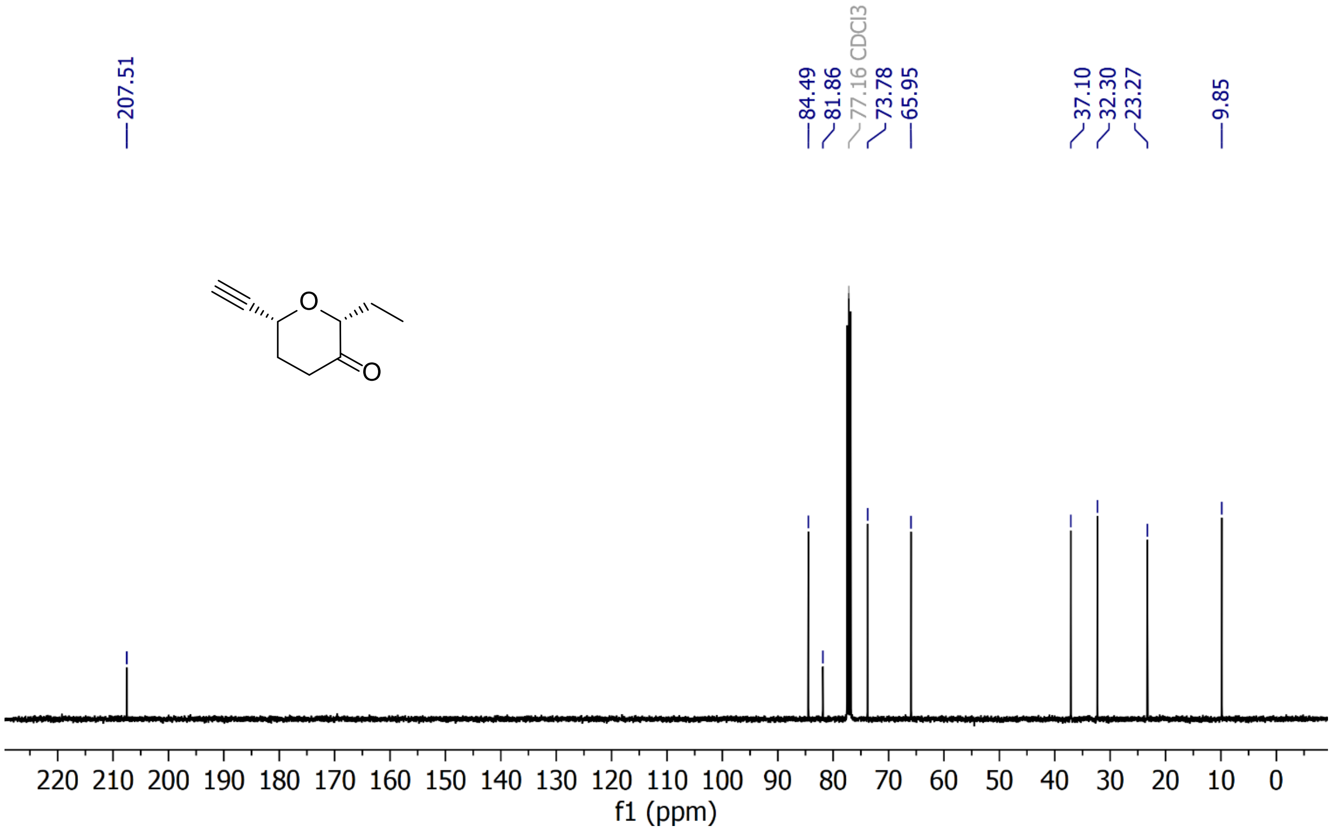

(2R,3R,6R)-2-Ethyl-6-ethynyl-3-methyltetrahydro-2H-pyran-3-ol 40

^{1}H-NMR (500 MHz, $CDCl_3$)

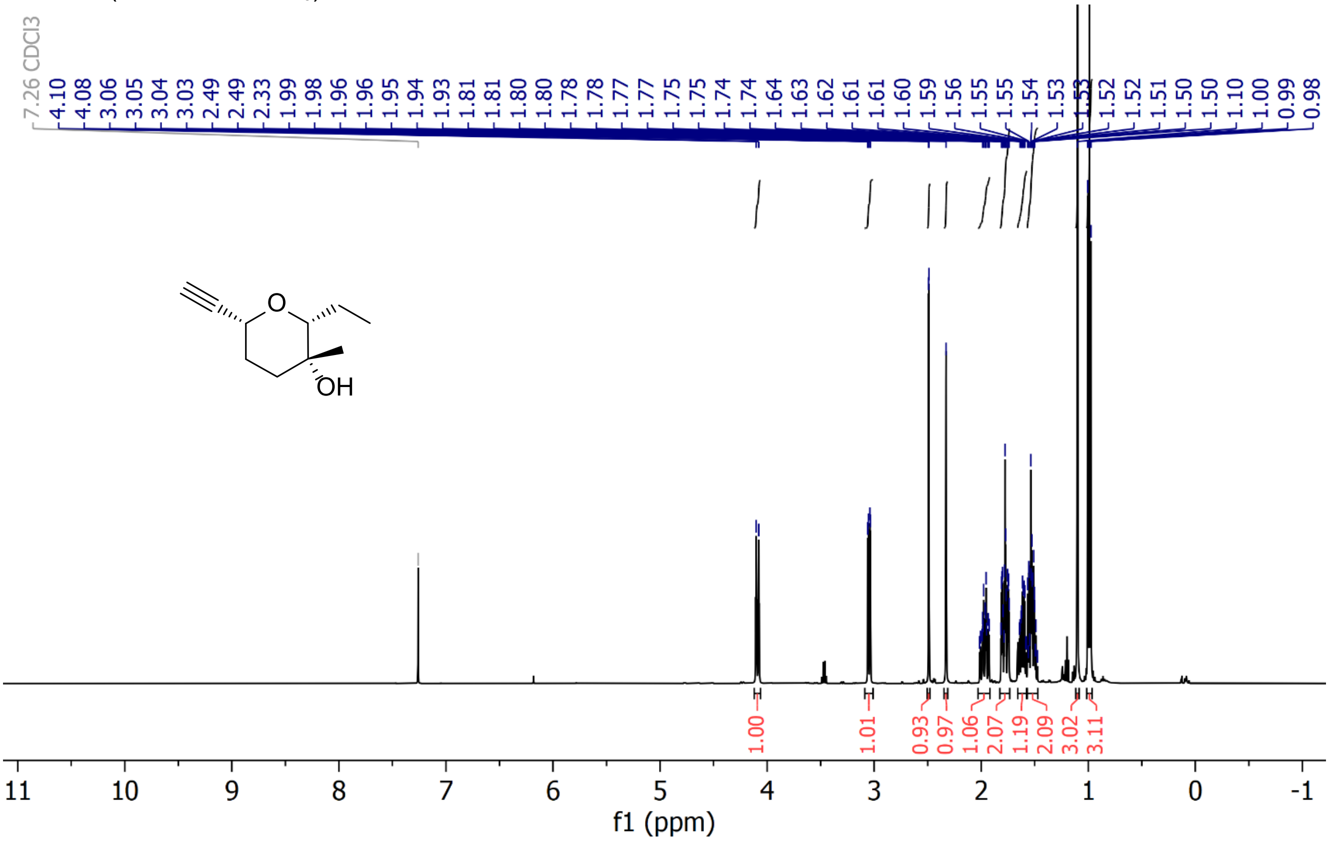

^{13}C-NMR (125 MHz, $CDCl_3$)

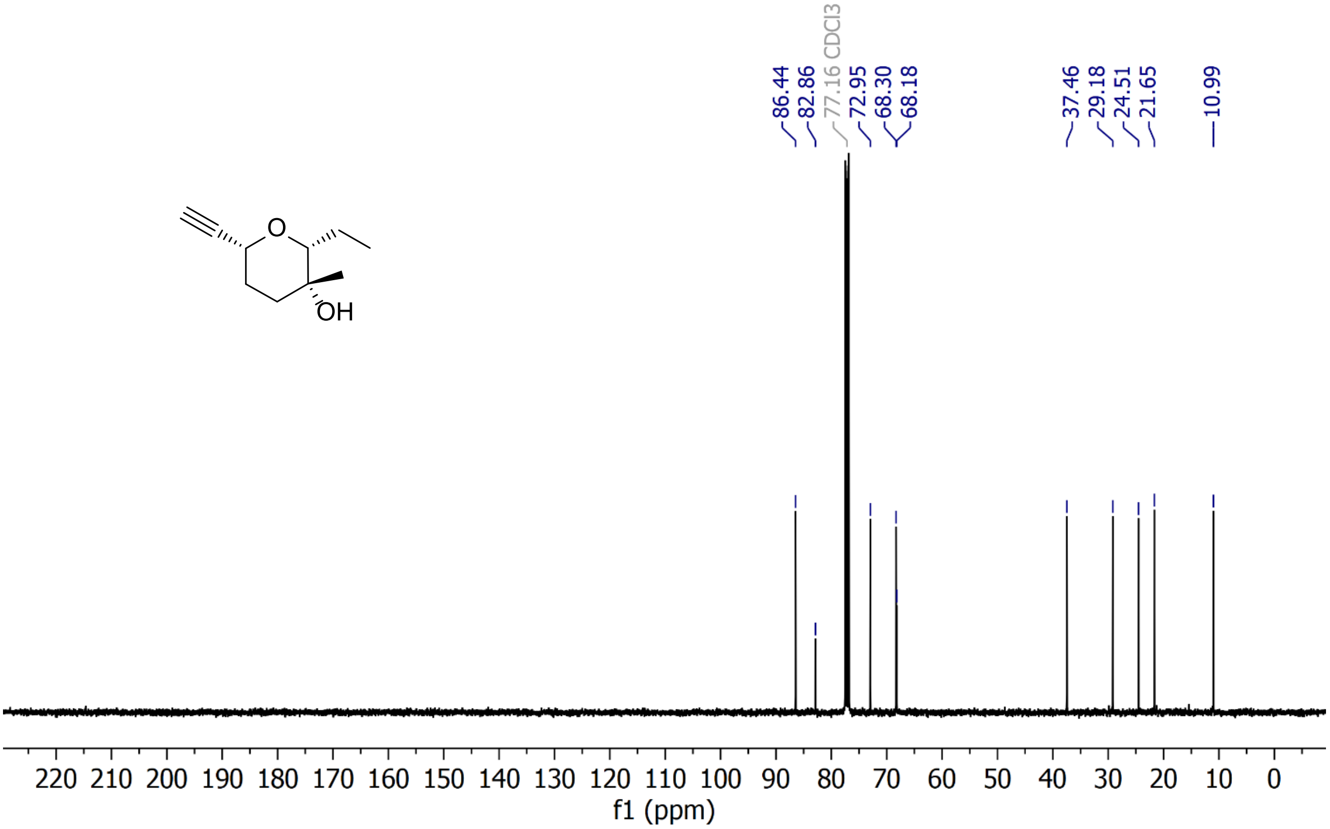

(2R,3S,6R)-2-ethyl-6-ethynyl-3-methyltetrahydro-2H-pyran-3-ol 40

^{1}H-NMR (400 MHz, $CDCl_3$)

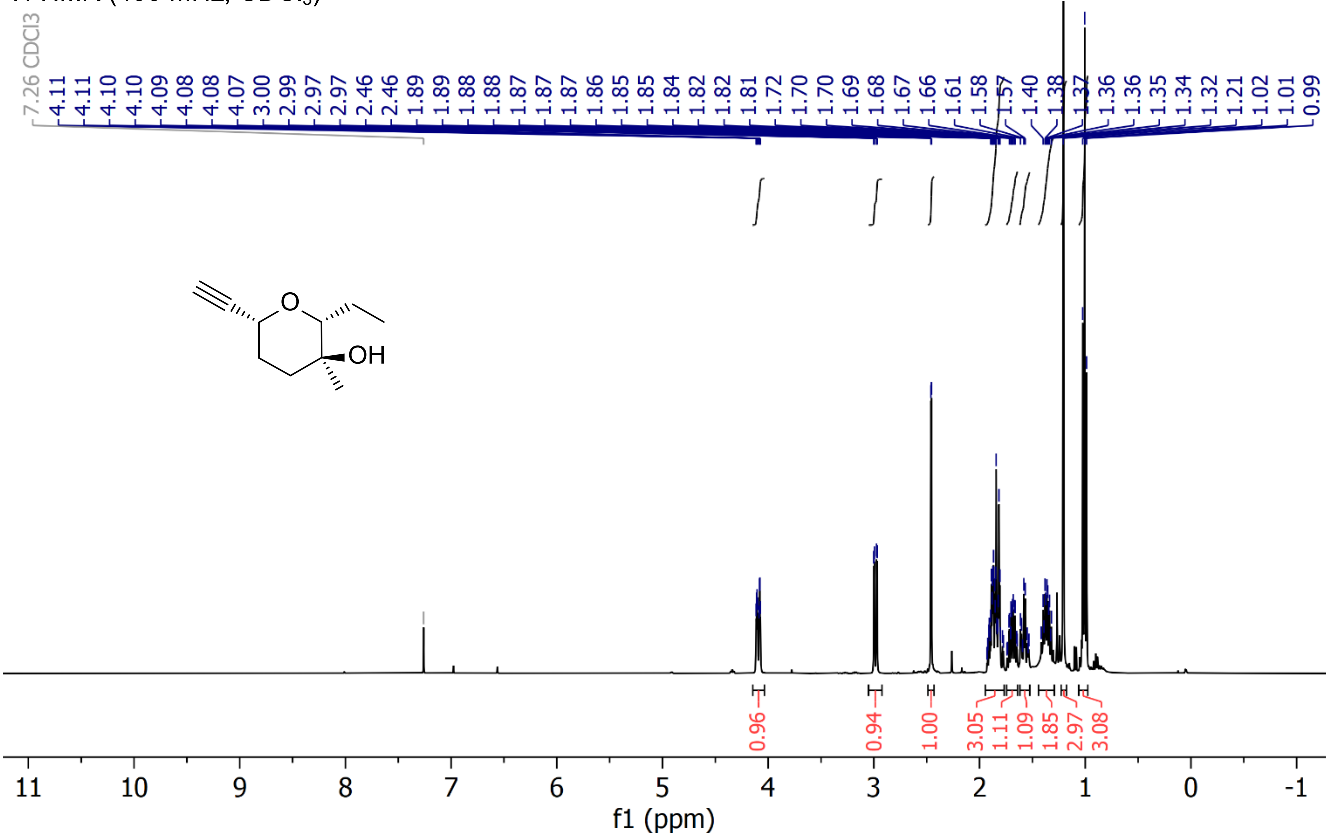

^{13}C-NMR (100 MHz, $CDCl_3$)

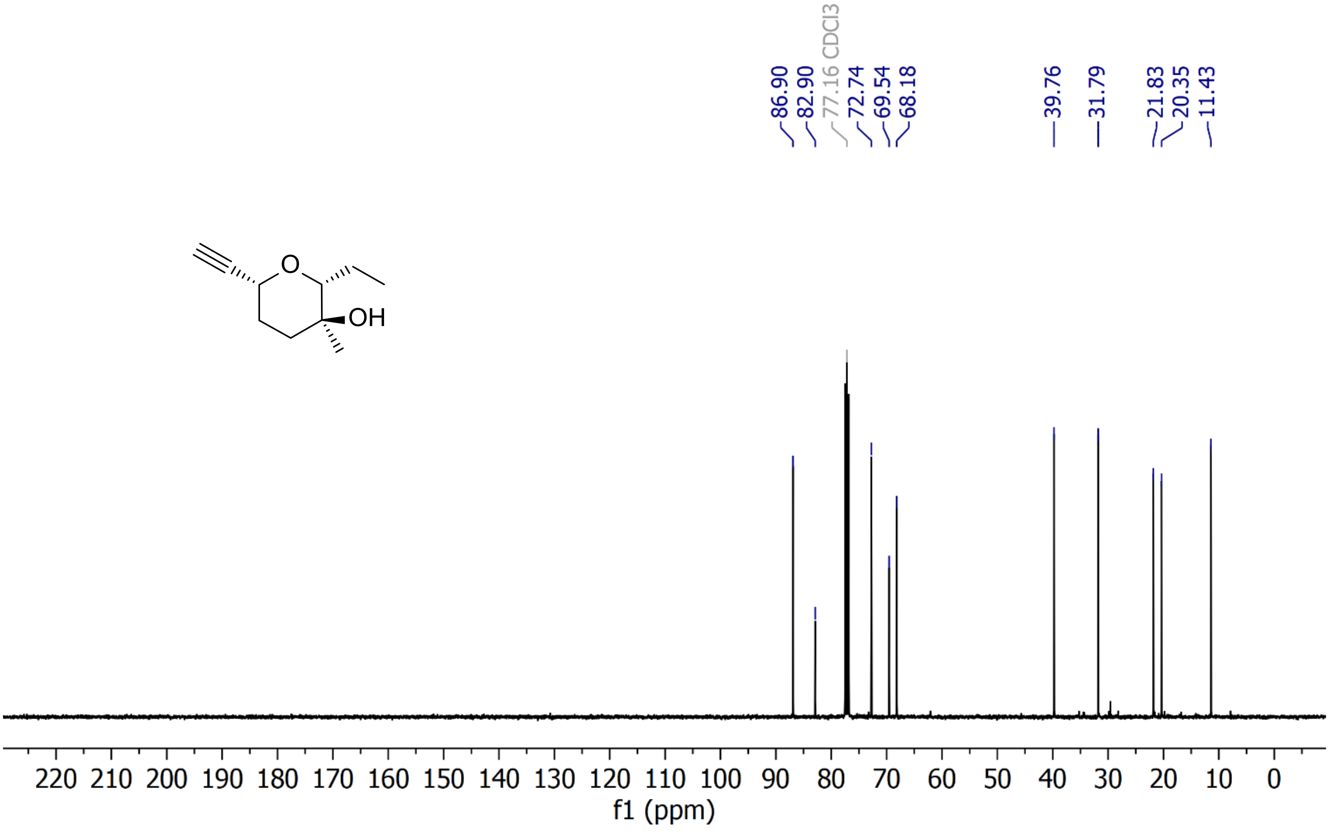

(2R,3R,6R)-2-Ethyl-6-ethynyl-3-methyltetrahydro-2H-pyran 43

^{1}H-NMR (400 MHz, $CDCl_3$)

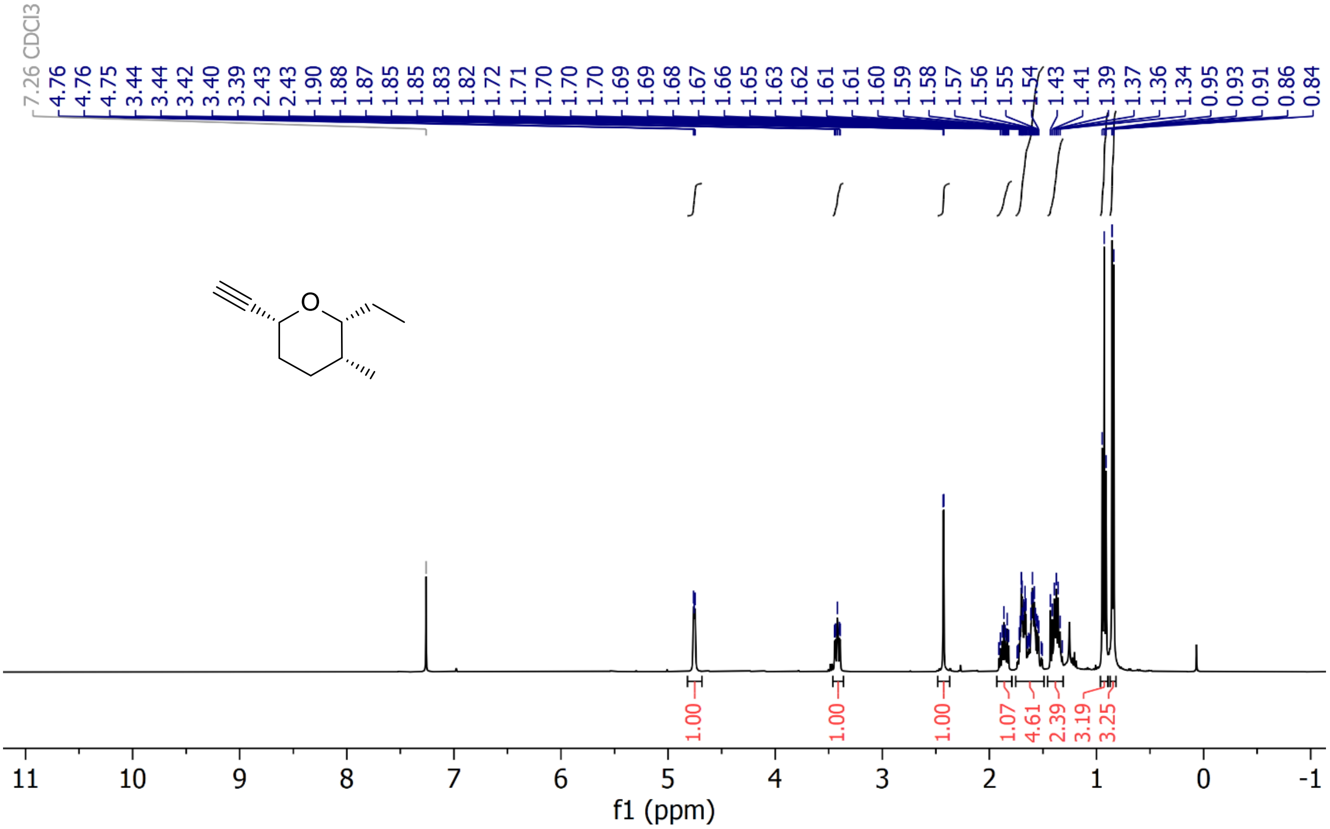

^{13}C-NMR (100 MHz, $CDCl_3$)

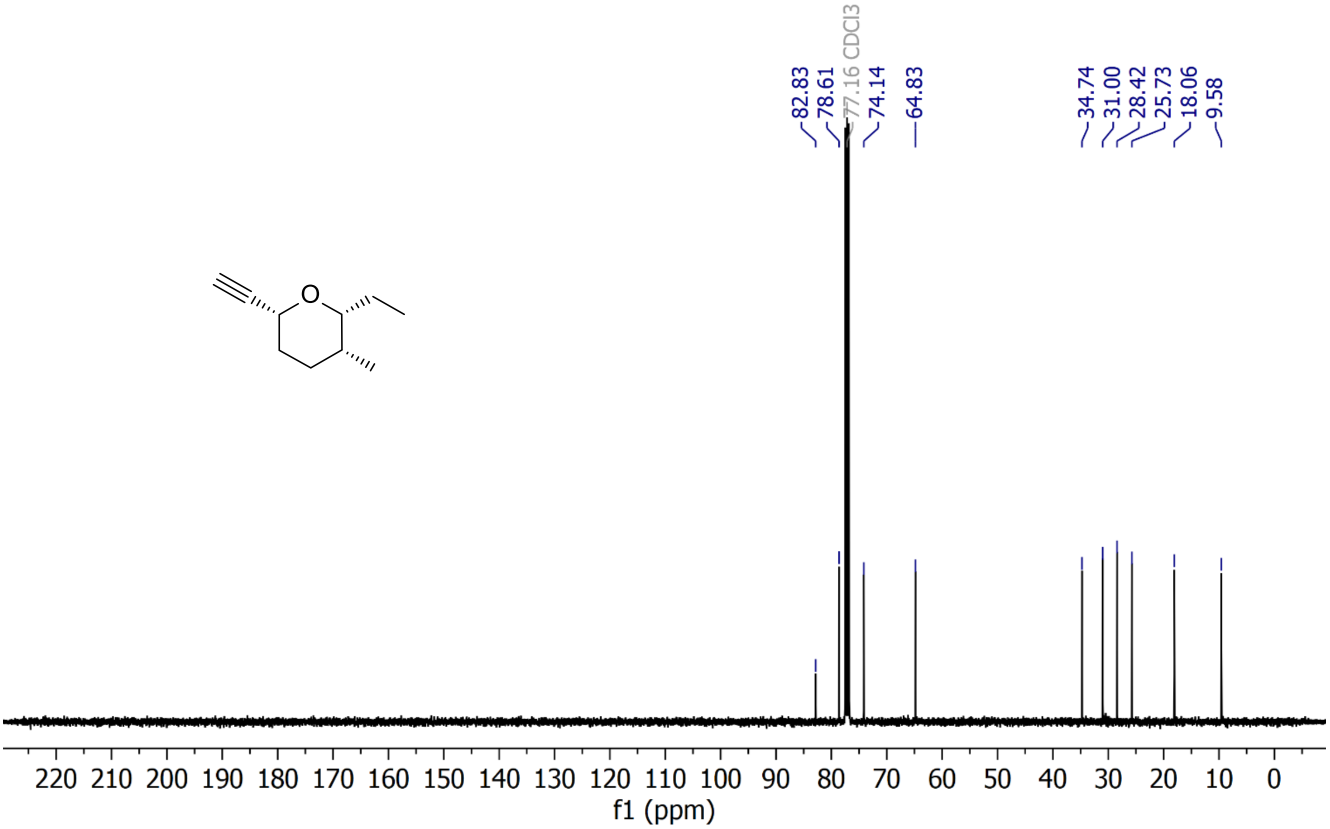

(2S,3R,6R)-2-Ethyl-6-ethynyl-3-methyltetrahydro-2H-pyran 44

^{1}H-NMR (400 MHz, $CDCl_3$)

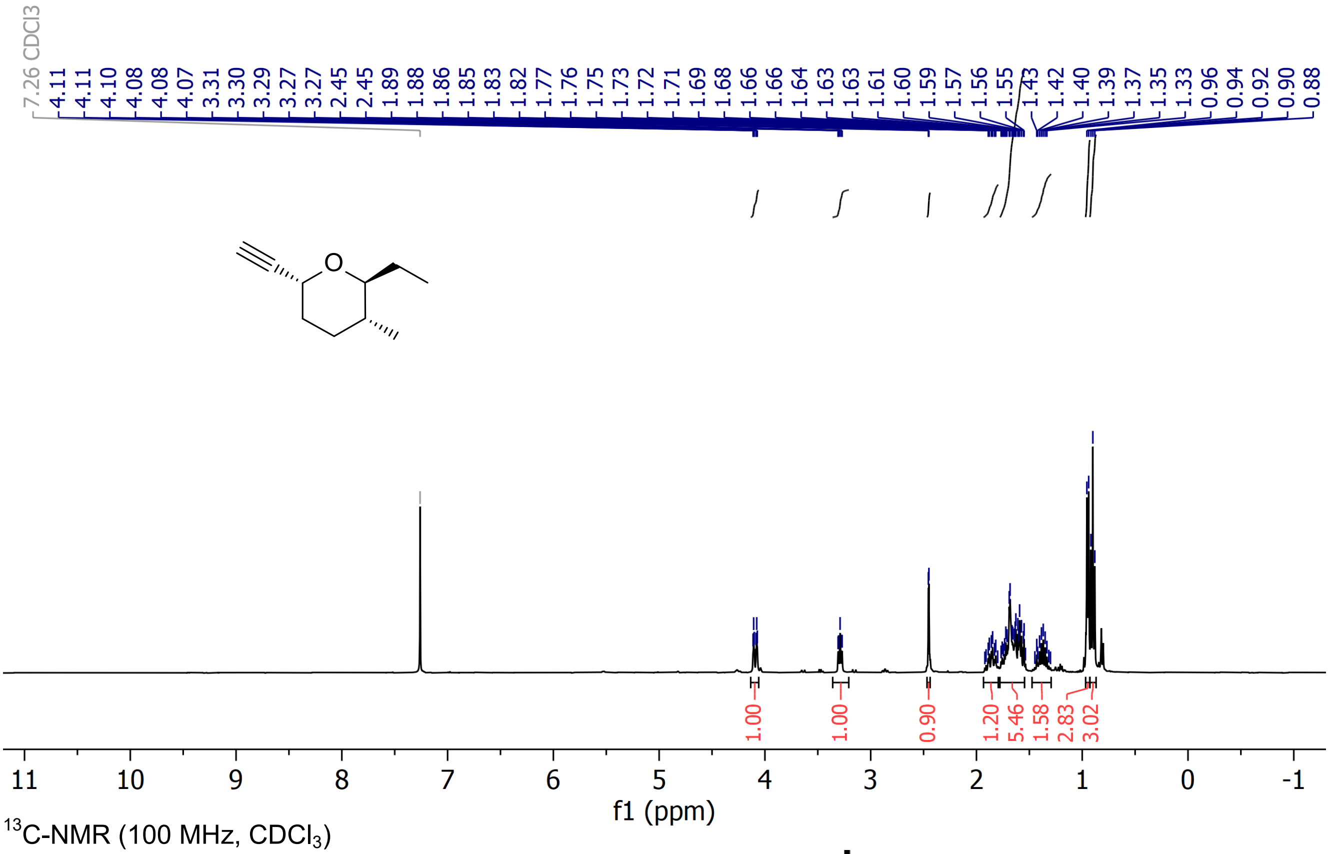

^{13}C-NMR (100 MHz, $CDCl_3$)

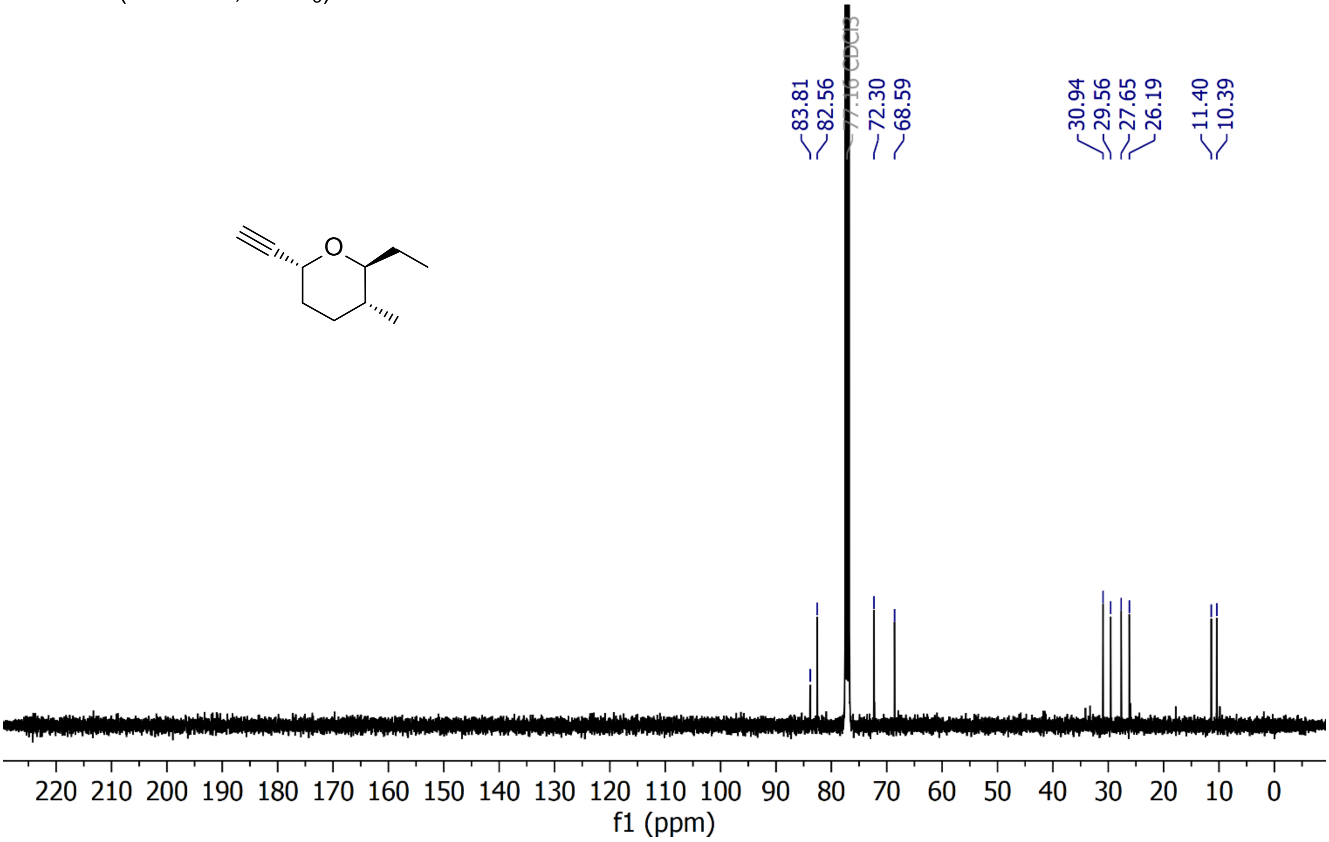

Tributyl((E)-2-((2R,5R,6R)-6-ethyl-5-methyltetrahydro-2H-pyran-2-yl)prop-1-en-1-yl)stannane 46

^{1}H-NMR (400 MHz, $CDCl_3$)

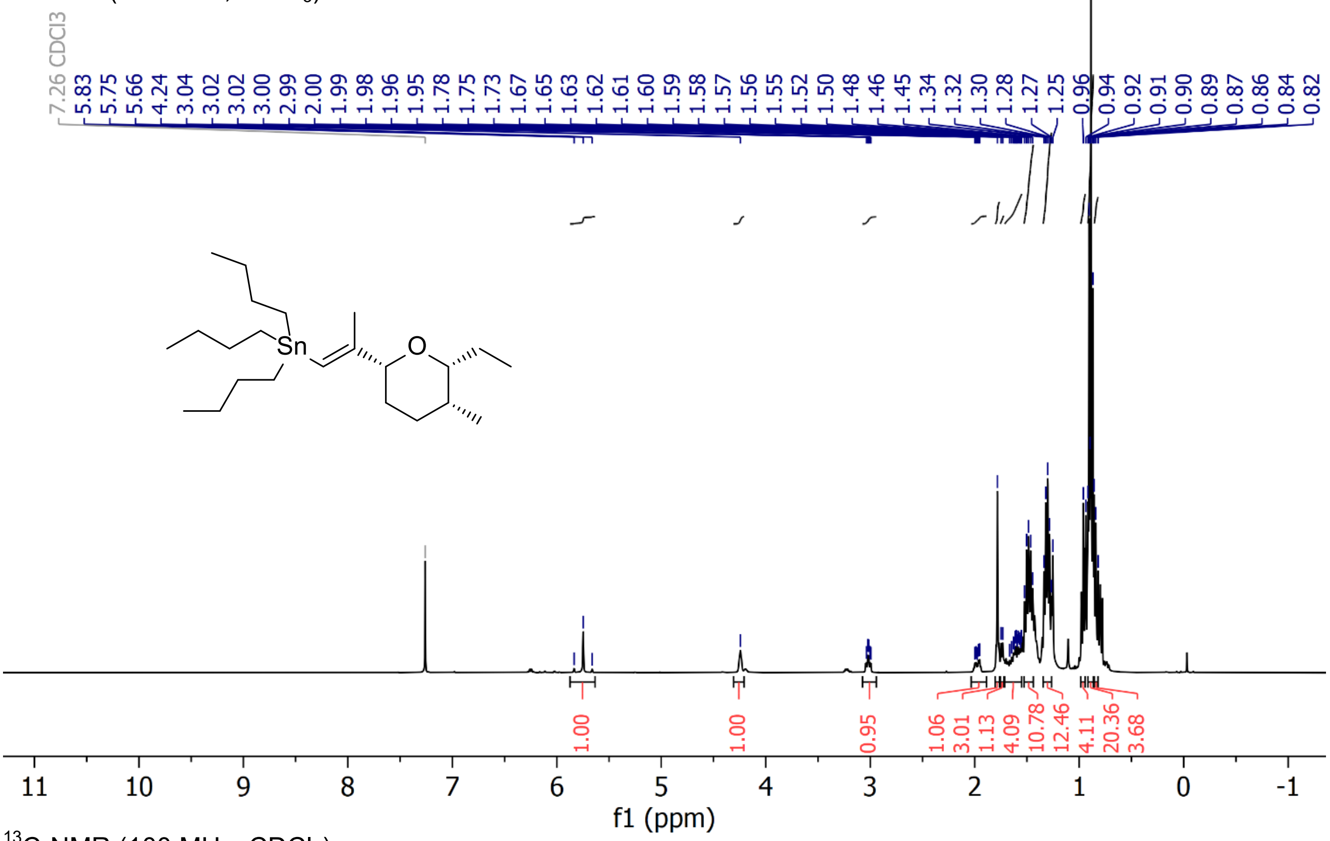

^{13}C-NMR (100 MHz, $CDCl_3$)

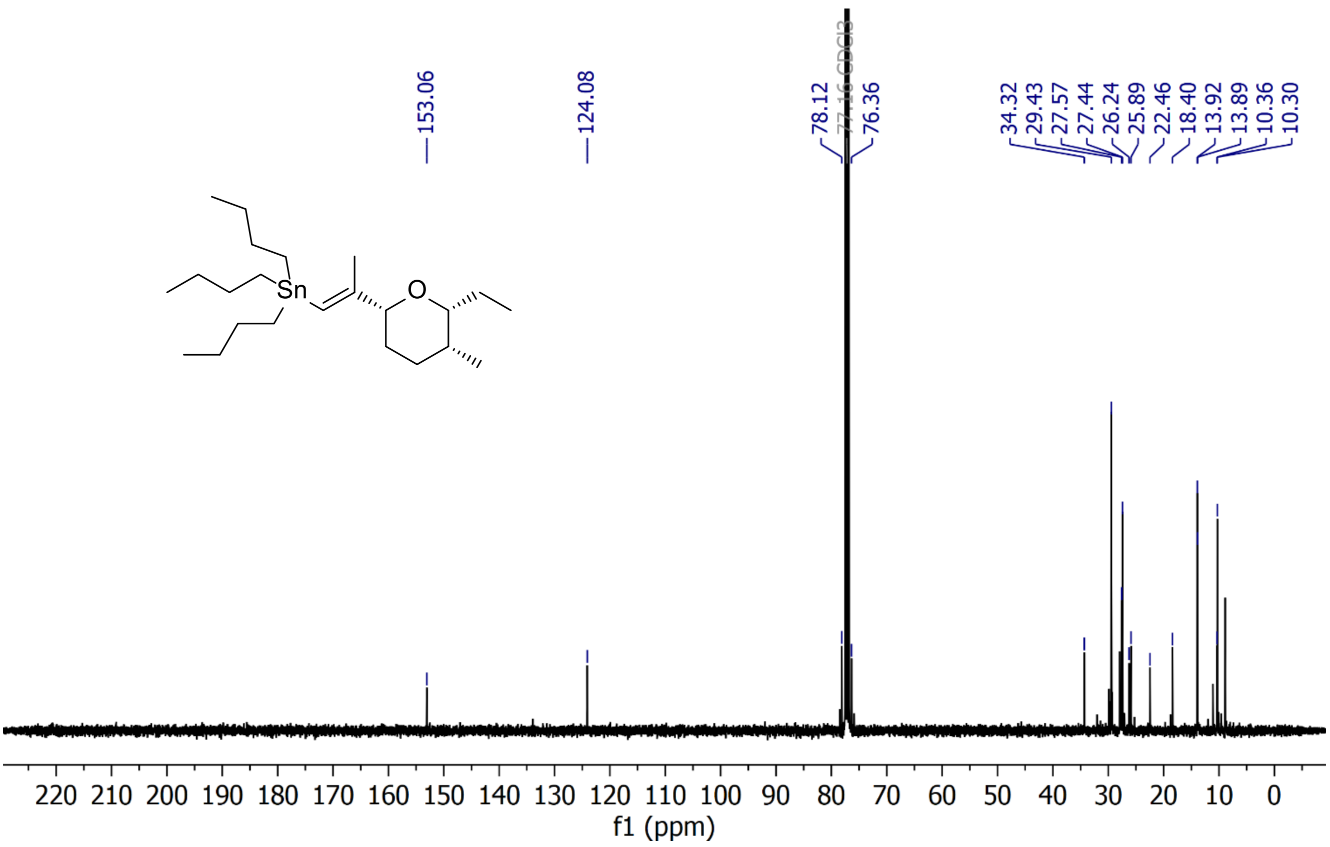

(((1S,2R,4R,6S)-2-Ethyl-1-methyl-3,7-dioxabicyclo[4.1.0]heptan-4-yl)ethynyl)trimethylsilane 47

^{1}H-NMR (400 MHz, $CDCl_3$)

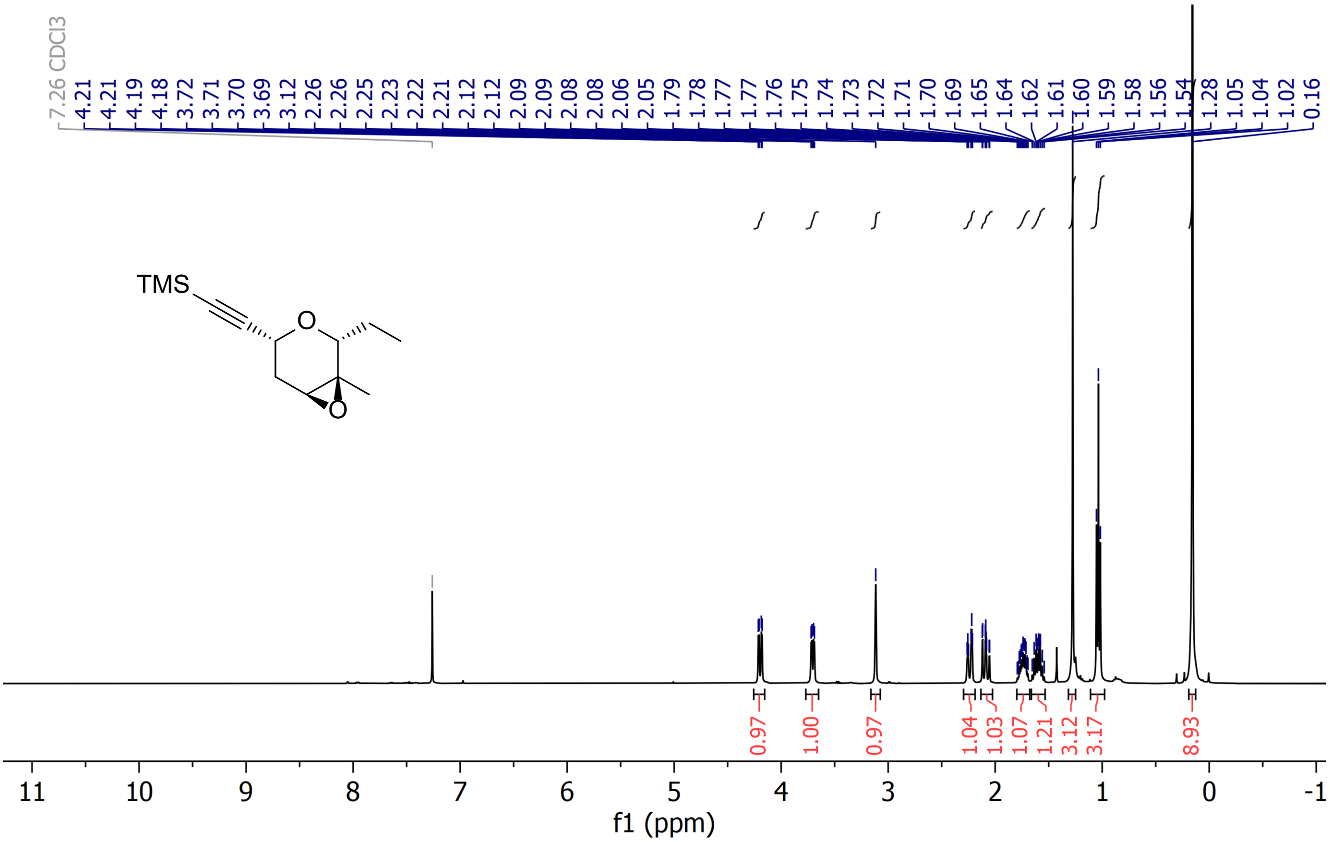

^{13}C-NMR (100 MHz, $CDCl_3$)

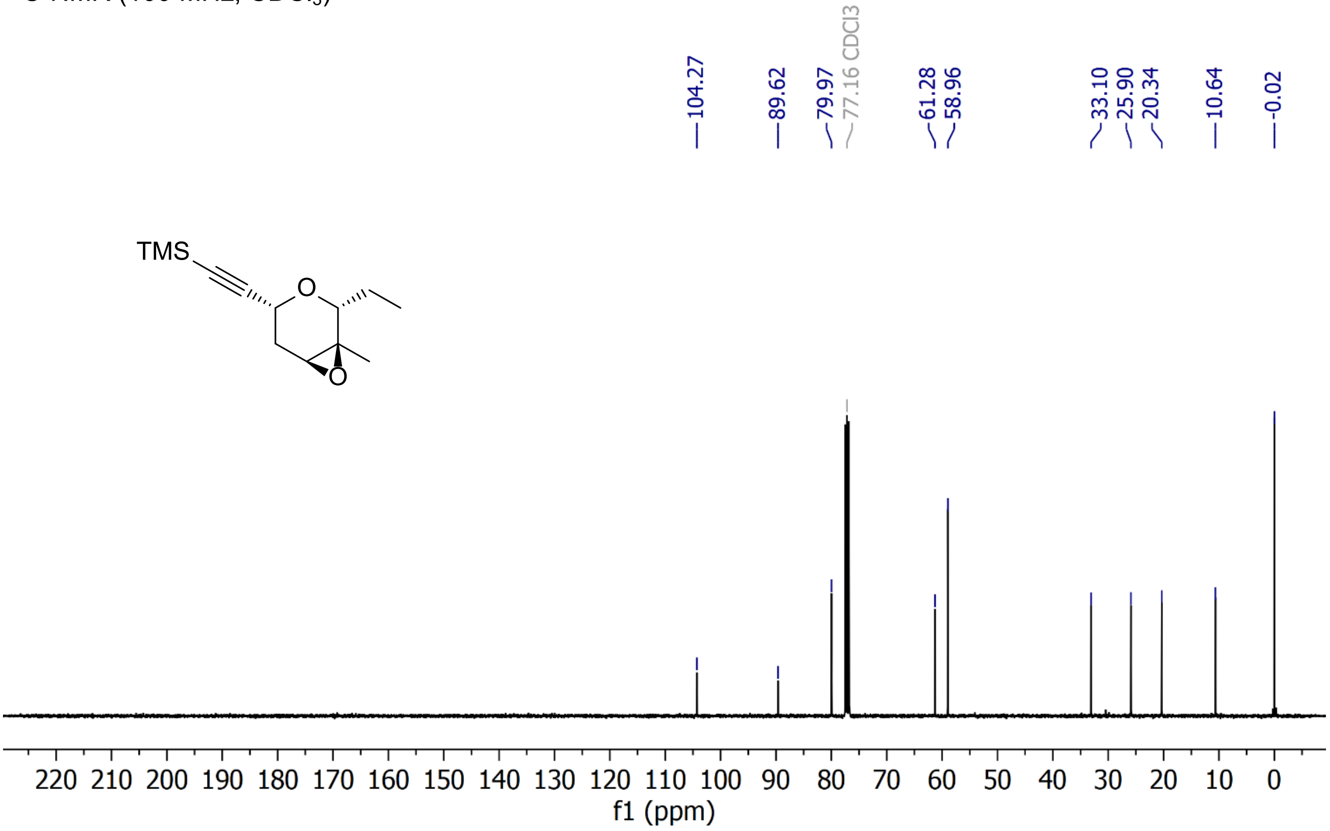

(((1R,2R,4R,6R)-2-Ethyl-1-methyl-3,7-dioxabicyclo[4.1.0]heptan-4-yl)ethynyl)trimethylsilane 47

^{1}H-NMR (400 MHz, $CDCl_3$)

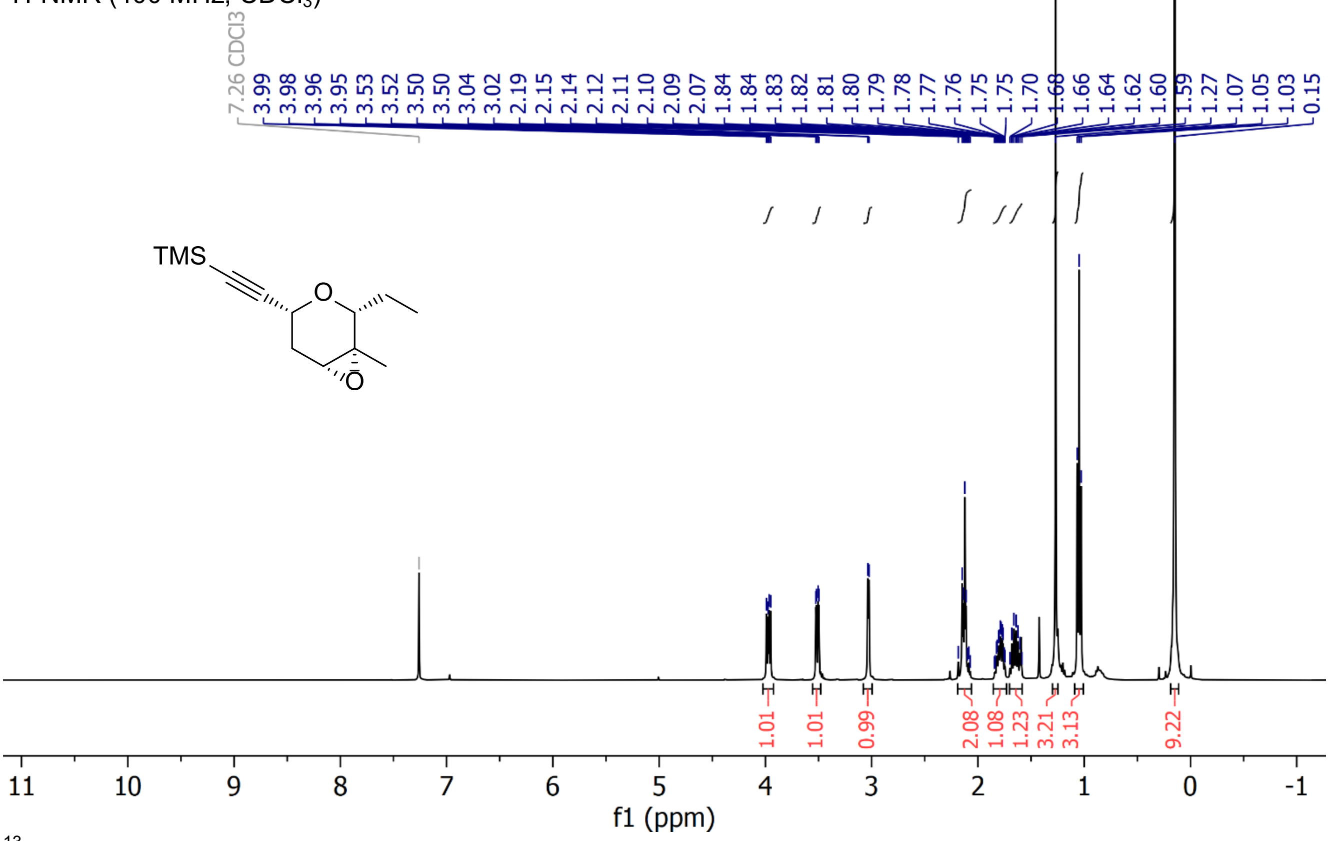

^{13}C-NMR (100 MHz, $CDCl_3$)

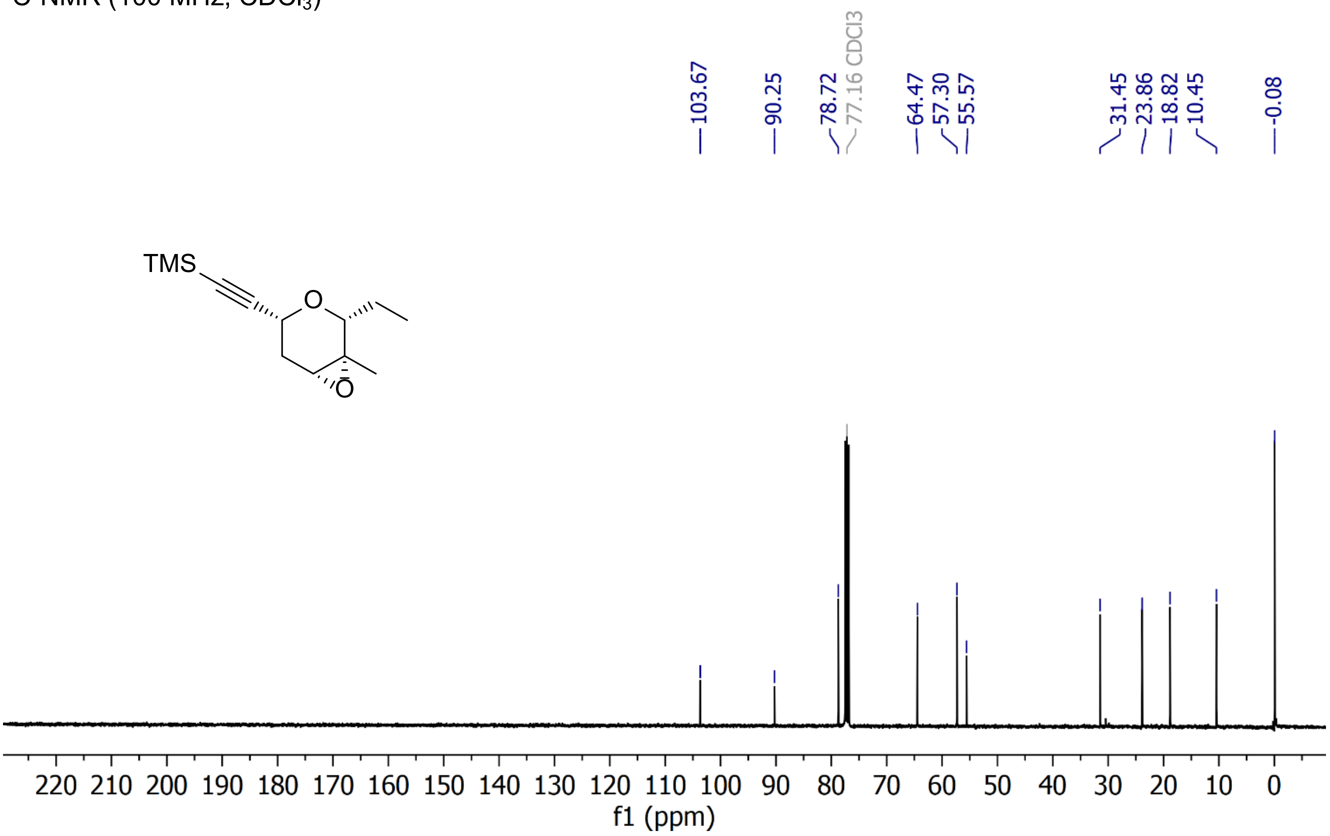

(2R,3S,4S,6R)-2-ethyl-3-methyl-6-((trimethylsilyl)ethynyl)tetrahydro-2H-pyran-4-ol 48

^{1}H-NMR (400 MHz, $CDCl_3$)

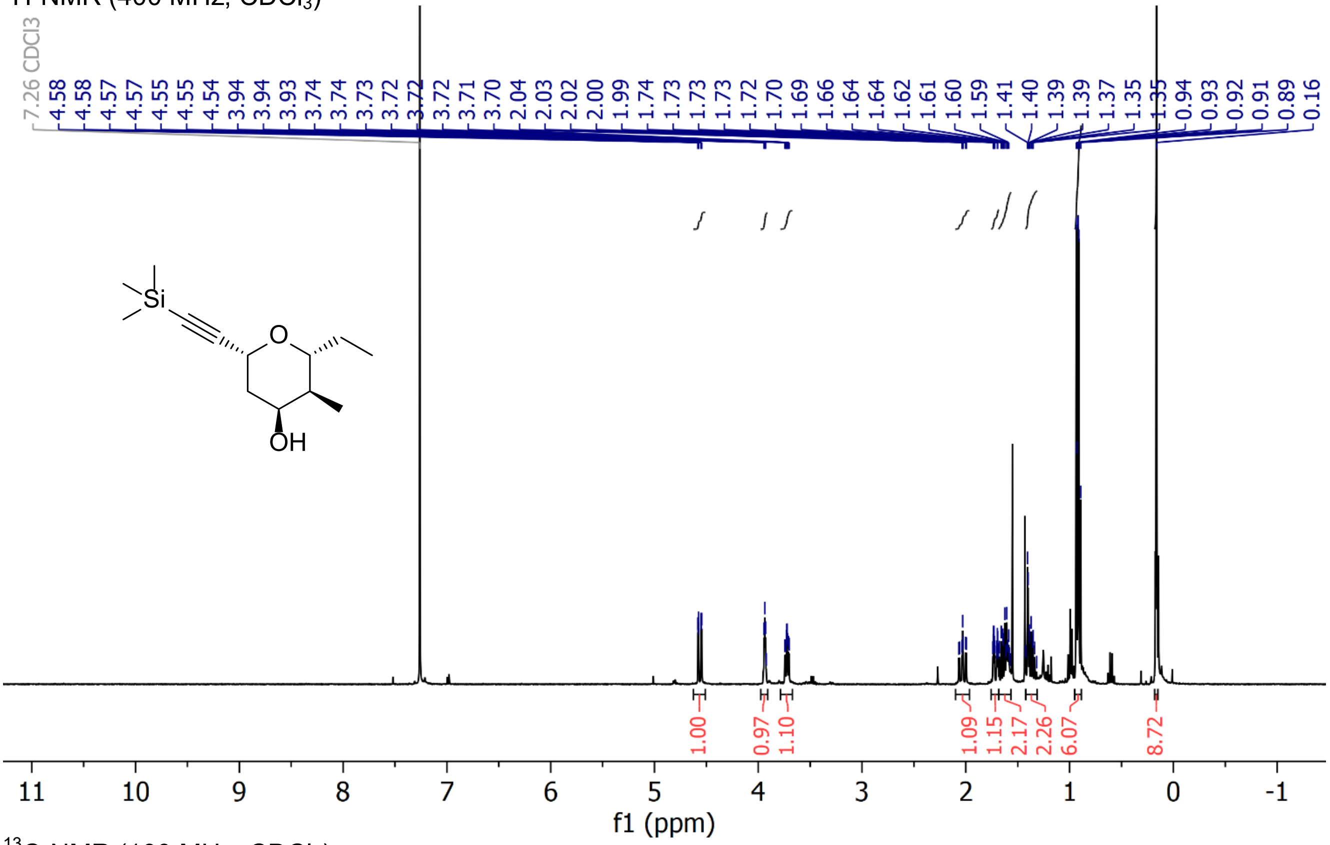

^{13}C-NMR (100 MHz, $CDCl_3$)

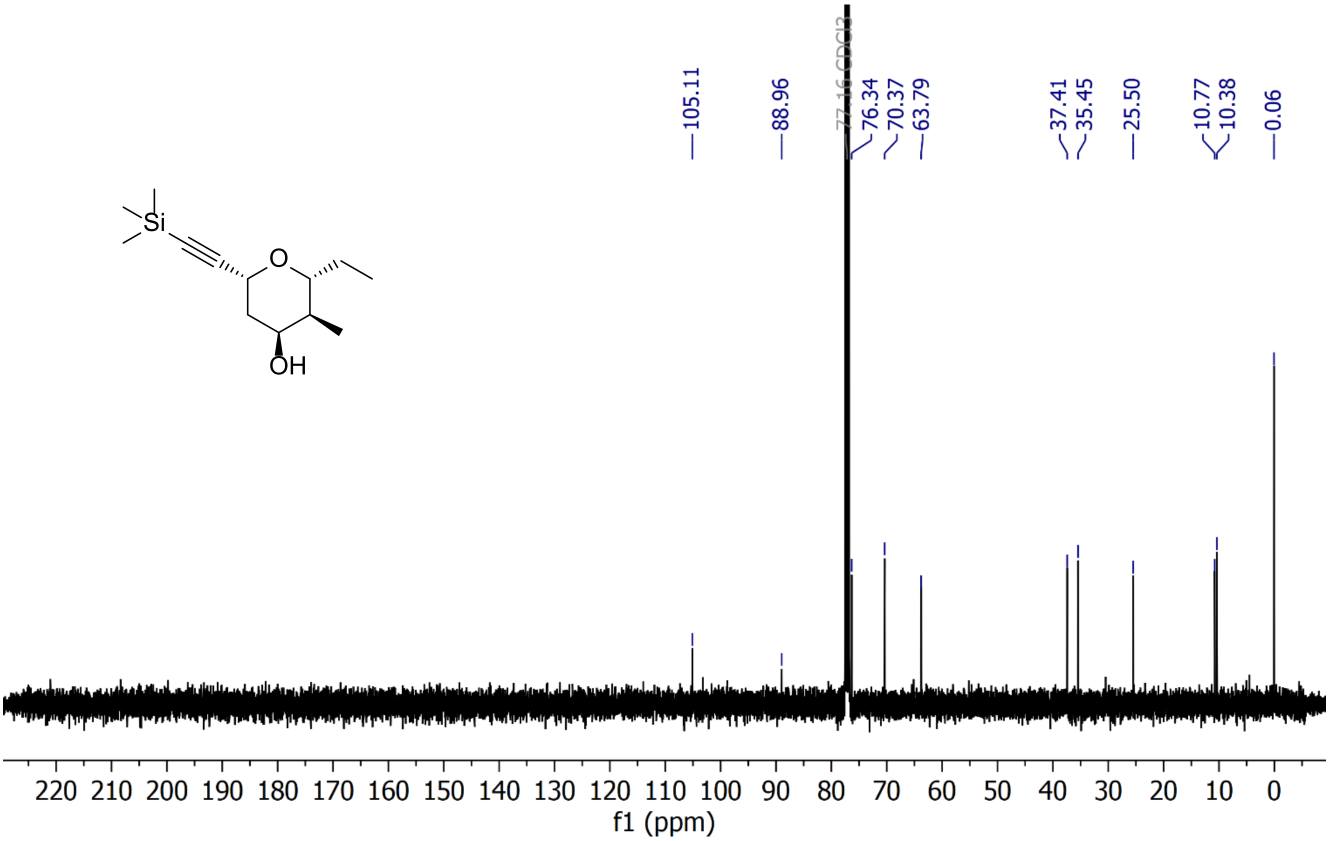

(2R,3S,4R,6R)-2-ethyl-3-methyl-6-((trimethylsilyl)ethynyl)tetrahydro-2H-pyran-4-ol 48

^{1}H-NMR (400 MHz, $CDCl_3$)

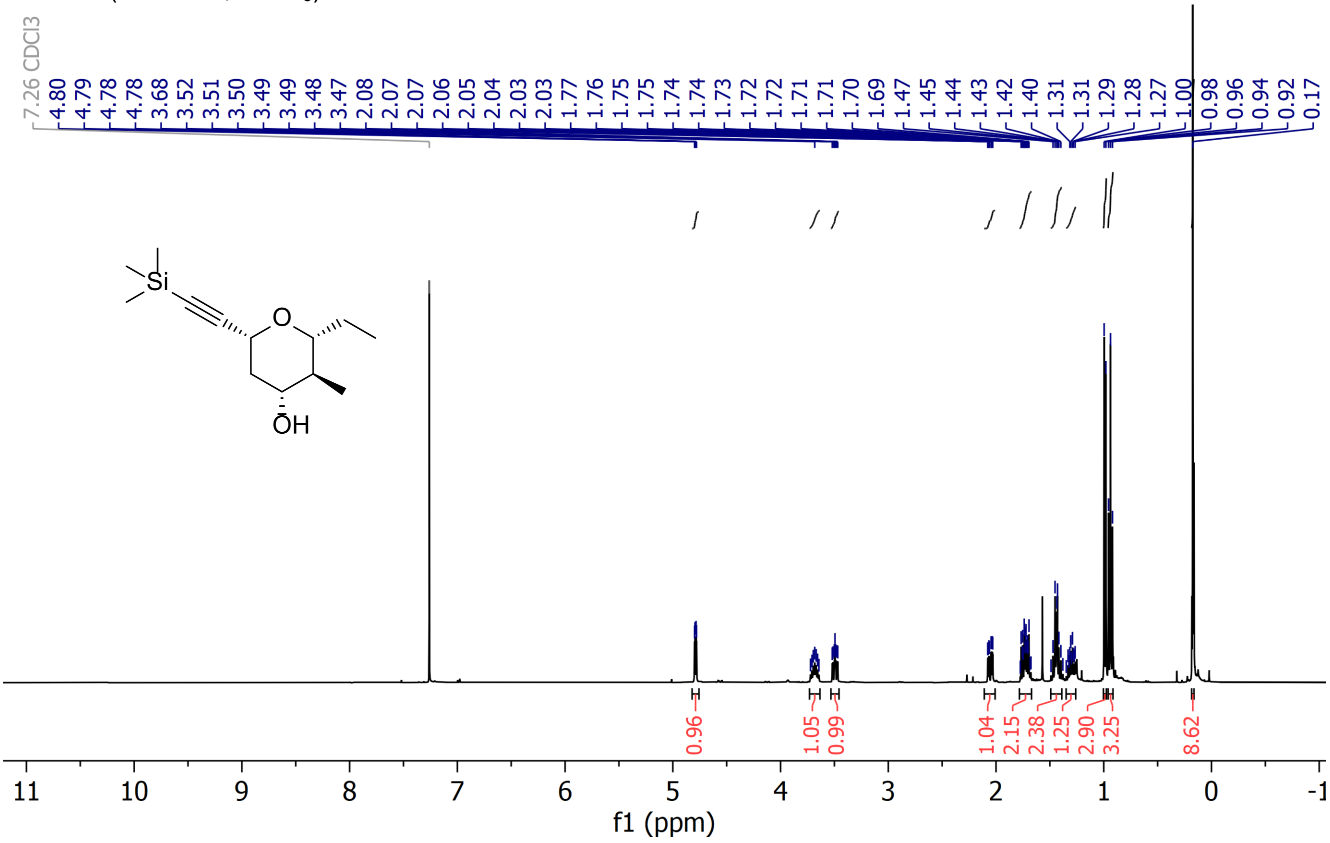

^{13}C-NMR (100 MHz, $CDCl_3$)

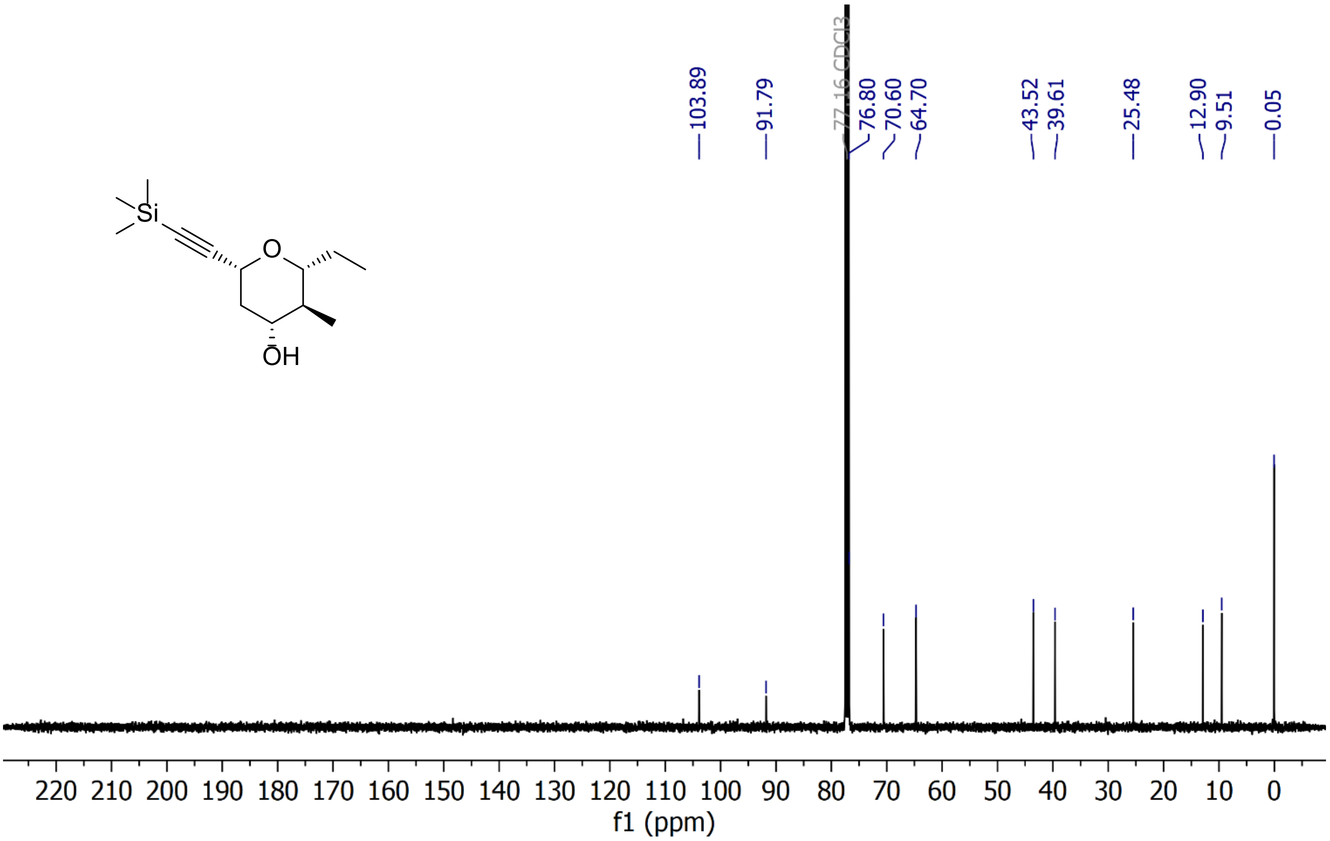

(2R,3R,4S)-2-Ethyl-3-methyl-3-((trimethylsilyl)ethynyl)tetrahydro-2H-pyran-4-ol 49

^{1}H-NMR (400 MHz, $CDCl_3$)

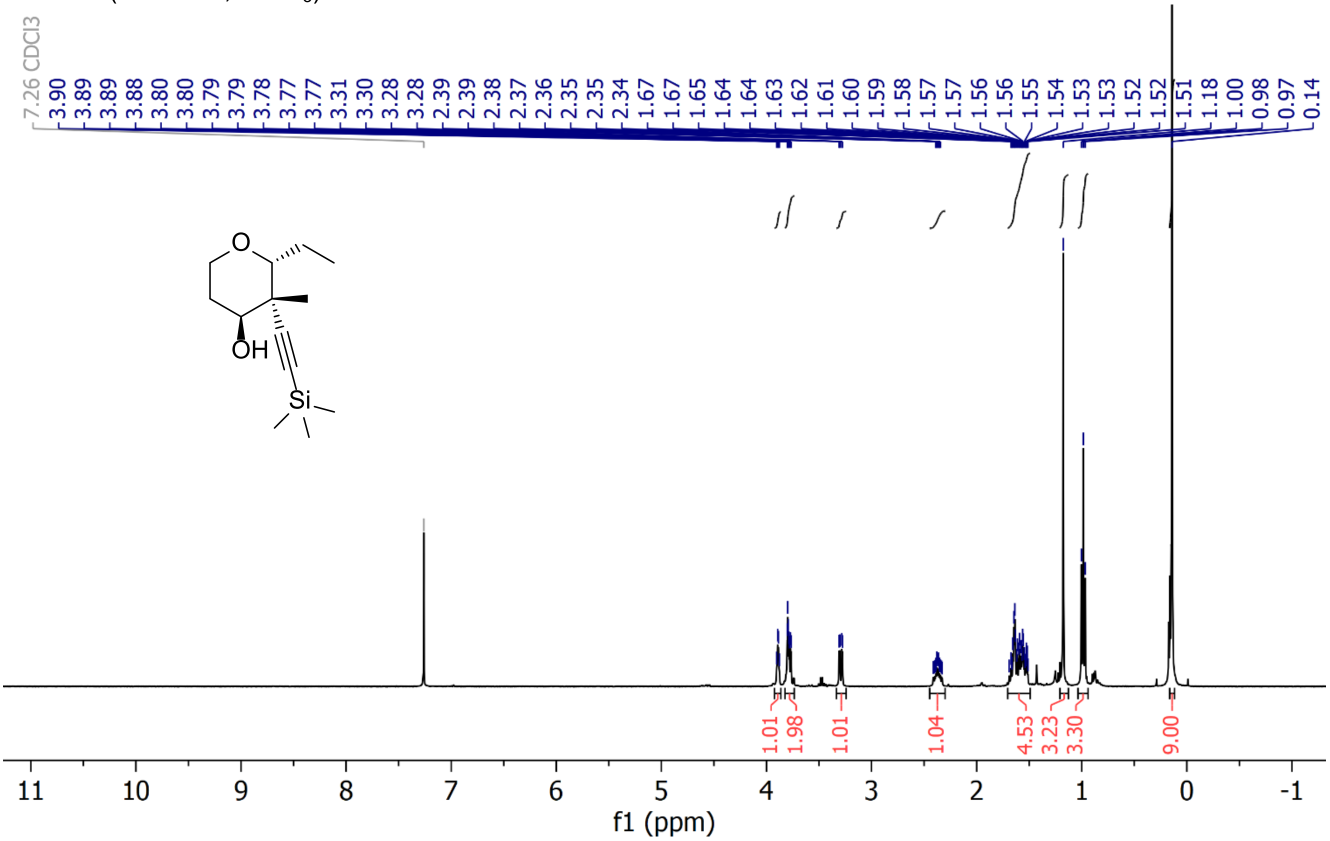

^{13}C-NMR (100 MHz, $CDCl_3$)

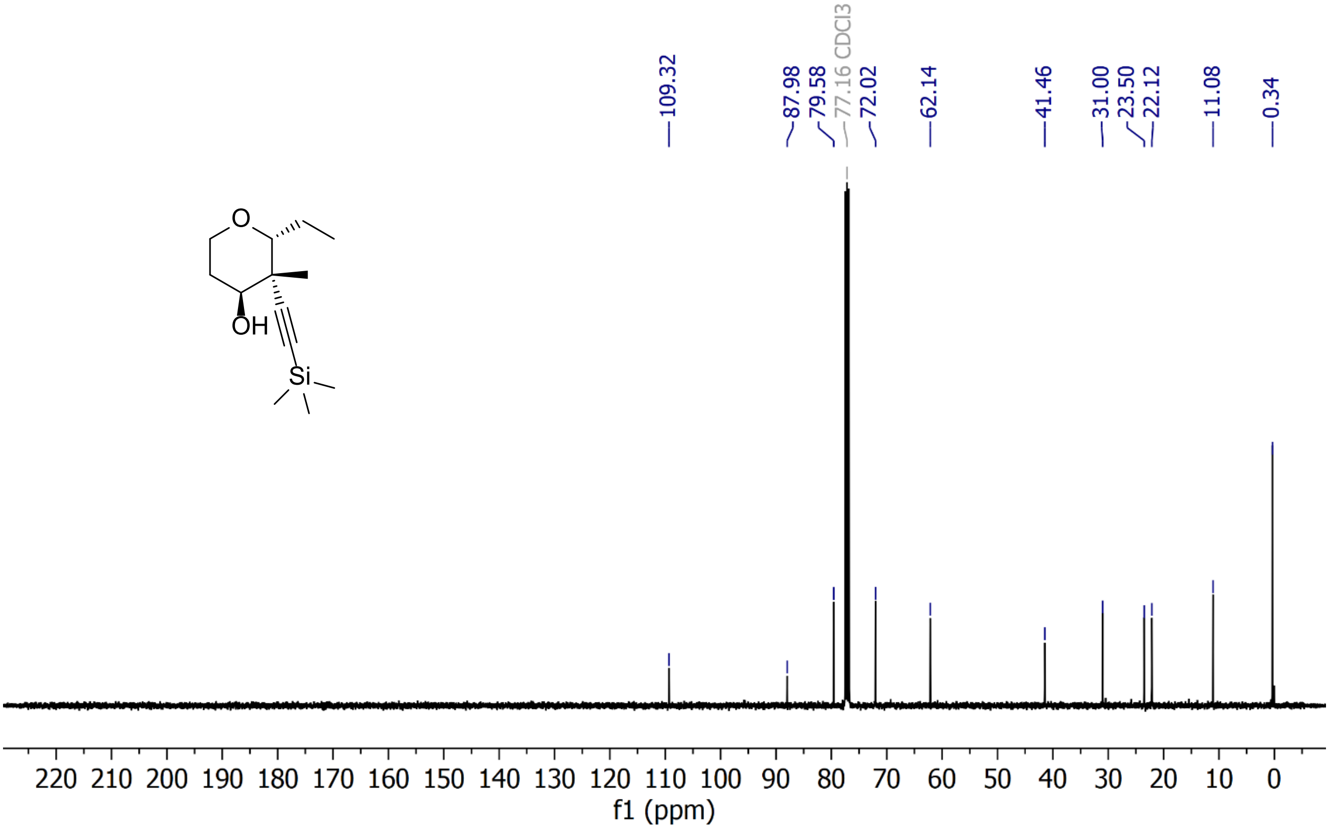

(2R,3R,6R)-2-Ethyl-2-(trimethylsilyl)-6-((trimethylsilyl)ethynyl)tetrahydro-2H-pyran-3-ol S7

^{1}H-NMR (400 MHz, $CDCl_3$)

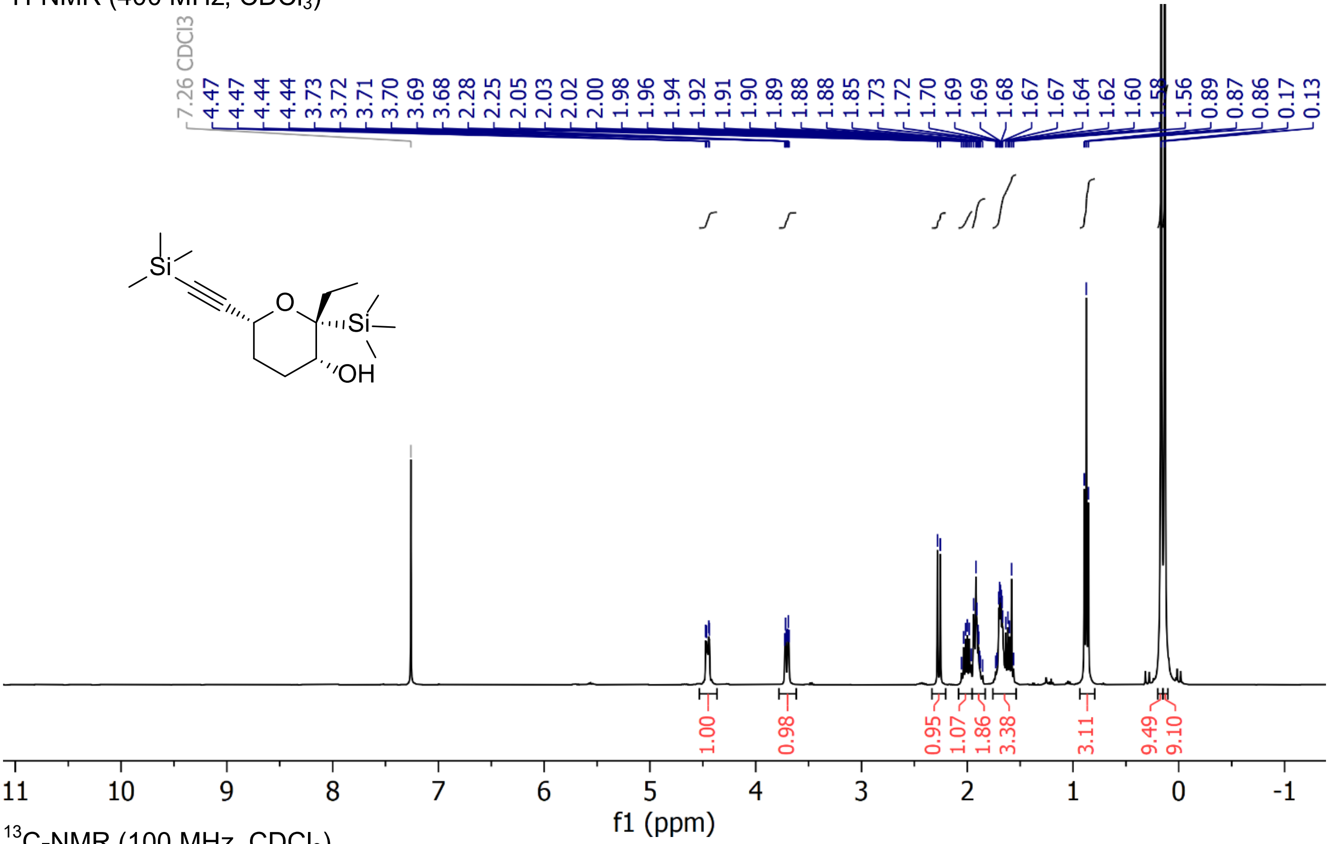

^{13}C-NMR (100 MHz, $CDCl_3$)

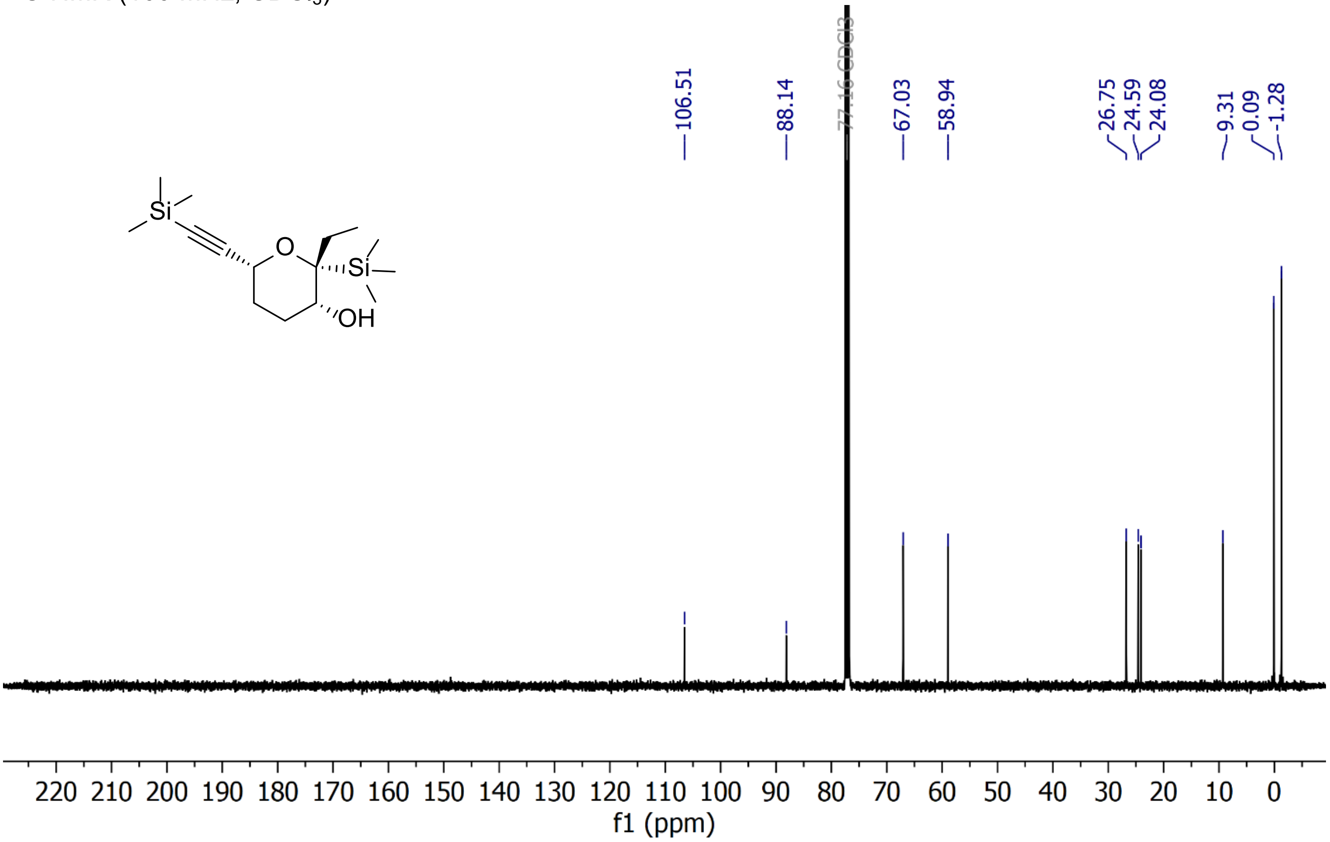

(2S,3R,6R)-2-Ethyl-6-ethynyltetrahydro-2H-pyran-3-ol S8

^{1}H-NMR (400 MHz, $CDCl_3$)

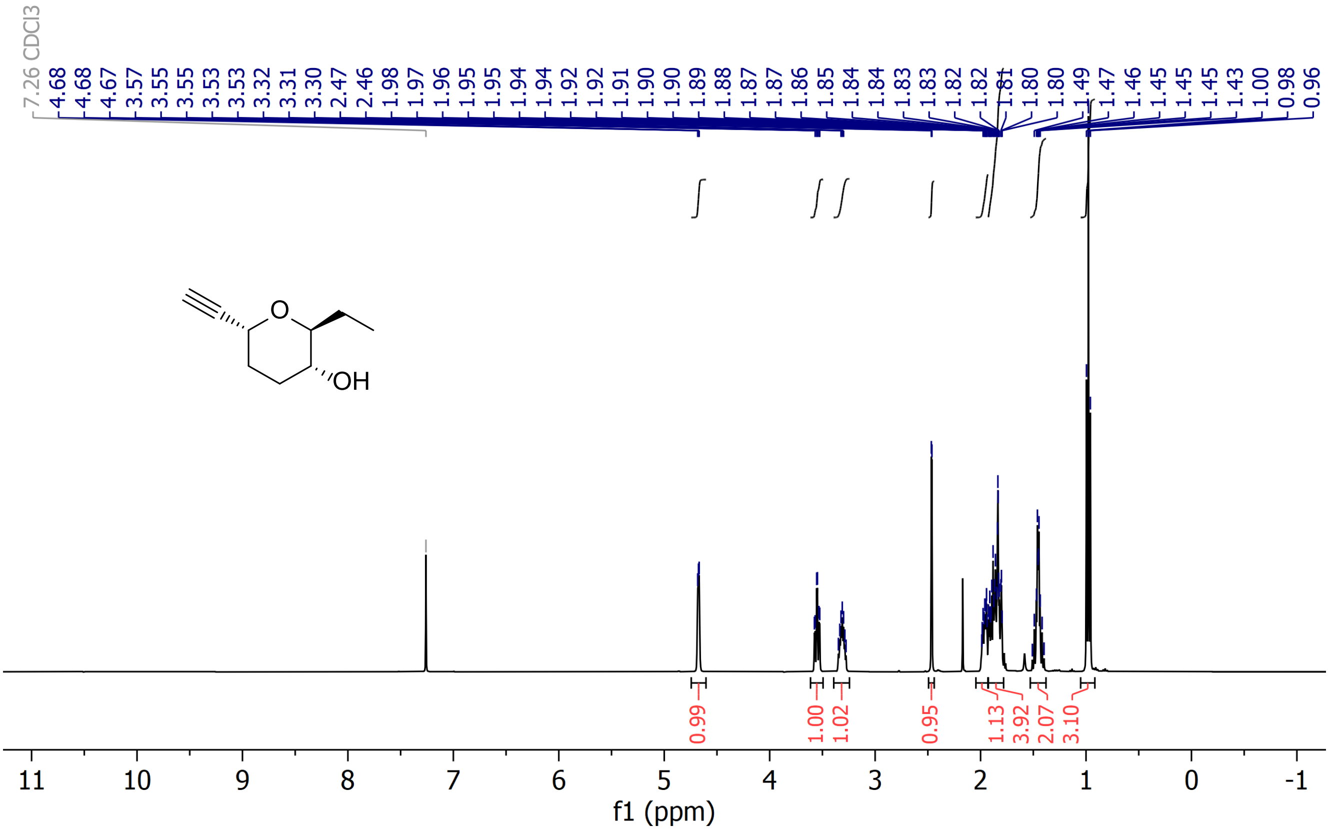

^{13}C-NMR (100 MHz, $CDCl_3$)

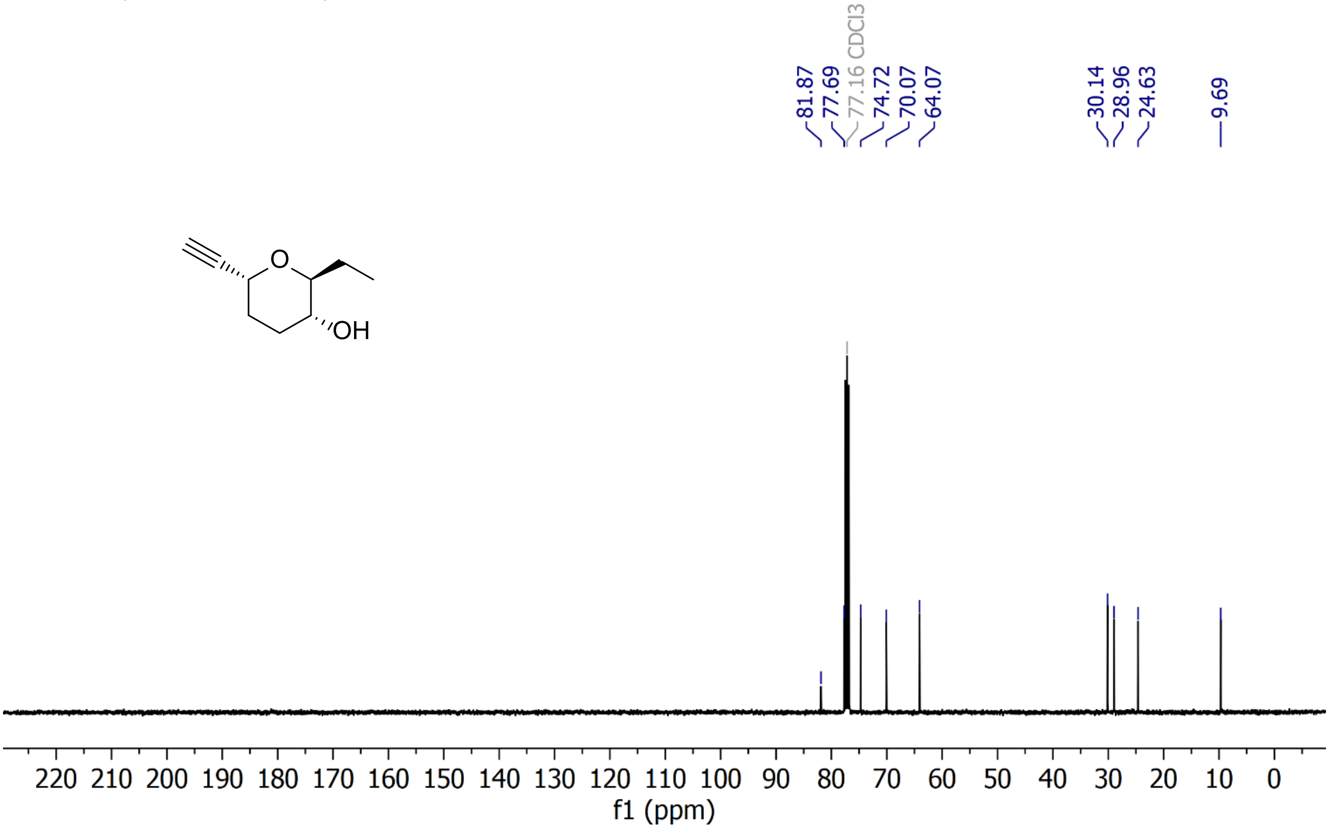

(2S,6R)-2-Ethyl-6-ethynyldihydro-2H-pyran-3(4H)-one S9

^{1}H-NMR (400 MHz, $CDCl_3$)

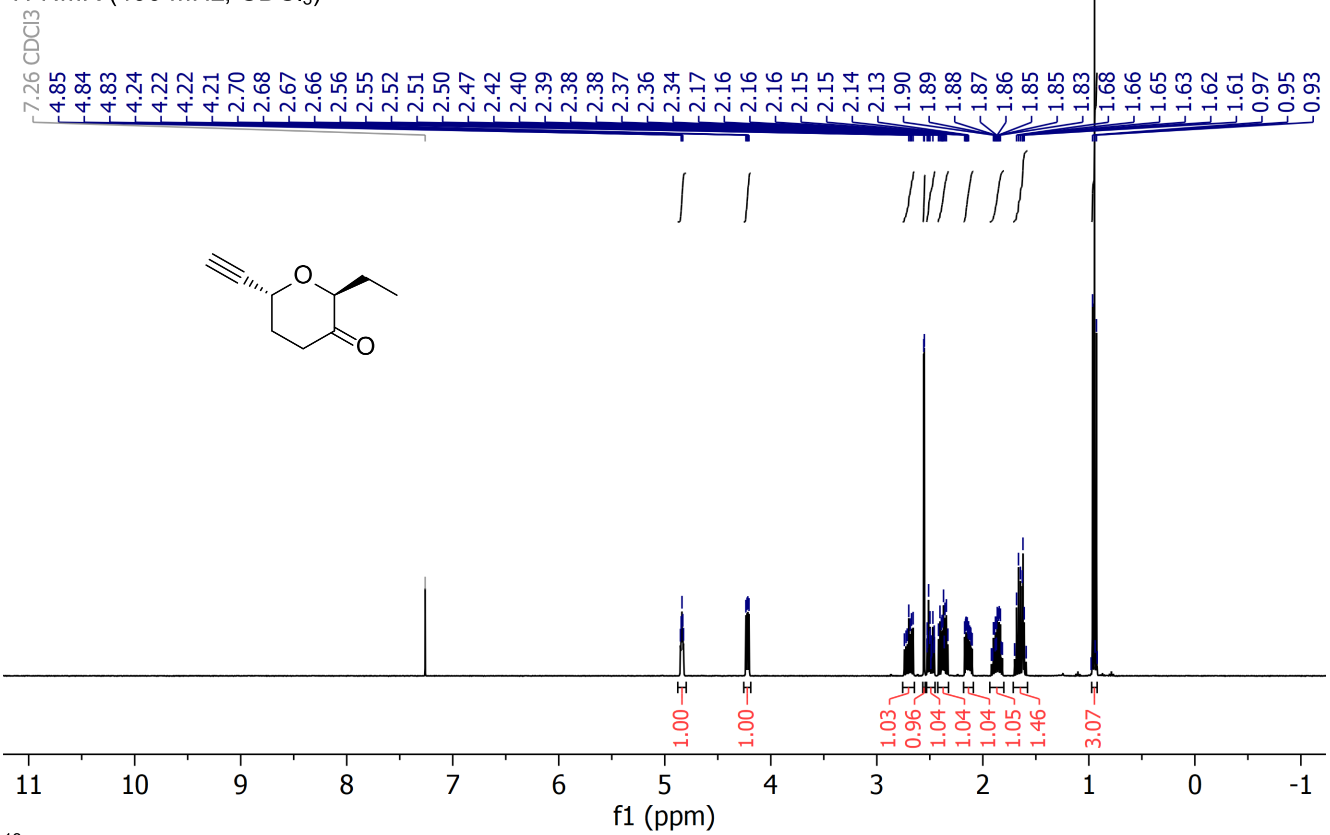

^{13}C-NMR (100 MHz, $CDCl_3$)

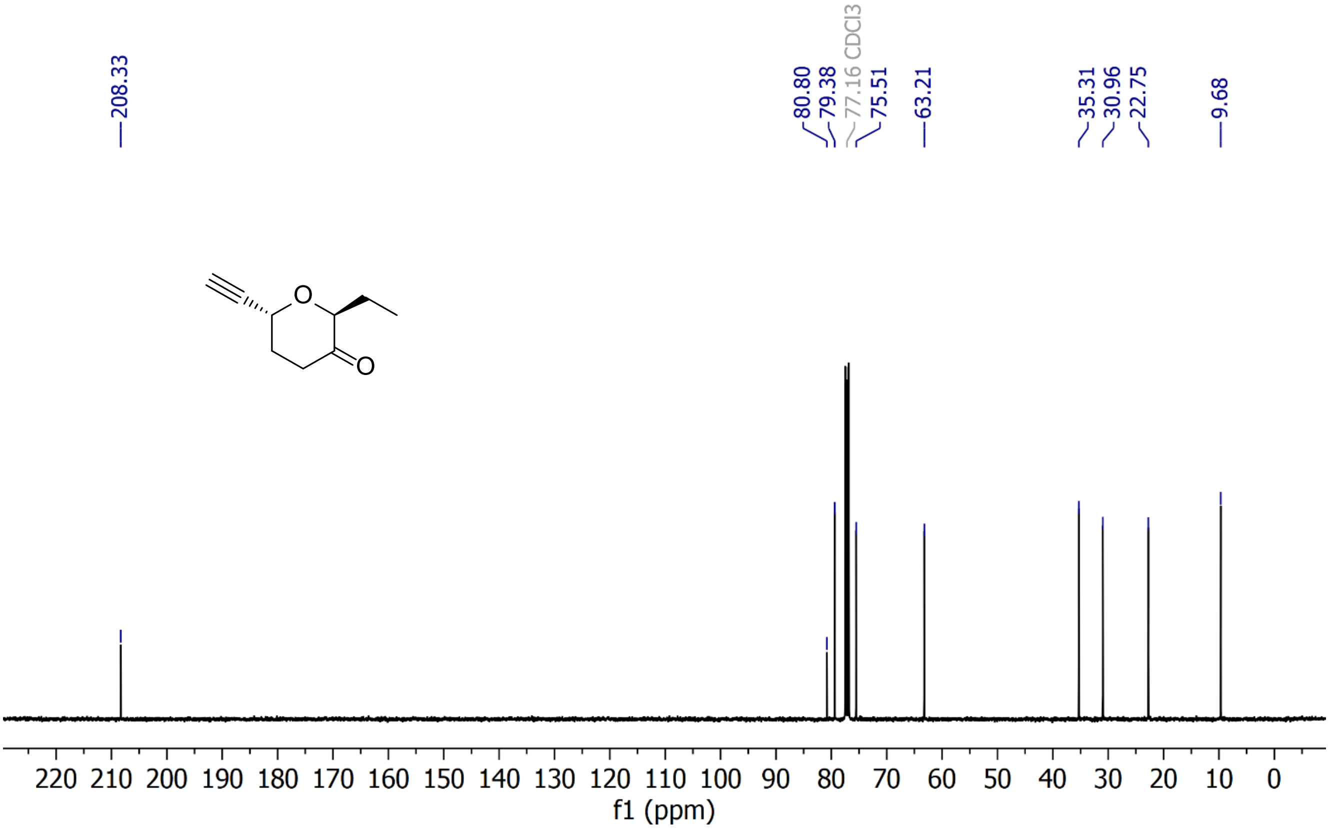

(2S,3R,6R)-2-ethyl-6-ethynyl-3-methyltetrahydro-2H-pyran-3-ol 52

^{1}H-NMR (400 MHz, $CDCl_3$)

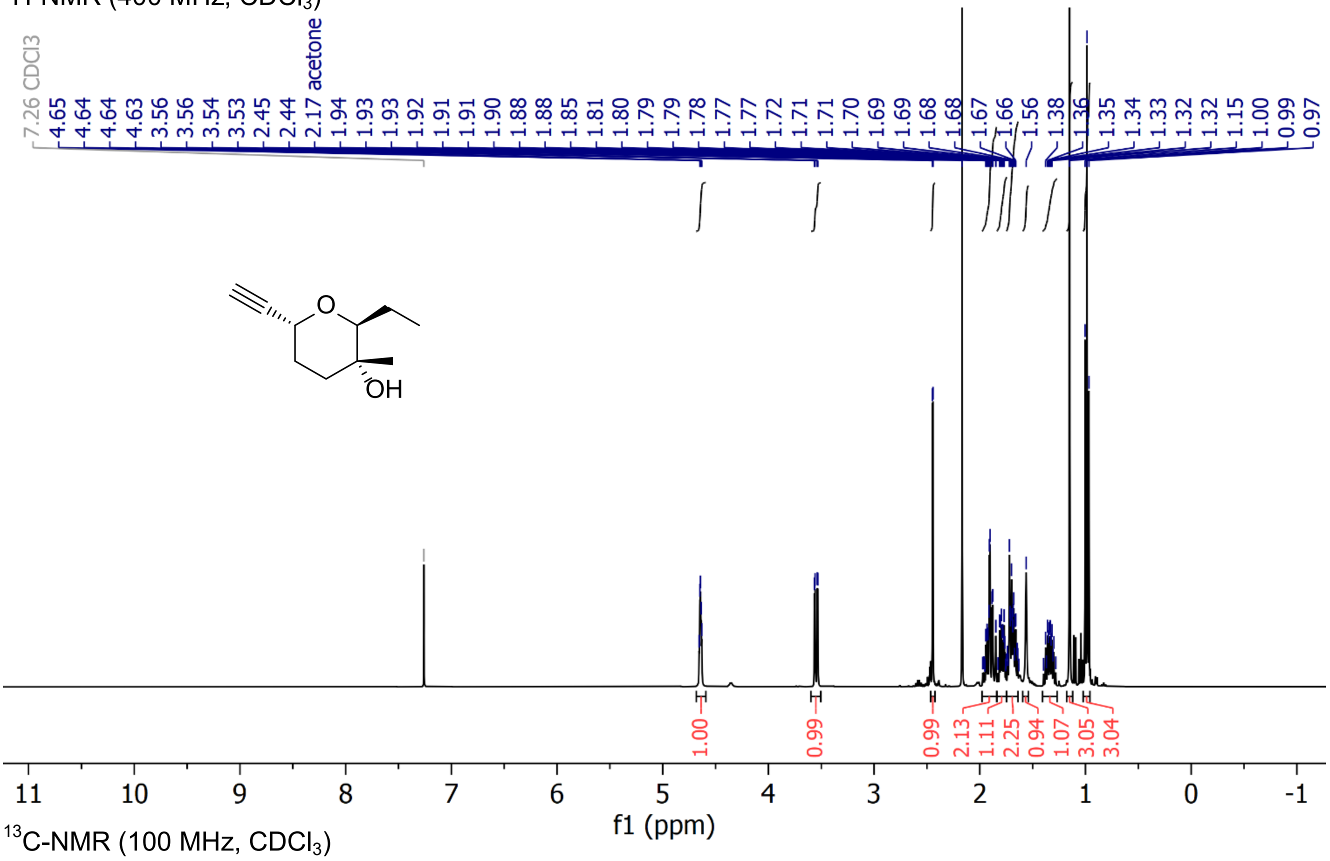

^{13}C-NMR (100 MHz, $CDCl_3$)

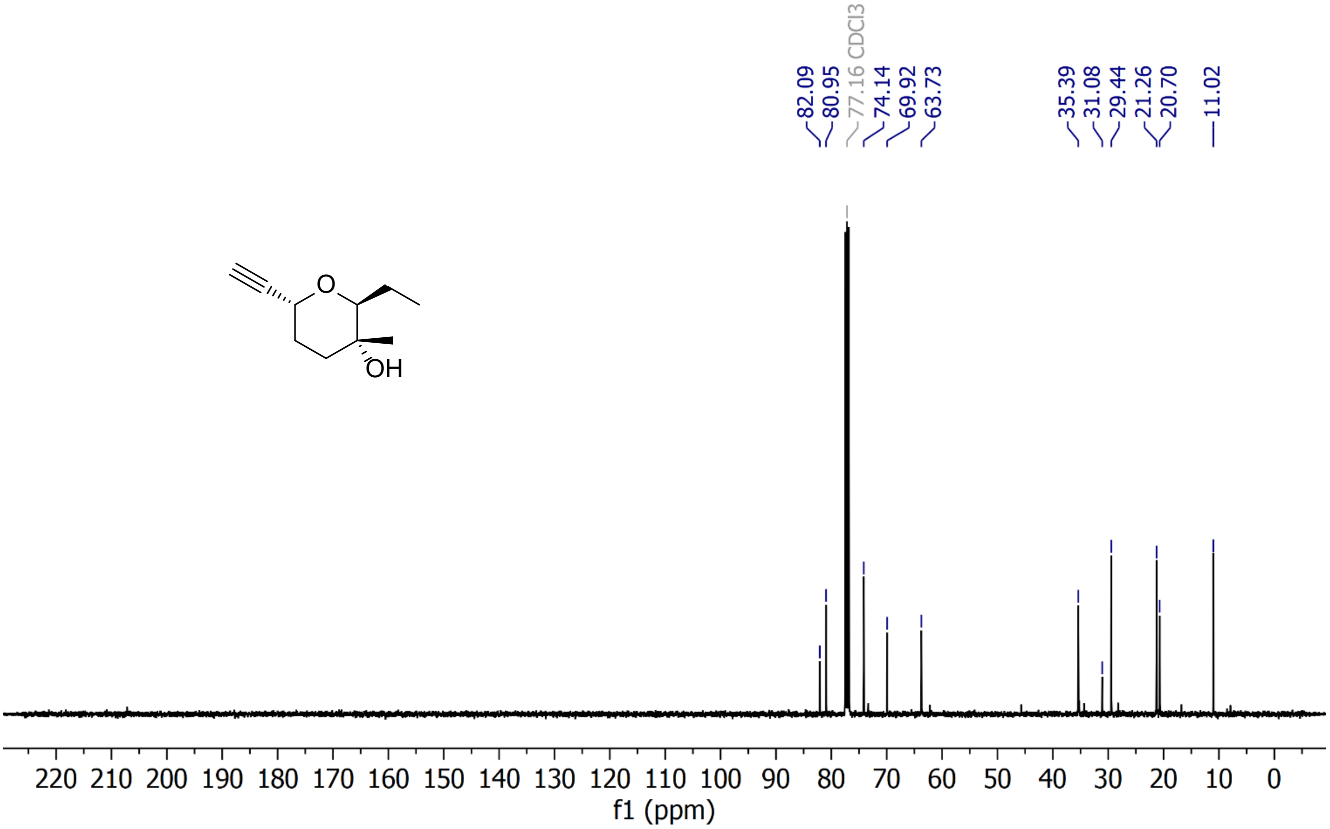

(2S,3S,6R)-2-ethyl-6-ethynyl-3-methyltetrahydro-2H-pyran-3-ol 52

^{1}H-NMR (400 MHz, $CDCl_3$)

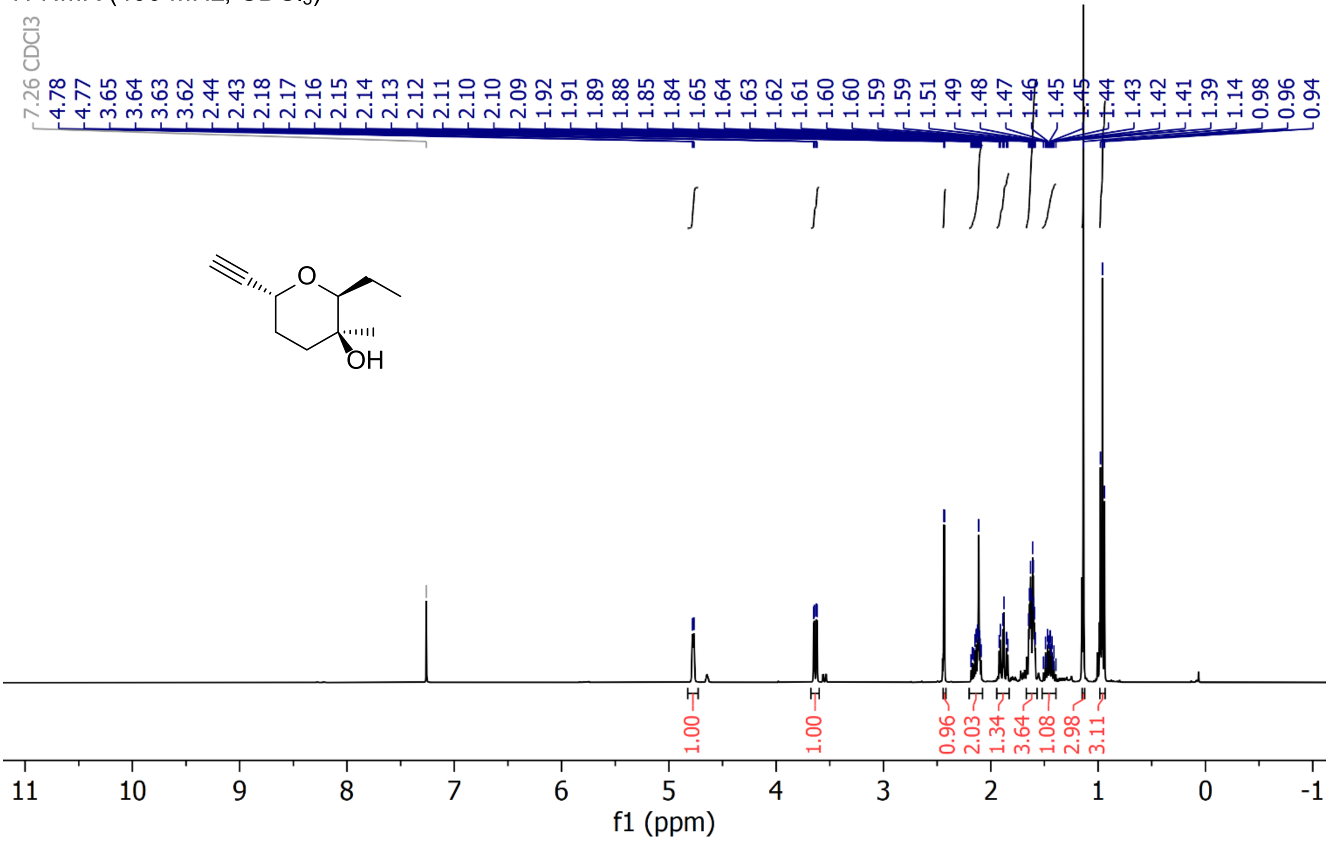

^{13}C-NMR (100 MHz, $CDCl_3$)

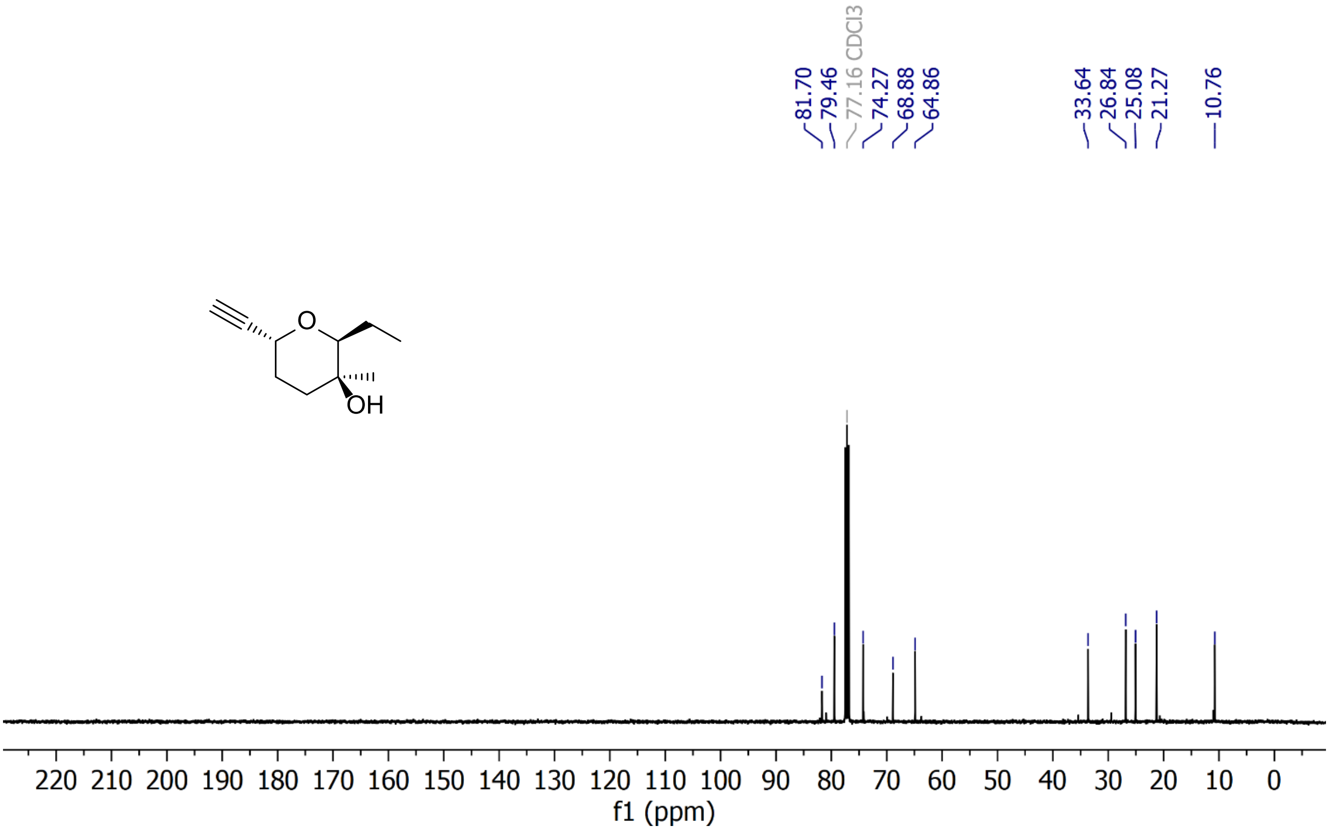

(2R,3S,6R)-2-Ethyl-6-ethynyl-3-methyltetrahydro-2H-pyran 45

^{1}H-NMR (500 MHz, $CDCl_3$)

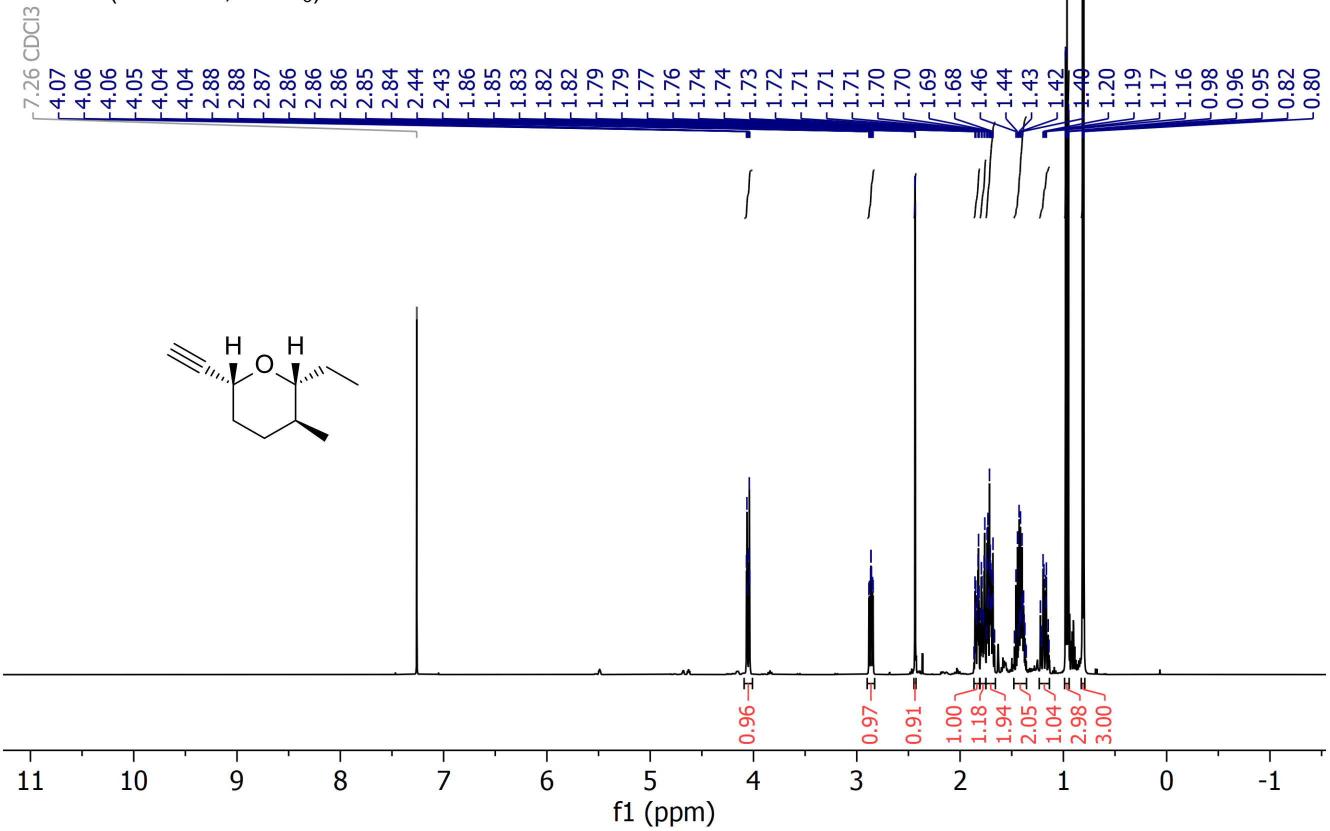

^{13}C-NMR (100 MHz, $CDCl_3$)

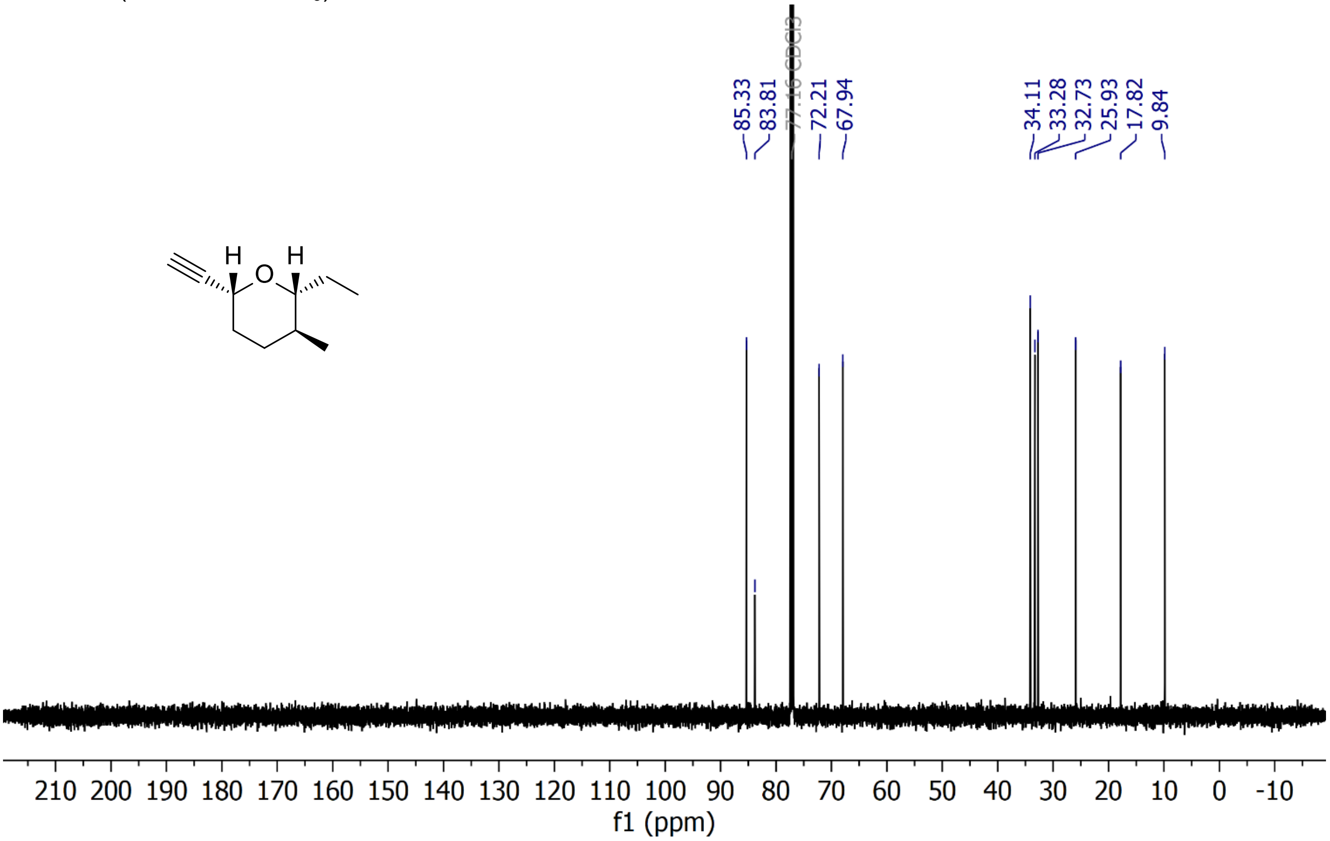

gNOESY

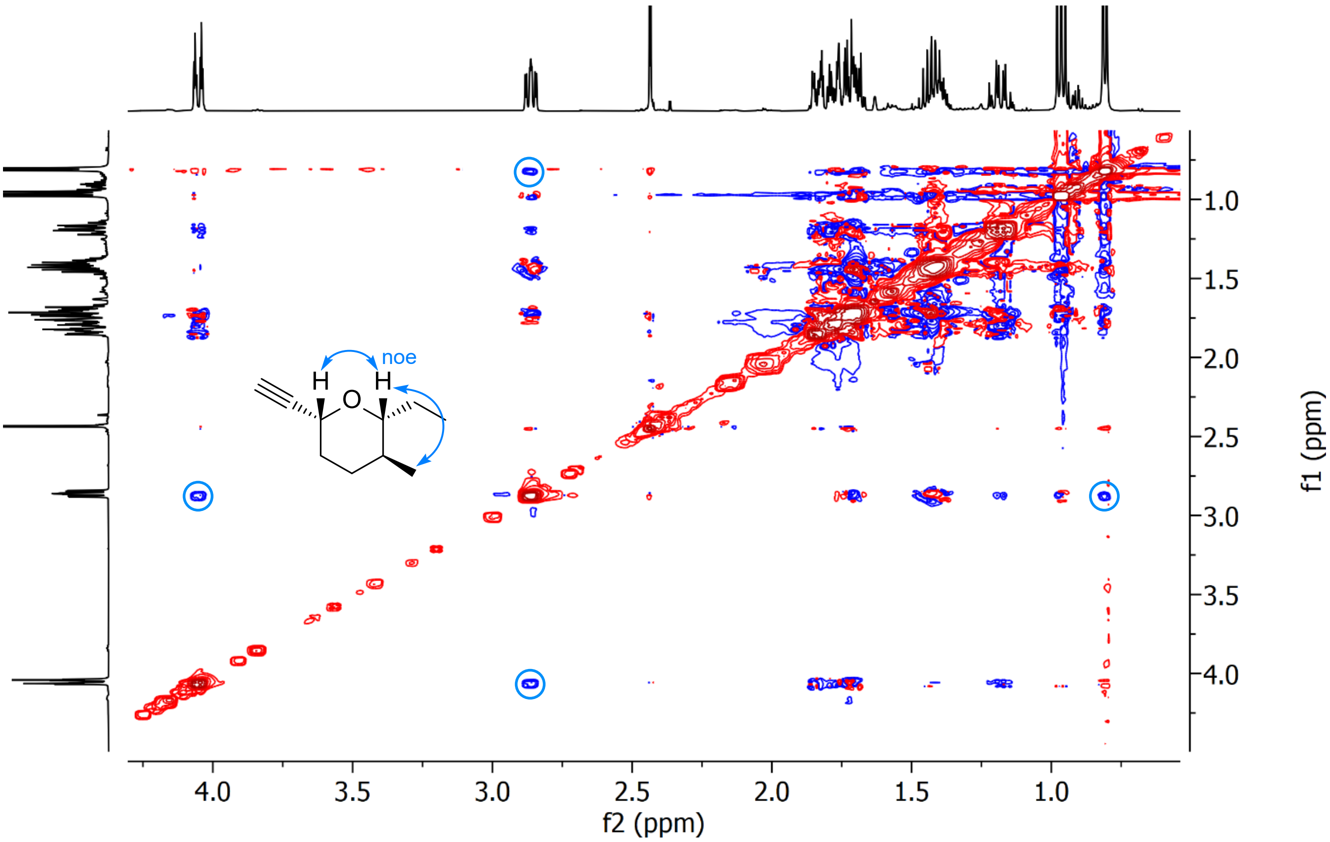

Tributyl((E)-2-((2R,5S,6R)-6-ethyl-5-methyltetrahydro-2H-pyran-2-yl)prop-1-en-1-yl)stannane 53

^{1}H-NMR (500 MHz, $CDCl_3$)

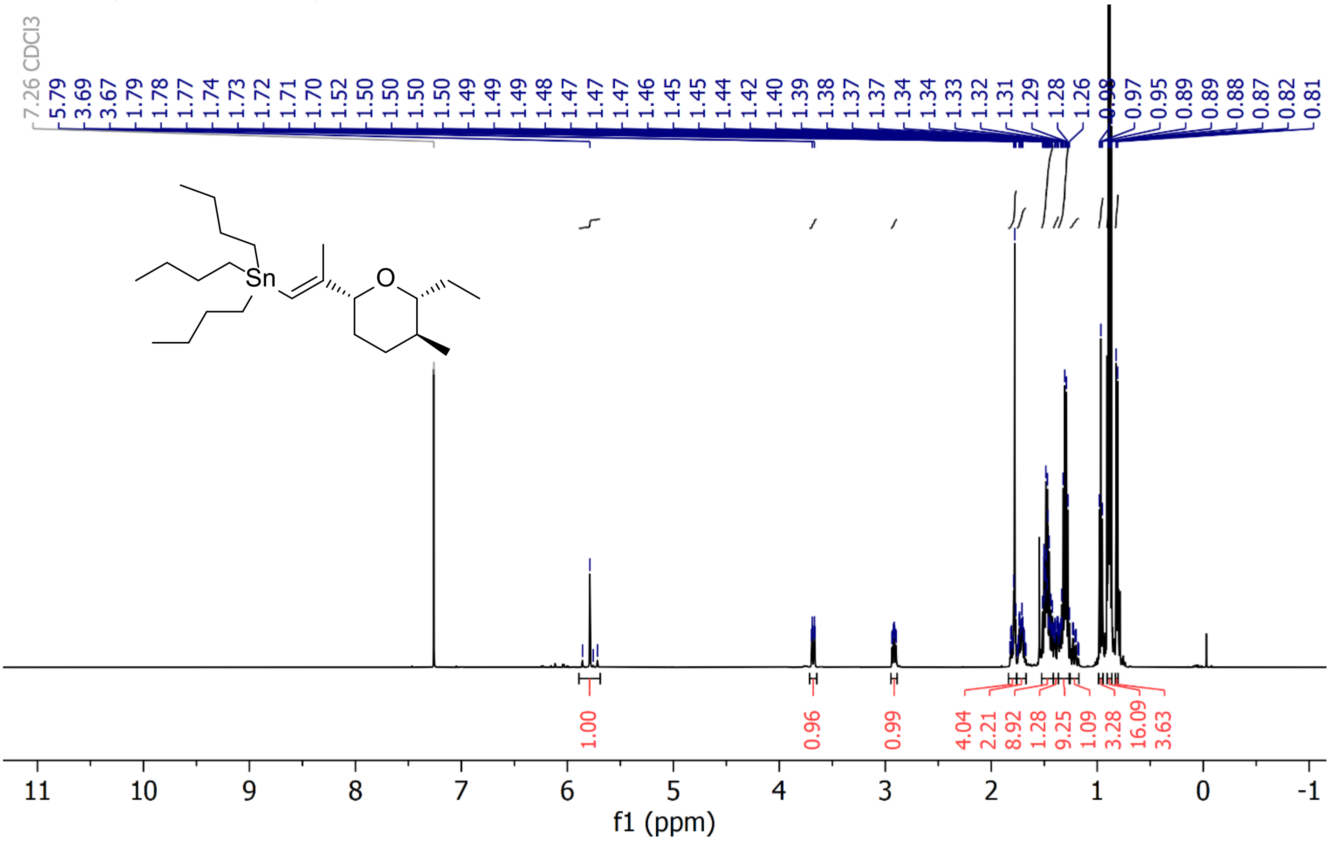

^{13}C-NMR (125 MHz, $CDCl_3$)

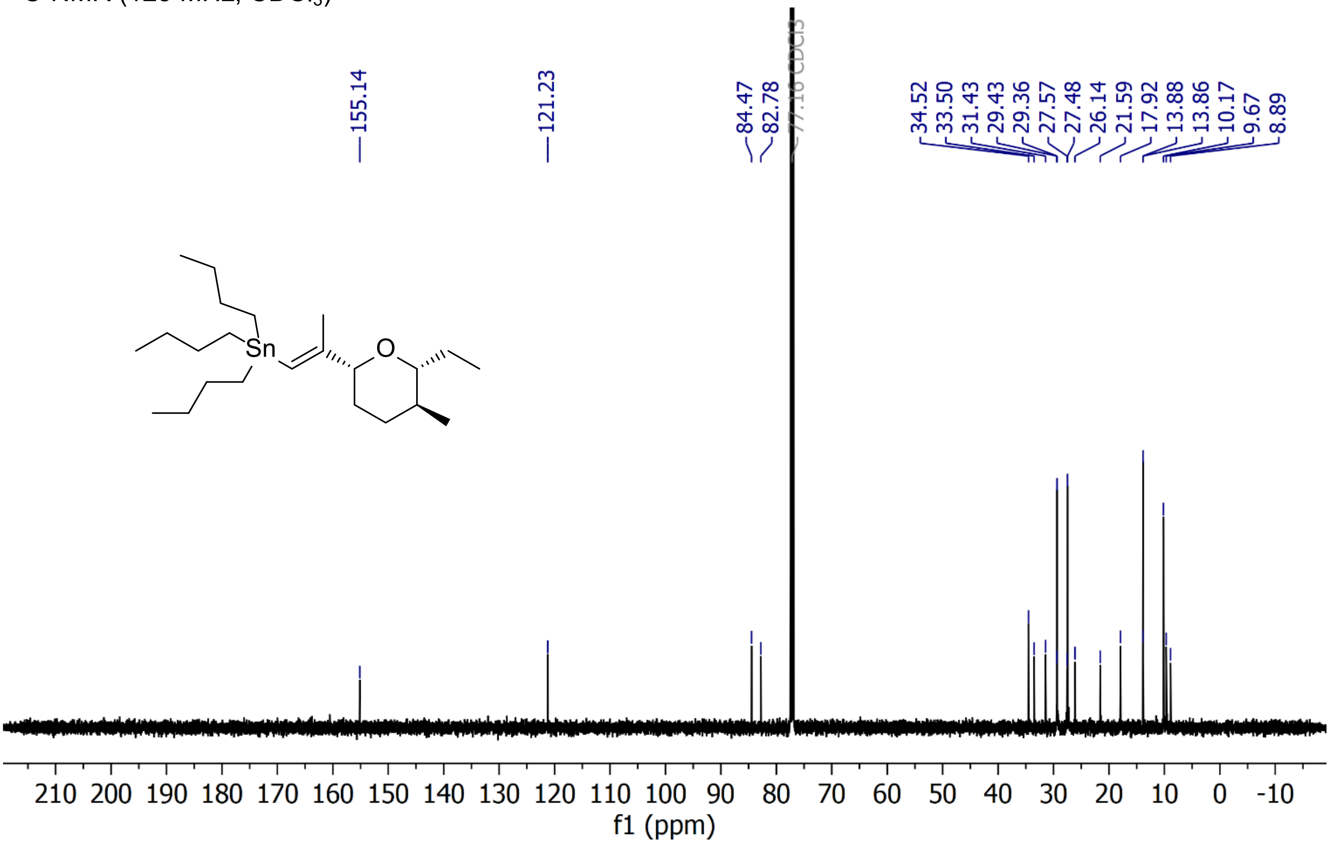

14-*epi* jerangolid E 55

^{1}H-NMR (400 MHz, CD_3OD)

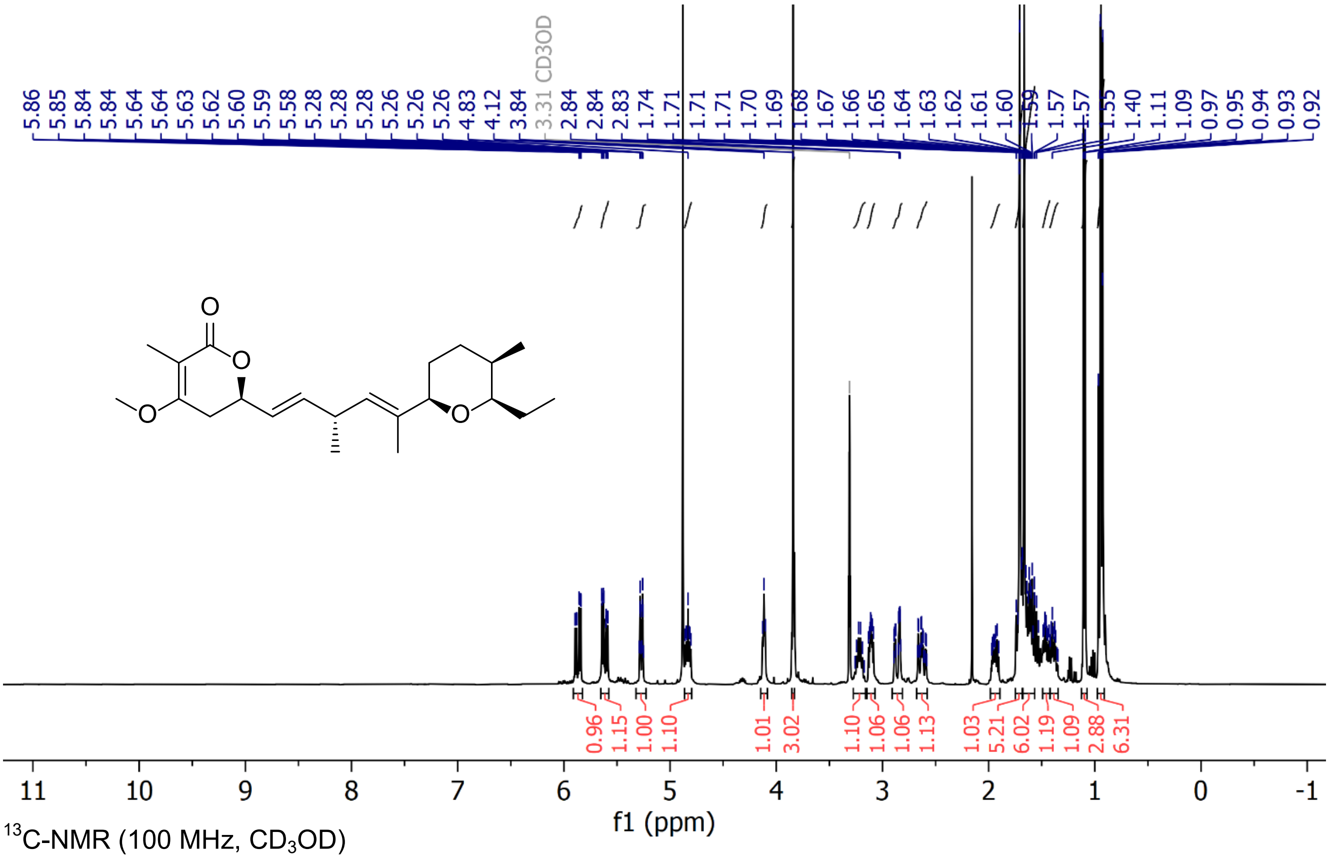

^{13}C-NMR (100 MHz, CD_3OD)

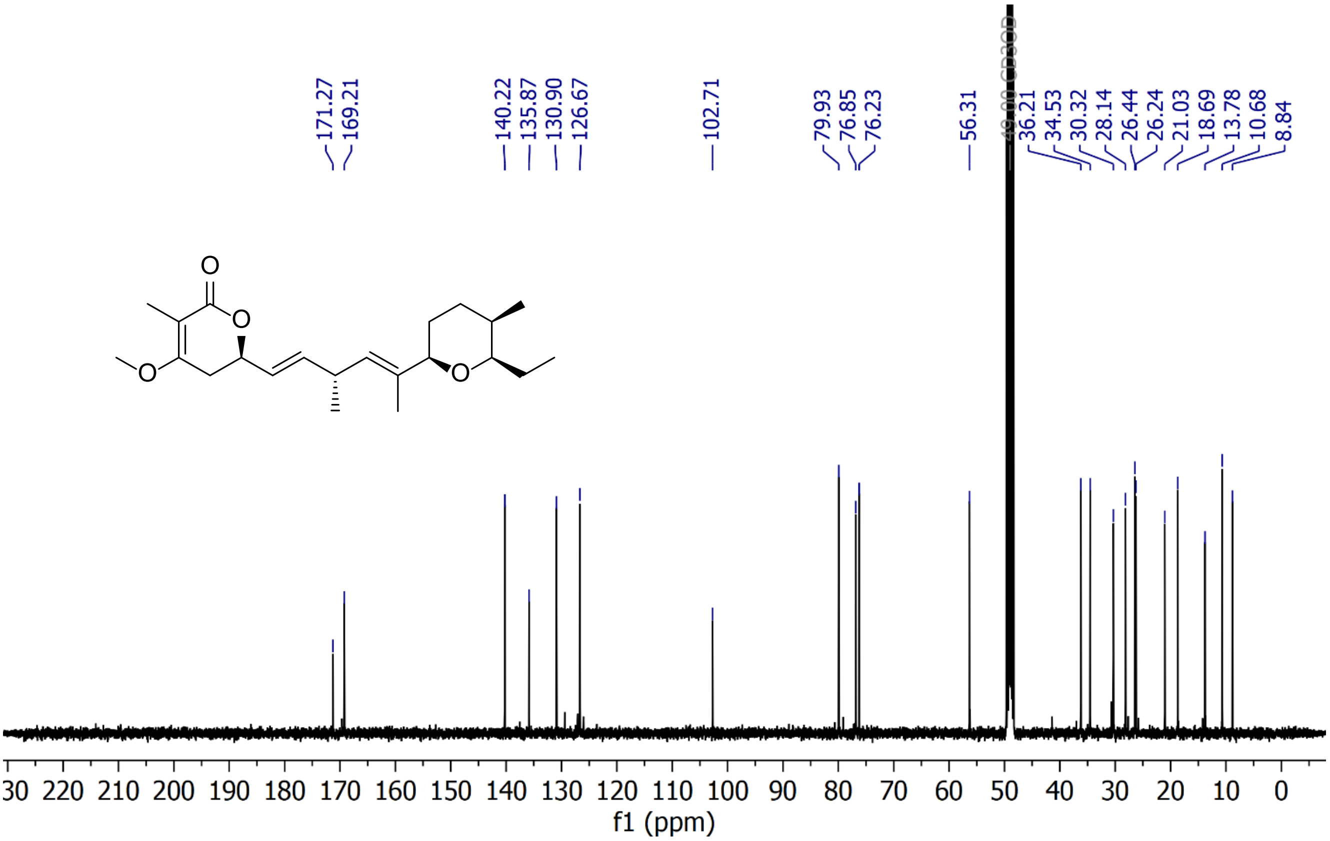

HH-gCOSY (CD_3OD)

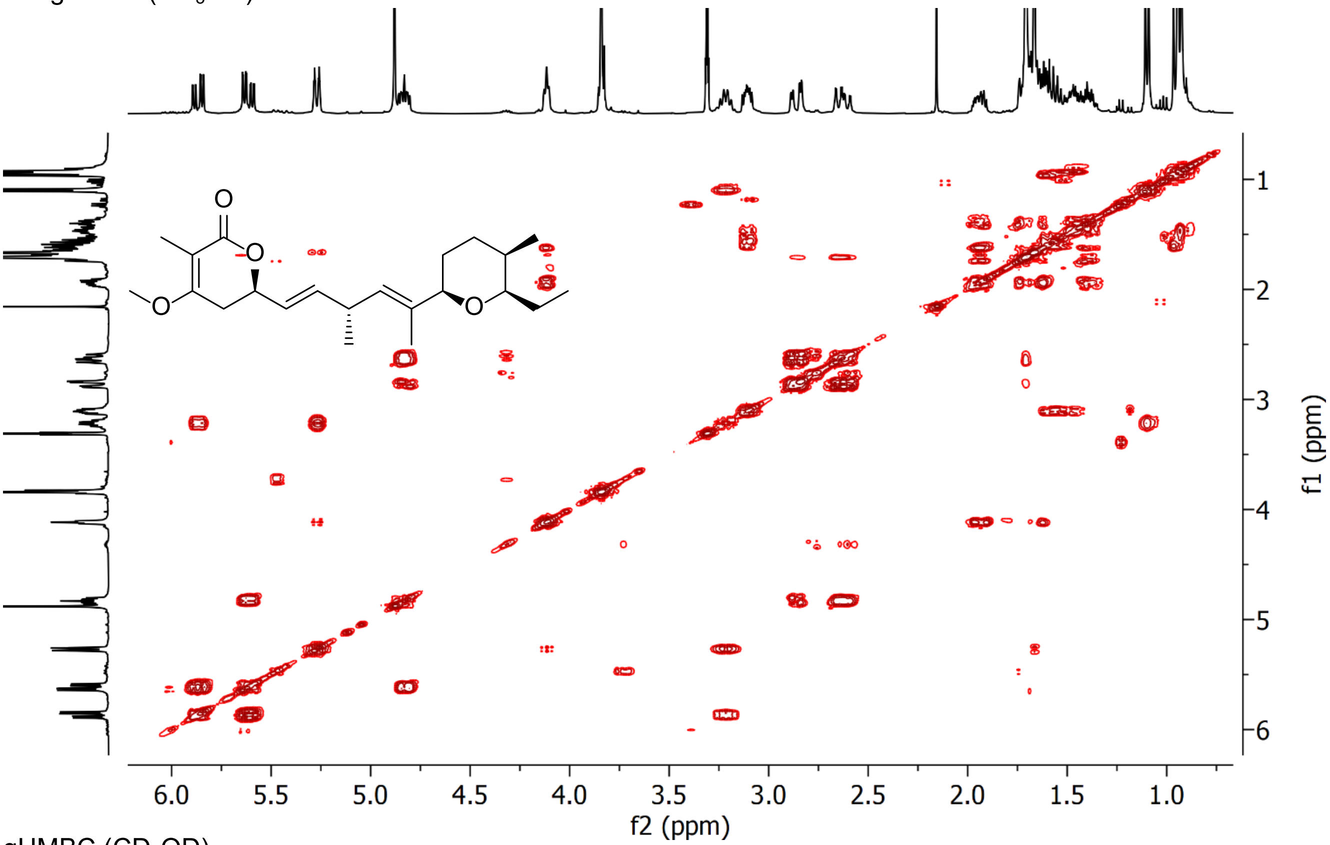

gHMBC (CD_3OD)

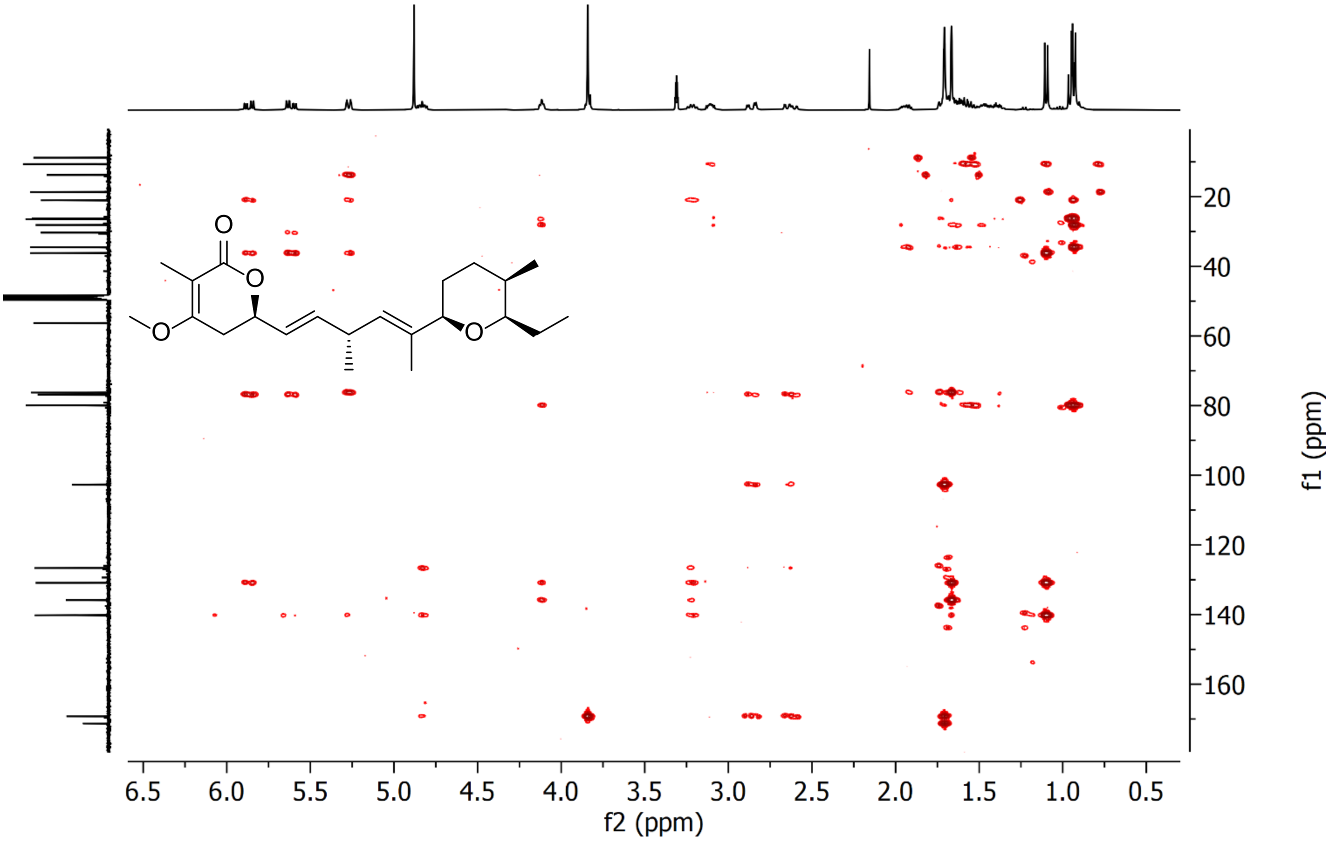

gHSQC-DEPT (CD_3OD)

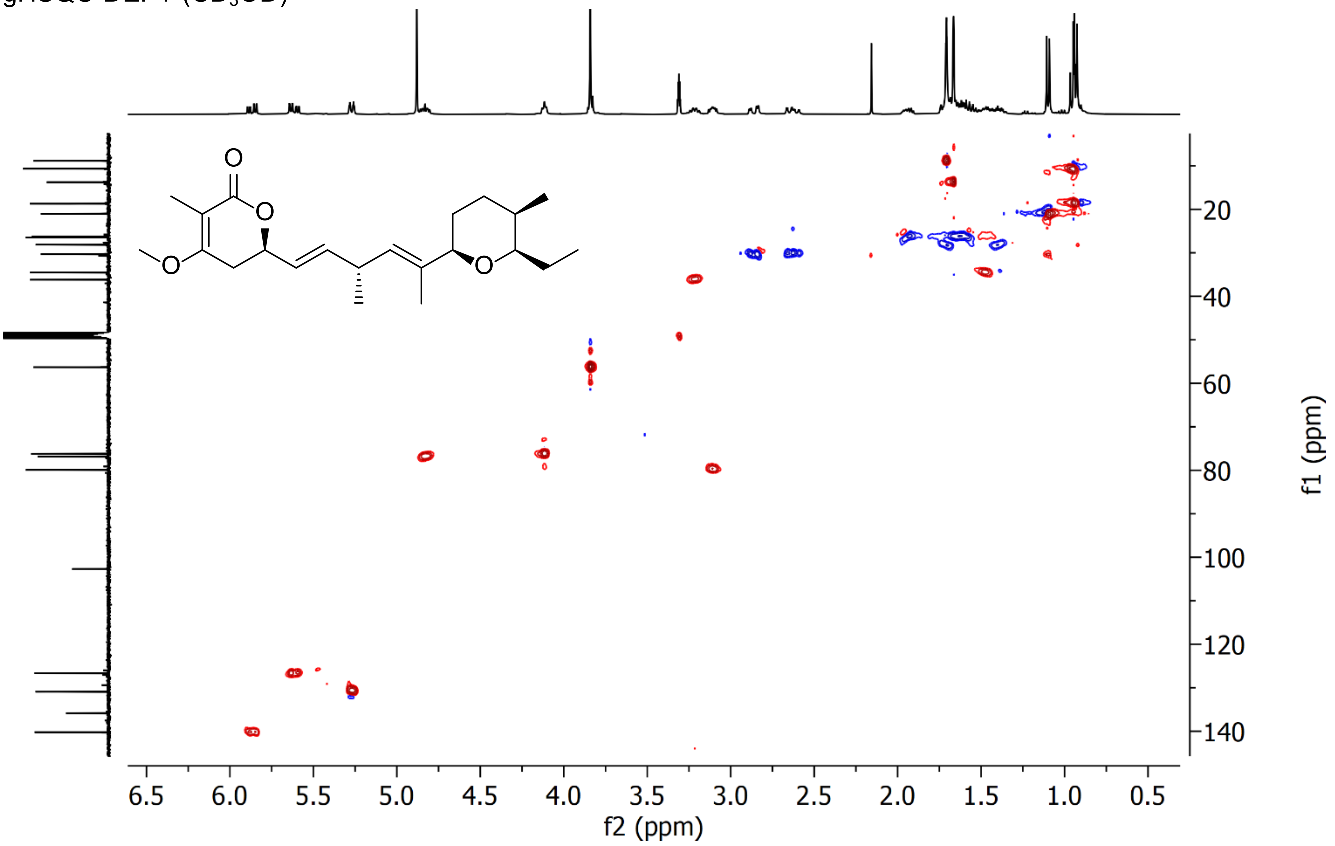

(R)-6-((R,1E,4E)-5-((2R,5R,6R)-6-Ethyl-5-methyltetrahydro-2H-pyran-2-yl)-3-methylhexa-1,4-dien-1-yl)-4-methoxy-3-(((4-methoxybenzyl)oxy)methyl)-5,6-dihydro-2H-pyran-2-one 56

^{1}H-NMR (400 MHz, $CDCl_3$)

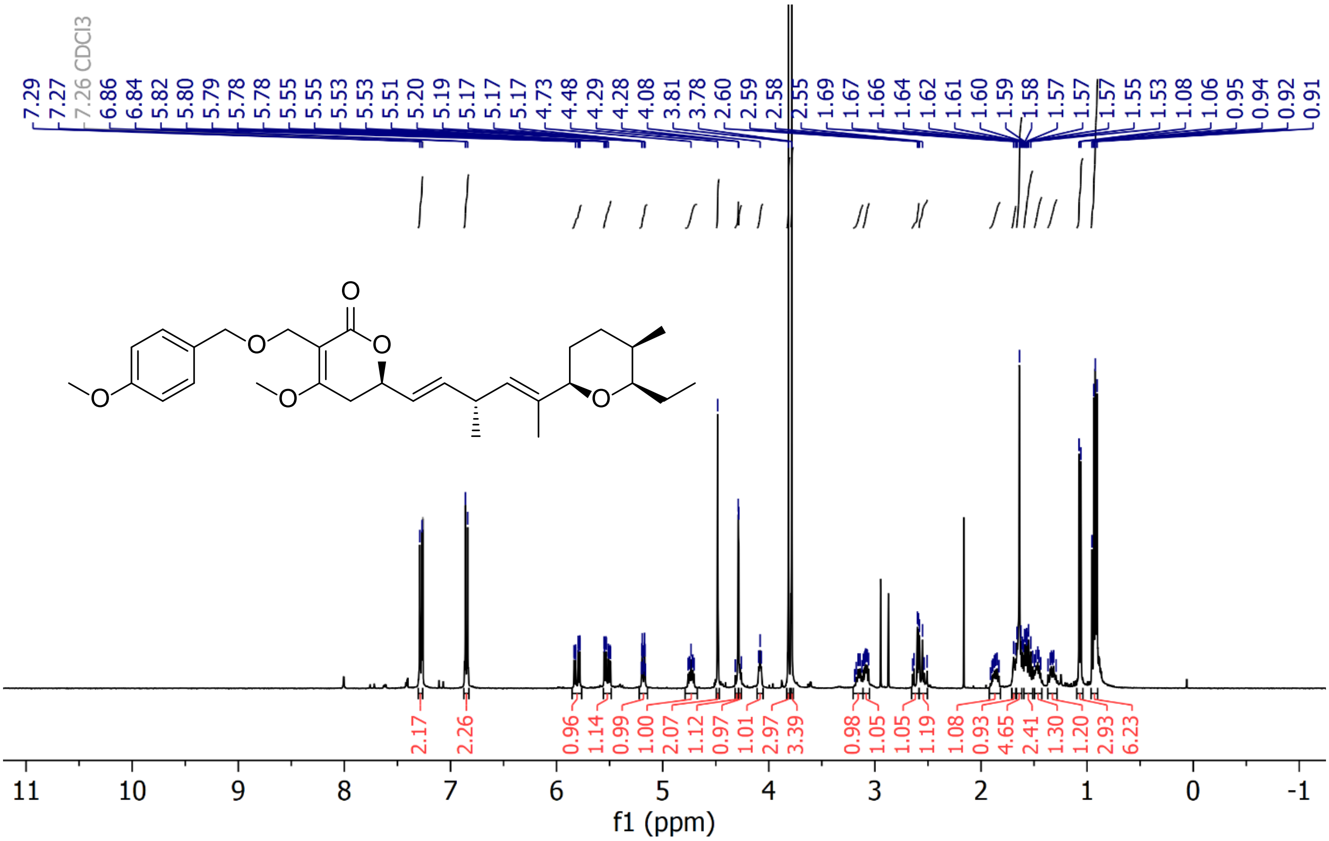

^{13}C-NMR (100 MHz, $CDCl_3$)

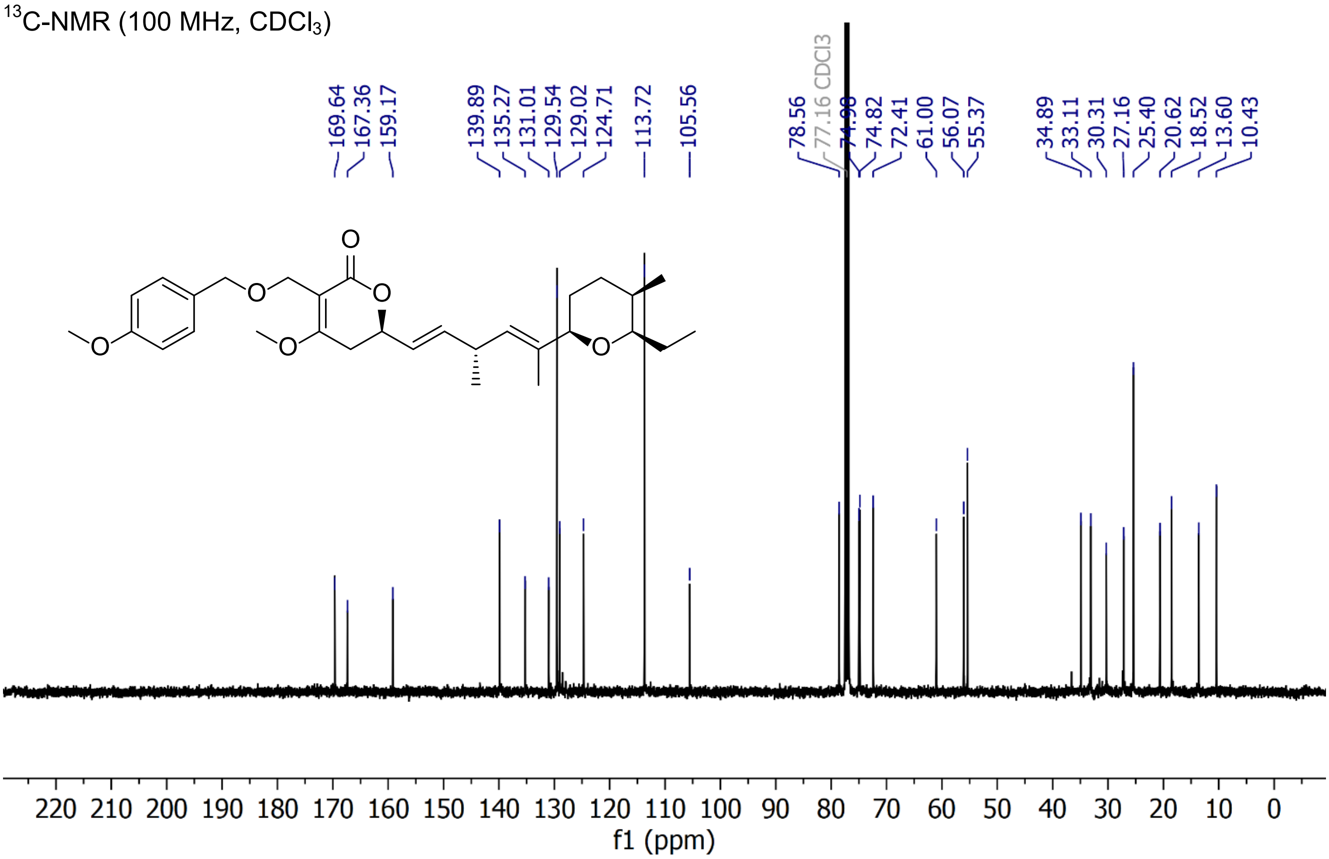

14-*epi* jerangolid H 57

^{1}H-NMR (400 MHz, CD_3OD)

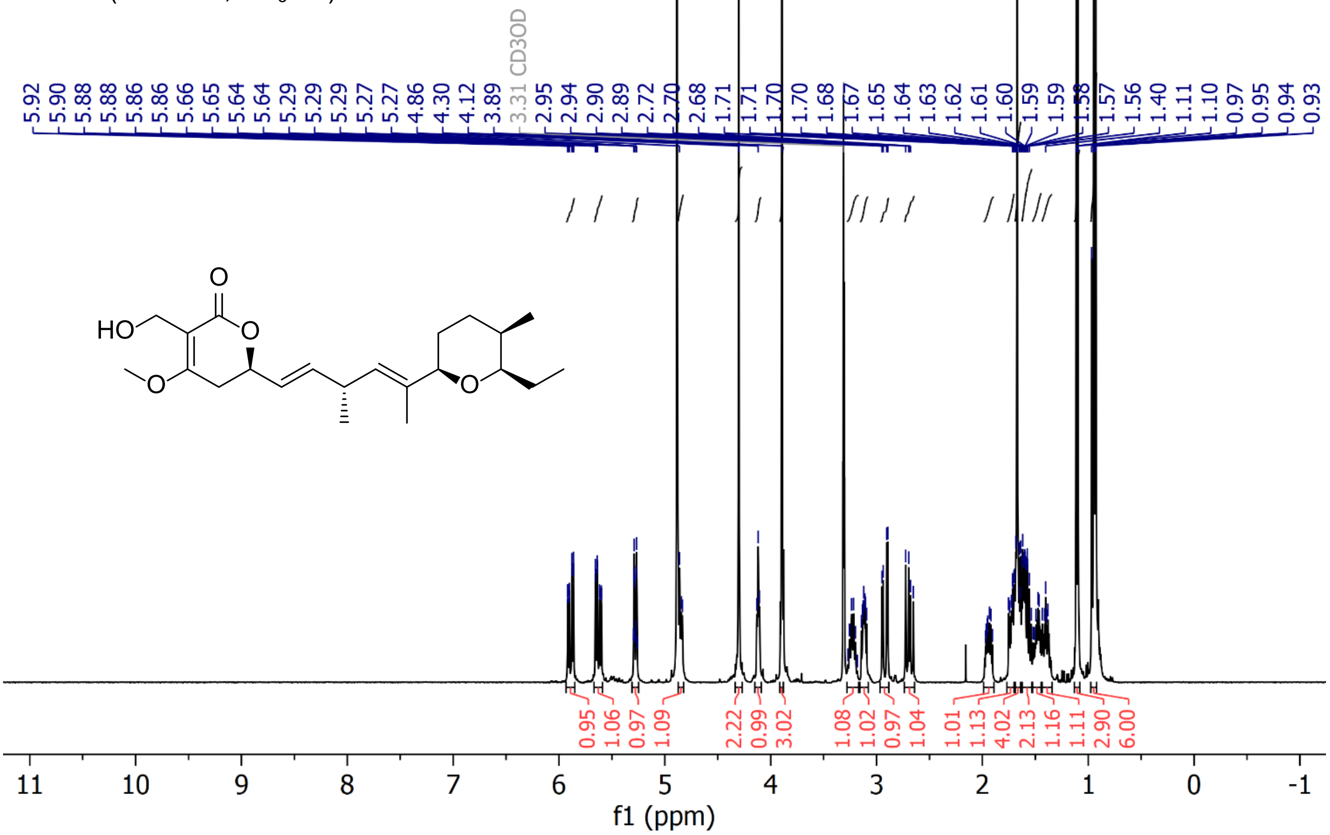

^{13}C-NMR (100 MHz, CD_3OD)

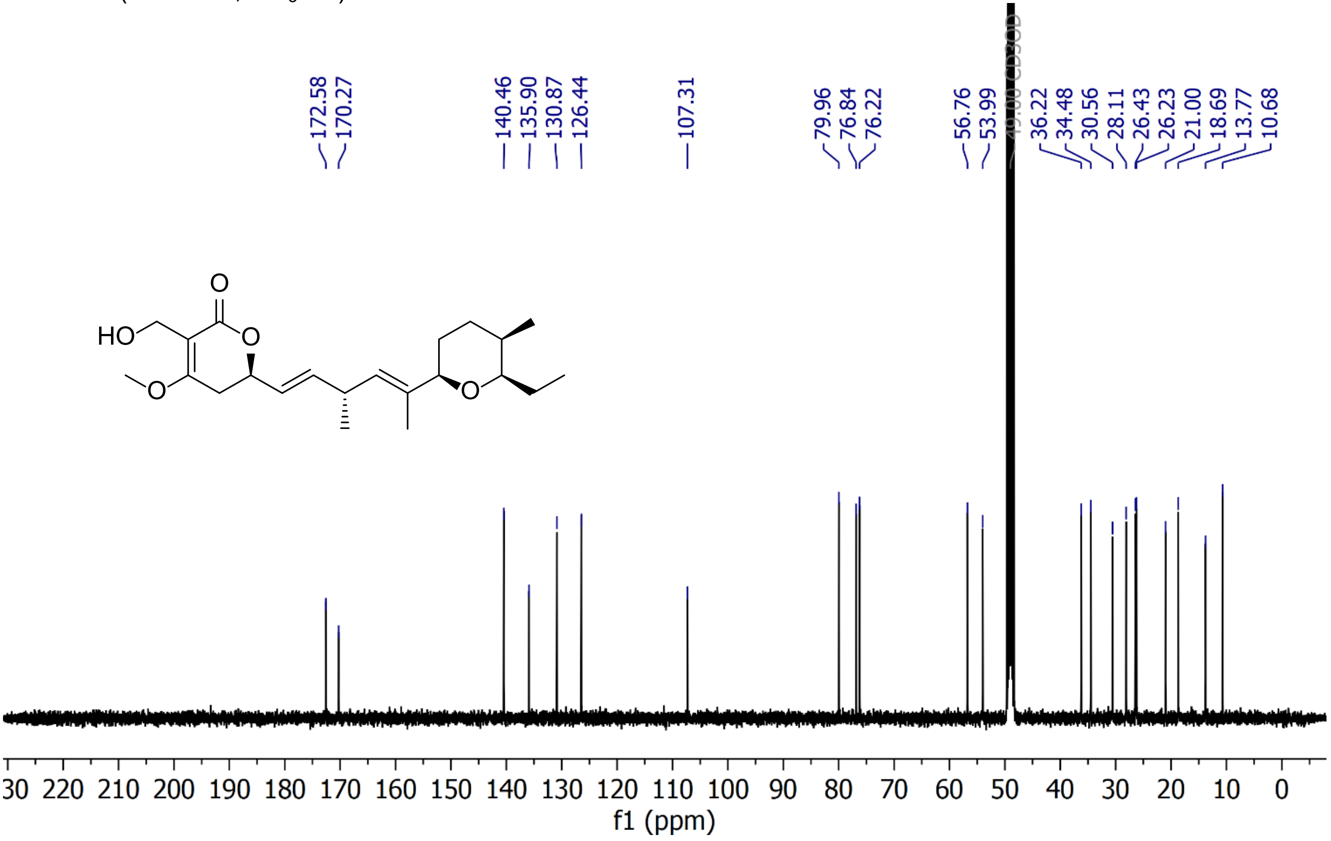

HH-gCOSY (CD_3OD)

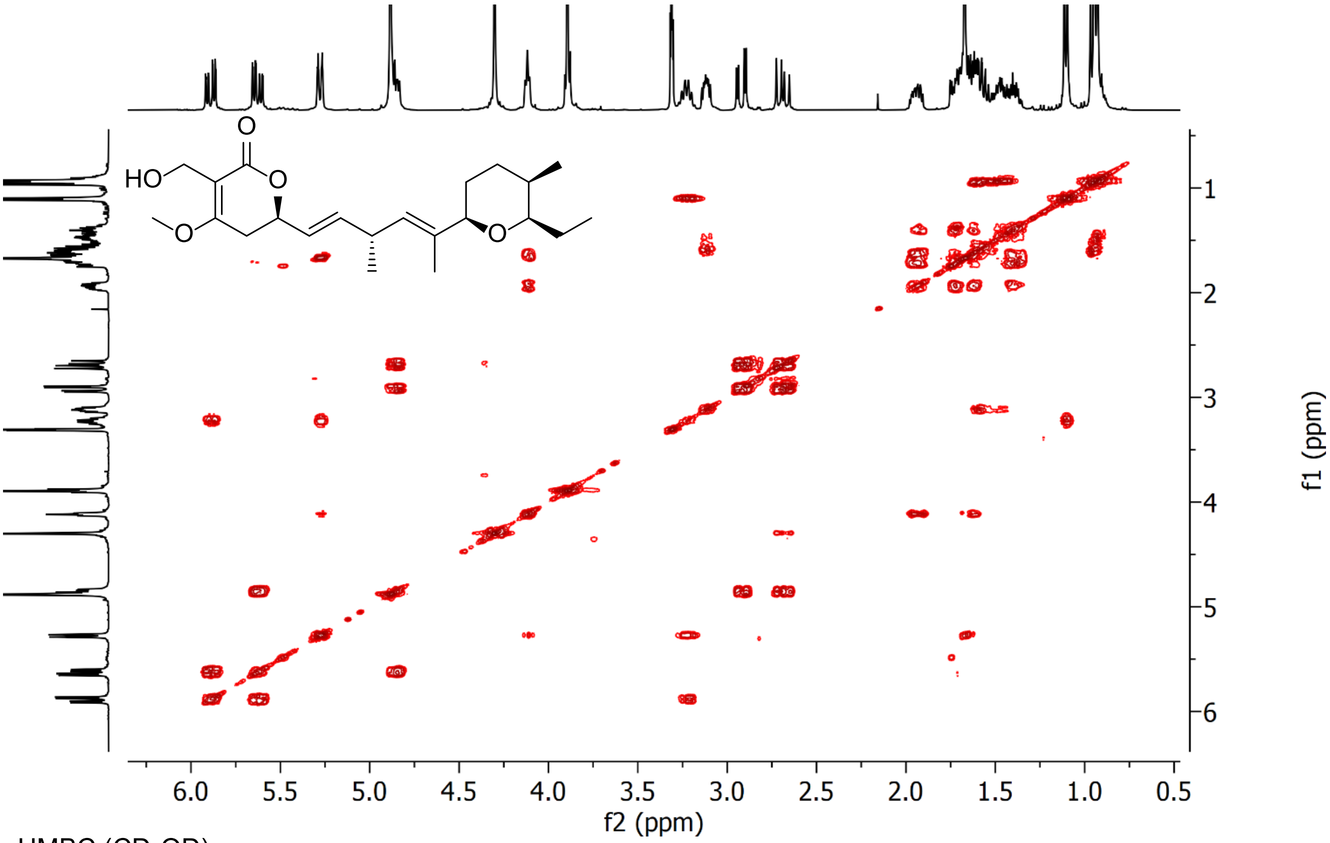

gHMBC (CD_3OD)

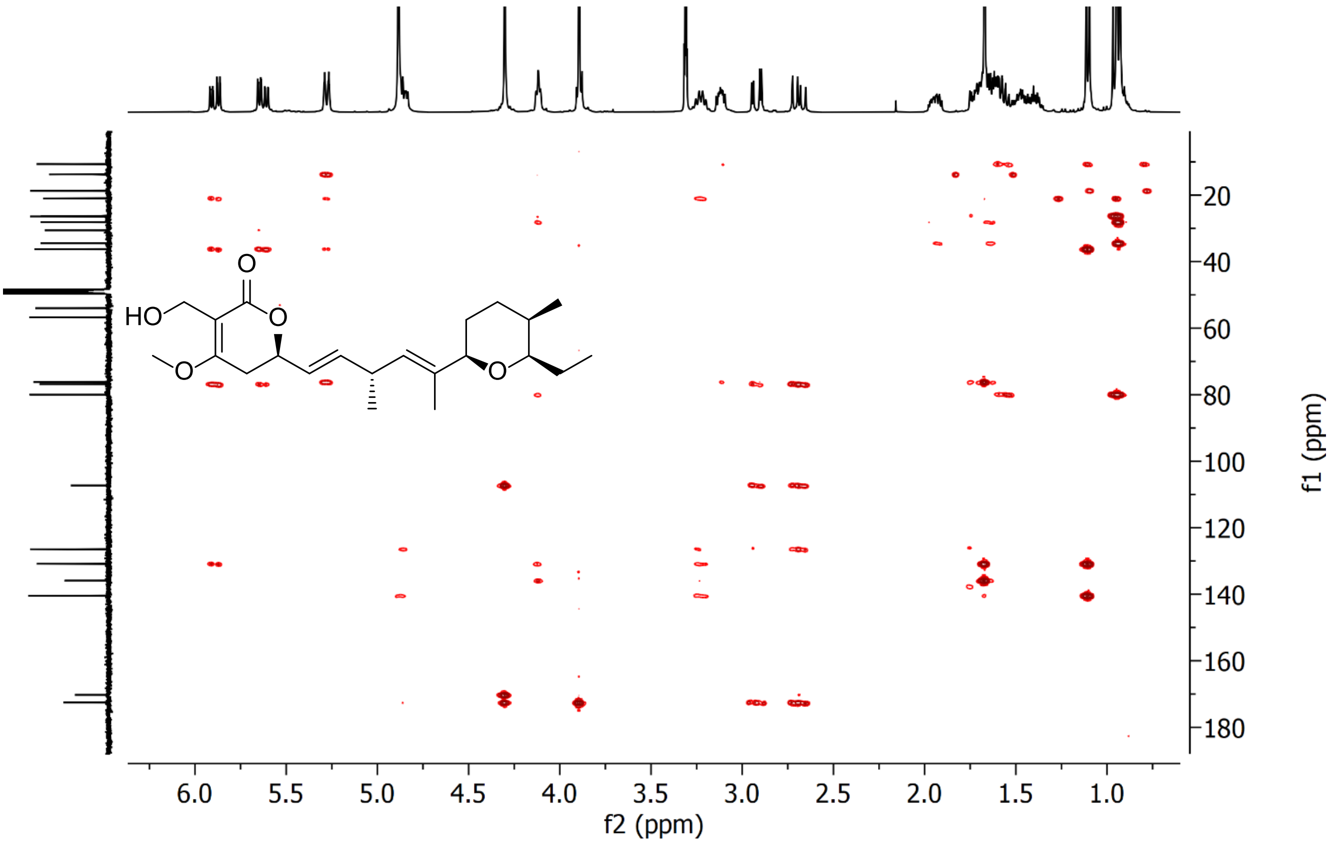

gHSQC-DEPT (CD_3OD)

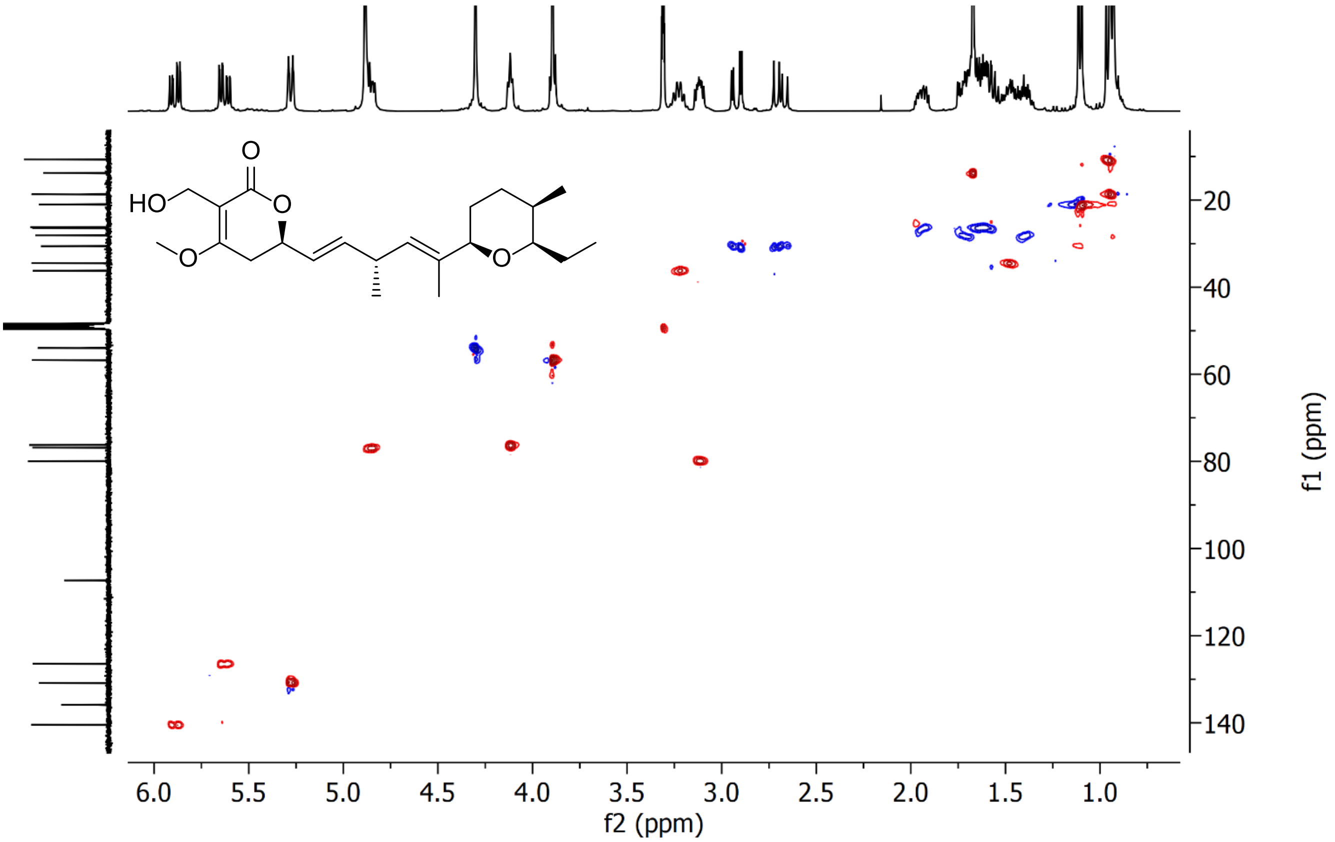

jerangolid E 2

^{1}H-NMR (500 MHz, CD_3OD)

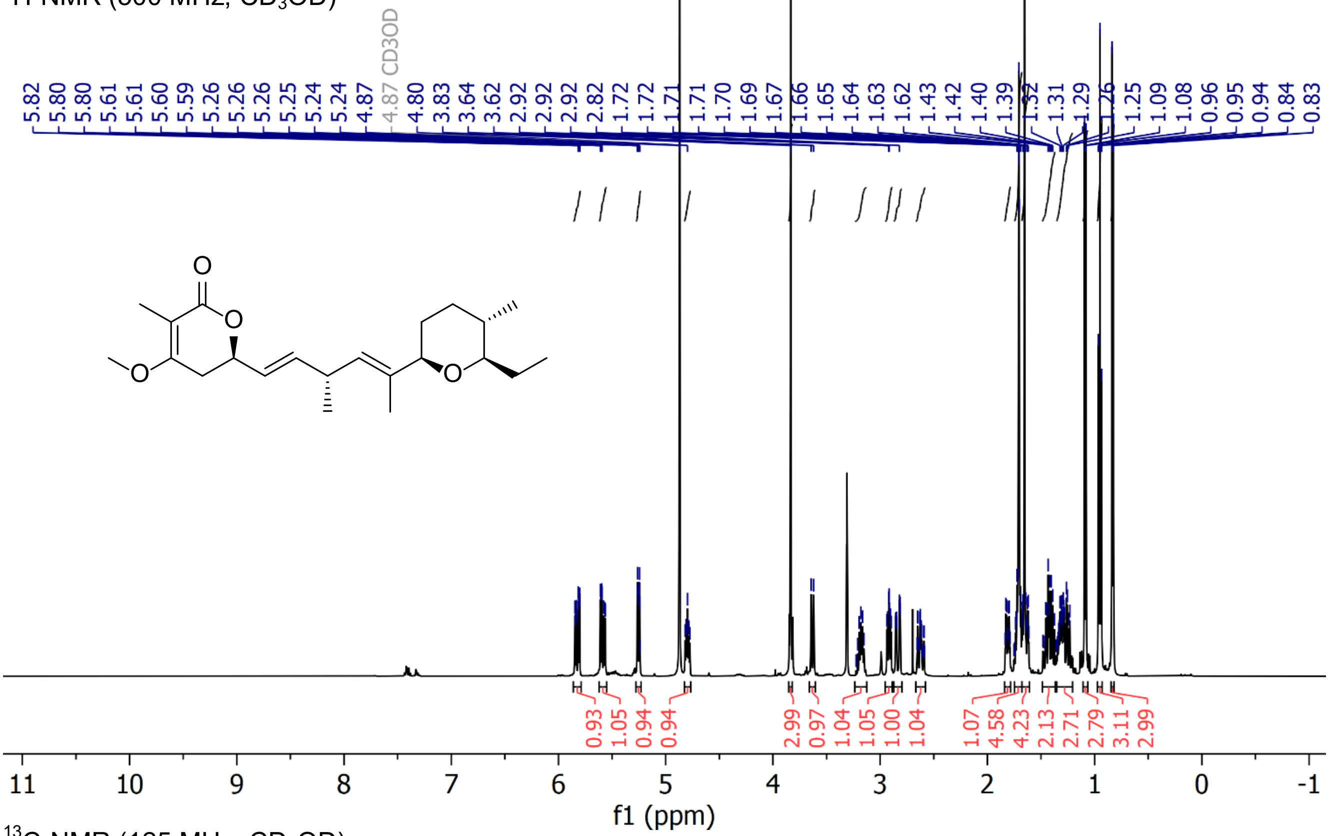

^{13}C-NMR (125 MHz, CD_3OD)

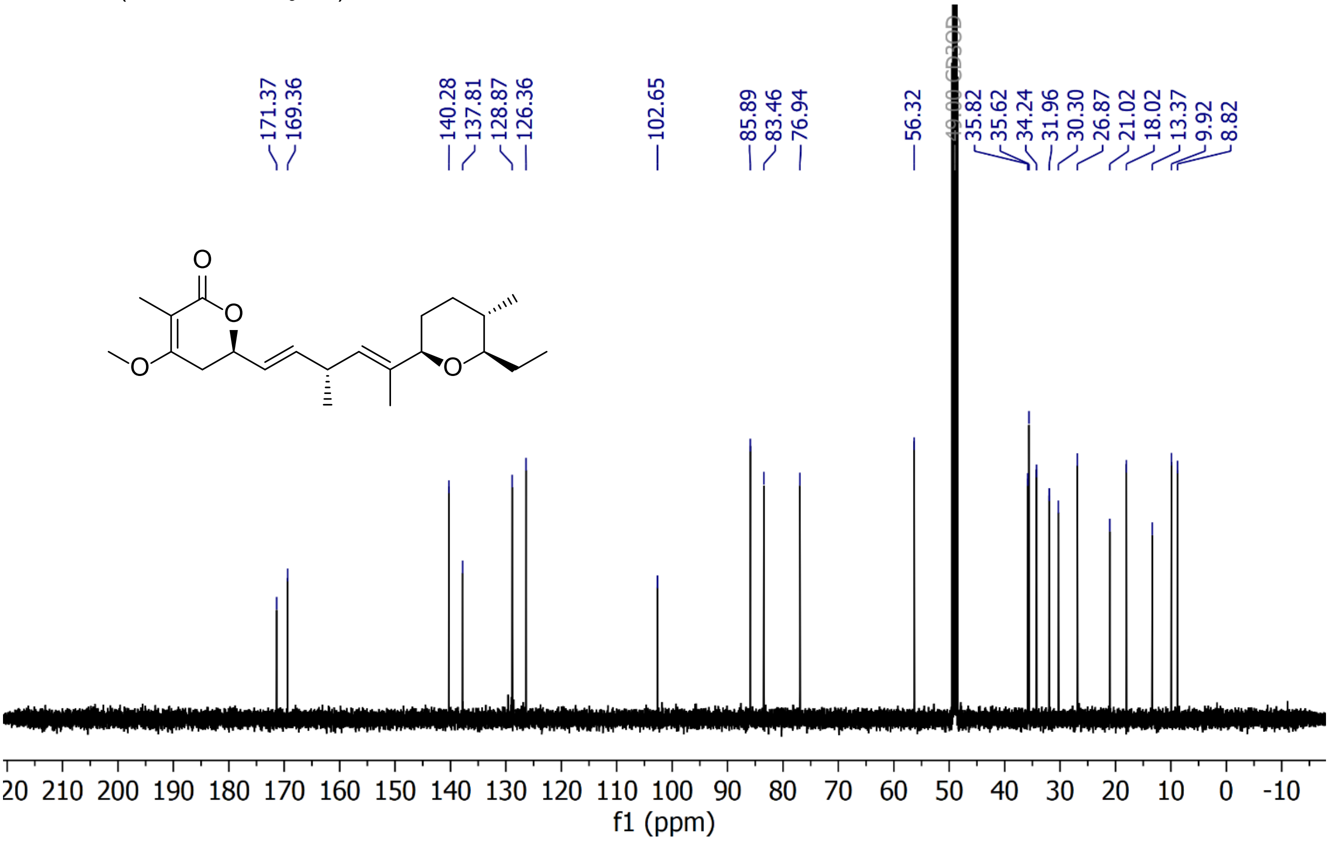

HH-gCOSY (CD_3OD)

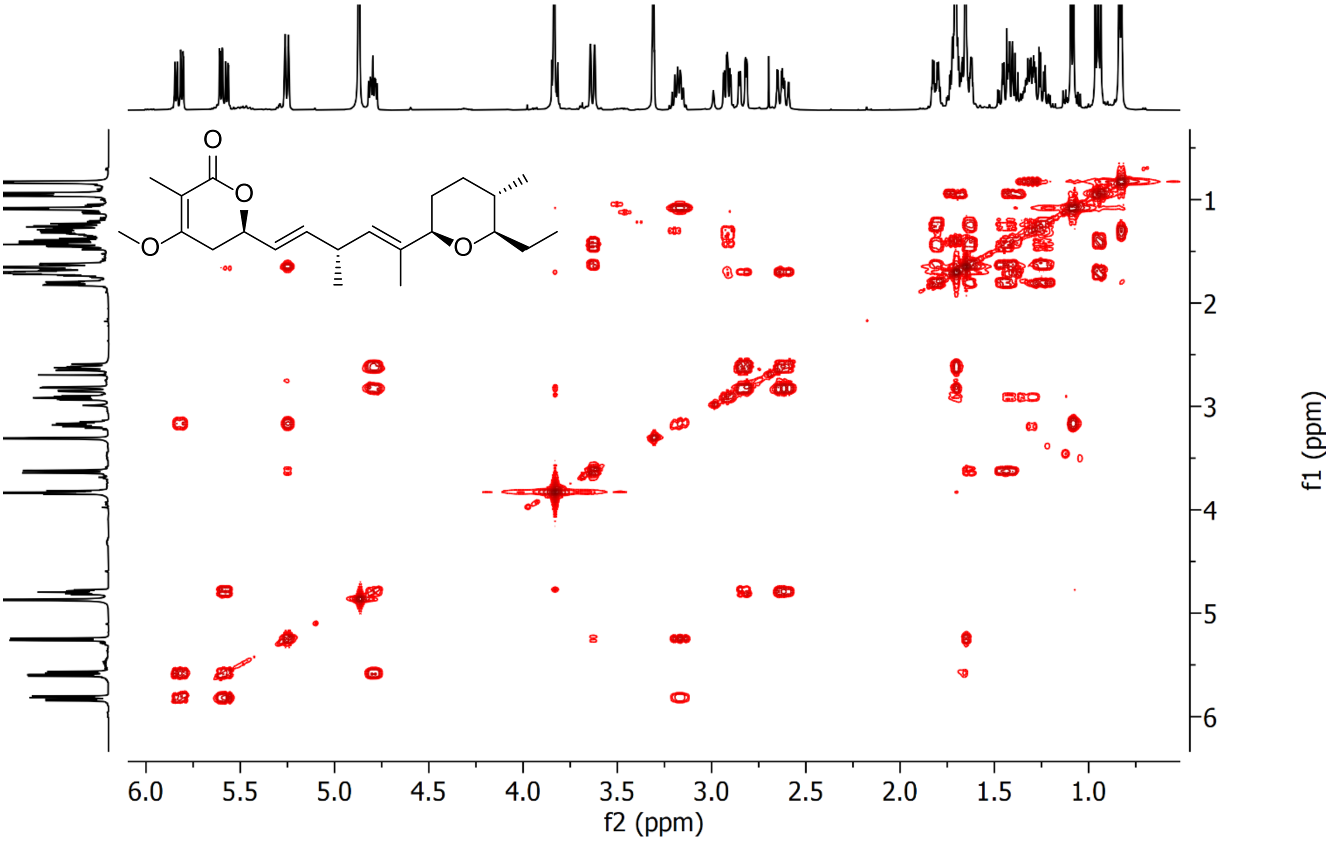

gHMBC (CD_3OD)

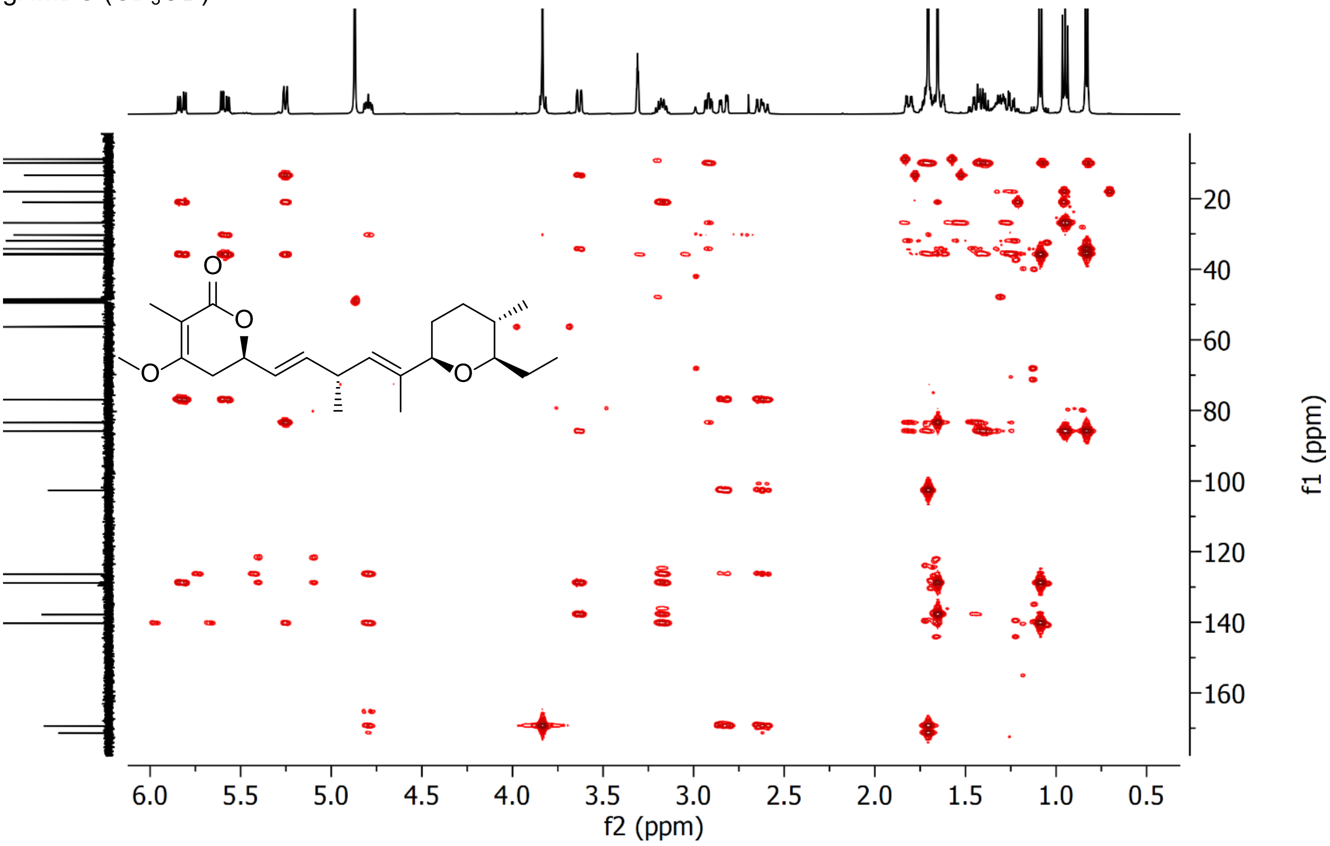

gHSQC-DEPT (CD_3OD)

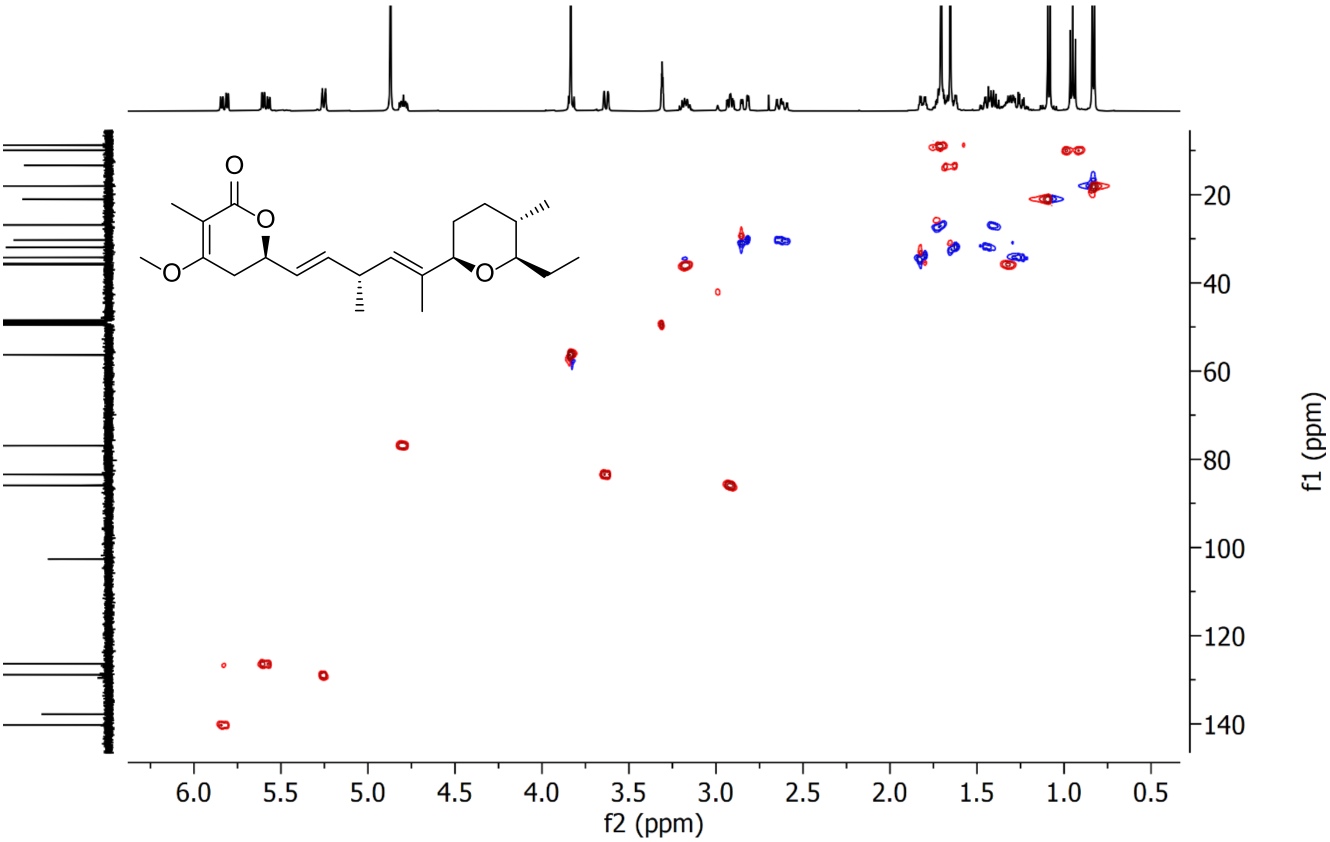

(R)-6-((R,1E,4E)-5-((2R,5S,6R)-6-Ethyl-5-methyltetrahydro-2H-pyran-2-yl)-3-methylhexa-1,4-dien-1-yl)-4-methoxy-3-(((4-methoxybenzyl)oxy)methyl)-5,6-dihydro-2H-pyran-2-one 58

^{1}H-NMR (500 MHz, CD_3OD)

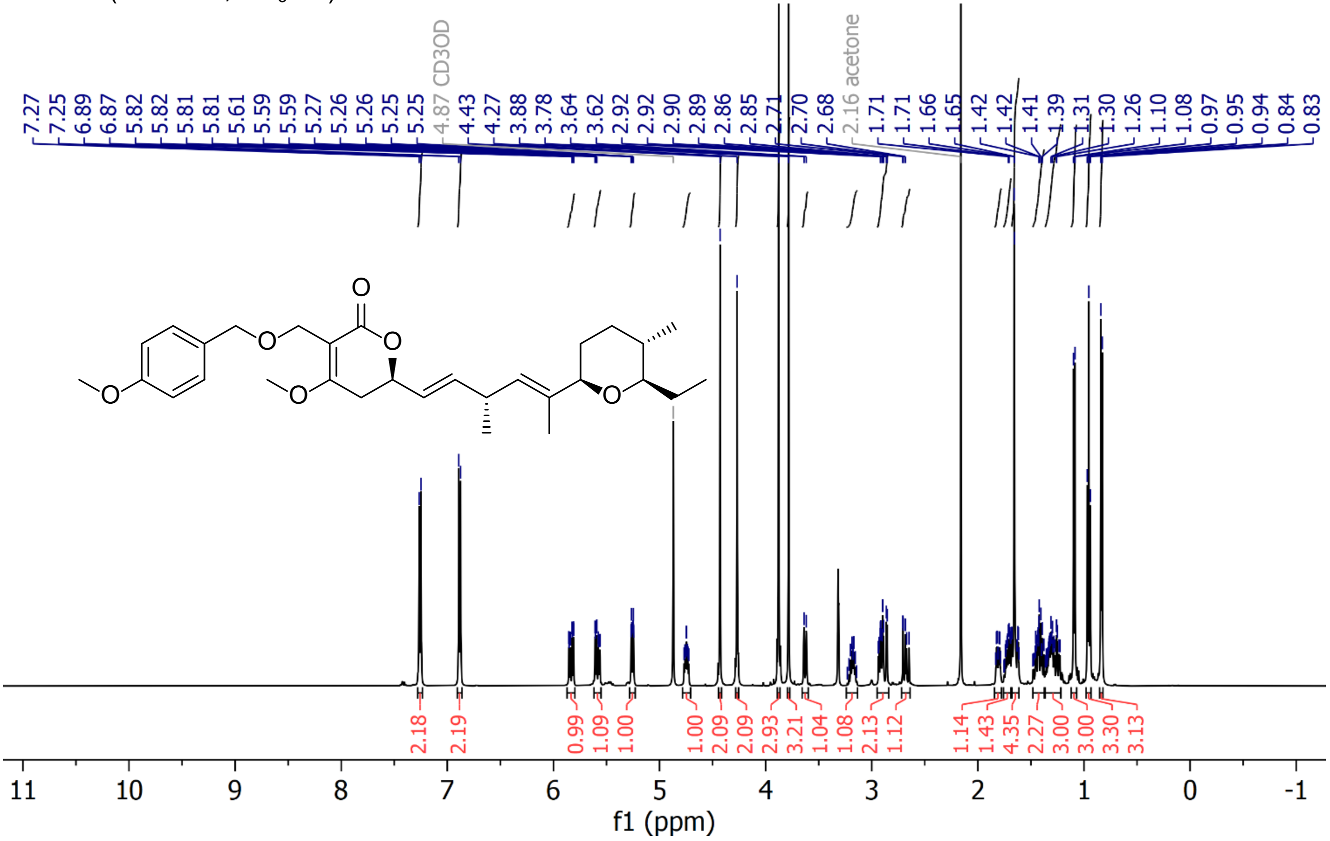

^{13}C-NMR (125 MHz, CD_3OD)

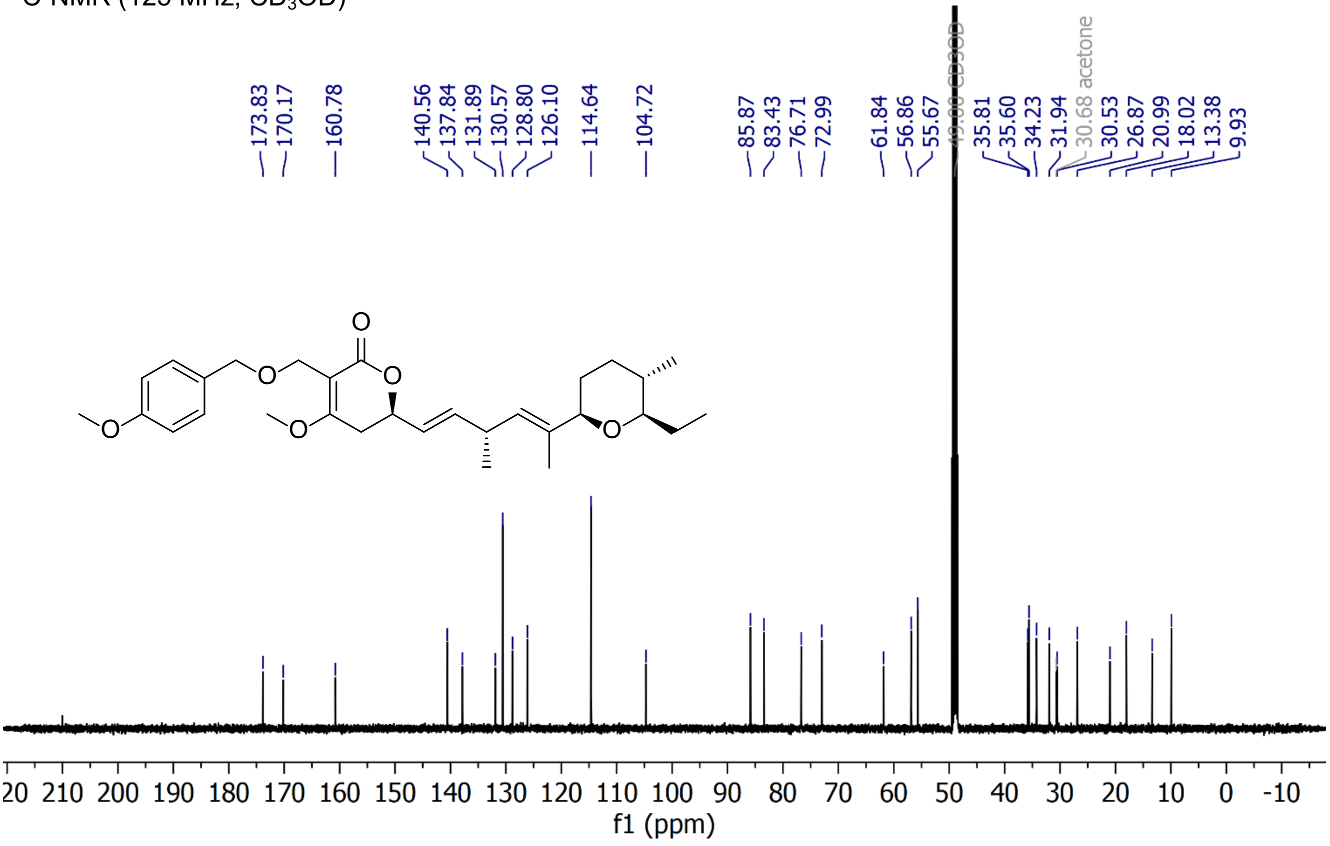

Jerangolid H 3

^{1}H-NMR (500 MHz, CD_3OD)

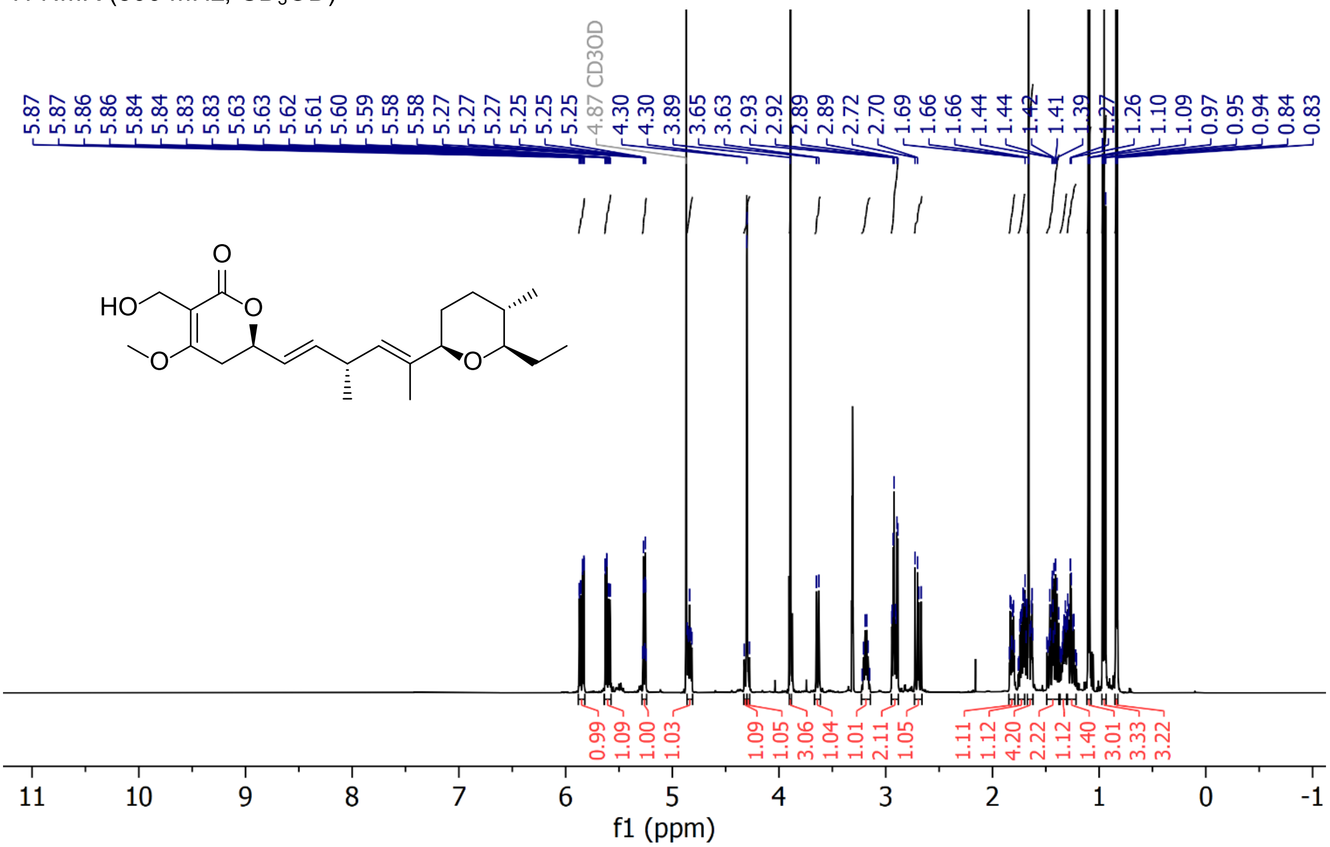

^{13}C-NMR (125 MHz, CD_3OD)

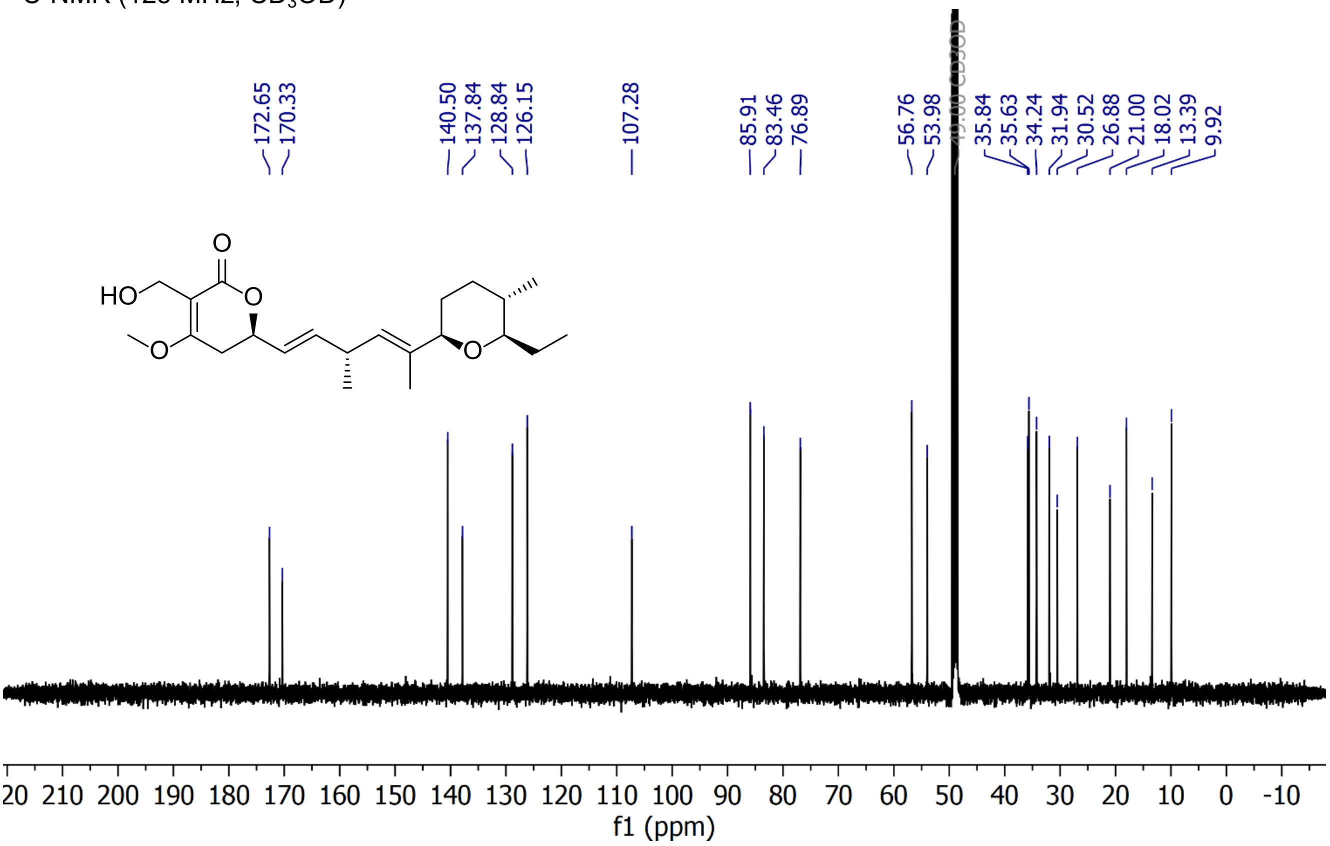

HH-gCOSY (CD_3OD)

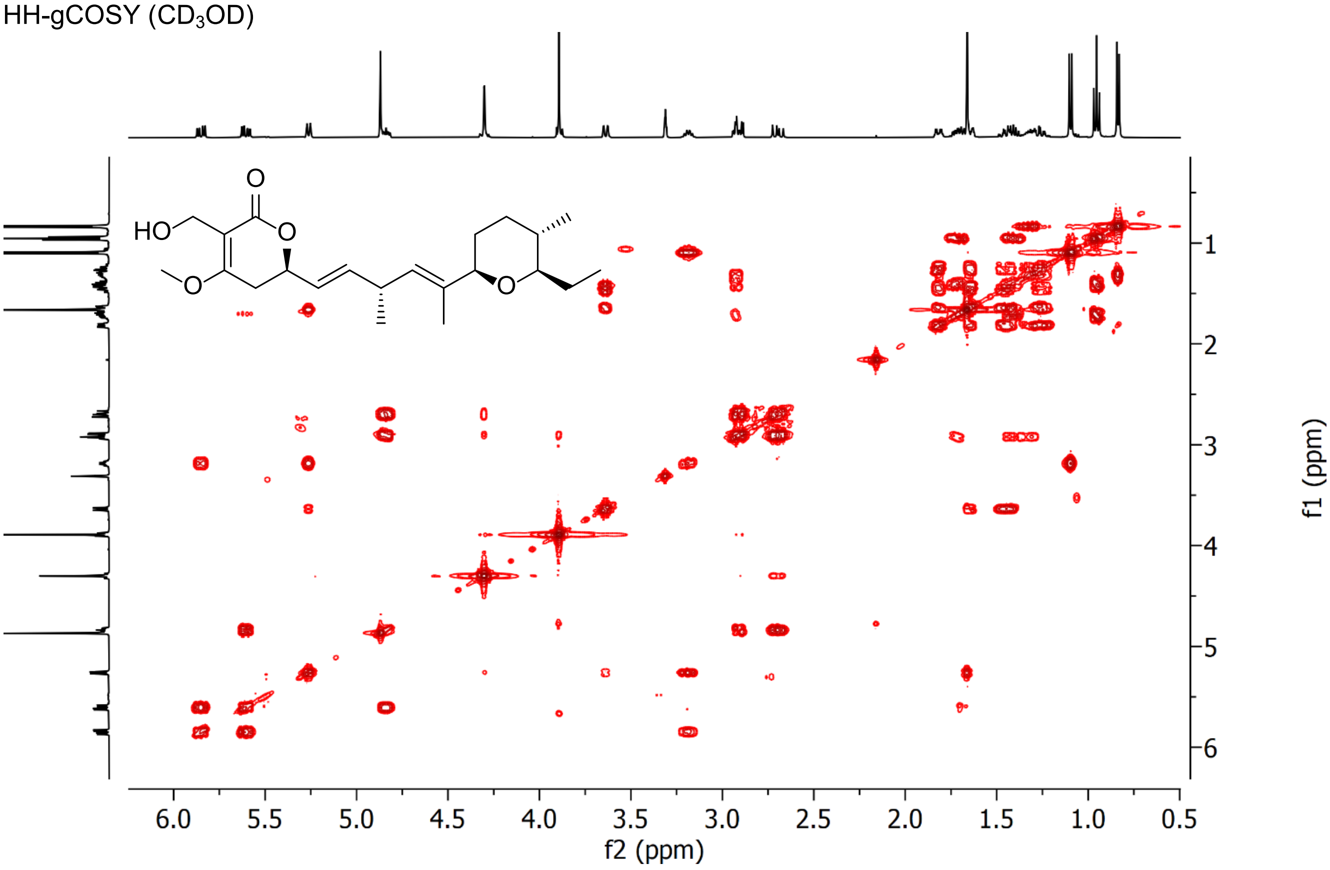

gHMBC (CD_3OD)

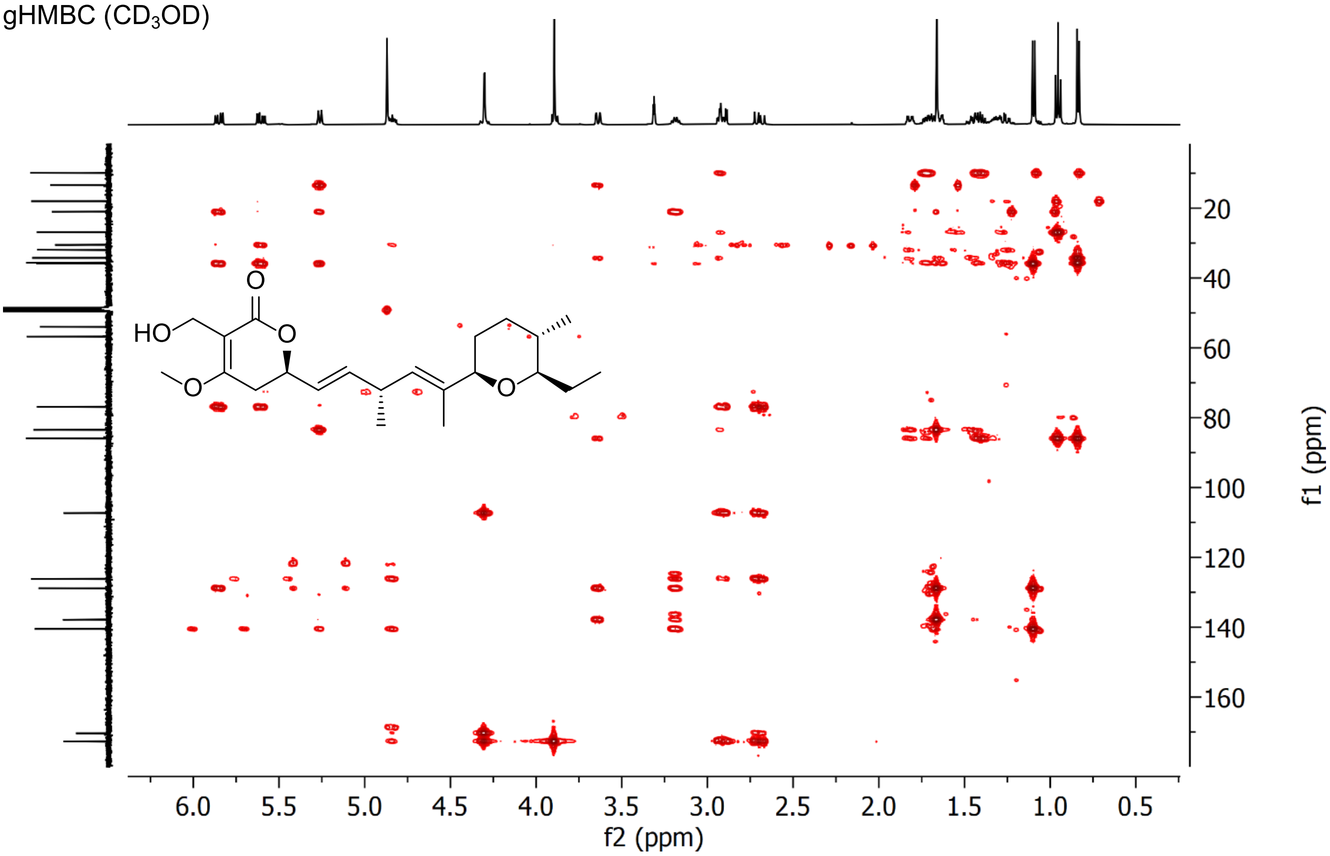

gHSQC-DEPT (CD_3OD)

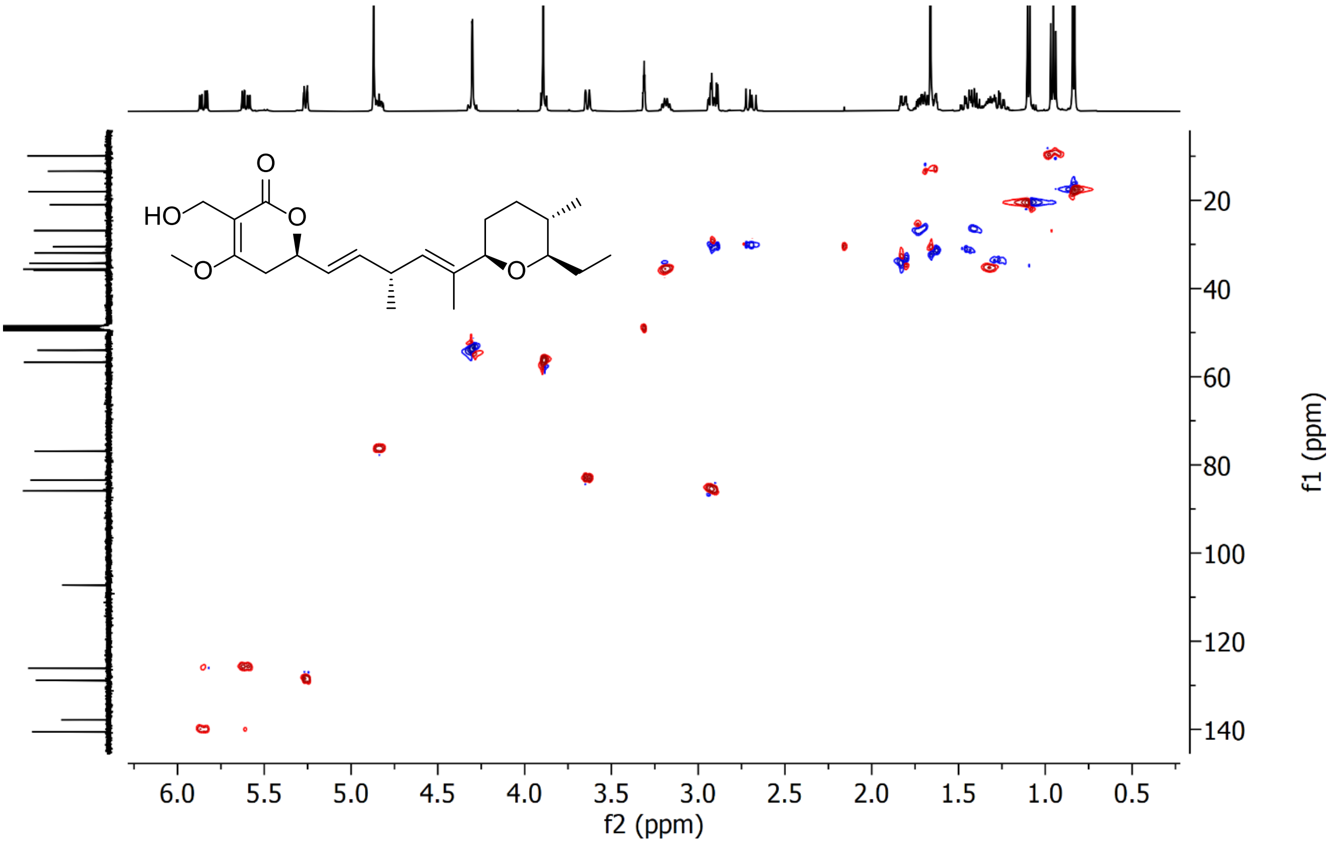

Jerangolid D 4

^{1}H-NMR (500 MHz, CD_3OD)

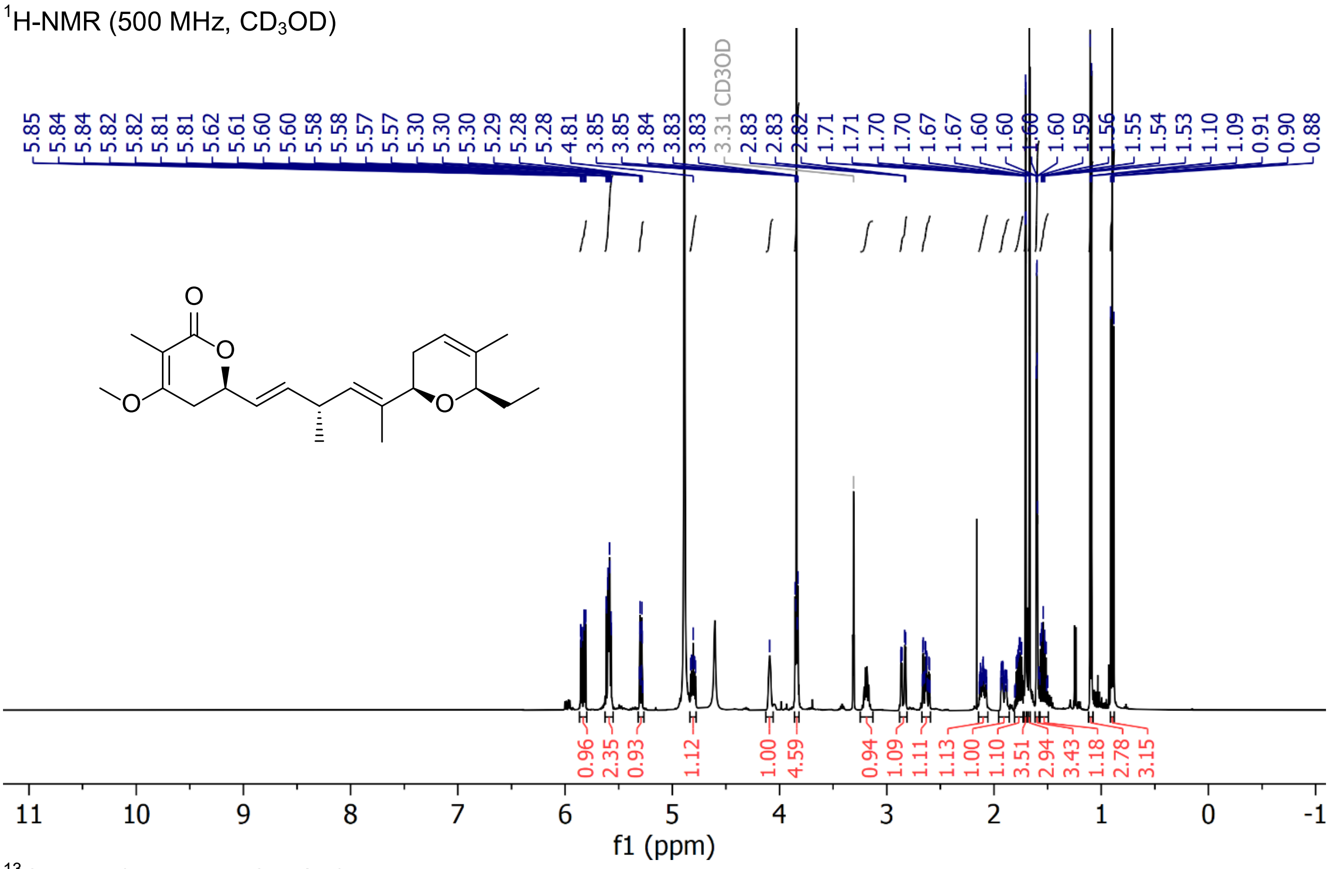

^{13}C-NMR (125 MHz, CD_3OD)

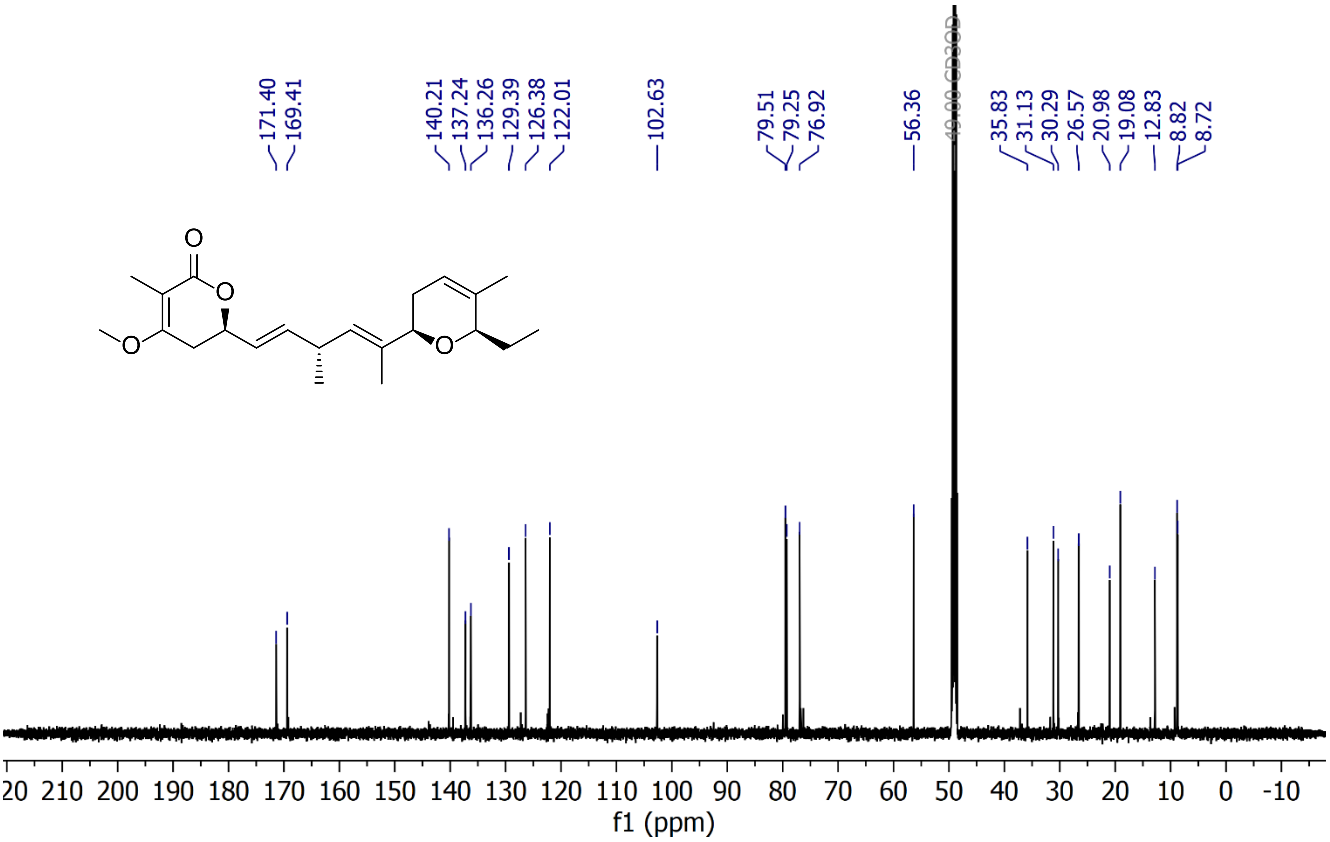

HH-gCOSY (CD_3OD)

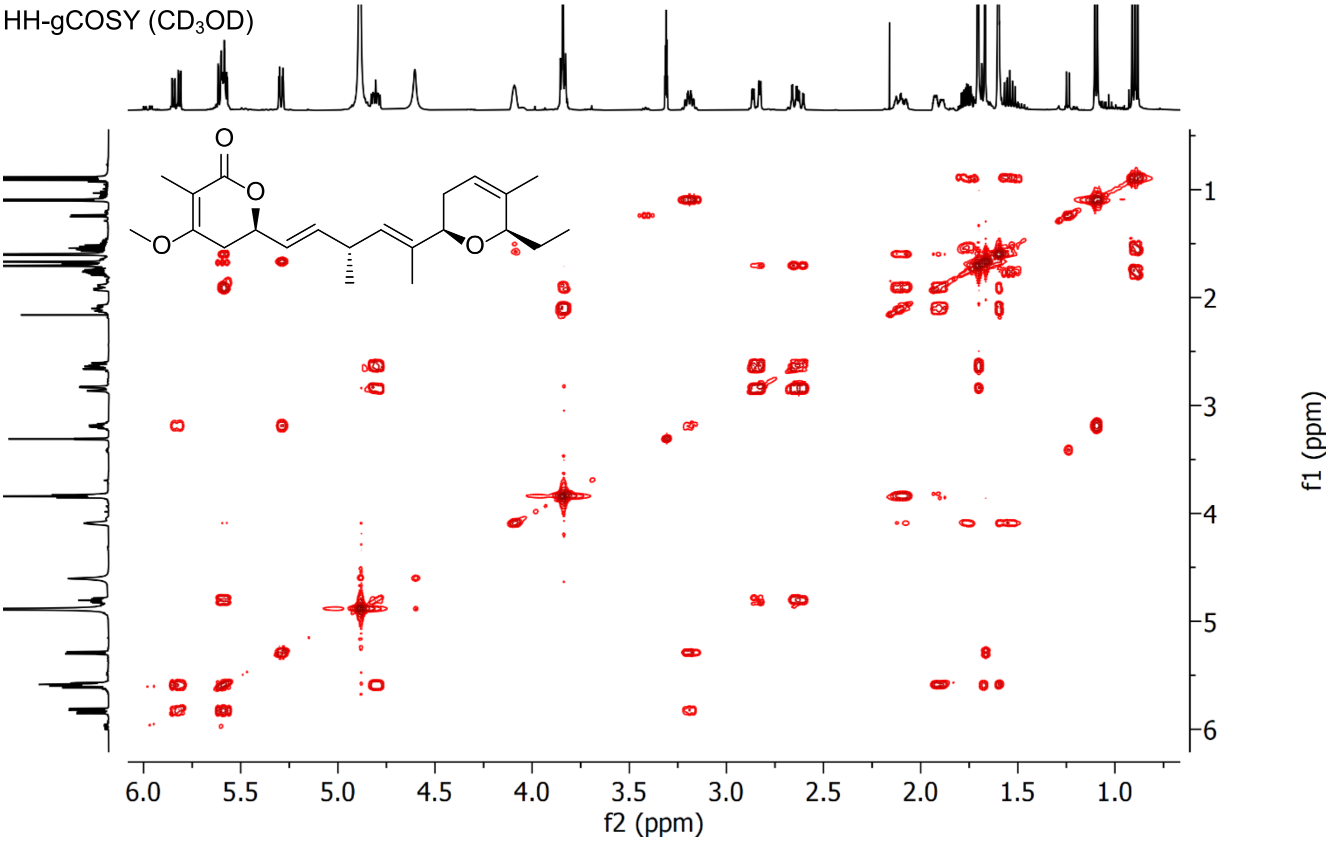

gHMBC (CD_3OD)

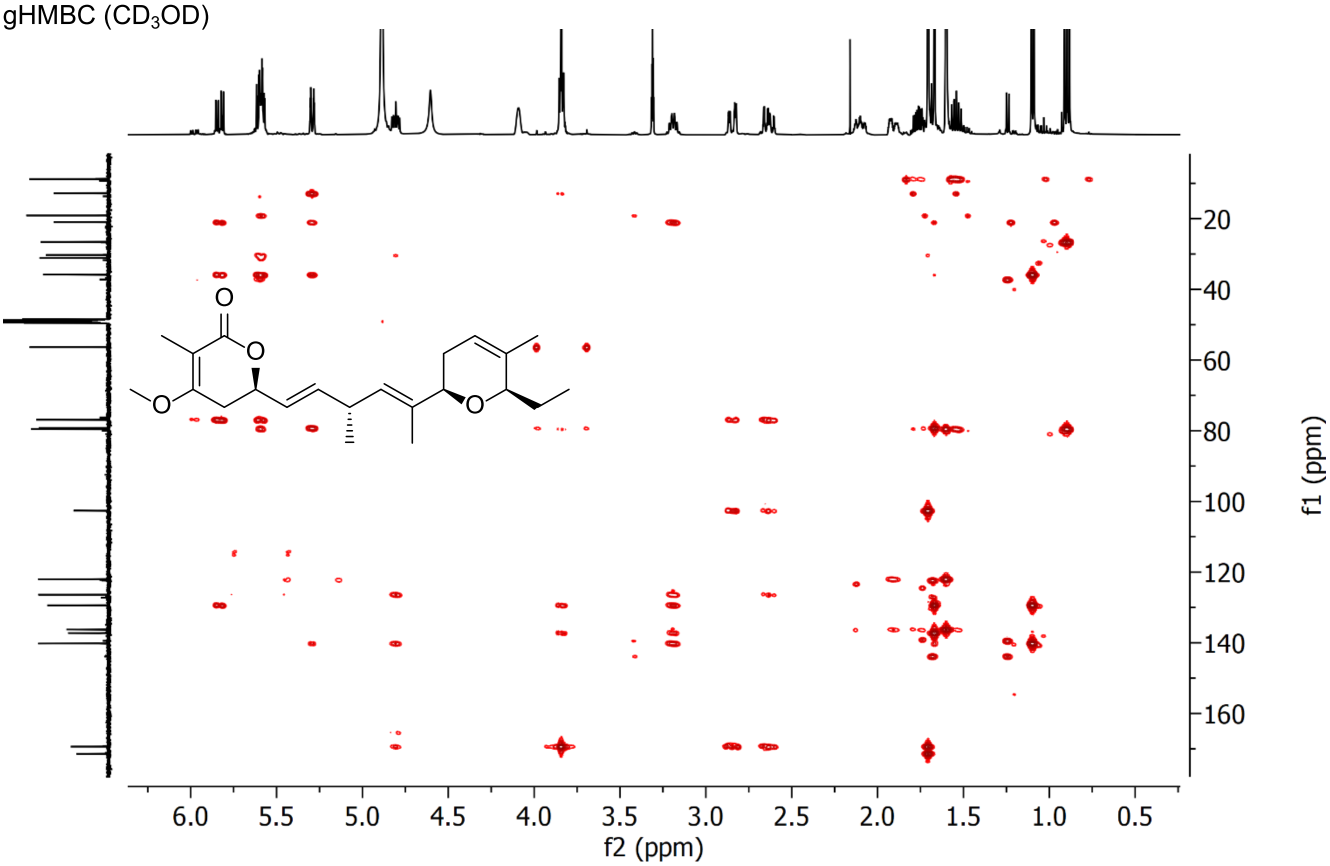

gHSQC-DEPT (CD_3OD)

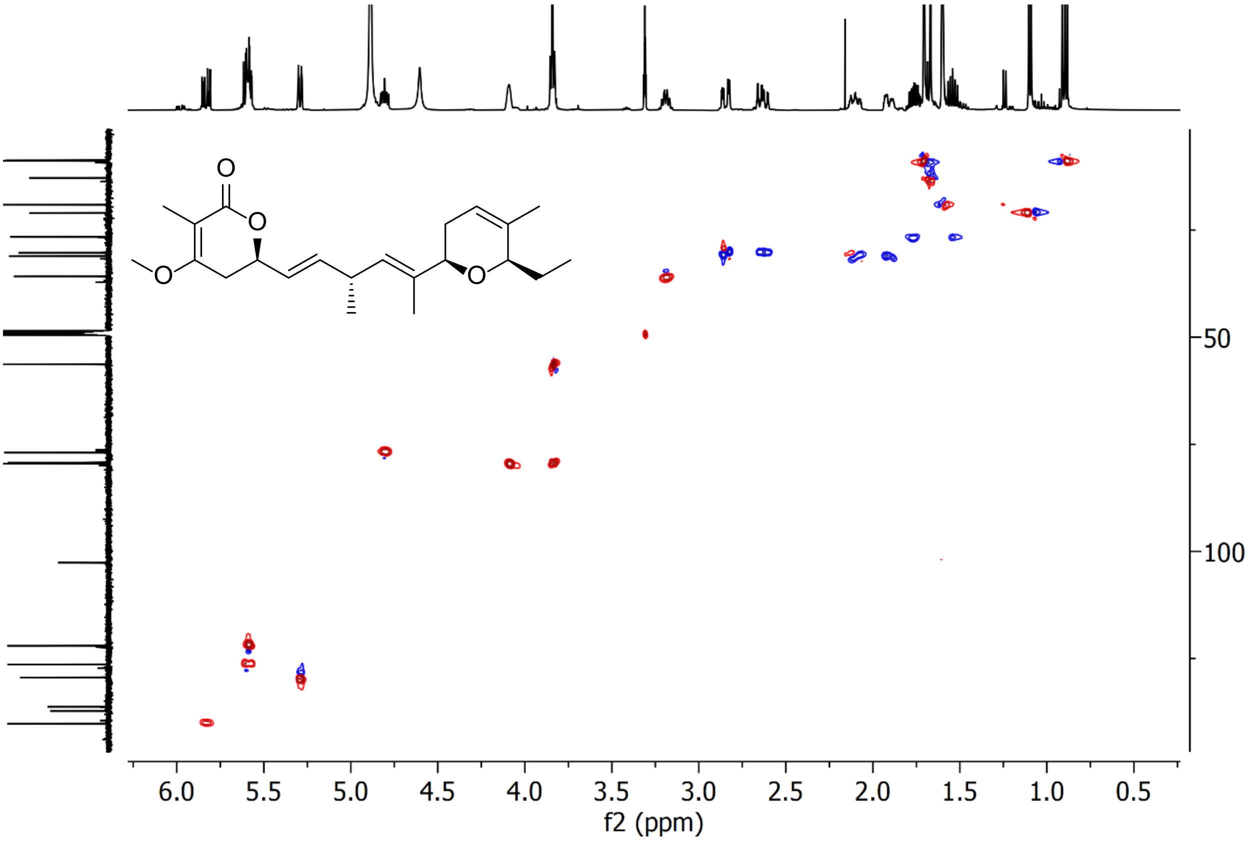

(R)-6-((R,1E,4E)-5-((2R,6R)-6-Ethyl-5-methyl-3,6-dihydro-2H-pyran-2-yl)-3-methylhexa-1,4-dien-1-yl)-4-methoxy-3-(((4-methoxybenzyl)oxy)methyl)-5,6-dihydro-2H-pyran-2-one 59

^{1}H-NMR (400 MHz, $CDCl_3$)

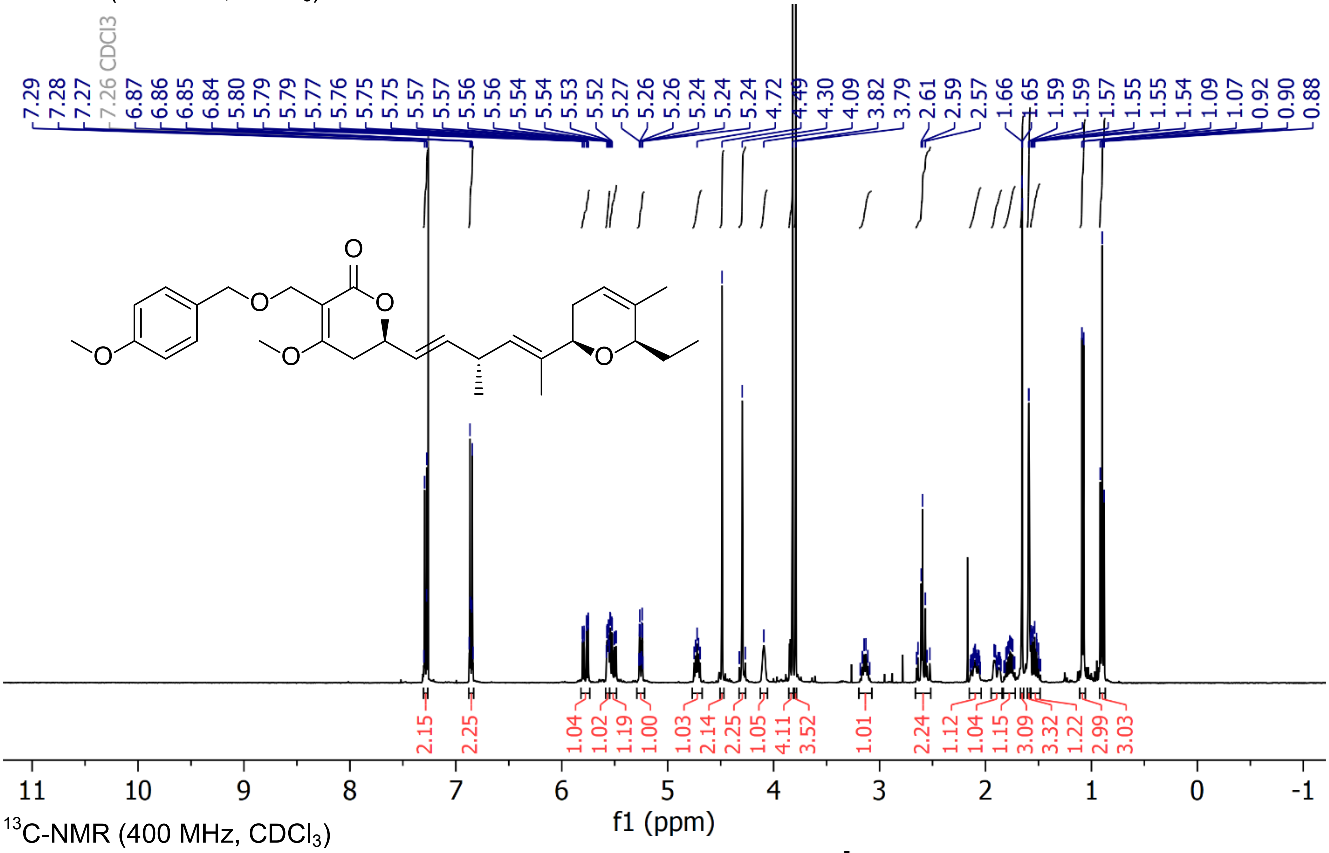

^{13}C-NMR (400 MHz, $CDCl_3$)

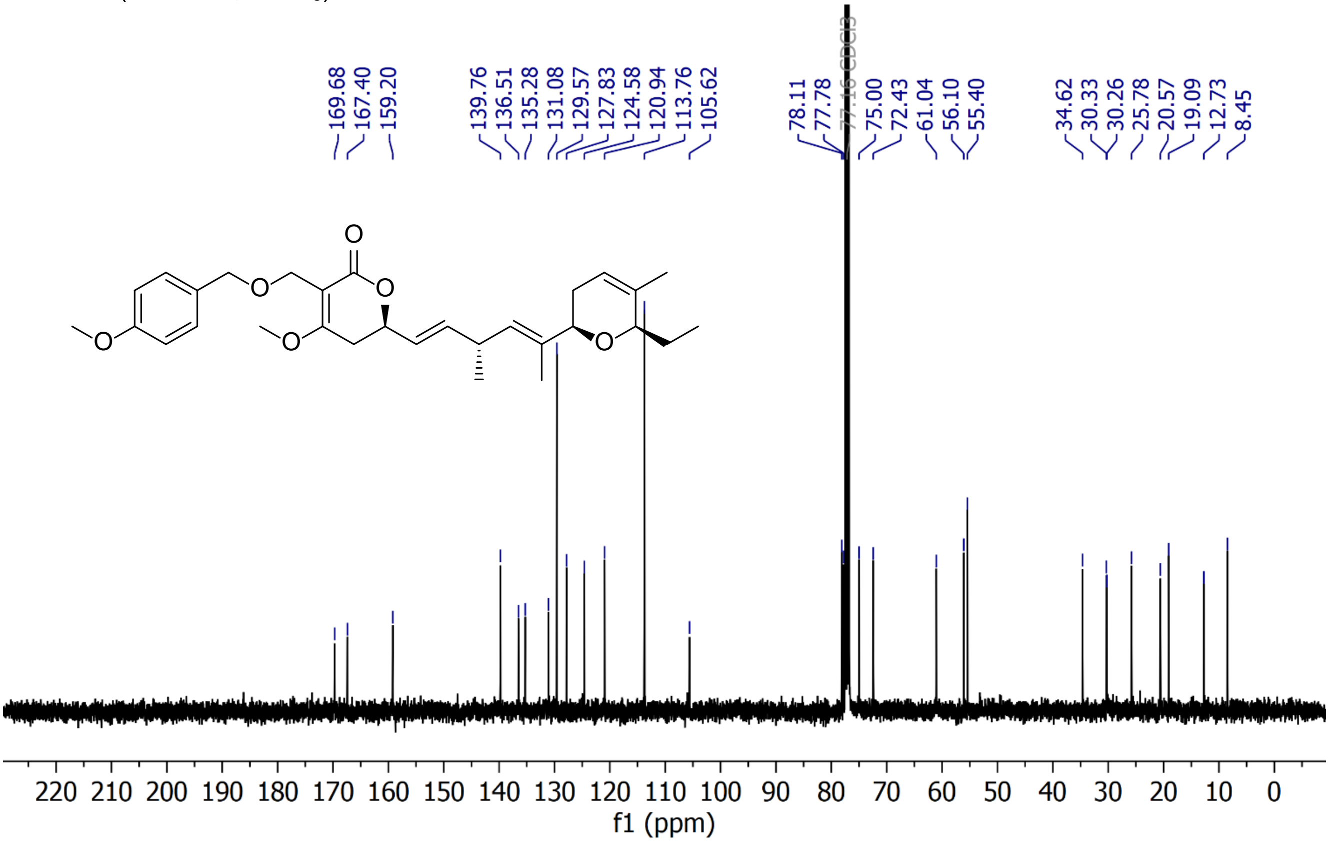

Jerangolid A 5

^{1}H-NMR (500 MHz, CD_3OD)

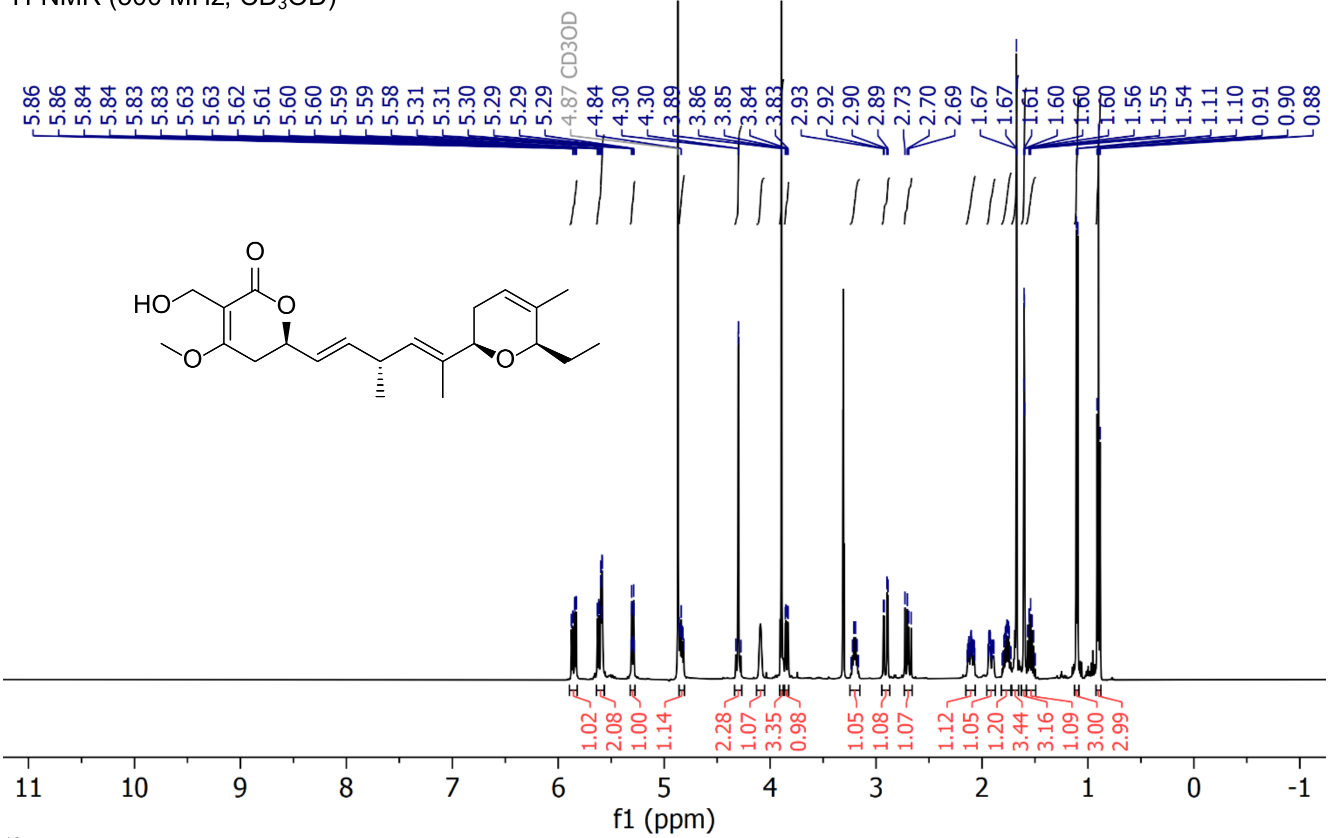

^{13}C-NMR (125 MHz, CD_3OD)

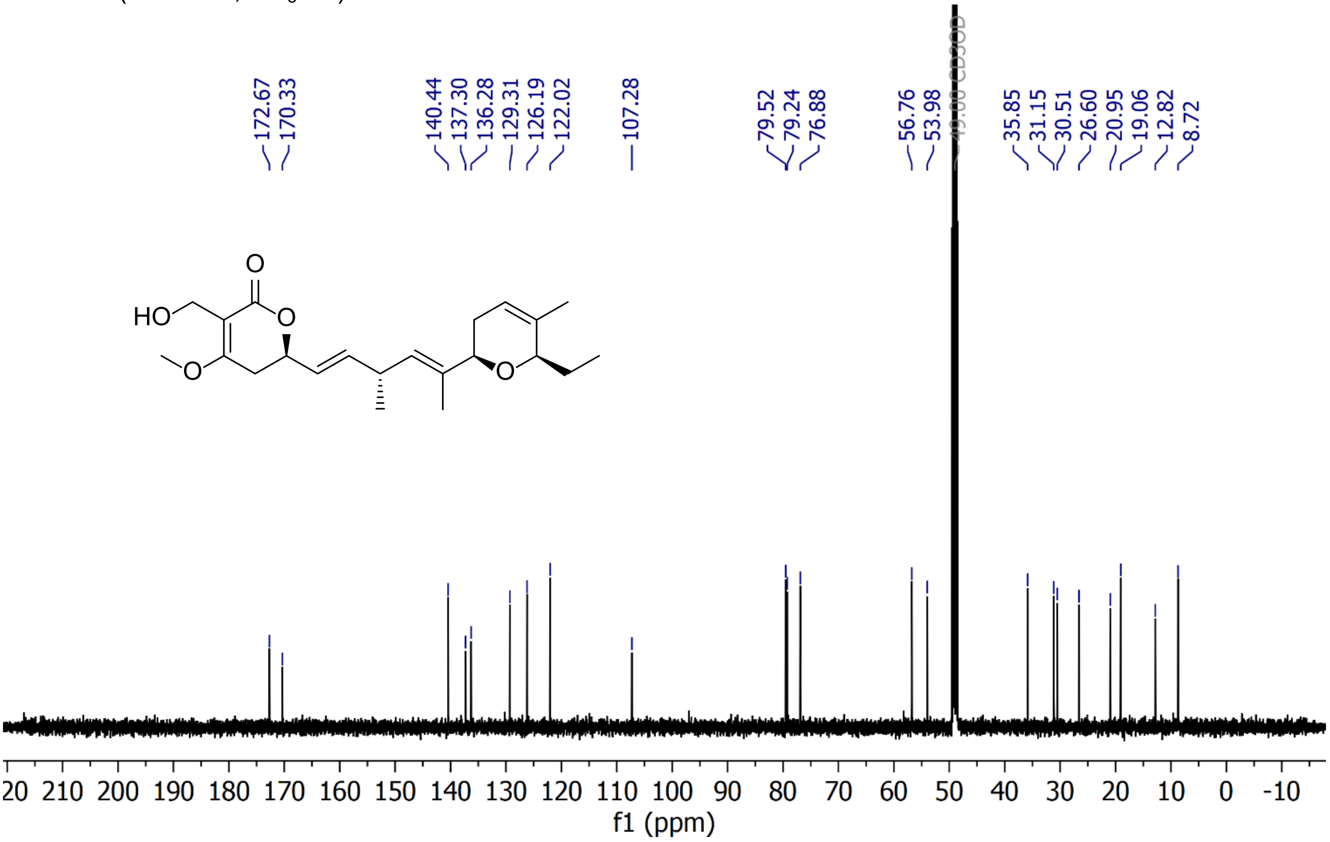

HH-gCOSY (CD_3OD)

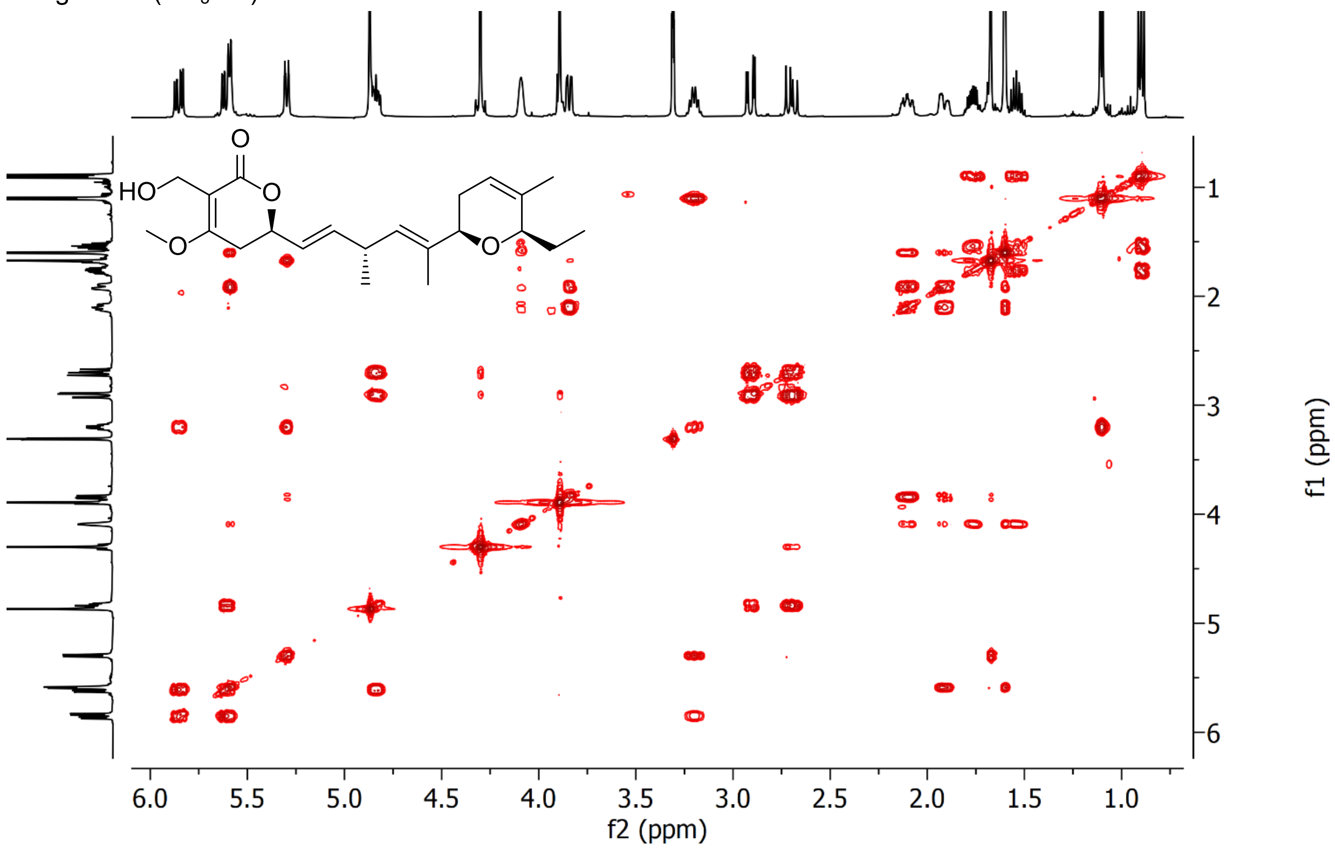

gHMBC (CD_3OD)

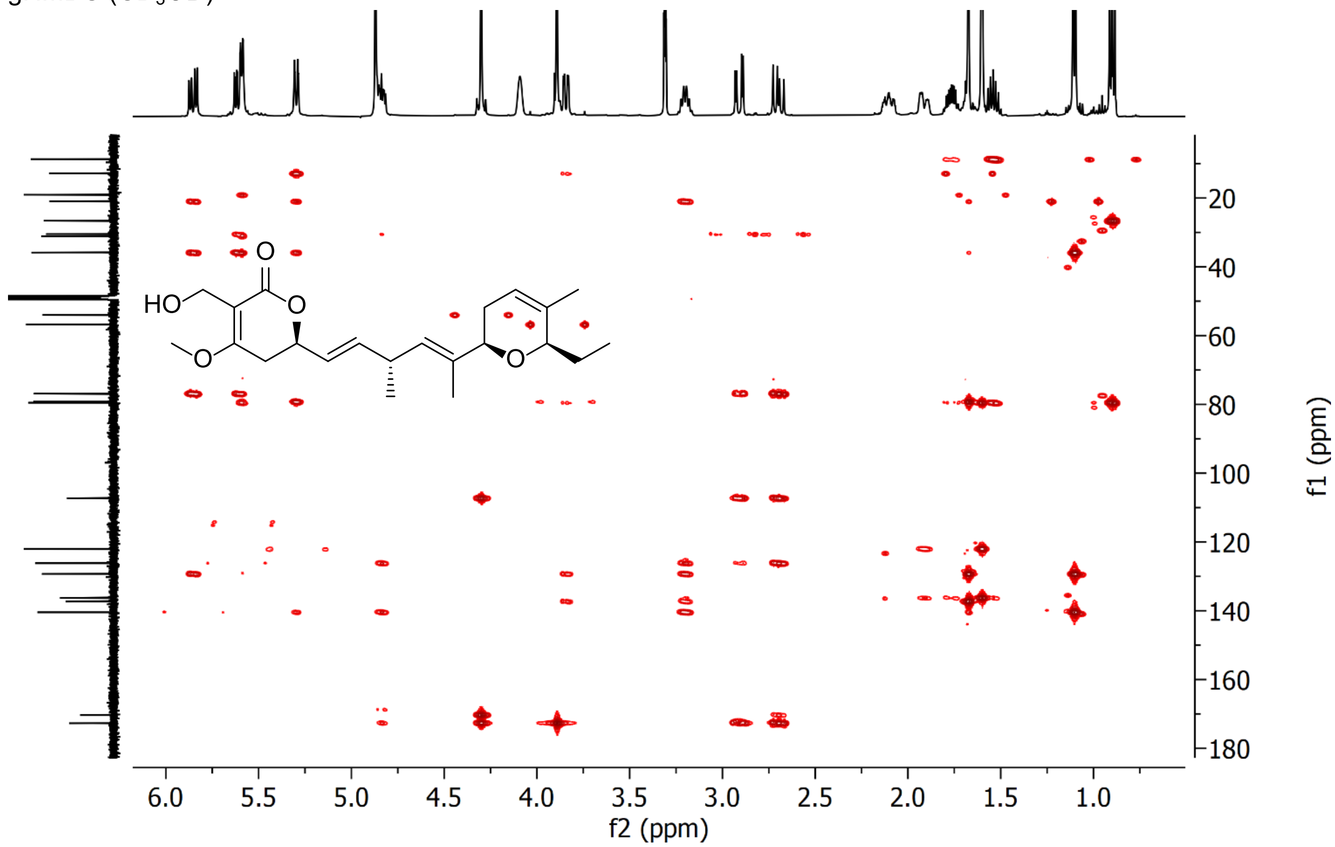

gHSQC-DEPT (CD_3OD)

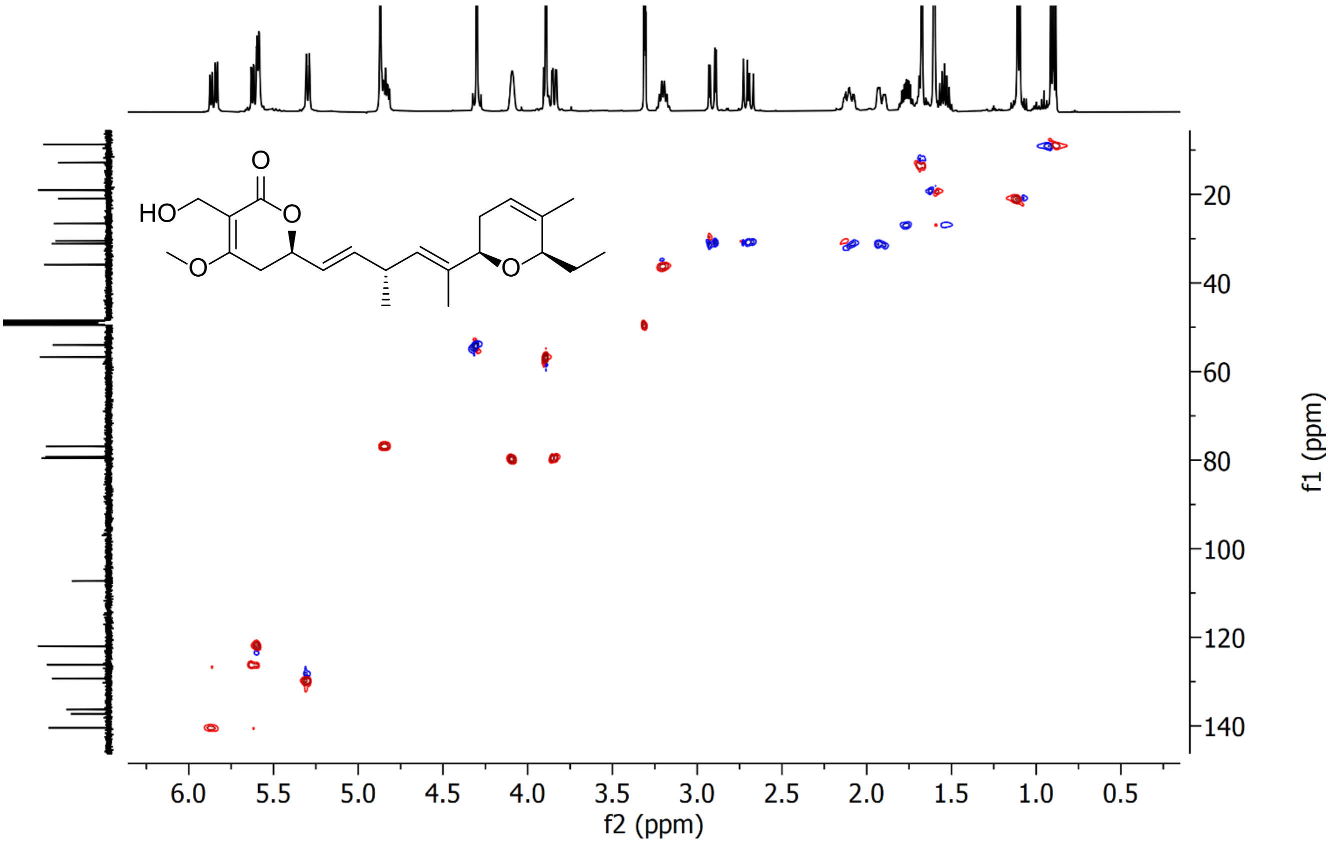

X-Ray crystallographic data

All compounds are crystallized after flash chromatography by dissolving a small amount of the purified material (<50 mg) in dry DCM (1-2 mL) in a 10 mL pear shaped flask. The flasks were sealed with a rubber septum and equipped with a cannula to allow slow solvent evaporation at room temperature over 2-3 days.

The data set for **20** was collected using a Bruker D8 Venture diffractometer with a microfocus sealed tube and a Photon II detector. Monochromated $Mo_{K\alpha}$ radiation (λ = 0.71073 Å) was used. Data were collected at 143(2) K and corrected for absorption effects using the multi-scan method.

The data sets for **28** and **49** were collected using a Rigaku XtaLAB Synergy S diffractometer with a microfocus sealed tube and a HyPix-6000HE Hybrid Photon Counting (HPC) detector. Monochromated $Mo_{K\alpha}$ radiation (λ = 0.71073 Å) was used. Data were collected at 130(2) K and corrected for absorption effects using the multi-scan method.

The structures were solved by direct methods using SHELXT[11] and was refined by full matrix least squares calculations on F^2 (SHELXL2018[12]) in the graphical user interface Shelxle.[13]

All non H-atoms were located in the electron density maps and refined anisotropically. C-bound H atoms were placed in positions of optimized geometry and treated as riding atoms. Their isotropic displacement parameters were coupled to the corresponding carrier atoms by a factor of 1.2 (CH, CH2) or 1.5 (CH3).

For the refinement of **20** the O bonded H-atoms were located in the electron density maps. Their positional parameters were refined using isotropic displacement parameters which were set at 1.5 times the Ueq value of their parent atoms. In addition, restraints of 0.84(0.01) Å were used for the O-H bond lengths.

For the refinement of **49** the O bonded H atoms H(2O) and H(4O) were located in the electron density maps. Their positional parameters were refined using isotropic displacement parameters which were set at 1.2 times the Ueq value of the parent atom. Restraints of 0.84 (0.01) Å were used for the O-H bond lengths.

Twinning: The data for **20** was checked for twinning in PLATON.[14] A twin matrix was found to be (1 0 1.247 0 -1 0 0 0 -1) from TwinRotMat. The final refinement was operated using the hklf5 routine in SHELX. The BASF value refined to 0.108(3).

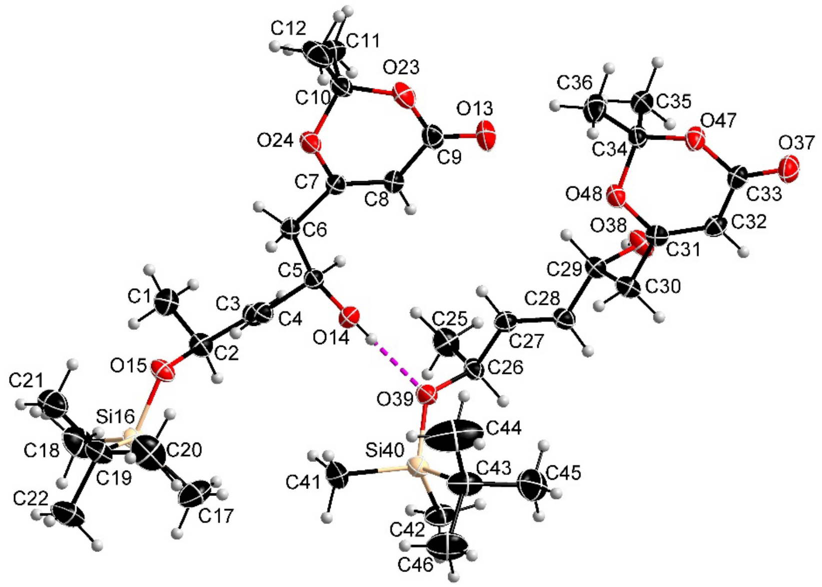

Molecular structure of lactone **20**. Displacement ellipsoids are displayed at the 50% probability level with hydrogen atoms being shown as spheres of arbitrary size.

Table S13. Crystal data and structure refinement for lactone **20**

Identification code	2520695	
Empirical formula	$C_{18}H_{32}O_5Si$	
Formula weight	356.52	
Temperature	143(2) K	
Wavelength	0.71073 Å	
Crystal system	Monoclinic	
Space group	C2	
Unit cell dimensions	a = 27.1154(11) Å	α = 90°
	b = 11.2486(4) Å	β = 109.106(2)°
	c = 14.2314(4) Å	γ = 90°
Volume	4101.6(3) Å^3	
Z	8	
Density (calculated)	1.155 Mg/m^3	
Absorption coefficient	0.136 mm^{-1}	

F(000)	1552
Crystal size	0.400 x 0.200 x 0.080 mm^3
Theta range for data collection	1.977 to 27.141°.
Index ranges	-34<=h<=34, -14<=k<=14, -16<=l<=18
Reflections collected	9068
Independent reflections	9068 [R(int) = ?]
Completeness to theta = 25.242°	99.7 %
Absorption correction	Semi-empirical from equivalents
Max. and min. transmission	0.7455 and 0.7166
Refinement method	Full-matrix least-squares on F^2
Data / restraints / parameters	9068 / 3 / 456
Goodness-of-fit on F^2	1.040
Final R indices [I>2sigma(I)]	R1 = 0.0398, wR2 = 0.1020
R indices (all data)	R1 = 0.0419, wR2 = 0.1037
Absolute structure parameter	0.03(3)
Extinction coefficient	n/a
Largest diff. peak and hole	0.976 and -0.302 e.$Å^{-3}$

Table S14. Atomic coordinates (x10^4) and equivalent isotropic displacement parameters (Å^{2}x10^3) for lactone **20**. U(eq) is defined as one third of the trace of the orthogonalized Uij tensor.

atom	X	Y	Z	U(eq)
C(1)	7839(1)	7070(3)	40(2)	33(1)
C(2)	8138(1)	6320(2)	931(2)	27(1)
C(3)	7827(1)	6085(3)	1613(2)	26(1)
C(4)	7710(1)	5031(2)	1880(2)	25(1)
C(5)	7372(1)	4839(2)	2522(2)	23(1)
C(6)	6879(1)	4165(3)	1951(2)	25(1)
C(7)	6463(1)	4134(3)	2422(2)	24(1)
C(8)	6458(1)	4653(3)	3262(2)	28(1)
C(9)	6026(1)	4460(3)	3646(2)	31(1)
C(10)	5560(1)	3776(3)	1998(2)	31(1)
C(11)	5364(1)	4949(3)	1501(2)	42(1)
C(12)	5202(1)	2752(4)	1572(3)	49(1)
O(13)	6018(1)	4760(3)	4446(2)	46(1)
O(14)	7632(1)	4142(2)	3388(1)	26(1)
O(15)	8287(1)	5219(2)	604(2)	27(1)
Si(16)	8876(1)	4939(1)	568(1)	25(1)
C(17)	9369(2)	5298(4)	1793(3)	52(1)
C(18)	9018(2)	5858(4)	-399(3)	50(1)
C(19)	8873(1)	3308(3)	303(2)	33(1)
C(20)	8792(2)	2615(3)	1170(3)	53(1)
C(21)	8438(1)	2999(4)	-661(3)	47(1)
C(22)	9402(2)	2943(4)	202(4)	56(1)
O(23)	5616(1)	3825(2)	3034(2)	34(1)
O(24)	6050(1)	3480(2)	1871(2)	30(1)
C(25)	8094(1)	7375(3)	4654(3)	36(1)
C(26)	8227(1)	6336(3)	5375(2)	25(1)
C(27)	7761(1)	5974(3)	5653(2)	26(1)

atom	X	Y	Z	U(eq)
C(28)	7717(1)	6088(2)	6544(2)	26(1)
C(29)	7228(1)	5847(2)	6786(2)	25(1)
C(30)	7299(1)	4828(3)	7533(2)	28(1)
C(31)	6804(1)	4553(2)	7732(2)	24(1)
C(32)	6711(1)	4725(2)	8587(2)	26(1)
C(33)	6231(1)	4312(2)	8713(2)	26(1)
C(34)	5908(1)	4173(3)	6920(2)	27(1)
C(35)	5715(1)	5428(3)	6641(2)	36(1)
C(36)	5598(1)	3247(3)	6199(2)	41(1)
O(37)	6132(1)	4277(2)	9476(2)	38(1)
O(38)	7085(1)	6865(2)	7235(2)	33(1)
O(39)	8389(1)	5352(2)	4892(1)	26(1)
Si(40)	8974(1)	4698(1)	5248(1)	27(1)
C(41)	9022(2)	3965(5)	4105(3)	56(1)
C(42)	9495(1)	5836(3)	5725(3)	35(1)
C(43)	9005(1)	3556(3)	6229(3)	43(1)
C(44)	8517(2)	2776(5)	5884(5)	90(2)
C(45)	9039(2)	4179(5)	7217(3)	62(1)
C(46)	9498(2)	2781(4)	6399(4)	57(1)
O(47)	5871(1)	3854(2)	7876(1)	30(1)
O(48)	6436(1)	4072(2)	6930(1)	27(1)

Table S15. Bond lengths [Å] for lactone **20**.

atoms	bond length [Å]	atoms	bond length [Å]
C(1)-C(2)	1.518(4)	C(25)-C(26)	1.518(4)
C(2)-O(15)	1.427(3)	C(26)-O(39)	1.444(3)
C(2)-C(3)	1.502(4)	C(26)-C(27)	1.500(4)
C(3)-C(4)	1.315(4)	C(27)-C(28)	1.318(4)
C(4)-C(5)	1.508(3)	C(28)-C(29)	1.502(4)
C(5)-O(14)	1.436(3)	C(29)-O(38)	1.426(3)
C(5)-C(6)	1.519(4)	C(29)-C(30)	1.532(4)
C(6)-C(7)	1.490(4)	C(30)-C(31)	1.491(3)
C(7)-C(8)	1.334(4)	C(31)-C(32)	1.336(4)
C(7)-O(24)	1.354(3)	C(31)-O(48)	1.359(3)
C(8)-C(9)	1.461(4)	C(32)-C(33)	1.446(4)
C(9)-O(13)	1.195(4)	C(33)-O(37)	1.201(3)
C(9)-O(23)	1.367(4)	C(33)-O(47)	1.369(3)
C(10)-O(23)	1.433(4)	C(34)-O(48)	1.435(3)
C(10)-O(24)	1.439(3)	C(34)-O(47)	1.441(3)
C(10)-C(12)	1.501(4)	C(34)-C(36)	1.510(4)
C(10)-C(11)	1.509(5)	C(34)-C(35)	1.512(5)
O(15)-Si(16)	1.646(2)	O(39)-Si(40)	1.669(2)
Si(16)-C(18)	1.859(3)	Si(40)-C(42)	1.862(3)
Si(16)-C(17)	1.860(4)	Si(40)-C(41)	1.866(4)
Si(16)-C(19)	1.873(3)	Si(40)-C(43)	1.879(3)
C(19)-C(21)	1.528(5)	C(43)-C(44)	1.528(5)
C(19)-C(20)	1.536(5)	C(43)-C(45)	1.546(6)
C(19)-C(22)	1.541(4)	C(43)-C(46)	1.548(5)

Table S16. Bond angles [°] for lactone **20**.

atoms	bond angle [°]	atoms	bond angle [°]
O(15)-C(2)-C(3)	109.7(2)	O(39)-C(26)-C(27)	109.3(2)
O(15)-C(2)-C(1)	109.9(2)	O(39)-C(26)-C(25)	108.1(2)
C(3)-C(2)-C(1)	112.2(2)	C(27)-C(26)-C(25)	110.7(2)
C(4)-C(3)-C(2)	125.7(3)	C(28)-C(27)-C(26)	125.2(3)
C(3)-C(4)-C(5)	123.8(2)	C(27)-C(28)-C(29)	124.6(3)
O(14)-C(5)-C(4)	111.9(2)	O(38)-C(29)-C(28)	110.3(2)
O(14)-C(5)-C(6)	106.6(2)	O(38)-C(29)-C(30)	106.3(2)
C(4)-C(5)-C(6)	110.5(2)	C(28)-C(29)-C(30)	112.0(2)
C(7)-C(6)-C(5)	115.7(2)	C(31)-C(30)-C(29)	111.8(2)
C(8)-C(7)-O(24)	120.5(2)	C(32)-C(31)-O(48)	121.2(2)
C(8)-C(7)-C(6)	128.6(2)	C(32)-C(31)-C(30)	126.4(2)
O(24)-C(7)-C(6)	110.9(2)	O(48)-C(31)-C(30)	112.4(2)
C(7)-C(8)-C(9)	121.0(3)	C(31)-C(32)-C(33)	120.8(2)
O(13)-C(9)-O(23)	119.2(3)	O(37)-C(33)-O(47)	117.9(3)
O(13)-C(9)-C(8)	125.6(3)	O(37)-C(33)-C(32)	126.7(3)
O(23)-C(9)-C(8)	115.1(2)	O(47)-C(33)-C(32)	115.3(2)
O(23)-C(10)-O(24)	110.3(2)	O(48)-C(34)-O(47)	110.4(2)
O(23)-C(10)-C(12)	106.4(3)	O(48)-C(34)-C(36)	106.4(2)
O(24)-C(10)-C(12)	105.9(2)	O(47)-C(34)-C(36)	105.9(2)
O(23)-C(10)-C(11)	110.6(3)	O(48)-C(34)-C(35)	109.6(2)
O(24)-C(10)-C(11)	109.9(3)	O(47)-C(34)-C(35)	110.9(2)
C(12)-C(10)-C(11)	113.6(3)	C(36)-C(34)-C(35)	113.5(3)
C(2)-O(15)-Si(16)	123.91(17)	C(26)-O(39)-Si(40)	126.86(17)
O(15)-Si(16)-C(18)	110.76(15)	O(39)-Si(40)-C(42)	109.85(13)
O(15)-Si(16)-C(17)	109.64(16)	O(39)-Si(40)-C(41)	105.15(14)
C(18)-Si(16)-C(17)	108.3(2)	C(42)-Si(40)-C(41)	111.10(19)
O(15)-Si(16)-C(19)	104.71(12)	O(39)-Si(40)-C(43)	109.52(13)

atoms	bond angle [°]	atoms	bond angle [°]
C(18)-Si(16)-C(19)	112.57(17)	C(42)-Si(40)-C(43)	110.78(15)
C(17)-Si(16)-C(19)	110.87(18)	C(41)-Si(40)-C(43)	110.3(2)
C(21)-C(19)-C(20)	109.5(3)	C(44)-C(43)-C(45)	109.9(4)
C(21)-C(19)-C(22)	108.9(3)	C(44)-C(43)-C(46)	109.9(4)
C(20)-C(19)-C(22)	108.8(3)	C(45)-C(43)-C(46)	108.8(3)
C(21)-C(19)-Si(16)	110.8(2)	C(44)-C(43)-Si(40)	109.2(3)
C(20)-C(19)-Si(16)	109.1(2)	C(45)-C(43)-Si(40)	109.9(3)
C(22)-C(19)-Si(16)	109.7(3)	C(46)-C(43)-Si(40)	109.1(3)
C(9)-O(23)-C(10)	117.7(2)	C(33)-O(47)-C(34)	118.6(2)
C(7)-O(24)-C(10)	115.1(2)	C(31)-O(48)-C(34)	115.6(2)

Table S17. Anisotropic displacement parameters (Å^{2}x 10^3) for lactone **20**. The anisotropic displacement factor exponent takes the form: $-2p^2[h^2 a^{*2}U^{11} + ... + 2hka^*b^*U^{12}]$

Atom	U^{11}	U^{22}	U^{33}	U^{23}	U^{13}	U^{12}
C(1)	38(2)	30(1)	33(2)	3(1)	13(1)	-3(1)
C(2)	28(1)	22(1)	33(1)	-3(1)	12(1)	-6(1)
C(3)	26(1)	26(1)	28(1)	-3(1)	10(1)	0(1)
C(4)	22(1)	25(1)	27(1)	-1(1)	10(1)	-1(1)
C(5)	24(1)	24(1)	23(1)	0(1)	9(1)	2(1)
C(6)	25(1)	28(1)	21(1)	-4(1)	9(1)	-1(1)
C(7)	23(1)	26(1)	21(1)	0(1)	6(1)	0(1)
C(8)	27(1)	34(2)	22(1)	-1(1)	8(1)	1(1)
C(9)	35(1)	33(2)	27(1)	5(1)	14(1)	7(1)
C(10)	25(1)	38(2)	34(2)	-6(1)	14(1)	-2(1)
C(11)	33(2)	47(2)	39(2)	3(2)	4(1)	7(1)
C(12)	31(2)	55(2)	66(2)	-27(2)	24(2)	-14(2)
O(13)	53(1)	64(2)	28(1)	-1(1)	23(1)	6(1)
O(14)	26(1)	26(1)	22(1)	0(1)	4(1)	0(1)
O(15)	24(1)	24(1)	38(1)	-2(1)	16(1)	-5(1)
Si(16)	21(1)	30(1)	28(1)	6(1)	11(1)	-4(1)
C(17)	37(2)	60(2)	47(2)	-1(2)	-1(2)	-9(2)
C(18)	53(2)	49(2)	61(2)	21(2)	36(2)	0(2)
C(19)	28(1)	34(2)	43(2)	3(1)	18(1)	4(1)
C(20)	69(3)	30(2)	71(3)	16(2)	37(2)	5(2)
C(21)	39(2)	45(2)	60(2)	-18(2)	18(2)	-5(2)
C(22)	39(2)	62(3)	70(3)	-1(2)	23(2)	16(2)
O(23)	34(1)	38(1)	37(1)	0(1)	22(1)	-2(1)
O(24)	24(1)	36(1)	32(1)	-11(1)	14(1)	-6(1)
C(25)	44(2)	27(2)	42(2)	3(1)	21(1)	-2(1)
C(26)	24(1)	28(1)	26(1)	-5(1)	11(1)	-4(1)
C(27)	23(1)	26(1)	27(1)	-1(1)	8(1)	-3(1)

Atom	U^{11}	U^{22}	U^{33}	U^{23}	U^{13}	U^{12}
C(28)	24(1)	27(1)	30(1)	-1(1)	10(1)	-2(1)
C(29)	24(1)	26(1)	27(1)	-1(1)	11(1)	0(1)
C(30)	25(1)	25(1)	35(1)	4(1)	14(1)	3(1)
C(31)	25(1)	18(1)	28(1)	0(1)	8(1)	1(1)
C(32)	25(1)	25(1)	25(1)	2(1)	5(1)	-3(1)
C(33)	29(1)	26(1)	23(1)	1(1)	7(1)	-1(1)
C(34)	26(1)	34(1)	22(1)	-4(1)	11(1)	-5(1)
C(35)	34(2)	46(2)	28(1)	2(1)	9(1)	9(1)
C(36)	39(2)	53(2)	32(2)	-14(1)	13(1)	-19(2)
O(37)	40(1)	51(1)	23(1)	1(1)	13(1)	-6(1)
O(38)	39(1)	24(1)	43(1)	3(1)	25(1)	4(1)
O(39)	24(1)	29(1)	27(1)	-4(1)	9(1)	0(1)
Si(40)	24(1)	32(1)	26(1)	-1(1)	10(1)	-2(1)
C(41)	40(2)	89(3)	40(2)	-24(2)	14(2)	11(2)
C(42)	25(1)	37(2)	42(2)	9(1)	9(1)	-6(1)
C(43)	31(2)	37(2)	59(2)	18(2)	12(2)	0(1)
C(44)	50(2)	52(3)	150(6)	44(3)	8(3)	-22(2)
C(45)	58(2)	93(3)	44(2)	29(2)	28(2)	16(2)
C(46)	47(2)	38(2)	81(3)	16(2)	13(2)	12(2)
O(47)	28(1)	39(1)	23(1)	-4(1)	11(1)	-11(1)
O(48)	28(1)	29(1)	27(1)	-6(1)	14(1)	-5(1)

Table S18. Hydrogen coordinates (x10^4) and isotropic displacement parameters (Å^{2}x10^3) for lactone **20**.

Atom	X	Y	Z	U(eq)
H(1A)	8060	7229	-368	50
H(1B)	7738	7824	271	50
H(1C)	7525	6642	-356	50
H(2)	8462	6759	1314	32
H(3)	7704	6758	1875	32
H(4)	7847	4351	1654	29
H(5)	7274	5627	2734	28
H(6A)	6732	4527	1284	30
H(6B)	6977	3336	1857	30
H(8)	6739	5155	3616	33
H(11A)	5363	4928	812	62
H(11B)	5593	5590	1860	62
H(11C)	5009	5091	1508	62
H(12A)	5144	2693	857	73
H(12B)	4867	2881	1681	73
H(12C)	5360	2014	1901	73
H(14)	7854(11)	4560(30)	3800(20)	38
H(17A)	9720	5180	1756	78
H(17B)	9318	4776	2304	78
H(17C)	9328	6129	1962	78
H(18A)	8763	5677	-1051	75
H(18B)	9370	5679	-407	75
H(18C)	8995	6702	-248	75
H(20A)	8464	2867	1260	80
H(20B)	9082	2775	1781	80
H(20C)	8776	1762	1024	80
H(21A)	8494	3429	-1216	71

Atom	X	Y	Z	U(eq)
H(21B)	8101	3228	-604	71
H(21C)	8441	2141	-782	71
H(22A)	9398	2090	60	83
H(22B)	9682	3116	824	83
H(22C)	9460	3390	-343	83
H(25A)	8407	7624	4506	54
H(25B)	7965	8040	4952	54
H(25C)	7824	7132	4037	54
H(26)	8519	6566	5987	30
H(27)	7474	5636	5147	31
H(28)	8017	6340	7071	32
H(29)	6938	5646	6161	30
H(30A)	7419	4109	7270	33
H(30B)	7571	5049	8165	33
H(32)	6962	5124	9120	31
H(35A)	5726	5611	5975	54
H(35B)	5939	5987	7123	54
H(35C)	5356	5500	6645	54
H(36A)	5606	3426	5531	61
H(36B)	5236	3252	6195	61
H(36C)	5751	2460	6405	61
H(38)	7155(16)	7460(30)	6940(30)	49
H(41A)	8912	4524	3547	84
H(41B)	8795	3264	3951	84
H(41C)	9384	3722	4217	84
H(42A)	9507	6349	5176	53
H(42B)	9832	5440	6015	53
H(42C)	9419	6320	6234	53

Atom	X	Y	Z	U(eq)
H(44A)	8206	3275	5756	135
H(44B)	8528	2193	6402	135
H(44C)	8506	2360	5272	135
H(45A)	9355	4668	7440	94
H(45B)	9051	3578	7723	94
H(45C)	8731	4685	7114	94
H(46A)	9479	2375	5779	86
H(46B)	9519	2190	6916	86
H(46C)	9809	3288	6610	86

Table S19. Torsion angles [°] for lactone **20**.

atoms	Torsion angle [°]	atoms	torsion angle [°]
O(15)-C(2)-C(3)-C(4)	0.4(4)	O(39)-C(26)-C(27)-C(28)	128.3(3)
C(1)-C(2)-C(3)-C(4)	122.9(3)	C(25)-C(26)-C(27)-C(28)	-112.8(3)
C(2)-C(3)-C(4)-C(5)	-176.5(3)	C(26)-C(27)-C(28)-C(29)	173.0(3)
C(3)-C(4)-C(5)-O(14)	-124.4(3)	C(27)-C(28)-C(29)-O(38)	-124.3(3)
C(3)-C(4)-C(5)-C(6)	117.0(3)	C(27)-C(28)-C(29)-C(30)	117.5(3)
O(14)-C(5)-C(6)-C(7)	70.3(3)	O(38)-C(29)-C(30)-C(31)	62.3(3)
C(4)-C(5)-C(6)-C(7)	-167.9(2)	C(28)-C(29)-C(30)-C(31)	-177.2(2)
C(5)-C(6)-C(7)-C(8)	2.5(4)	C(29)-C(30)-C(31)-C(32)	-112.5(3)
C(5)-C(6)-C(7)-O(24)	-178.0(2)	C(29)-C(30)-C(31)-O(48)	68.3(3)
O(24)-C(7)-C(8)-C(9)	4.6(4)	O(48)-C(31)-C(32)-C(33)	5.3(4)
C(6)-C(7)-C(8)-C(9)	-176.0(3)	C(30)-C(31)-C(32)-C(33)	-173.9(3)
C(7)-C(8)-C(9)-O(13)	170.0(3)	C(31)-C(32)-C(33)-O(37)	169.8(3)
C(7)-C(8)-C(9)-O(23)	-6.5(4)	C(31)-C(32)-C(33)-O(47)	-6.2(4)
C(3)-C(2)-O(15)-Si(16)	-135.1(2)	C(27)-C(26)-O(39)-Si(40)	-122.1(2)
C(1)-C(2)-O(15)-Si(16)	101.0(2)	C(25)-C(26)-O(39)-Si(40)	117.4(2)
C(2)-O(15)-Si(16)-C(18)	-68.1(3)	C(26)-O(39)-Si(40)-C(42)	-38.2(3)
C(2)-O(15)-Si(16)-C(17)	51.3(3)	C(26)-O(39)-Si(40)-C(41)	-157.8(3)
C(2)-O(15)-Si(16)-C(19)	170.3(2)	C(26)-O(39)-Si(40)-C(43)	83.7(2)
O(15)-Si(16)-C(19)-C(21)	57.1(2)	O(39)-Si(40)-C(43)-C(44)	48.4(4)
C(18)-Si(16)-C(19)-C(21)	-63.3(3)	C(42)-Si(40)-C(43)-C(44)	169.7(4)
C(17)-Si(16)-C(19)-C(21)	175.3(2)	C(41)-Si(40)-C(43)-C(44)	-66.8(4)
O(15)-Si(16)-C(19)-C(20)	-63.5(3)	O(39)-Si(40)-C(43)-C(45)	-72.2(3)
C(18)-Si(16)-C(19)-C(20)	176.1(3)	C(42)-Si(40)-C(43)-C(45)	49.1(3)
C(17)-Si(16)-C(19)-C(20)	54.6(3)	C(41)-Si(40)-C(43)-C(45)	172.5(3)
O(15)-Si(16)-C(19)-C(22)	177.4(2)	O(39)-Si(40)-C(43)-C(46)	168.5(3)
C(18)-Si(16)-C(19)-C(22)	57.0(3)	C(42)-Si(40)-C(43)-C(46)	-70.1(3)
C(17)-Si(16)-C(19)-C(22)	-64.5(3)	C(41)-Si(40)-C(43)-C(46)	53.3(3)

atoms	Torsion angle [°]	atoms	torsion angle [°]
O(13)-C(9)-O(23)-C(10)	162.1(3)	O(37)-C(33)-O(47)-C(34)	162.9(3)
C(8)-C(9)-O(23)-C(10)	-21.1(4)	C(32)-C(33)-O(47)-C(34)	-20.8(4)
O(24)-C(10)-O(23)-C(9)	48.2(3)	O(48)-C(34)-O(47)-C(33)	46.2(3)
C(12)-C(10)-O(23)-C(9)	162.6(3)	C(36)-C(34)-O(47)-C(33)	161.1(3)
C(11)-C(10)-O(23)-C(9)	-73.6(3)	C(35)-C(34)-O(47)-C(33)	-75.4(3)
C(8)-C(7)-O(24)-C(10)	24.4(4)	C(32)-C(31)-O(48)-C(34)	22.2(4)
C(6)-C(7)-O(24)-C(10)	-155.2(2)	C(30)-C(31)-O(48)-C(34)	-158.5(2)
O(23)-C(10)-O(24)-C(7)	-49.3(3)	O(47)-C(34)-O(48)-C(31)	-46.1(3)
C(12)-C(10)-O(24)-C(7)	-164.0(3)	C(36)-C(34)-O(48)-C(31)	-160.6(2)
C(11)-C(10)-O(24)-C(7)	72.9(3)	C(35)-C(34)-O(48)-C(31)	76.3(3)

Table S20. Hydrogen bonds and angles for lactone **20** [Å and °].

D-H...A	d(D-H) [Å]	d(H...A) [Å]	d(D...A) [Å]	<(DHA) [°]
O(14)-H(14)...O(39)	0.839(14)	1.955(15)	2.788(3)	172(4)
O(38)-H(38)...O(14)#1	0.843(14)	2.077(19)	2.895(3)	164(4)
Symmetry transformation used to generate equivalent atoms: #1 -x+3/2,y+1/2,-z+1				

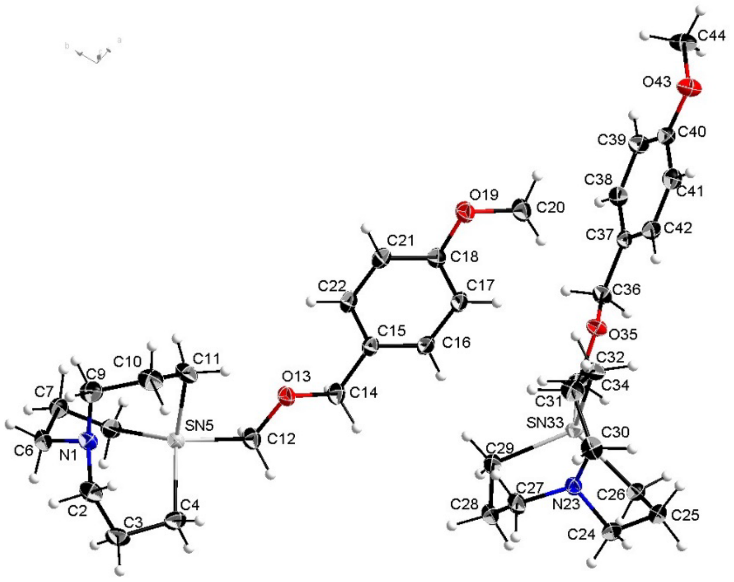

Molecular structure of stannatrane **28**. Displacement ellipsoids are displayed at the 50% probability level with hydrogen atoms being shown as spheres of arbitrary size.

Table S21. Crystal data and structure refinement for stannatrane **28**

Identification code	2520696	
Empirical formula	$C_{18}H_{29}NO_2Sn$	
Formula weight	410.11	
Temperature	130(2) K	
Wavelength	0.71073 Å	
Crystal system	Triclinic	
Space group	P-1	
Unit cell dimensions	a = 11.7229(2) Å	α = 104.293(2)°.
	b = 12.2845(3) Å	β = 96.430(2)°.
	c = 13.1866(3) Å	γ = 91.345(2)°.
Volume	1826.06(7) $Å^3$	
Z	4	
Density (calculated)	1.492 Mg/m^3	
Absorption coefficient	1.406 mm^{-1}	
F(000)	840	
Crystal size	0.220 x 0.120 x 0.040 mm^3	
Theta range for data collection	2.227 to 31.613°.	
Index ranges	-15<=h<=15, -16<=k<=16, -18<=l<=17	
Reflections collected	29172	
Independent reflections	9871 [R(int) = 0.0170]	

Completeness to theta = 25.242°	99.9 %
Absorption correction	Semi-empirical from equivalents
Max. and min. transmission	1.00000 and 0.80490
Refinement method	Full-matrix least-squares on F^2
Data / restraints / parameters	9871 / 0 / 399
Goodness-of-fit on F^2	1.030
Final R indices [I>2sigma(I)]	R1 = 0.0172, wR^2 = 0.0406
R indices (all data)	R1 = 0.0208, wR^2 = 0.0418
Extinction coefficient	n/a
Largest diff. peak and hole	0.476 and -0.373 e.Å^{-3}

Table S22. Atomic coordinates (x10^4) and equivalent isotropic displacement parameters (Å^{2}x10^3) for stannatrane **28**. U(eq) is defined as one third of the trace of the orthogonalized Uij tensor.

atom	X	Y	Z	U(eq)
Sn(5)	1804(1)	6113(1)	1732(1)	19(1)
Sn(33)	6821(1)	-98(1)	1799(1)	18(1)
O(13)	4037(1)	4984(1)	1731(1)	26(1)
O(19)	8872(1)	4436(1)	4187(1)	31(1)
O(35)	9148(1)	1000(1)	1830(1)	23(1)
O(43)	13996(1)	2607(1)	4454(1)	33(1)
N(1)	316(1)	7382(1)	2669(1)	27(1)
N(23)	5304(1)	-1005(1)	2665(1)	22(1)
C(2)	-668(1)	6612(1)	2654(1)	34(1)
C(3)	-815(1)	5677(1)	1637(1)	33(1)
C(4)	267(1)	5015(1)	1504(1)	29(1)
C(6)	58(1)	8190(1)	2029(1)	34(1)
C(7)	1117(1)	8469(1)	1549(1)	32(1)
C(8)	1613(1)	7423(1)	897(1)	27(1)
C(9)	896(1)	7890(1)	3733(1)	35(1)
C(10)	1703(1)	7069(2)	4098(1)	33(1)
C(11)	2597(1)	6692(1)	3346(1)	29(1)
C(12)	3089(1)	5034(1)	957(1)	26(1)
C(14)	4884(1)	4283(1)	1288(1)	26(1)
C(15)	5916(1)	4310(1)	2082(1)	22(1)
C(16)	6688(1)	3465(1)	1883(1)	24(1)
C(17)	7681(1)	3460(1)	2566(1)	25(1)
C(18)	7906(1)	4329(1)	3474(1)	24(1)
C(20)	9698(1)	3597(1)	3948(1)	31(1)
C(21)	7133(1)	5177(1)	3698(1)	26(1)
C(22)	6149(1)	5167(1)	3007(1)	25(1)
C(24)	5120(1)	-2177(1)	2029(1)	28(1)
C(25)	6221(1)	-2603(1)	1595(1)	28(1)
C(26)	6687(1)	-1854(1)	949(1)	25(1)
C(27)	4289(1)	-337(1)	2597(1)	28(1)
C(28)	4181(1)	62(1)	1585(1)	29(1)
C(29)	5260(1)	759(1)	1516(1)	26(1)
C(30)	5827(1)	-898(1)	3754(1)	29(1)
C(31)	6587(1)	183(1)	4148(1)	30(1)
C(32)	7531(1)	223(1)	3442(1)	29(1)

atom	X	Y	Z	U(eq)
C(34)	8164(1)	609(1)	1059(1)	24(1)
C(36)	10060(1)	1436(1)	1403(1)	23(1)
C(37)	11100(1)	1762(1)	2217(1)	20(1)
C(38)	11958(1)	2495(1)	2084(1)	24(1)
C(39)	12940(1)	2814(1)	2809(1)	25(1)
C(40)	13065(1)	2379(1)	3691(1)	24(1)
C(41)	12205(1)	1653(1)	3843(1)	26(1)
C(42)	11236(1)	1348(1)	3112(1)	24(1)
C(44)	14889(1)	3354(2)	4328(1)	37(1)

Table S23. Bond lengths [Å] for stannatrane **28**.

atoms	bond length [Å]	atome	bond length [Å]
Sn(5)-C(11)	2.1629(14)	C(3)-C(4)	1.526(2)
Sn(5)-C(8)	2.1659(14)	C(6)-C(7)	1.524(2)
Sn(5)-C(4)	2.1723(14)	C(7)-C(8)	1.526(2)
Sn(5)-C(12)	2.2005(14)	C(9)-C(10)	1.526(2)
Sn(5)-N(1)	2.5806(12)	C(10)-C(11)	1.525(2)
Sn(33)-C(26)	2.1653(14)	C(14)-C(15)	1.5032(19)
Sn(33)-C(32)	2.1690(14)	C(15)-C(16)	1.3882(19)
Sn(33)-C(29)	2.1741(14)	C(15)-C(22)	1.3958(19)
Sn(33)-C(34)	2.2093(13)	C(16)-C(17)	1.391(2)
Sn(33)-N(23)	2.5885(11)	C(17)-C(18)	1.388(2)
O(13)-C(14)	1.4112(17)	C(18)-C(21)	1.394(2)
O(13)-C(12)	1.4354(17)	C(21)-C(22)	1.385(2)
O(19)-C(18)	1.3701(17)	C(24)-C(25)	1.521(2)
O(19)-C(20)	1.4337(18)	C(25)-C(26)	1.530(2)
O(35)-C(36)	1.4151(15)	C(27)-C(28)	1.525(2)
O(35)-C(34)	1.4365(16)	C(28)-C(29)	1.534(2)
O(43)-C(40)	1.3726(17)	C(30)-C(31)	1.525(2)
O(43)-C(44)	1.4267(19)	C(31)-C(32)	1.532(2)
N(1)-C(9)	1.468(2)	C(36)-C(37)	1.5070(18)
N(1)-C(6)	1.469(2)	C(37)-C(38)	1.3880(19)
N(1)-C(2)	1.4699(19)	C(37)-C(42)	1.3916(19)
N(23)-C(27)	1.4678(17)	C(38)-C(39)	1.394(2)
N(23)-C(30)	1.4698(19)	C(39)-C(40)	1.390(2)
N(23)-C(24)	1.4739(18)	C(40)-C(41)	1.394(2)
C(2)-C(3)	1.526(2)	C(41)-C(42)	1.385(2)

Table S24. Bond angles [°] for stannatrane **28**.

atoms	bond angle [°]	atoms	bond angle [°]
C(11)-Sn(5)-C(8)	114.20(6)	N(1)-C(6)-C(7)	110.83(12)
C(11)-Sn(5)-C(4)	115.09(6)	C(6)-C(7)-C(8)	112.54(13)
C(8)-Sn(5)-C(4)	113.23(6)	C(7)-C(8)-Sn(5)	111.97(10)
C(11)-Sn(5)-C(12)	103.24(5)	N(1)-C(9)-C(10)	110.44(13)
C(8)-Sn(5)-C(12)	104.62(6)	C(11)-C(10)-C(9)	111.95(13)
C(4)-Sn(5)-C(12)	104.80(6)	C(10)-C(11)-Sn(5)	111.39(10)
C(11)-Sn(5)-N(1)	75.86(5)	O(13)-C(12)-Sn(5)	108.89(9)
C(8)-Sn(5)-N(1)	75.91(5)	O(13)-C(14)-C(15)	111.38(11)
C(4)-Sn(5)-N(1)	75.58(5)	C(16)-C(15)-C(22)	118.19(13)
C(12)-Sn(5)-N(1)	179.09(5)	C(16)-C(15)-C(14)	119.07(12)
C(26)-Sn(33)-C(32)	114.65(6)	C(22)-C(15)-C(14)	122.72(12)
C(26)-Sn(33)-C(29)	112.45(5)	C(15)-C(16)-C(17)	122.07(13)
C(32)-Sn(33)-C(29)	114.71(6)	C(18)-C(17)-C(16)	118.81(13)
C(26)-Sn(33)-C(34)	102.58(5)	O(19)-C(18)-C(17)	124.17(13)
C(32)-Sn(33)-C(34)	103.92(5)	O(19)-C(18)-C(21)	115.74(12)
C(29)-Sn(33)-C(34)	107.04(5)	C(17)-C(18)-C(21)	120.08(13)
C(26)-Sn(33)-N(23)	75.47(5)	C(22)-C(21)-C(18)	120.19(13)
C(32)-Sn(33)-N(23)	75.50(5)	C(21)-C(22)-C(15)	120.64(13)
C(29)-Sn(33)-N(23)	75.48(5)	N(23)-C(24)-C(25)	110.70(11)
C(34)-Sn(33)-N(23)	177.35(5)	C(24)-C(25)-C(26)	111.92(12)
C(14)-O(13)-C(12)	111.72(10)	C(25)-C(26)-Sn(33)	111.80(9)
C(18)-O(19)-C(20)	116.57(11)	N(23)-C(27)-C(28)	110.70(12)
C(36)-O(35)-C(34)	112.45(10)	C(27)-C(28)-C(29)	111.30(12)
C(40)-O(43)-C(44)	116.92(12)	C(28)-C(29)-Sn(33)	111.78(9)
C(9)-N(1)-C(6)	114.15(13)	N(23)-C(30)-C(31)	110.32(12)
C(9)-N(1)-C(2)	113.42(13)	C(30)-C(31)-C(32)	111.58(12)
C(6)-N(1)-C(2)	113.37(12)	C(31)-C(32)-Sn(33)	111.26(9)
C(9)-N(1)-Sn(5)	104.83(9)	O(35)-C(34)-Sn(33)	109.25(8)
C(6)-N(1)-Sn(5)	105.01(9)	O(35)-C(36)-C(37)	110.67(11)
C(2)-N(1)-Sn(5)	104.80(9)	C(38)-C(37)-C(42)	118.17(12)
C(27)-N(23)-C(30)	113.16(12)	C(38)-C(37)-C(36)	119.99(12)
C(27)-N(23)-C(24)	113.50(11)	C(42)-C(37)-C(36)	121.84(12)
C(30)-N(23)-C(24)	113.79(12)	C(37)-C(38)-C(39)	122.13(13)
C(27)-N(23)-Sn(33)	104.94(8)	C(40)-C(39)-C(38)	118.75(13)
C(30)-N(23)-Sn(33)	105.10(8)	O(43)-C(40)-C(39)	124.73(13)

atoms	bond angle [°]	atoms	bond angle [°]
C(24)-N(23)-Sn(33)	105.19(8)	O(43)-C(40)-C(41)	115.45(13)
N(1)-C(2)-C(3)	110.42(12)	C(39)-C(40)-C(41)	119.81(13)
C(2)-C(3)-C(4)	111.42(12)	C(42)-C(41)-C(40)	120.44(13)
C(3)-C(4)-Sn(5)	111.95(10)	C(41)-C(42)-C(37)	120.68(13)

Table S25. Anisotropic displacement parameters (Å^{2}x 10^3) for stannatrane **28**. The anisotropic displacement factor exponent takes the form: -2p^2[h^2 a^{*2}U^{11} + ... + 2hka*b*U^{12}]

Atom	U11	U22	U33	U23	U13	U12
Sn(5)	17(1)	21(1)	19(1)	6(1)	1(1)	1(1)
Sn(33)	16(1)	19(1)	19(1)	2(1)	3(1)	-1(1)
O(13)	22(1)	26(1)	26(1)	1(1)	3(1)	6(1)
O(19)	29(1)	31(1)	27(1)	1(1)	0(1)	9(1)
O(35)	17(1)	28(1)	24(1)	8(1)	3(1)	-2(1)
O(43)	27(1)	38(1)	30(1)	8(1)	-4(1)	-6(1)
N(1)	18(1)	27(1)	35(1)	5(1)	4(1)	1(1)
N(23)	17(1)	22(1)	30(1)	8(1)	4(1)	2(1)
C(2)	18(1)	40(1)	44(1)	11(1)	8(1)	0(1)
C(3)	21(1)	36(1)	42(1)	13(1)	0(1)	-10(1)
C(4)	29(1)	25(1)	33(1)	8(1)	2(1)	-6(1)
C(6)	22(1)	27(1)	54(1)	11(1)	1(1)	6(1)
C(7)	28(1)	24(1)	44(1)	14(1)	-2(1)	-1(1)
C(8)	28(1)	26(1)	30(1)	12(1)	-1(1)	-3(1)
C(9)	28(1)	36(1)	34(1)	-4(1)	9(1)	2(1)
C(10)	32(1)	43(1)	21(1)	1(1)	4(1)	0(1)
C(11)	22(1)	41(1)	22(1)	5(1)	0(1)	4(1)
C(12)	26(1)	29(1)	23(1)	6(1)	4(1)	7(1)
C(14)	26(1)	25(1)	25(1)	4(1)	6(1)	6(1)
C(15)	23(1)	19(1)	24(1)	6(1)	6(1)	2(1)
C(16)	25(1)	18(1)	27(1)	2(1)	5(1)	2(1)
C(17)	26(1)	20(1)	29(1)	4(1)	6(1)	6(1)
C(18)	24(1)	24(1)	26(1)	7(1)	4(1)	2(1)
C(20)	27(1)	34(1)	30(1)	6(1)	5(1)	9(1)
C(21)	31(1)	21(1)	25(1)	1(1)	6(1)	3(1)
C(22)	28(1)	20(1)	28(1)	6(1)	9(1)	6(1)
C(24)	23(1)	21(1)	39(1)	9(1)	2(1)	-3(1)
C(25)	28(1)	19(1)	34(1)	4(1)	1(1)	3(1)
C(26)	24(1)	22(1)	26(1)	1(1)	2(1)	2(1)
C(27)	16(1)	29(1)	42(1)	11(1)	9(1)	4(1)
C(28)	19(1)	28(1)	40(1)	10(1)	1(1)	6(1)

Atom	U^{11}	U^{22}	U^{33}	U^{23}	U^{13}	U^{12}
C(29)	25(1)	22(1)	32(1)	8(1)	4(1)	4(1)
C(30)	29(1)	35(1)	27(1)	12(1)	8(1)	3(1)
C(31)	30(1)	37(1)	20(1)	4(1)	6(1)	2(1)
C(32)	22(1)	37(1)	23(1)	1(1)	1(1)	-3(1)
C(34)	21(1)	26(1)	23(1)	3(1)	4(1)	-2(1)
C(36)	19(1)	26(1)	25(1)	9(1)	4(1)	0(1)
C(37)	18(1)	18(1)	25(1)	5(1)	5(1)	4(1)
C(38)	23(1)	25(1)	27(1)	9(1)	6(1)	2(1)
C(39)	22(1)	25(1)	30(1)	7(1)	6(1)	-2(1)
C(40)	22(1)	22(1)	26(1)	2(1)	2(1)	1(1)
C(41)	28(1)	25(1)	26(1)	11(1)	2(1)	1(1)
C(42)	23(1)	21(1)	30(1)	10(1)	5(1)	-1(1)
C(44)	29(1)	42(1)	34(1)	2(1)	-1(1)	-11(1)

Table S26. Hydrogen coordinates (x10^{4}) and isotropic displacement parameters (Å^{2}x10^{3}) for stannatrane **28**.

Atom	X	Y	Z	U(eq)
H(2A)	-1376	7036	2711	40
H(2B)	-545	6279	3267	40
H(3A)	-989	6010	1028	39
H(3B)	-1472	5160	1645	39
H(4A)	192	4502	789	35
H(4B)	344	4549	2021	35
H(6A)	-204	8888	2476	41
H(6B)	-570	7868	1459	41
H(7A)	1713	8866	2121	38
H(7B)	910	8983	1094	38
H(8A)	2373	7631	718	33
H(8B)	1100	7132	229	33
H(9A)	316	8097	4228	42
H(9B)	1341	8585	3731	42
H(10A)	2099	7433	4809	40
H(10B)	1246	6401	4151	40
H(11A)	3023	6078	3550	35
H(11B)	3154	7329	3403	35
H(12A)	2741	4268	629	31
H(12B)	3361	5345	397	31
H(14A)	5124	4532	681	31
H(14B)	4554	3501	1024	31
H(16)	6533	2872	1261	29
H(17)	8196	2872	2415	30
H(20A)	9341	2855	3903	46
H(20B)	10351	3759	4507	46
H(20C)	9969	3604	3273	46
H(21)	7282	5764	4325	32
H(22)	5628	5748	3165	30
H(24A)	4507	-2221	1438	33
H(24B)	4867	-2660	2471	33
H(25A)	6067	-3378	1145	33
H(25B)	6809	-2629	2188	33
H(26A)	6172	-1948	281	30
H(26B)	7455	-2094	769	30

Atom	X	Y	Z	U(eq)
H(27A)	4354	322	3214	34
H(27B)	3591	-798	2613	34
H(28A)	3502	524	1563	35
H(28B)	4061	-598	970	35
H(29A)	5289	1493	2042	31
H(29B)	5214	905	807	31
H(30A)	6294	-1553	3783	35
H(30B)	5215	-890	4216	35
H(31A)	6944	235	4876	36
H(31B)	6106	838	4165	36
H(32A)	8093	-348	3525	34
H(32B)	7943	972	3665	34
H(34A)	8386	25	461	29
H(34B)	7864	1241	785	29
H(36A)	9812	2103	1160	27
H(36B)	10262	863	787	27
H(38)	11872	2789	1479	29
H(39)	13514	3320	2703	30
H(41)	12285	1366	4452	31
H(42)	10657	851	3223	29
H(44A)	15208	3033	3669	56
H(44B)	15497	3461	4923	56
H(44C)	14576	4081	4304	56

Table S27. Torsion angles [°] for stannatrane **28**.

atoms	torsion angle [°]	atoms	torsion angle [°]
C(9)-N(1)-C(2)-C(3)	-149.02(13)	C(27)-N(23)-C(24)-C(25)	146.43(12)
C(6)-N(1)-C(2)-C(3)	78.68(17)	C(30)-N(23)-C(24)-C(25)	-82.23(15)
Sn(5)-N(1)-C(2)-C(3)	-35.27(14)	Sn(33)-N(23)-C(24)-C(25)	32.27(13)
N(1)-C(2)-C(3)-C(4)	58.12(17)	N(23)-C(24)-C(25)-C(26)	-56.37(16)
C(2)-C(3)-C(4)-Sn(5)	-49.70(15)	C(24)-C(25)-C(26)-Sn(33)	50.85(14)
C(9)-N(1)-C(6)-C(7)	80.82(16)	C(30)-N(23)-C(27)-C(28)	148.47(12)
C(2)-N(1)-C(6)-C(7)	-147.24(14)	C(24)-N(23)-C(27)-C(28)	-79.89(15)
Sn(5)-N(1)-C(6)-C(7)	-33.42(14)	Sn(33)-N(23)-C(27)-C(28)	34.43(13)
N(1)-C(6)-C(7)-C(8)	55.93(18)	N(23)-C(27)-C(28)-C(29)	-57.82(16)
C(6)-C(7)-C(8)-Sn(5)	-48.25(15)	C(27)-C(28)-C(29)-Sn(33)	50.34(15)
C(6)-N(1)-C(9)-C(10)	-148.00(13)	C(27)-N(23)-C(30)-C(31)	-80.70(15)
C(2)-N(1)-C(9)-C(10)	80.09(16)	C(24)-N(23)-C(30)-C(31)	147.79(12)
Sn(5)-N(1)-C(9)-C(10)	-33.65(14)	Sn(33)-N(23)-C(30)-C(31)	33.24(13)
N(1)-C(9)-C(10)-C(11)	57.56(18)	N(23)-C(30)-C(31)-C(32)	-57.88(17)
C(9)-C(10)-C(11)-Sn(5)	-50.49(16)	C(30)-C(31)-C(32)-Sn(33)	51.82(15)
C(14)-O(13)-C(12)-Sn(5)	178.71(9)	C(36)-O(35)-C(34)-Sn(33)	178.32(9)
C(12)-O(13)-C(14)-C(15)	175.33(11)	C(34)-O(35)-C(36)-C(37)	-176.17(11)
O(13)-C(14)-C(15)-C(16)	162.92(12)	O(35)-C(36)-C(37)-C(38)	-161.16(12)
O(13)-C(14)-C(15)-C(22)	-18.58(19)	O(35)-C(36)-C(37)-C(42)	18.86(18)
C(22)-C(15)-C(16)-C(17)	-0.8(2)	C(42)-C(37)-C(38)-C(39)	0.6(2)
C(14)-C(15)-C(16)-C(17)	177.72(13)	C(36)-C(37)-C(38)-C(39)	-179.42(13)
C(15)-C(16)-C(17)-C(18)	-0.1(2)	C(37)-C(38)-C(39)-C(40)	0.3(2)
C(20)-O(19)-C(18)-C(17)	1.4(2)	C(44)-O(43)-C(40)-C(39)	0.5(2)
C(20)-O(19)-C(18)-C(21)	-177.55(13)	C(44)-O(43)-C(40)-C(41)	-179.54(14)
C(16)-C(17)-C(18)-O(19)	-177.72(13)	C(38)-C(39)-C(40)-O(43)	178.80(13)
C(16)-C(17)-C(18)-C(21)	1.1(2)	C(38)-C(39)-C(40)-C(41)	-1.1(2)
O(19)-C(18)-C(21)-C(22)	177.82(13)	O(43)-C(40)-C(41)-C(42)	-178.83(13)
C(17)-C(18)-C(21)-C(22)	-1.1(2)	C(39)-C(40)-C(41)-C(42)	1.1(2)
C(18)-C(21)-C(22)-C(15)	0.1(2)	C(40)-C(41)-C(42)-C(37)	-0.2(2)
C(16)-C(15)-C(22)-C(21)	0.9(2)	C(38)-C(37)-C(42)-C(41)	-0.6(2)
C(14)-C(15)-C(22)-C(21)	-177.65(13)	C(36)-C(37)-C(42)-C(41)	179.40(13)

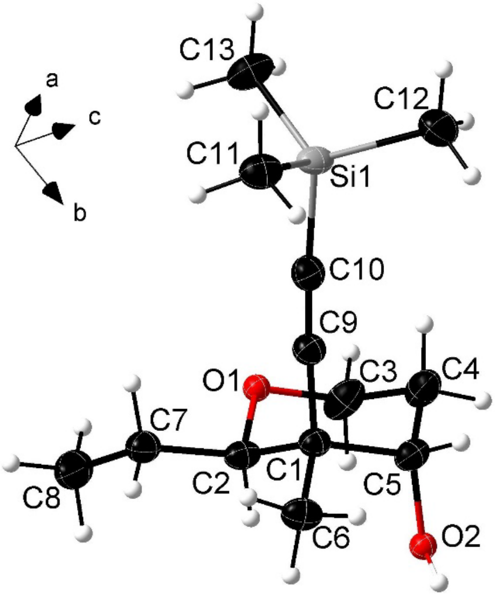

Molecular structure of pyran **49**. Displacement ellipsoids are displayed at the 50% probability level with hydrogen atoms being shown as spheres of arbitrary size.

Table S28. Crystal data and structure refinement for pyran **49**.

Identification code	2520699	
Empirical formula	$C_{13}H_{24}O_2Si$	
Formula weight	240.41	
Temperature	130(2) K	
Wavelength	0.71073 Å	
Crystal system	Monoclinic	
Space group	C2	
Unit cell dimensions	a = 19.8650(8) Å	α = 90°.
	b = 6.1756(3) Å	β = 93.479(4)°.
	c = 24.3605(11) Å	γ = 90°.
Volume	2983.0(2) $Å^3$	
Z	8	
Density (calculated)	1.071 Mg/m^3	
Absorption coefficient	0.145 mm^{-1}	
F(000)	1056	
Crystal size	0.320 x 0.040 x 0.030 mm^3	
Theta range for data collection	2.265 to 26.399°.	
Index ranges	-24<=h<=22, -7<=k<=7, -30<=l<=30	
Reflections collected	13983	
Independent reflections	5861 [R(int) = 0.0498]	

Completeness to theta = 25.242°	99.8 %
Absorption correction	Semi-empirical from equivalents
Max. and min. transmission	1.0000 and 0.5306
Refinement method	Full-matrix least-squares on F^2
Data / restraints / parameters	5861 / 3 / 305
Goodness-of-fit on F^2	1.019
Final R indices [I>2sigma(I)]	R1 = 0.0497, wR^2 = 0.1203
R indices (all data)	R1 = 0.0589, wR^2 = 0.1242
Absolute structure parameter	-0.03(9)
Extinction coefficient	n/a
Largest diff. peak and hole	0.425 and -0.357 e.Å^{-3}

Table S29. Atomic coordinates (x10^4) and equivalent isotropic displacement parameters (Å^{2}x10^3) for pyran **49**. U(eq) is defined as one third of the trace of the orthogonalized Uij tensor.

atom	X	Y	Z	U(eq)
Si(1)	9022(1)	543(2)	3733(1)	25(1)
Si(2)	5591(1)	6331(2)	1157(1)	33(1)
O(1)	6410(1)	989(4)	4055(1)	27(1)
O(2)	6418(1)	6526(5)	4283(1)	33(1)
O(3)	8239(1)	6880(4)	1123(1)	22(1)
O(4)	8259(1)	12429(4)	866(1)	26(1)
C(1)	7027(2)	4048(6)	3699(1)	21(1)
C(2)	6405(2)	2566(6)	3622(1)	24(1)
C(3)	6360(2)	1970(6)	4584(2)	34(1)
C(4)	6946(2)	3479(7)	4718(2)	33(1)
C(5)	6999(2)	5176(6)	4268(1)	26(1)
C(6)	7028(2)	5765(7)	3248(1)	29(1)
C(7)	6347(2)	1367(7)	3082(1)	28(1)
C(8)	5689(2)	108(7)	2998(2)	42(1)
C(9)	7653(2)	2778(6)	3700(1)	24(1)
C(10)	8185(2)	1803(6)	3707(1)	27(1)
C(11)	9402(2)	1050(7)	3065(2)	33(1)
C(12)	9541(2)	1775(8)	4307(2)	40(1)
C(13)	8948(2)	-2440(7)	3835(2)	37(1)
C(14)	7556(2)	9981(5)	1382(1)	19(1)
C(15)	8159(2)	8487(6)	1540(1)	20(1)
C(16)	8399(2)	7848(6)	612(1)	25(1)
C(17)	7833(2)	9332(6)	395(1)	24(1)
C(18)	7685(2)	11045(6)	816(1)	22(1)
C(19)	7473(2)	11739(6)	1813(1)	29(1)
C(20)	8106(2)	7289(6)	2076(1)	27(1)
C(21)	8726(2)	5932(7)	2240(2)	35(1)
C(22)	6928(2)	8698(6)	1316(1)	25(1)
C(23)	6403(2)	7728(7)	1265(2)	30(1)
C(24)	5406(3)	4855(13)	1786(2)	85(3)
C(25)	5654(2)	4373(10)	595(2)	49(1)
C(26)	4946(2)	8411(10)	979(3)	95(3)

Table S30. Bond lengths [Å] for pyran **49**.

atoms	bond length [Å]	atoms	bond length [Å]
Si(1)-C(10)	1.835(4)	C(1)-C(2)	1.539(5)
Si(1)-C(12)	1.850(4)	C(1)-C(5)	1.555(5)
Si(1)-C(11)	1.861(3)	C(2)-C(7)	1.509(5)
Si(1)-C(13)	1.866(4)	C(3)-C(4)	1.511(6)
Si(2)-C(23)	1.835(4)	C(4)-C(5)	1.524(5)
Si(2)-C(25)	1.836(5)	C(7)-C(8)	1.524(5)
Si(2)-C(24)	1.840(5)	C(9)-C(10)	1.215(5)
Si(2)-C(26)	1.846(6)	C(14)-C(22)	1.478(4)
O(1)-C(3)	1.432(4)	C(14)-C(19)	1.527(5)
O(1)-C(2)	1.434(4)	C(14)-C(15)	1.543(4)
O(2)-C(5)	1.427(4)	C(14)-C(18)	1.561(4)
O(3)-C(16)	1.432(4)	C(15)-C(20)	1.511(5)
O(3)-C(15)	1.437(4)	C(16)-C(17)	1.521(5)
O(4)-C(18)	1.424(4)	C(17)-C(18)	1.515(5)
C(1)-C(9)	1.470(5)	C(20)-C(21)	1.523(5)
C(1)-C(6)	1.528(5)	C(22)-C(23)	1.202(5)

Table S31. Bond angles [°] for pyran **49**.

atoms	bond angle [°]	atoms	bond angle [°]
C(10)-Si(1)-C(12)	108.31(17)	C(3)-C(4)-C(5)	110.8(3)
C(10)-Si(1)-C(11)	108.23(16)	O(2)-C(5)-C(4)	107.0(3)
C(12)-Si(1)-C(11)	110.80(18)	O(2)-C(5)-C(1)	110.9(3)
C(10)-Si(1)-C(13)	110.16(18)	C(4)-C(5)-C(1)	109.9(3)
C(12)-Si(1)-C(13)	110.5(2)	C(2)-C(7)-C(8)	112.8(3)
C(11)-Si(1)-C(13)	108.79(19)	C(10)-C(9)-C(1)	177.3(4)
C(23)-Si(2)-C(25)	108.53(18)	C(9)-C(10)-Si(1)	175.3(3)
C(23)-Si(2)-C(24)	109.3(2)	C(22)-C(14)-C(19)	109.3(3)
C(25)-Si(2)-C(24)	108.7(3)	C(22)-C(14)-C(15)	110.2(3)
C(23)-Si(2)-C(26)	107.3(2)	C(19)-C(14)-C(15)	111.5(3)
C(25)-Si(2)-C(26)	111.2(3)	C(22)-C(14)-C(18)	108.2(3)
C(24)-Si(2)-C(26)	111.7(4)	C(19)-C(14)-C(18)	109.8(3)
C(3)-O(1)-C(2)	112.1(3)	C(15)-C(14)-C(18)	107.8(2)
C(16)-O(3)-C(15)	111.5(2)	O(3)-C(15)-C(20)	106.9(3)
C(9)-C(1)-C(6)	109.4(3)	O(3)-C(15)-C(14)	110.7(2)
C(9)-C(1)-C(2)	110.8(3)	C(20)-C(15)-C(14)	114.5(3)
C(6)-C(1)-C(2)	111.2(3)	O(3)-C(16)-C(17)	110.8(3)
C(9)-C(1)-C(5)	108.3(3)	C(18)-C(17)-C(16)	110.8(3)
C(6)-C(1)-C(5)	109.4(3)	O(4)-C(18)-C(17)	106.8(2)
C(2)-C(1)-C(5)	107.6(3)	O(4)-C(18)-C(14)	110.6(2)
O(1)-C(2)-C(7)	107.7(3)	C(17)-C(18)-C(14)	110.6(3)
O(1)-C(2)-C(1)	110.2(3)	C(15)-C(20)-C(21)	113.4(3)
C(7)-C(2)-C(1)	114.6(3)	C(23)-C(22)-C(14)	177.4(4)
O(1)-C(3)-C(4)	111.1(3)	C(22)-C(23)-Si(2)	177.0(4)

Table S32. Anisotropic displacement parameters (Å^{2}x 10^3) for pyran **49**. The anisotropic displacement factor exponent takes the form: -2p^2[h^2 a^{*2}U^{11} + ... + 2hka*b*U^{12}]

Atom	U^{11}	U^{22}	U^{33}	U^{23}	U^{13}	U^{12}
Si(1)	16(1)	24(1)	36(1)	2(1)	1(1)	2(1)
Si(2)	16(1)	38(1)	44(1)	-9(1)	8(1)	-11(1)
O(1)	34(1)	19(1)	31(1)	2(1)	10(1)	-1(1)
O(2)	34(1)	17(1)	48(2)	2(1)	14(1)	3(1)
O(3)	27(1)	15(1)	26(1)	0(1)	6(1)	-1(1)
O(4)	21(1)	12(1)	45(2)	-2(1)	8(1)	-5(1)
C(1)	18(2)	21(2)	25(2)	0(1)	2(1)	1(1)
C(2)	18(2)	20(2)	33(2)	3(1)	3(1)	0(1)
C(3)	47(2)	22(2)	33(2)	2(2)	16(2)	-2(2)
C(4)	47(2)	24(2)	28(2)	-1(2)	5(2)	2(2)
C(5)	25(2)	21(2)	33(2)	-2(2)	4(1)	1(2)
C(6)	26(2)	25(2)	35(2)	9(2)	4(1)	-1(2)
C(7)	24(2)	25(2)	34(2)	-1(2)	-1(1)	1(2)
C(8)	30(2)	34(3)	60(3)	-10(2)	-7(2)	-1(2)
C(9)	20(2)	25(2)	28(2)	0(1)	1(1)	0(1)
C(10)	24(2)	27(2)	31(2)	-1(2)	1(1)	-3(2)
C(11)	24(2)	32(2)	43(2)	4(2)	6(1)	1(2)
C(12)	33(2)	39(3)	47(2)	-2(2)	-9(2)	2(2)
C(13)	38(2)	25(2)	49(2)	4(2)	13(2)	4(2)
C(14)	14(1)	18(2)	26(2)	0(1)	2(1)	-3(1)
C(15)	19(2)	18(2)	24(2)	-3(1)	1(1)	-4(1)
C(16)	30(2)	21(2)	26(2)	0(1)	7(1)	2(1)
C(17)	27(2)	21(2)	24(2)	3(1)	1(1)	-2(1)
C(18)	16(1)	17(2)	33(2)	3(1)	0(1)	-2(1)
C(19)	27(2)	24(2)	35(2)	-6(2)	7(1)	1(2)
C(20)	31(2)	22(2)	28(2)	0(1)	1(1)	-2(2)
C(21)	38(2)	27(2)	38(2)	6(2)	-8(1)	1(2)
C(22)	18(2)	28(2)	29(2)	0(2)	3(1)	-3(1)
C(23)	19(2)	34(2)	37(2)	-2(2)	4(1)	-6(2)
C(24)	84(4)	128(7)	45(3)	-7(3)	18(3)	-76(4)
C(25)	31(2)	65(3)	52(3)	-20(2)	6(2)	-11(2)
C(26)	17(2)	49(4)	217(8)	-22(5)	-8(3)	-3(2)

Table S33. Bond angles [°] for pyran **49**

atoms	torsion angle [°]	atoms	torsion angle [°]
C(3)-O(1)-C(2)-C(7)	-171.1(3)	C(16)-O(3)-C(15)-C(20)	-171.0(2)
C(3)-O(1)-C(2)-C(1)	63.3(3)	C(16)-O(3)-C(15)-C(14)	63.7(3)
C(9)-C(1)-C(2)-O(1)	58.9(3)	C(22)-C(14)-C(15)-O(3)	59.9(3)
C(6)-C(1)-C(2)-O(1)	-179.2(3)	C(19)-C(14)-C(15)-O(3)	-178.5(2)
C(5)-C(1)-C(2)-O(1)	-59.3(3)	C(18)-C(14)-C(15)-O(3)	-57.9(3)
C(9)-C(1)-C(2)-C(7)	-62.8(4)	C(22)-C(14)-C(15)-C(20)	-61.0(4)
C(6)-C(1)-C(2)-C(7)	59.2(4)	C(19)-C(14)-C(15)-C(20)	60.6(4)
C(5)-C(1)-C(2)-C(7)	179.0(3)	C(18)-C(14)-C(15)-C(20)	-178.8(3)
C(2)-O(1)-C(3)-C(4)	-60.6(4)	C(15)-O(3)-C(16)-C(17)	-61.8(3)
O(1)-C(3)-C(4)-C(5)	55.4(4)	O(3)-C(16)-C(17)-C(18)	56.3(4)
C(3)-C(4)-C(5)-O(2)	66.8(4)	C(16)-C(17)-C(18)-O(4)	67.6(3)
C(3)-C(4)-C(5)-C(1)	-53.7(4)	C(16)-C(17)-C(18)-C(14)	-52.8(3)
C(9)-C(1)-C(5)-O(2)	177.1(3)	C(22)-C(14)-C(18)-O(4)	175.8(3)
C(6)-C(1)-C(5)-O(2)	57.8(3)	C(19)-C(14)-C(18)-O(4)	56.6(3)
C(2)-C(1)-C(5)-O(2)	-63.1(4)	C(15)-C(14)-C(18)-O(4)	-65.1(3)
C(9)-C(1)-C(5)-C(4)	-64.9(4)	C(22)-C(14)-C(18)-C(17)	-66.1(3)
C(6)-C(1)-C(5)-C(4)	175.9(3)	C(19)-C(14)-C(18)-C(17)	174.7(3)
C(2)-C(1)-C(5)-C(4)	54.9(4)	C(15)-C(14)-C(18)-C(17)	53.1(3)
O(1)-C(2)-C(7)-C(8)	63.0(4)	O(3)-C(15)-C(20)-C(21)	60.5(4)
C(1)-C(2)-C(7)-C(8)	-174.0(3)	C(14)-C(15)-C(20)-C(21)	-176.6(3)

Table S34. Hydrogen bonds and angles for pyran **49** [Å and °].

D-H...A	d(D-H) [Å]	d(H...A) [Å]	d(D...A) [Å]	<(DHA) [°]
O(2)-H(2O)...O(1)#1	0.837(14)	2.03(2)	2.811(4)	156(4)
O(4)-H(4O)...O(3)#1	0.838(13)	2.06(2)	2.820(3)	150(4)

Symmetry transformation used to generate equivalent atoms: #1 x,y+1,z

Literature

[1] K. Gerth, P. Washausen, G. Höfle, H. Irschik, H. Reichenbach, "The Jerangolids: A Family of New Antifungal Compounds from Sorangium cellulosum (Myxobacteria). Production, Physico-chemical and Biological Properties of Jerangolid A." *J. Antibiot.* **1996**, *49*, 71–75.

[2] H. Reichenbach, G. Höfle, K. Gerth, P. Washausen, "Heterocyclic compounds obtainable from sorangium cellulosum basteria, their preparation process, and agents containing these compounds" *P. PCT Int. Appl. DE 19607702* **1997**.

[19] J. Pospíšil, I. E. Markó, "Total Synthesis of Jerangolid D" *J. Am. Chem. Soc.* **2007**, *129*, 3516–3517.

[20] S. Hanessian, T. Focken, R. Oza, "Total Synthesis of Jerangolid A" *Org. Lett.* **2010**, *12*, 3172–3175.

[21] F. Lindner, S. Friedrich, F. Hahn, "Total Synthesis of Complex Biosynthetic Late-Stage Intermediates and Bioconversion by a Tailoring Enzyme from Jerangolid Biosynthesis" *J. Org. Chem.* **2018**, *83*, 14091–14101.

[22] J. Schug, B. Morgenstern, J. Jauch, "Total Synthesis of Jerangolid B via sp^3 –sp^2 Stille Coupling" *Org. Lett.* **2025**, *27*, 7719–7726.

[23] D. Könning, W. Hiller, M. Christmann, "One-pot oxidation/isomerization of Z-allylic alcohols with oxygen as stoichiometric oxidant" *Org. Lett.* **2012**, *14*, 5258–5261.

[26] M. Schlosser, T. Jenny, Y. Guggisberg, "Monomeric Formaldehyde in Ethereal Solution" *Synlett* **1990**, *1990*, 704–704.

[50] M. S. Jensen, C. Yang, Y. Hsiao, N. Rivera, K. M. Wells, J. Y. L. Chung, N. Yasuda, D. L. Hughes, P. J. Reider, "Synthesis of an Anti-Methicillin-Resistant *Staphylococcus aureus* (MRSA) Carbapenem via Stannatrane-Mediated Stille Coupling" *Org. Lett.* **2000**, *2*, 1081–1084.

[59] M.-X. Zhao, Y. Shi, "Practical Synthesis of an L -Fructose-Derived Ketone Catalyst for Asymmetric Epoxidation of Olefins" *J. Org. Chem.* **2006**, *71*, 5377–5379.

[85] G. M. Sheldrick, "*SHELXT* – Integrated space-group and crystal-structure determination" *Acta Crystallogr A Found Adv* **2015**, *71*, 3–8.

[86] G. M. Sheldrick, "Crystal structure refinement with *SHELXL*" *Acta Crystallogr C Struct Chem* **2015**, *71*, 3–8.

[87] C. B. Hübschle, G. M. Sheldrick, B. Dittrich, "*ShelXle* : a Qt graphical user interface for *SHELXL*" *J Appl Crystallogr* **2011**, *44*, 1281–1284.

[88] A. L. Spek, "Structure validation in chemical crystallography" *Acta Crystallogr D Biol Crystallogr* **2009**, *65*, 148–155.
